# Suppressing carboxylate nucleophilicity with inorganic salts enables selective electrocarboxylation without sacrificial anodes†

**DOI:** 10.1039/d1sc02413b

**Published:** 2021-08-16

**Authors:** Nathan Corbin, Deng-Tao Yang, Nikifar Lazouski, Katherine Steinberg, Karthish Manthiram

**Affiliations:** Department of Chemical Engineering, Massachusetts Institute of Technology 77 Massachusetts Avenue Cambridge Massachusetts 02139 USA karthish@mit.edu

## Abstract

Although electrocarboxylation reactions use CO_2_ as a renewable synthon and can incorporate renewable electricity as a driving force, the overall sustainability and practicality of this process is limited by the use of sacrificial anodes such as magnesium and aluminum. Replacing these anodes for the carboxylation of organic halides is not trivial because the cations produced from their oxidation inhibit a variety of undesired nucleophilic reactions that form esters, carbonates, and alcohols. Herein, a strategy to maintain selectivity without a sacrificial anode is developed by adding a salt with an inorganic cation that blocks nucleophilic reactions. Using anhydrous MgBr_2_ as a low-cost, soluble source of Mg^2+^ cations, carboxylation of a variety of aliphatic, benzylic, and aromatic halides was achieved with moderate to good (34–78%) yields without a sacrificial anode. Moreover, the yields from the sacrificial-anode-free process were often comparable or better than those from a traditional sacrificial-anode process. Examining a wide variety of substrates shows a correlation between known nucleophilic susceptibilities of carbon–halide bonds and selectivity loss in the absence of a Mg^2+^ source. The carboxylate anion product was also discovered to mitigate cathodic passivation by insoluble carbonates produced as byproducts from concomitant CO_2_ reduction to CO, although this protection can eventually become insufficient when sacrificial anodes are used. These results are a key step toward sustainable and practical carboxylation by providing an electrolyte design guideline to obviate the need for sacrificial anodes.

## Abstract 2

Selective electrocarboxylation of nucleophilically susceptible organic halides without sacrificial anodes is enabled by inorganic salt additives, which suppress the nucleophilicity of anions in the electrolyte.

## Graphical abstract

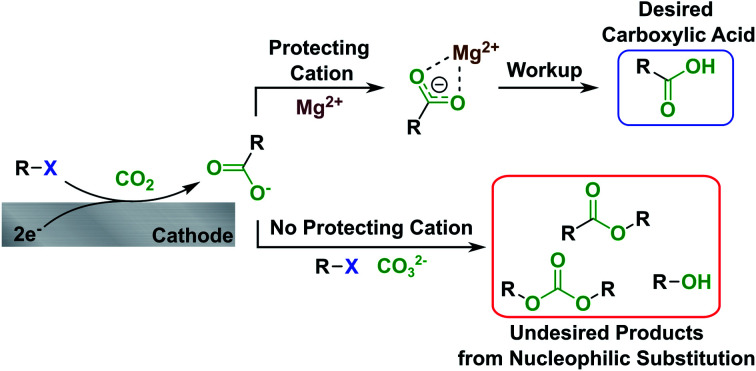

## Introduction

Incorporating CO_2_ into organic molecules represents a promising, sustainable strategy to synthesize value-added carboxylic acids.^1–3^ Compared to the traditional oxidative^4–6^ and carbonylation^7^ approaches used to make carboxylic acids, carboxylation with CO_2_ is endowed with several advantageous synthetic properties such as extending the carbon chain length, tolerating easily oxidized functional groups, and using an abundant, renewable, and non-toxic carbon source. Traditionally, highly reactive organometallic species such as Grignard and organolithium reagents have been used to directly react with CO_2_ to form carboxylates, but the difficulty in preparing and handling these reagents and their limited functional group tolerance are major drawbacks.^8,9^ Many alternative thermocatalytic, photocatalytic, and electrocatalytic processes have been investigated to provide milder reaction conditions, greater functional group tolerance, and better scalability. Compared to thermocatalytic^10–12^ and photocatalytic^13,14^ approaches which require homogeneous (photo)catalysts and stoichiometric chemical reductants, electrochemical approaches to carboxylate carbon–halogen bonds can use heterogeneous catalysts (cathodes) and reductants (electrons), albeit with the extra requirement of a conductive electrolyte salt. The ability to tune the reduction potential *via* the externally applied voltage allows greater selectivity in reducing particular functional groups,^15^ and the use of electricity facilitates incorporation of renewable sources of energy to drive the reaction.^16^ The heterogeneous nature of the catalyst and reductant can ease post-reaction separations, especially by eliminating the need to recycle catalyst or remove toxic metals.

Traditionally, electrochemical carboxylation has been performed with sacrificial metal anodes.^15,17,18^ These anodes are chosen such that the metal anode is preferentially oxidized over any other species in solution, thereby avoiding unwanted oxidation of the substrate or product.^19^ Sacrificial anodes also simplify the reaction setup by allowing undivided cells to be used, obviating the need to find membranes or separators that are stable in organic media. However, sacrificial anodes limit the sustainability of the process, as the processes used to regenerate these anodes (often magnesium or aluminum) are energy-intensive and incur their own CO_2_ emissions.^20–23^ Using metals that can be more easily recycled such as nickel or zinc poses an electroplating risk if the metal cation reduces more easily than the carboxylation substrate,^19^ resulting in parasitic current and possible degradation of the cathodic electrocatalyst. Because the anode is gradually consumed over time, possibly in an uneven fashion, the development of continuous electrocarboxylation processes is complicated due to the need to periodically replace the anode.^17^ A specific drawback of using sacrificial anodes in electrocarboxylation processes is related to the accompanying CO_2_ reduction, which produces carbonate, oxalate, or both anions in aprotic media.^24–26^ These anions can form insoluble salts with the cation from the sacrificial anode, leading to cathodic passivation, increased cell voltage and resistive heating, and eventual stoppage of the electrochemical reaction.^27–30^

Given the sustainability and operability drawbacks of using sacrificial anodes, numerous strategies have been developed to avoid their use.^17,31^ All of these strategies rely on alternative oxidation chemistries including oxidation of oxalate,^32,33^ trimethylamine,^34^ acetonitrile (MeCN),^35^*N*,*N*-dimethylformamide (DMF),^36^ halides,^37–39^ water,^40,41^ H_2_,^42^ conjugated dienes,^43^ and alcohols.^44^ Several of these alternative oxidation chemistries have also been accompanied by improved cell designs to achieve better carboxylation selectivities.^40,45^ While simply changing the anodic chemistry has worked well for carboxylating substrates without good leaving groups such as olefins and ketones, many organic halides have proved to be more challenging to carboxylate without a sacrificial anode. Organic halides represent an important class of carboxylation substrates due to the wide variety of carbon–halide bonds that can be carboxylated (*e.g.* benzyl, aryl, alkyl, allyl);^46–52^ in addition, electrocarboxylation of secondary benzylic halides has been envisioned to lead to more sustainable syntheses of pharmaceutically relevant arylacetic acids.^53,54^ Attempts to carboxylate many organic halides without sacrificial anodes have led to product selectivity issues because the carboxylate product is a nucleophile that can undergo nucleophilic substitution with the organic halide substrate to form an ester.^40,55,56^ In fact, one of the primary reasons sacrificial anodes are so widely used for electrocarboxylation is to prevent nucleophilic side reactions with the organic halide substrate.^18,19^

In this work, a synthetic strategy to enable selective carboxylation of organic halides without using sacrificial anodes is presented. The addition of a soluble inorganic salt such as magnesium bromide (MgBr_2_) significantly reduces the nucleophilicity of carboxylate and carbonate anions, allowing selective electrocarboxylation to proceed. For several substrates, similar, if not better, carboxylation yields are demonstrated without a sacrificial anode in comparison to a traditional sacrificial-anode process. The use of an inorganic salt also fixes the amount of cations in solution, which helps to mitigate cathodic passivation from inorganic carbonate precipitation; in contrast, the oxidation of sacrificial anodes continuously increases the amount of metal cations in solution. These results present a viable design strategy for maintaining carboxylate selectivity at the cathode while increasing the flexibility to incorporate more sustainable or economical anodic reactions for electrocarboxylation processes.

## Results and discussion

### Effect of magnesium cations on product distribution

**Scheme 1:**
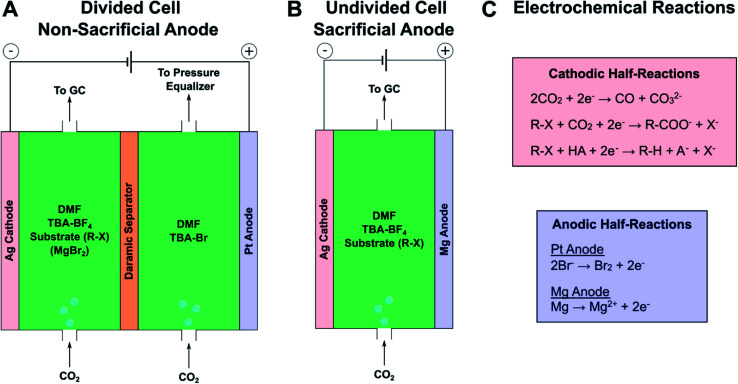
Electrochemical cell configurations for initial product distribution experiments at low substrate conversion. (A) Divided cell setups with and without MgBr_2_. A pressure equalizer, composed of a column of dimethyl sulfoxide (DMSO) in a centrifuge tube, was necessary to prevent flow through the Daramic separator due to the pressure imposed by the in-line gas chromatograph (GC). (B) Sacrificial anode setup in an undivided cell. (C) Summary of key half-reactions occurring at each electrode. The substrate is indicated as a generic organic halide (R–X), but for these experiments, the model substrate was 1-bromo-3-phenylpropane (**1b**). Specific compositions for each type of experiment are given below in the caption of Fig. 1. See Fig. S1–S3† for diagrams of the cell components and assembly procedures.

To demonstrate the effect of an inorganic salt on product selectivity, electrocarboxylation was performed using 1-bromo-3-phenylpropane (**1b**) as a model substrate and anhydrous MgBr_2_ as a source of Mg^2+^. MgBr_2_ was used because it can be purchased as a low-cost, anhydrous salt and is reasonably soluble in aprotic polar solvents.^57,58^ Initially, the cathodic electrochemical reactions and homogeneous reactions were investigated at low substrate conversions to understand the scope of chemistries occurring in the system and the general effect of magnesium cations; this information was used later to optimize reaction conditions and obtain higher product yields (*vide infra*). To this end, mass and charge balances were performed for three reaction configurations to observe the effects of magnesium cations and type of anodic reaction (Scheme 1). Silver was chosen as the cathode because studies have consistently found it to be the most active simple metal catalyst for reductively cleaving carbon–halide bonds,^59^ and it has been routinely used in electrocarboxylation studies.^48,51,60^ DMF was chosen as the solvent, since it has also been commonly used in previous electrocarboxylation studies and can be readily separated into the aqueous phase during workup.^34,61^ Based on condition optimization described below, silver and DMF turned out to be optimal choices for the cathode and solvent, respectively. Tetra-*n*-butylammonium tetrafluoroborate (TBA-BF_4_) was used as an inert supporting electrolyte, and TBA-Br was used to perform bromide oxidation. Galvanostatic operation at −5 mA cm^−2^ was employed as a simple way to achieve comparable reaction rates across all three configurations; more precise cathodic potential control *via* a reference electrode was not necessary for the level of analysis performed here. For the reactions involving a non-sacrificial Pt anode, a divided cell was used to reduce the impact of the anodic bromide oxidation on the cathodic chemistry. A Daramic separator was used as a divider since it is stable in organic solvents and easily dried to eliminate moisture, in comparison with ion-exchange membranes. While not necessarily conducted at fully optimized conditions, these initial experiments provided valuable insight into the role Mg^2+^ cations play in the cathodic and solution-phase reactions.

**Fig. 1:**
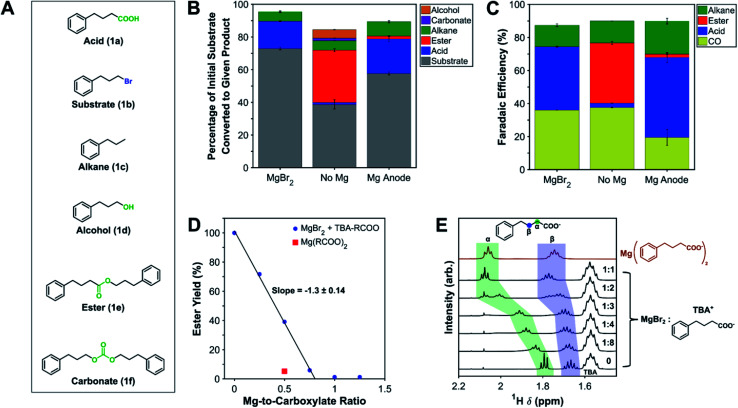
Product distributions at low substrate conversions and mechanistic studies of cation–carboxylate interactions. (A) Chemical structures of the model substrate and its various reaction products. (B) Mass balance closure and (C) charge balance closure for carboxylation of **1b** in three different setups: **MgBr**_**2**_ involved using a two-compartment cell with 0.1 M MgBr_2_, 0.1 M **1b**, and 0.1 M TBA-BF_4_ in the catholyte, a Daramic polyporous separator, and bromide oxidation on Pt as the anodic reaction (0.1 M TBA-Br); **no Mg** had the same conditions as **MgBr**_**2**_ but with no added MgBr_2_; **Mg anode** involved using a one-compartment cell with a sacrificial magnesium anode, 0.1 M **1b**, and 0.1 M TBA-BF_4_, representative of a typical electrocarboxylation reaction. In all three configurations, DMF was the solvent, 20 sccm CO_2_ was bubbled into the electrolyte at ambient pressure, a polycrystalline Ag foil was used as the cathode, and −5 mA cm^−2^ was applied for 1 h. (D) Yields of ester obtained after letting **1b** and tetra-*n*-butylammonium 4-phenylbutyrate (TBA–RCOO, TBA salt of **1a**) react for 12 h in the presence of varying amounts of MgBr_2_ in DMF, quantified by ^1^H NMR. (E) ^1^H NMR shifts of α and β protons of the TBA salt of **1a** for varying amounts of MgBr_2_ as well as the magnesium salt of **1a** in DMSO-d_6_.

The overall mass balance of the substrate can be closed reasonably well over all three systems (Fig. 1). Performing the carboxylation with a Pt anode and no Mg^2+^ source resulted in a wide product distribution, with most of the carboxylate reacting further to produce an ester (**1e**). Additionally, smaller amounts of the alcohol (**1d**) and the organic carbonate (**1f**) were formed as well, which are the result of nucleophilic attacks by carbonate (CO_3_^2−^) anions on the substrate.^62,63^ Carboxylation performed with MgBr_2_ added to the catholyte shows a much greater selectivity toward the carboxylic acid (**1a**) after workup, demonstrating the ability of Mg^2+^ cations to inhibit nucleophilicity of all species in solution. Other inorganic salts were investigated as well to assess their effectiveness in suppressing nucleophilic reactions; AlBr_3_ was found to be equally effective as MgBr_2_, while many alkali metal bromides suffered from low solubility or carbonate-precipitation problems in DMF (Fig. S9†). Carboxylation with a sacrificial Mg anode also achieved good carboxylic acid selectivity, but small amounts of ester were detectable; the ester can form at the beginning of the electrolysis when not enough Mg^2+^ cations are present since only a small amount of the anode has oxidized. The mass balance also indicates a noticeable amount of hydrogenation of the substrate, leading to the alkane (**1c**). This hydrogenation is the main competing electrochemical reaction with carboxylation and appears to result from a reaction with the solvent (*vide infra*). Small amounts of substrate dimerization (∼1%) to 1,6-diphenylhexane could be detected by gas chromatography-mass spectrometry (GC-MS) but was not calibrated and quantified.

The charge balance can also be closed well in all three systems. Carboxylation and hydrogenation are the electrochemical reactions of the substrate, while direct reductive disproportionation of CO_2_ to carbon monoxide (CO) and CO_3_^2−^ accounts for the majority of the outstanding charge.^25^ The higher faradaic efficiencies (FEs) for carboxylation and hydrogenation (*i.e.* reactions involving the substrate) in the sacrificial anode system likely stem from diffusion of the substrate through the Daramic separator in the two-compartment systems, lowering its concentration at the cathode. Together with substrate consumption, this diffusion results in time transience in the CO FEs (Fig. S10†), which combined with the fact that they are measured every five minutes, can lead to underestimation of the true CO FE. The greater time variance of the CO FEs in the **no Mg** experiment is consistent with nucleophilic reactions consuming additional substrate, as observed in the mass balance closure. These facts may account for why the total FEs are consistently under 100%. Comparing the **MgBr**_**2**_ and **no Mg** experiments, the effect of MgBr_2_ is small on the electrochemical reactions themselves, an observation further supported by a negligible interaction with **1b** (see ESI†).

^1^H NMR spectroscopy was used to gain insights into the coordinating effect of Mg^2+^ on the carboxylate and the subsequent suppression of esterification. A ^1^H NMR kinetic experiment demonstrated rapid esterification of the brominated substrate **1b** in the presence of TBA 4-phenylbutyrate (TBA salt of **1a**), with nearly full conversion to the ester after about 30 min (Fig. S11†). Fig. 1C shows the effect of titrating varying amounts of MgBr_2_ into this mixture and examining the equilibrium ester yield after 12+ h. The ester yield decreases linearly with added MgBr_2_ until almost no ester is detectable. Based on the ratio of ionic charges, each magnesium ion would be expected to coordinate with two carboxylate anions, leading to a slope of −2. The observed slope of −1.3 suggests that the bromide anions also compete for Mg^2+^ coordination, a conclusion further supported by an experiment where Mg(4-phenylbutyrate)_2_ (Mg salt of **1a**) was added, which resulted in a lower ester yield than when a 1 : 2 ratio of MgBr_2_ to TBA 4-phenylbutyrate was used. Moreover, the ^1^H NMR chemical shifts of protons along the alkyl chain of the carboxylate showed a trend of shifting downfield as more MgBr_2_ was present in solution (Fig. 1E), a trend also seen in the literature.^64,65^ The shift downfield at higher MgBr_2_ concentrations likely stems from greater electron withdrawal by Mg^2+^ coordinating to the carboxylate group, causing the protons to become deshielded. ^13^C NMR chemical shifts also change as a function of MgBr_2_ concentration, moving either upfield or downfield depending on the carbon atom identity (Fig. S12 and S13†).^65–67^ Collectively, these experiments demonstrate the coordination of Mg^2+^ cations to carboxylate groups, leading to suppression of nucleophilicity.

### Syntheses of carboxylic acids without sacrificial anodes

These product-distribution and NMR data indicated that the protecting ability of cations produced by sacrificial anodes can be mimicked by adding a soluble inorganic salt with the same cation. Moreover, the charge and mass balances revealed that the primary side reaction of the substrate in the presence of protecting cations is hydrogenation. As a first step toward demonstrating the synthetic ability of the sacrificial-anode-free system, reaction conditions were screened in two-compartment cells to optimize the acid-to-alkane ratio (AAR). As shown in Fig. S14,† the amount of MgBr_2_ and TBA-BF_4_ supporting electrolyte had little effect on the AAR. A lower current density resulted in a lower AAR, while a higher current density had little effect, suggesting that the cathodic voltage needs to remain reductive enough but does not need to be precisely controlled for good selectivity.

The substrate concentration and solvent identity had the largest effects on the AAR. Out of DMF, dimethyl sulfoxide (DMSO), MeCN, and propylene carbonate, DMF had the highest AAR, followed by DMSO. MeCN and propylene carbonate had poor carboxylation selectivities, possibly due to their higher acidities. Deuterium labelling using DMSO-d_6_ revealed that the alkane side product does incorporate hydrogen atoms from the solvent (see ESI†). Interestingly, higher substrate concentrations led to lower AARs, with the carboxylation rate being unaffected by substrate concentration. Such behavior indicates the rate determining steps for carboxylation and hydrogenation are different, although further work is needed to gain a more detailed understanding of the reaction mechanism at the cathode. To validate the use of silver as the cathode, copper and gold, which have some of the highest activities for reductively cleaving carbon–halide bonds aside from silver,^59^ were tested. These tests resulted in lower carboxylation FEs, which would make synthesis more time consuming, if not more challenging (Fig. S15†), so silver remained the cathode material of choice.

With an understanding of how to achieve high AARs, further parameter screening was performed to enable high-yield synthesis of carboxylic acids with a non-sacrificial anodic reaction. For the goal of obtaining high acid yields, one-compartment cells were found to be preferable to two-compartment ones for obtaining high substrate conversion and minimizing esterification. In fact, even when both chambers were filled with 0.1 M MgBr_2_, esterification occurred in the two-compartment cells. These lower yields may be partially explained by substrate and MgBr_2_ diffusion through the separator to the anolyte. Nearly equal concentrations of the alkane product **1c** were detected in the catholyte and anolyte after electrolysis in divided cells, suggesting that diffusion through the physical separator is significant over the timespan of these high-conversion experiments (Tables S4 and S5†). A suitable oxidation reaction needed to be selected which avoided the use of a sacrificial anode. Bromide oxidation was chosen over tertiary amine oxidation and ferrocene oxidation for its simplicity and relatively low interference with the cathodic chemistry since it does not produce protons upon oxidation (Fig. S16B†). An important consideration is that in an undivided cell, the bromine produced at the anode can reduce to bromide at the cathode, resulting in parasitic current. For this reason, the current density was re-optimized. While a range of current densities appeared suitable (Fig. S16A†), reaction conditions of −20 mA cm^−2^ for 3.5 h were selected. The cathode–anode separation distance and electrolyte convection help govern the rate of bromine transport across the cell, so for cells with different cathode–anode distances than the ones used in this study (0.5 in.), the current density and reaction time may need to be re-optimized. Under these operating conditions, an optimal yield of 63% was obtained for **1a**.

**Scheme 2:**
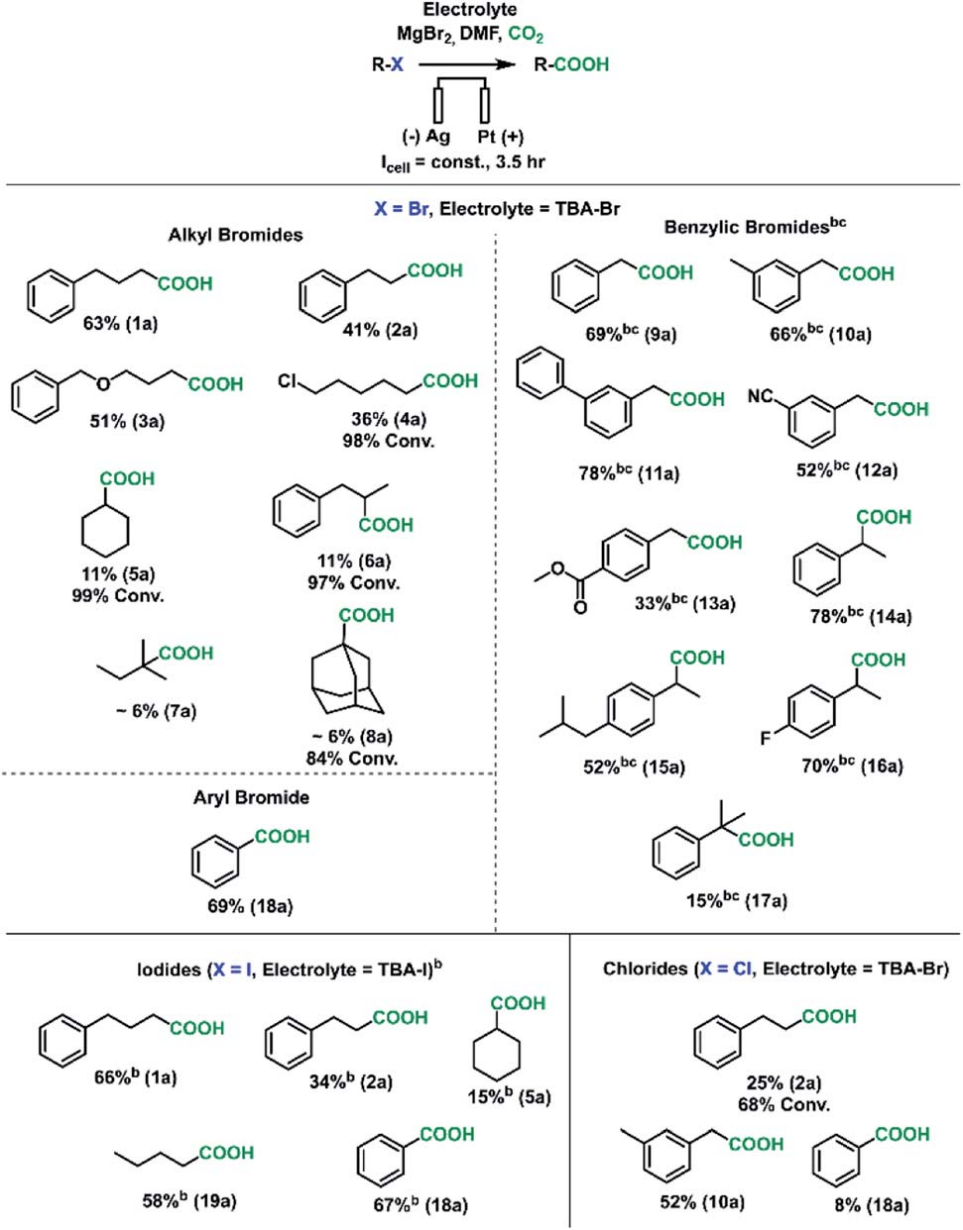
Substrate scope for the sacrificial-anode-free electrochemical carboxylation of organic halides. ^a^Standard reaction conditions: 100 mM electrolyte, 100 mM substrate, 100 mM MgBr_2_, silver cathode, platinum anode, 20 sccm CO_2_, 2.2 mL DMF, −20 mA cm^−2^ for 3.5 h. TBA-Br was used for chlorinated substrates because bromide oxidizes more readily than chloride, and only a small amount of chloride was replaced by bromide (<1% for the alkyl chloride, ∼4% for the benzylic chloride). Yields are referenced to the initial amount of substrate and were calculated from ^1^H NMR spectroscopy using either 1,3,5-trimethoxybenzene or ethylene carbonate as internal standards. ^b^−15 mA cm^−2^ instead of −20 mA cm^−2^. ^c^150 mM MgBr_2_ instead of 100 mM MgBr_2_.

A wide range of different organic halides were used as substrates to demonstrate the synthetic utility of this sacrificial-anode-free electrocarboxylation process (Scheme 2). Given the wide variation in reduction potentials and nucleophilic susceptibilities of these substrates, current densities and MgBr_2_ loadings did need to be adjusted for some substrate classes. Benzylic bromides and all iodides were run at −15 mA cm^−2^ instead of −20 mA cm^−2^ (corresponding to less reductive cathode potentials) to approximately keep the carboxylation rate constant, as these substrates have a higher tendency to be reduced compared to aliphatic bromides (see Experimental details in ESI† for **1a** and **9a**).^68–70^ Moreover, 150 mM MgBr_2_ (1.5 eq.) was used in experiments with benzylic bromides due to their high susceptibility to S_N_2 reactions.^71–73^ For primary alkyl bromides and iodides, moderate to good yields (34–66%) were obtained; the primary alkyl chloride tested had a low yield of **2a**. GC-FID analysis found a large amount of unreacted substrate (∼70% conversion), which is in agreement with the more reductive potentials needed to cleave alkyl-chloride bonds. For the difunctionalized substrate 1-bromo-5-chloropentane (**4b**), smaller amounts of 1-hexanoic acid and 1,7-heptanedioic acid were found, a result of some reduction of the carbon–chloride bond.

As the degree of substitution of the halide-bearing carbon atom increased, the carboxylation yields decreased, with tertiary bromides having very low yields. GCMS-FID analysis revealed lower substrate conversions and lower AARs for tertiary alkyl halides. Given that several studies have implicated the presence of a carbanion intermediate in the reduction of aliphatic carbon–halide bonds,^74,75^ we hypothesize the yield trend can be rationalized by the greater instability of carbanions with greater degrees of carbon substitution. The inductive electron donation from these surrounding carbon atoms increase the negative charge of the carbanion, making it more unstable. The more reactive tertiary carbanions would react more quickly with solvent molecules to abstract a proton, resulting in the lower observed AARs. While future work is needed to improve yields for some of these substrates, it is worth noting that alkyl halides have rarely been carboxylated electrochemically, and the yields found here are comparable to those found from other studies using sacrificial anodes.^46,51,52,76^

To further broaden the substrate scope, benzylic and aromatic halides were investigated. Except for chlorobenzene, all benzylic and aryl halides tested gave moderate to good (52–78%) carboxylate yields. Importantly, secondary benzylic bromides were amenable to this carboxylation protocol, with the carboxylation of **17b** yielding ibuprofen with a moderate yield. These results suggest this sacrificial-anode free protocol could be useful for the synthesis of pharmaceutically relevant aryl acetic acids such as NSAIDs.^33^ The tolerances to nitrile, ester, and alkyl chloride groups are notable, as these are usually reactive toward Grignard reagents (ESI†). Overall, with a few substrate-dependent procedural adjustments, a wide variety of organic halides could be carboxylated in a sacrificial-anode-free process.

### Comparison to sacrificial-anode carboxylation

**Fig. 2:**
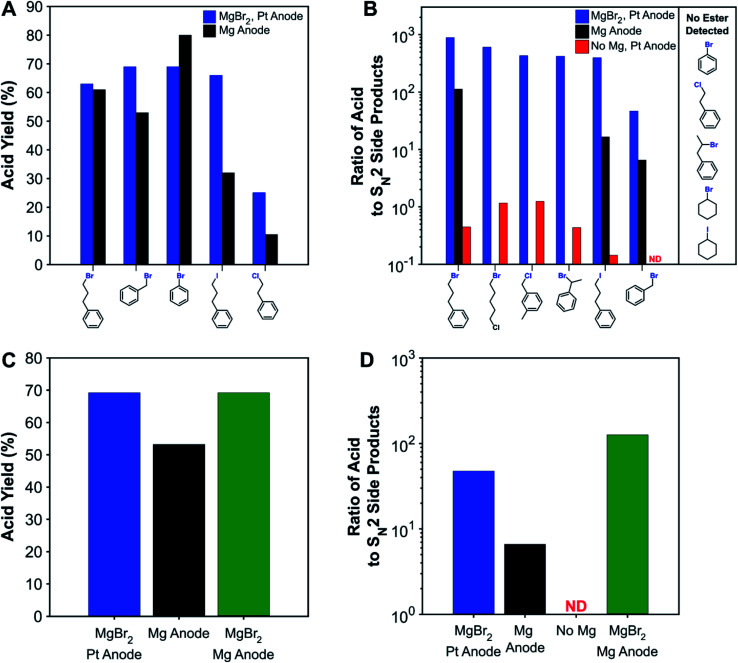
(A) Comparison of acid yields for non-sacrificial-anode and sacrificial-anode carboxylation of various substrates. (B) Ratio of carboxylic acid to nucleophilic side products (ester + carbonate + alcohol) for various systems and substrates. Effect of adding MgBr_2_ to the sacrificial-anode system on the (C) acid yield and (D) ratio of acid to S_N_2 side products for benzyl bromide. Acid yields are tabulated in Table S6.† ND: acid not detected (acid-to-S_N_2 ratio <0.1).

To compare with traditional processes which use sacrificial anodes, several substrates were carboxylated with a sacrificial magnesium anode. Fig. 2A shows that the sacrificial-anode-free process was able to perform similarly or better than the sacrificial-anode process for all substrates except bromobenzene. For benzyl bromide and the primary alkyl iodide, higher acid yields were obtained in the sacrificial-anode-free process due to better protection against esterification, since these substrates are the most susceptible to nucleophilic attacks. When MgBr_2_ was added to the electrolyte for the sacrificial-anode process, an identical yield was obtained for benzyl-bromide carboxylation as was obtained from the sacrificial-anode-free process. Although its yield is consistently low across all reaction conditions, the primary alkyl chloride had a higher carboxylation yield with the sacrificial-anode-free process. For all sacrificial-anode syntheses, the reaction was terminated early due to the cell voltage exceeding the potentiostat's limits from MgCO_3_ precipitation at the cathode (Fig. S24†). In most cases, a high conversion of substrate could be achieved before this event, but in the case of the primary alkyl chloride, the substrate conversion was not high enough due to the greater difficulty with reducing this substrate. The carboxylation of the primary alkyl chloride illustrates another drawback of metallic sacrificial anodes: the reaction can be stopped prematurely if too much CO_3_^2−^ forms and precipitates with the metal cations produced from the anode.

While the protecting effect of Mg^2+^ cations was demonstrated for the carboxylation of a primary alkyl bromide, a broader understanding of the protection needs for a wide variety of substrates would provide better design guidance for sacrificial-anode-free reaction conditions. Carboxylation of several representative substrates in a sacrificial-anode-free setup but without MgBr_2_ were performed (Fig. 2B). As expected, carboxylation of aromatic halides does not require a protecting cation, since aromatic carbons are not susceptible to S_N_2 reactions. In fact, nearly identical carboxylation yields were obtained for bromobenzene with or without MgBr_2_ and a non-sacrificial anode. Additionally, the secondary alkyl bromides, secondary alkyl iodide, and primary alkyl chloride showed very little, if any, esterification.

Since some substrates were susceptible enough to also react with CO_3_^2−^, the ratio of acid to the sum of S_N_2-derived products (ester + carbonate + alcohol) was used as a metric to quantify nucleophilic susceptibility; this metric is denoted as the acid-to-S_N_2 ratio. The ranked order of nucleophilic susceptibility based on this ratio is as follows: 1° benzylic Cl < 1° alkyl Br < 2° benzylic Br < 1° alkyl I < 1° benzylic Br. This trend compares well with general S_N_2 reactivity trends of alkyl halides in aprotic solvents and computationally estimated reaction barriers (Fig. S17†).^77,78^ The acid-to-S_N_2 ratios here are not perfectly intrinsic values since in some of the experiments, especially when using benzylic halides, there was also substrate and product oxidation. Moreover, the acid-to-S_N_2-ratio can also be sensitive to how long the reaction mixture remains in the cell before workup since S_N_2 reactions can happen in the absence of current. While not entirely comprehensive, these selected substrates should be diverse enough to enable some prediction of the necessity of a protecting cation to compounds containing other types of carbon–halide bonds.

Adding a protective cation increases the acid-to-S_N_2 ratio by several orders of magnitude (Fig. 2B). For the substrates susceptible to S_N_2 reactions, the increase is typically several orders of magnitude in the presence of MgBr_2_. Notably, for those substrates where a sacrificial anode experiment was performed, the acid-to-S_N_2-ratio is also roughly an order of magnitude higher with MgBr_2_, although the effect of this increase on acid yields is not always large. For the experiment where both MgBr_2_ and a sacrificial Mg anode were used, an even higher acid-to-S_N_2 ratio was achieved, showing the benefits that adding inorganic salts such as MgBr_2_ can have on sacrificial-anode processes as well.

**Scheme 3:**
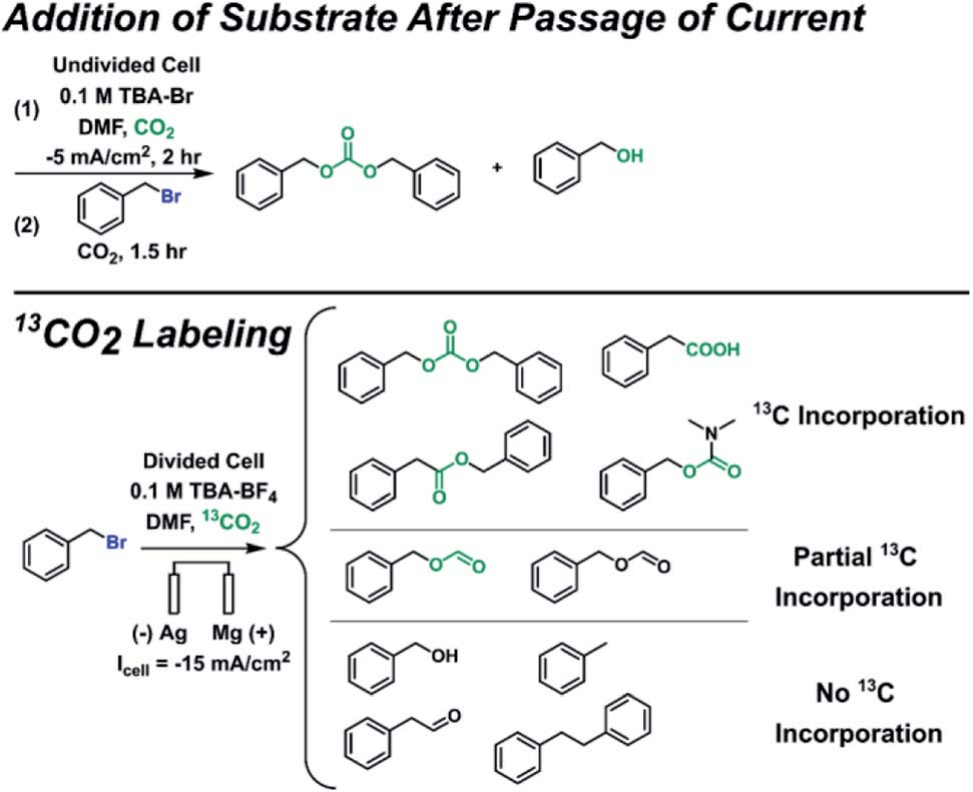
Mechanistic and control experiments.

Since additional side products formed for some substrates in addition to the ester and alkane, several control experiments were performed to identify the full range of products (Scheme 3 and ESI†). Benzyl bromide was used as the substrate here since it is the most susceptible to side reactions of all the substrates investigated. Control experiments where the substrate was added after current was passed under CO_2_ bubbling revealed that carbonate and alcohol products were formed, confirming that carbonate produced during CO_2_ reduction at the cathode can act as a nucleophile. The alcohol is not formed from trace water as confirmed by other control experiments; it can arise from an organic carbonate intermediate R–OCOO^−^ that loses CO_2_, resulting in an oxide R–O^−^ that can form an alcohol during workup.^63^ To minimize the effects of interference from anodic oxidation of the substrate and products, carboxylation was performed in a divided cell but with a Mg anode to allow for some nucleophilic reactions to happen in the catholyte. A ^13^CO_2_ labeling experiment under these conditions confirmed the carboxylic acid, ester, and carbonate all derive from CO_2_. Several additional side products were detected that appeared to originate from reactions with the solvent, including benzyl formate, phenylacetaldehyde, and *N*,*N*-dimethylbenzylcarbamate. While it is beyond the scope of this work to thoroughly study the formation mechanisms of these additional side products, possible formation mechanisms can be proposed. Phenylacetaldehyde could arise from a pathway similar to the Bouveault reaction, whereby an aldehyde is formed from the reaction of a Grignard reagent and an *N*,*N*-disubstituted formamide.^79^ The ^12^C benzyl formate could originate from a Vilsmeier intermediate that becomes hydrolyzed during workup,^80^ while the ^13^C benzyl formate could arise from a nucleophilic attack by a formate anion, itself a product of CO_2_ reduction at the cathode. The ^13^C *N*,*N*-dimethylbenzylcarbamate could result from a nucleophilic attack by a carbamate anion, itself formed by CO_2_ reacting with dimethylamine; the dimethylamine could be present from DMF decomposition. All in all, these mechanistic and control experiments reveal the breadth of chemistry that can occur, especially in the absence of a protecting cation such as Mg^2+^.

### Cathodic protection by the carboxylate product

**Fig. 3:**
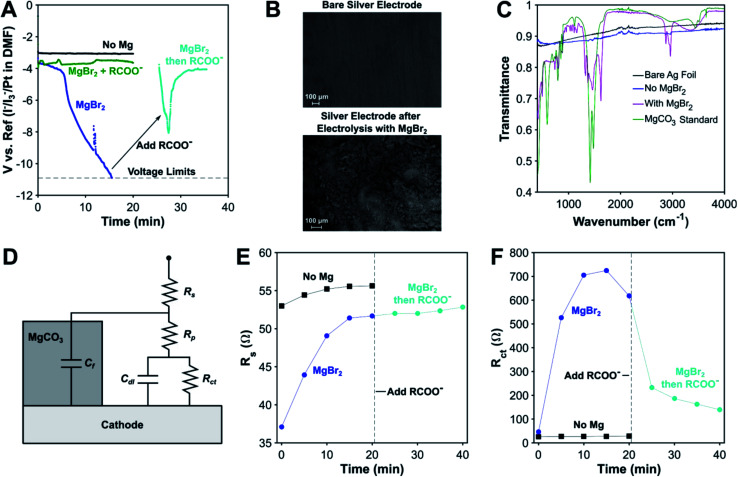
Investigation of MgCO_3_ passivation of the cathode during electrocarboxylation. (A) Effect of Mg^2+^ and RCOO^−^ on the cathodic potential over time. Conditions: undivided cell, 20 sccm CO_2_, 0.1 M TBA-Br, −5 mA cm^−2^, DMF. Reference electrode: 10 mM I^−^/I_3_^−^/Pt in DMF. R = 3-phenylpropyl. Solution resistance was not compensated. (B) SEM images of the Ag cathode after electrolysis without (top) and with (bottom) Mg^2+^ present. (C) FTIR of the Ag surface after electrolysis with and without MgBr_2_. (D) Equivalent circuit model for EIS analysis of MgCO_3_ passivation. Effect of Mg^2+^ and RCOO^−^ on (E) solution resistance (*R*_s_) and (F) charge transfer resistance (*R*_ct_) over time.

As mentioned above, one of the key disadvantages of using sacrificial metal anodes for carboxylation is the precipitation of inorganic carbonates, which can passivate the cathode. We have discovered that the carboxylate anion plays a decisive role in protecting the cathode from passivation by inorganic carbonates through electrolysis conducted without a carboxylation substrate. While the cathodic voltage is stable in the absence of MgBr_2_, it becomes highly unstable when MgBr_2_ is added (Fig. 3A). Scanning electron microscopy (SEM) images and Fourier Transform Infrared (FTIR) spectra of the electrode surface post reaction as well as previous studies^29^ confirm that this decrease in cathodic voltage is attributable to MgCO_3_ precipitation onto the cathode (Fig. 3B and C). Surprisingly, once an organic carboxylate is added, the cathodic voltage regains stability, even after being passivated by MgCO_3_, illustrating the profound effect the carboxylate anion has in ensuring the stability of the electrochemical system. A limit does exist to the carboxylate's protecting ability, as most experiments involving a magnesium anode finished prematurely as a result of the potentiostat's voltage limits being exceeded (Fig. S24†). A combination of the protecting ability of the organic carboxylate and the fixed amount of Mg^2+^ cations maintains the operational stability of the non-sacrificial-anode process.

To gain some insight into the origins of this phenomenon, galvanostatic electrochemical impedance spectroscopy (GEIS) was performed at the operating current density of −5 mA cm^−2^. An equivalent circuit consisting of the standard Randle's circuit with additional circuit elements to account for blockage by MgCO_3_ was used, as has been previously used to model EIS spectra of CaCO_3_ scaling.^81^ For the experiment without MgBr_2_, only the Randles circuit (*C*_f_ = 0 in Fig. 3D) was needed to obtain a good fit, and the circuit parameters varied slightly over the course of 20 min. In the presence of MgBr_2_, the charge transfer resistance increased greatly over 20 min coupled with a smaller increase in solution resistance. A second smaller loop developed as well, which is indicative of a second RC time constant (Fig. S20†). This extra feature supports the use of a more complex surface passivation circuit model. Once the carboxylate is added, the charge-transfer resistance decreases significantly while the solution resistance stays almost constant, increasing slightly. The second time constant also disappears in the GEIS spectra. A control experiment where TBA 4-phenylbutyrate was added to DMF and MgCO_3_ revealed that the carboxylate does not make the MgCO_3_ soluble. Based on these data, the role of the carboxylate appears to be removing MgCO_3_ off the surface of the cathode. Given the evidence for Mg–carboxylate interactions discussed earlier, a plausible mechanism is the carboxylate coordinating to the outer surface of the MgCO_3_ layer. The organic side chains of the carboxylate can form a channel that enables reactants and products to diffuse to and away from the cathode. For some deeply cathodic reactions such as electrochemical Birch reductions,^82^ specific protecting agents are needed to protect against cathodic passivation, but here, we have discovered that electrocarboxylation has an inherent protecting mechanism *via* the carboxylate product.

**Scheme 4:**
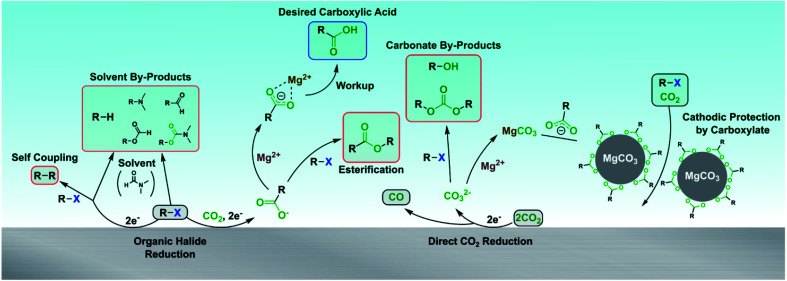
Summary of possible reaction pathways during the electrocarboxylation of organic halides.

A full picture of the complex reaction chemistry occurring at the cathode and in the catholyte is depicted in Scheme 4. The desired carboxylation pathway requires the substrate to be reduced at the cathode and react with CO_2_, avoiding reacting with another substrate molecule or the solvent. Depending on the substrate, Mg^2+^ cations are then required to stabilize the carboxylate and prevent S_N_2 reactions until the reaction is over and workup can be performed. The carboxylate also protects the cathode from passivation due to MgCO_3_ precipitation from concomitant CO_2_ reduction to CO. This comprehensive picture of the reaction chemistry provides many new insights that will accelerate further developments to electrocarboxylation systems.

### Broader perspective

As a final note, we would like to provide perspective on the sustainability and practicality of the sacrificial-anode-free process developed in this work. While the use of a sacrificial metallic anode was eliminated, the source of the anhydrous MgBr_2_ salt can pose some sustainability problems. Historically, anhydrous MgBr_2_ has been made by reacting metallic Mg with liquid bromine or another suitable bromine source.^83^ This process essentially consumes metallic Mg as a sacrificial anode would, but the ability to only add as much Mg^2+^ as needed (typically a stoichiometric amount) reduces the overall consumption of metallic Mg and reduces the risk of MgCO_3_ precipitation passivating the cathode. Better sustainability can be achieved by recycling hydrated MgBr_2_ back into its anhydrous form, although direct drying under heat causes reversion into MgO or Mg(OH)_2_.^84^ Hydrated MgBr_2_ must either be dried under anhydrous HBr^85^ or precipitated and dried from a mixture of alcohol and ether.^84^ Although it may be somewhat challenging to recycle, anhydrous MgBr_2_ does improve the sustainability of electrocarboxylation by eliminating the need for consuming metallic magnesium.

In terms of practicality, there likely exists a certain production scale and product value for which a sacrificial-anode-free carboxylation process would make more economical sense than one with a sacrificial anode. For high-value, complex substrates that can be carboxylated in batch systems, sacrificial-anode-based processes are likely more desirable since the system setup is simpler and oxidation of the high-value substrate is avoided. For processes at a scale where continuous manufacturing would enable significant cost reduction, a sacrificial-anode-free process would likely fare better. Therefore, exploring and improving both sacrificial-anode and sacrificial-anode-free carboxylation processes is beneficial to maximizing the potential of electrocarboxylation as a synthetic pathway to carboxylic acids.

## Conclusions

This work demonstrates a design strategy to perform electrochemical carboxylation without a sacrificial anode while maintaining carboxylic acid selectivity. Sacrificial anodes protect the cathodically generated carboxylate by producing cations that coordinate the carboxylate and prevent unwanted S_N_2 reactions that produce esters, carbonates, and alcohols. Soluble inorganic salts can mimic this protecting property, allowing other anodic reactions to be used. After screening reaction conditions, a wide variety of aliphatic, benzylic, and aromatic halides were carboxylated with decent to good yields (34–78%) without a sacrificial anode. Generally comparable or higher yields were obtained with the sacrificial-anode-free methodology relative to a traditional sacrificial-anode methodology. The need for a protecting cation such as Mg^2+^ was evaluated for numerous types of organic halides and found to correlate with known nucleophilic susceptibilities of the carbon–halide bond type, providing an understanding of which substrates will need protecting cations for selective carboxylation. This work also revealed the protecting effect of the organic carboxylate product against cathodic passivation by insoluble carbonates. Taken together, these results show that electrocarboxylation with CO_2_ can be done without a sacrificial anode while preserving selectivity, providing a step towards realizing the sustainability potential of this process.

## Data availability

All of the data to support this article are in the main text and the ESI.†

## Author contributions

N. C., D.-T. Y., and K. M. conceptualized the work. N. C. and D.-T. Y. developed methodology and designed experiments. N. C. carried out the experimental work and performed data analysis. N. L. acquired SEM images. N. C. wrote the original draft and created the original visuals. N. C., N. L., K. S., and K. M. edited the draft and visuals.

## Conflicts of interest

There are no conflicts to declare.

## Supplementary Material

SC-012-D1SC02413B-s001
