# Correction: Suppressing carboxylate nucleophilicity with inorganic salts enables selective electrocarboxylation without sacrificial anodes

**DOI:** 10.1039/d1sc90196f

**Published:** 2021-09-23

**Authors:** Nathan Corbin, Deng-Tao Yang, Nikifar Lazouski, Katherine Steinberg, Karthish Manthiram

**Affiliations:** Department of Chemical Engineering, Massachusetts Institute of Technology 77 Massachusetts Avenue Cambridge Massachusetts 02139 USA karthish@mit.edu

## Abstract

Correction for ‘Suppressing carboxylate nucleophilicity with inorganic salts enables selective electrocarboxylation without sacrificial anodes’ by Nathan Corbin *et al.*, *Chem. Sci.*, 2021, DOI: 10.1039/D1SC02413B.

We regret that there was a minor error in the structure of the benzyl chloride in Scheme 2, Fig. 2 and the ESI. The structure of the benzyl chloride should be 4-methyl benzyl chloride but was instead given as 3-methyl benzyl. The correct figure and scheme are shown below, and the ESI has been updated.

**Scheme 2:**
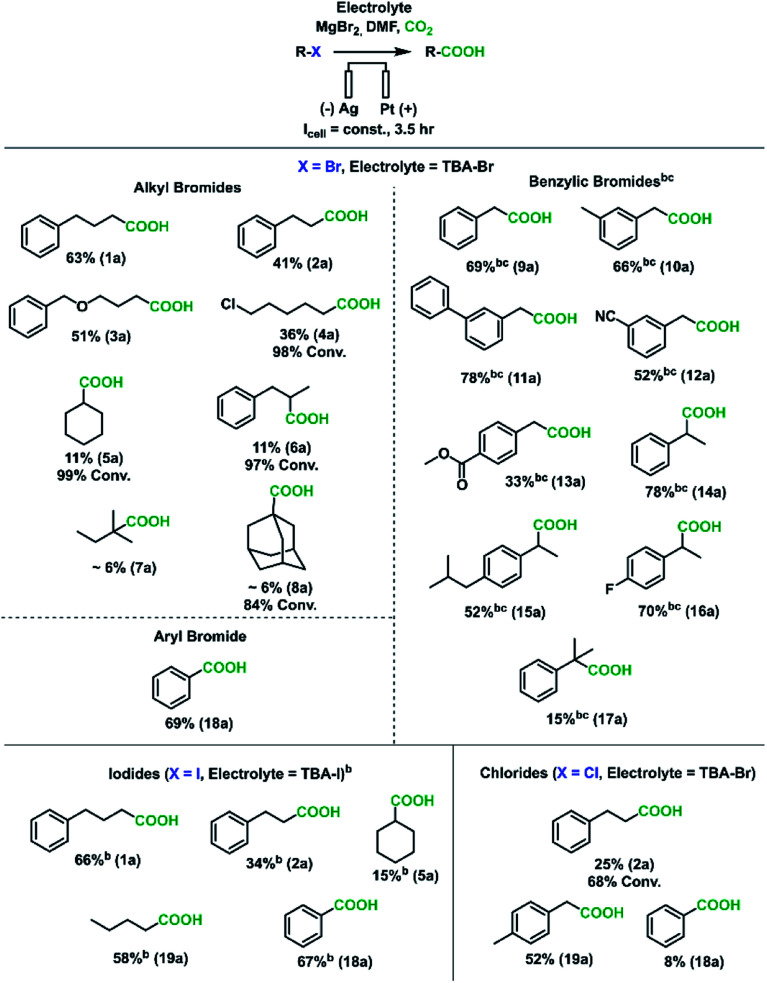
Substrate scope for the sacrificial-anode-free electrochemical carboxylation of organic halides. ^a^Standard reaction conditions: 100 mM electrolyte, 100 mM substrate, 100 mM MgBr_2_, silver cathode, platinum anode, 20 sccm CO_2_, 2.2 mL DMF, −20 mA cm^−2^ for 3.5 h. TBA-Br was used for chlorinated substrates because bromide oxidizes more readily than chloride, and only a small amount of chloride was replaced by bromide (<1% for the alkyl chloride, ∼4% for the benzylic chloride). Yields are referenced to the initial amount of substrate and were calculated from ^1^H NMR spectroscopy using either 1,3,5-trimethoxybenzene or ethylene carbonate as internal standards. ^b^−15 mA cm^−2^ instead of −20 mA cm^−2^. ^c^150 mM MgBr_2_ instead of 100 mM MgBr_2_.

**Fig. 2:**
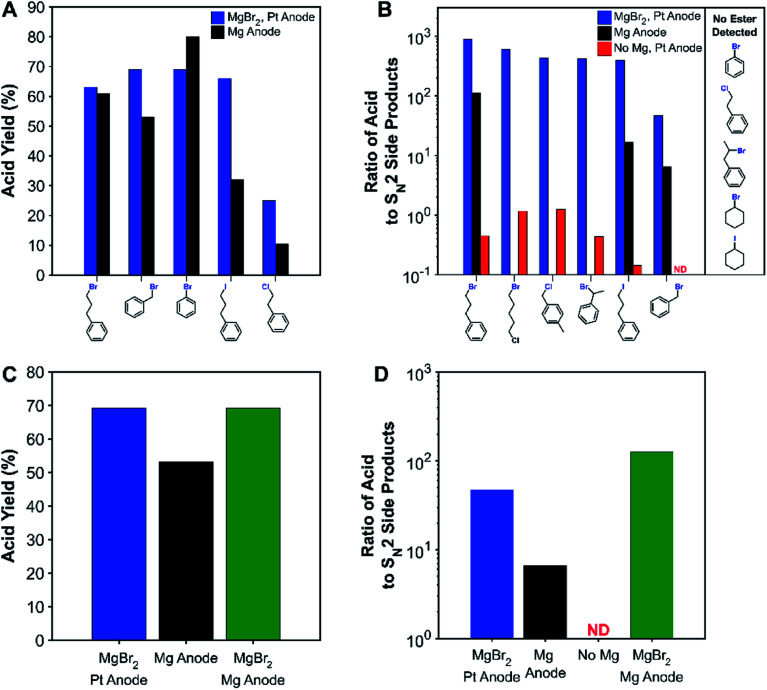
(A) Comparison of acid yields for non-sacrificial-anode and sacrificial-anode carboxylation of various substrates. (B) Ratio of carboxylic acid to nucleophilic side products (ester + carbonate + alcohol) for various systems and substrates. Effect of adding MgBr_2_ to the sacrificial-anode system on the (C) acid yield and (D) ratio of acid to S_N_2 side products for benzyl bromide. Acid yields are tabulated in Table S6.† ND: acid not detected (acid-to-S_N_2 ratio <0.1).

The Royal Society of Chemistry apologises for these errors and any consequent inconvenience to authors and readers.

## Supplementary Material

