# Supplementary material for: Suppressing carboxylate nucleophilicity with inorganic salts enables selective electrocarboxylation without sacrificial anodes
Source: Chem Sci. 2021 Aug 16;12(37):12365–76. doi: 10.1039/d1sc02413b (PMC8480422; doi:10.1039/d1sc02413b)
Supplement: SC-012-D1SC02413B-s001 [file SC-012-D1SC02413B-s001.pdf]

Electronic Supplementary Material (ESI) for Chemical Science.
This journal is © The Royal Society of Chemistry 2021
ESI updated 24/09/2021

Supporting Information

Suppressing Carboxylate Nucleophilicity with Inorganic Salts Enables Selective Electrocarboxylation without Sacrificial Anodes

Nathan Corbin, Deng-Tao Yang, Nikifar Lazouski, Katherine Steinberg, and Karthish Manthiram*

Department of Chemical Engineering, Massachusetts Institute of Technology, 77 Massachusetts Avenue, Cambridge, Massachusetts 02139, United States

*Correspondence: karthish@mit.edu

Contents

1 Materials 3
1.1 Materials Handling and Preparation 6
2 Electrolyte Preparation 6
3 Cell Preparation and Cleaning 7
3.1 Silver Cathodes 7
3.2 Pt Anodes 7
3.3 Mg Anodes 7
3.4 Cell Components 7
3.5 Cell Assembly 8
4 Electrochemical Experiments 9
5 Product Quantification 10
5.1 General Workup Procedure for Two-Compartment, Low-Conversion Experiments with 1-Bromo-3-Phenylpropane (Procedure A) 10
5.2 General Workup Procedure for Obtaining Isolated Yields of Acids in Presence of $MgBr_2$ or with a Sacrificial Anode (Procedure B) 11
5.2.1 Quantification of Ester Yields 11
5.3 General Workup Procedure for Experiments without any Magnesium Source (Procedure C) 12
5.3.1 Workup Procedure for EtOAc (Procedure C1) 12
5.3.2 Workup Procedure for Et_2O (Procedure C2) 12
5.4 NMR Analysis 13
5.5 In-Line Gas Chromatography for CO and H_2 Quantification 13
5.6 Gas Chromatography-Mass Spectrometry (GC-MS) 16

6 Syntheses of Carboxylate and TBA Salts 18
6.1 Tetra-n-butylammonium 4-Phenylbutyrate 18
6.2 Magnesium 4-Phenylbutyrate 18
7 Additional Figures 19
7.1 Electrochemical Impedance Spectroscopy 28
7.2 Fourier Transform Infrared Spectroscopy (FTIR) 32
8 Scanning Electron Microscopy (SEM) 33
9 Carboxylation Mechanistic & Control Experiments 33
9.1 Divided Cell with a Magnesium Anode 33
9.2 $^{13}CO_2$ Experiment, Divided Cell, Mg Anode 37
9.3 Deuterated Solvent Experiment 47
9.4 Control Experiments without Current 50
9.5 Control Experiments Adding Substrate After Passing Current 51
9.6 Carbon monoxide as an Intermediate in Carboxylation 51
9.7 Lack of Interaction Between $MgBr_2$ and R-Br 52
10 Grignard Carboxylation Reactions 53
11 Experimental Details and Characterization Data of Products 55
11.1 Notes about Impurities and Internal Standards in NMR Spectra 55
11.2 Carboxylation Using $MgBr_2$ and a Non-Sacrificial Anode 56
11.3 Carboxylation with Sacrificial Anodes 70
11.4 Carboxylation without Magnesium 73
11.5 Reactions Which Had Cell Leakage 81
12 Voltage-Time Traces for Non-Sacrificial and Sacrificial-Anode Syntheses 85
13 NMR and Mass Spectra 86
13.1 Reactions with $MgBr_2$ and a Non-Sacrificial Anode 86
13.2 Spectra from Carboxylations with Sacrificial Anodes 125
13.3 Spectra from Reactions without Magnesium 130
13.4 Reactions Which Had Cell Leakage 148
14 References 152

1 Materials

Name	Specs	Vendor	Lot #
Acetone	ACS	Fisher	194715<br>202370
	ACS	VWR BDH	0000252292<br>0000280733
Deuterated dimethyl sulfoxide (DMSO-d_6)	99.9%	Sigma-Aldrich	MKCL4439<br>MKCP3259
Diethyl ether (Et_2O)	ACS	J.T. Baker	0000188135<br>0000241406
Tetrahydrofuran (THF)	Anhydrous, ≥ 99.9%<br>Inhibitor free	Sigma-Aldrich	SHBJ0753
Ethyl acetate (EtOAc)	ACS, 99.9%	Fisher	182005
Hexane	ACS	VWR	0000247836
N,N-Dimethylformamide (DMF)	99.8% anhydrous	Sigma-Aldrich	SHBK9314<br>SHBM0052<br>SHBM5870
Acetonitrile (MeCN)	99.8+% anhydrous	Alfa Aesar	Q026708
Dimethyl sulfoxide (DMSO)	99.90%	Fisher	181773
Propylene carbonate	99%	Alfa Aesar	T07B027
Methanol (MeOH)	99.9% anhydrous	Alfa Aesar	U05F795<br>N26G717
Deuterated chloroform ($CDCl_3$)	99.8 (0.03% TMS)	Sigma-Aldrich	MKCL1035<br>MKCJ1046
Water (MilliQ)	18.2 Ohm	EMD Millipore	
Tetra-n-butylammonium hydroxide (TBA-OH) in methanol	1 M	Beantown Chemical	50018025
Carbon dioxide (CO_2)	99.999%	Airgas	
Carbon monoxide (CO)	99.99%	Airgas	
CO, H_2 Calibration Gas	1% CO, 1% H_2, 1% CH_4 in CO_2	Airgas	160-400915689-1
4-Phenylbutyric acid	99%	Beantown Chemical	A50011203
n-Propylbenzene	Analytical standard	Sigma-Aldrich	LRAB8369
Maleic acid	≥ 99% (HPLC)	Sigma-Aldrich	SLBZ1196
Ethylene carbonate	98%	Sigma-Aldrich	MKBQ3570V
Nitric acid (HNO_3)	Trace metal grade, 67-70 wt.%	Fisher	1119080
Hydrobromic acid (HBr)	ACS, 47-49 wt.%	Beantown Chemical	50010396
Hydrochloric acid (HCl)	ACS, 37 wt.%	Sigma-Aldrich	MKCJ5987<br>2020090321
Dibromomethane	99%	Sigma-Aldrich	WXBC7356V
Nitrogen (N_2)		Airgas, liquid N_2 boil off	
Argon	UHP 5.0 grade	Airgas	
1,3,5-Trimethoxybenzene (TMB)	≥ 99%	Sigma-Aldrich	STBH6762

			STBJ8805
Triethylamine (Et_3N)	≥ 99%	Sigma-Aldrich	SHBH7216
N,N-Dicyclohexylmethylamine (Cy_2MeN)	97%	Sigma-Aldrich	SHBJ7958
Iodine	ACS, ≥ 99.8%	Sigma-Aldrich	SHBJ4561
Magnesium bromide ($MgBr_2$)	98%, anhydrous	Acros Organics	A0402216
Magnesium sulfate ($MgSO_4$)	Reagent grade	VWR	19G1056703
Magnesium oxide	99.995% trace metals basis	Beantown Chemical	50024742
Magnesium carbonate ($MgCO_3$)	Pharmaceutical reference standard	Sigma-Aldrich	LRAC4013
Lithium bromide (LiBr)	Anhydrous, reagent plus, ≥ 99%	Sigma-Aldrich	MKCG6451
Sodium bromide (NaBr)	Anhydrous, reagent plus, ≥ 99%	Sigma-Aldrich	MKCF6811
Potassium bromide (KBr)	FTIR grade, ≥ 99% trace metals basis	Sigma-Aldrich	BCBV957
Cesium bromide (CsBr)	Anhydrous beads, 10 mesh, 99.999% trace metals basis	Sigma-Aldrich	0000046836
Aluminum bromide ($AlBr_3$)	Anhydrous 98+%	Acros Organics	A0410603
Potassium hydroxide (KOH)	ACS	Mallinckrodt Chemical	J10K51
Sodium thiosulfate pentahydrate	ACS, ≥ 99.5%	Sigma-Aldrich	MKBT8948V
Sodium bicarbonate ($NaHCO_3$)	ACS, ≥ 99.7%	Sigma-Aldrich	SLBZ5815
Sodium carbonate (Na_2CO_3)	BioXtra ≥ 99.0%	Sigma-Aldrich	SLBT0414
Sodium hydroxide (NaOH)	ACS	Macron	0000149802
Sodium sulfate (Na_2SO_4)	ACS	VWR BDH	2986C508
Sodium chloride (NaCl)	ACS, ≥ 99.0%	Sigma-Aldrich	SLBZ3816
Potassium phosphate monobasic (KH_2PO_4)	≥ 99%, anhydrous	Sigma-Aldrich	SLBV4931
Sodium borohydride ($NaBH_4$)	99.99% trace metals	Sigma-Aldrich	MKBZ2376V
Magnesium turnings	Sure-Seal bottle	Sigma-Aldrich	MKCF5135
Tetra-n-butylammonium tetrafluoroborate (TBA-BF_4)	> 98%	TCI	60ZJF
Tetra-n-butylammonium bromide (TBA-Br)	≥ 99.0%	Sigma-Aldrich	BCCB5962
Tetra-n-butylammonium iodide (TBA-I)	98%	Sigma-Aldrich	BCBV8505
1-Bromo-3-phenylpropane	98%	Sigma-Aldrich	BCBV8040
1-Bromohexane	98%	Sigma-Aldrich	WXBC4599V
Bromocyclohexane	98%	Sigma-Aldrich	BCBV8389
1-Bromoadamantane	99%	Sigma-Aldrich	MKCH3609
2-Bromo-2-methylbutane	95%	Sigma-Aldrich	MKCF3955
Benzyl 3-bromopropyl ether	98%	Combi-Blocks	B37471
1-Bromo-4-fluorobutane	97%	Alfa Aesar	81302237
2-Bromo-1-phenylpropane	96%	Combi-Blocks	A36901
1-Bromo-5-chloropentane	98%	Combi-Blocks	B70314
(2-Bromoethyl)benzene	98%	Alfa Aesar	T28C015

Bromobenzene	≥ 99.5%	Sigma-Aldrich	BCBT5468
Chlorobenzene	99.8%	Sigma-Aldrich	SHBK5435
(2-Choroethyl)benzene	99%	Sigma-Aldrich	MKCF2472
3-Bromocyclohexene	95%, propylene oxide stabilized	Alfa Aesar	10164206
1-(1-Bromoethyl)-4-fluorobenzene	95%	Asta Tech	P102-15026
3-Phenyl benzyl bromide	97%	Sigma-Aldrich	BCBM6382V
4-Methyl benzyl chloride	98%	Alfa Aesar	Z19F052
3-Methyl benzyl bromide	96%	Frontier Scientific	L580O87
3-(Bromomethyl)benzonitrile	> 98.0%	TCI	UVIBM-BJ
4-Trifluoromethyl benzyl bromide	98%	Alfa Aesar	B19S033
(1-Bromoethyl)benzene	97%	Alfa Aesar	10201471
Benzyl bromide	98%	Sigma-Aldrich	MKCK2732
1-Iodo-3-phenylpropane	97%	Sigma-Aldrich	MKCK9205
(2-Iodoethyl)benzene	97%	Sigma-Aldrich	MKCF5417
Iodobenzene	98%	Sigma-Aldrich	BCCB1269
Iodocyclohexane	> 98.0%, stabilized with copper chip	TCI	AEDZB-GF
1-Iodobutane	99%, stabilized with copper	Alfa Aesar	10222687
1-Bromobutane	99%	Sigma-Aldrich	MMKCC0531
Bromocyclopentane	≥ 98%	Sigma-Aldrich	STBH4882
Methyl 4-(bromomethyl)benzoate	98%	Alfa Aesar	E2992A
4-Isobutylacetophenone	98%	J & K	LE60O94
2-Phenyl-2-propanol	97%	Sigma-Aldrich	STBH8338
Dibromoethane	≥ 99%	Sigma-Aldrich	BCBV6295
Bromotrimethylsilane (TMSBr)	98%	Acros Organics	A0376248
Phosphorus tribromide (PBr_3)	99%	Acros Organics	A0386840
Platinum foils	99.99% trace metals basis, 0.025 mm thick	Beantown Chemical	50040063
Magnesium foils	Magnesium alloy	More Metals (Amazon)	ASIN: B07JBZ31HD
Silver foils	99.998% metals basis, 0.1 mm thick, hard, Premion	Alfa Aesar	X22E033
Copper foils	99.999% trace metals basis, 2.0 mm thick, Puratronic	Alfa Aesar	
Gold foils	99.99% trace metals basis, 0.127 mm thick, Premion	Alfa Aesar	
Daramic 175 polyporous separator		Daramic (Charlotte, NC)	
Celgard 3501 separator		Celgard	
3 Å Molecular sieves	4-8 mesh	Acros Organics	A0399419

1.1 Materials Handling and Preparation

Tetra-n-butylammonium salts were dried in a vacuum oven at 60 – 80 °C overnight (12+ hr) and stored in an argon-filled glovebox (Vacuum Atmospheres Genesis) with H_2O levels at or below 11 ppb. Magnesium bromide was taken directly into the glovebox before opening. Solvents were dried over 3 Å molecular sieves (~10 – 20 vol.%) overnight before use in amber-colored glass vials or jars; for non-anhydrous solvents (i.e. solvents not stored in bottles with septa), two rounds of sieve drying were used. Each round involved drying with sieves overnight (> 12 hr), refreshing the sieves between rounds. Moisture levels of solvents were checked with Karl-Fischer titration (Mettler Toledo C10s KF Titrator) and confirmed to be < 1 mM (10 μg detection limit, 500 – 750 μL solvent). Molecular sieves were regenerated by washing them with acetone and then drying at 350 °C for at least four hours. 1-Bromo-3-phenylpropane and tertiary amines were also stored over 3 Å sieves to minimize moisture sources in the electrolyte. Containers holding substrates and tertiary amines were flushed with N_2 for 10 - 15 sec after use. All chemicals that were dried with molecular sieves were stored in desiccators.

2 Electrolyte Preparation

All electrolyte salts were stored and weighed in an argon-purged glovebox with H_2O levels at or below 11 ppb. Magnesium bromide is a very hygroscopic salt, so as many precautions were taken as possible to minimize moisture exposure before introduction into the cell.

After the appropriate amounts of electrolyte salts were weighed out, they were added to 15 mL polypropylene centrifuge tubes (Corning, CentriStar caps) in the glovebox and transferred to a nitrogen-filled glove bag (Aldrich AtmosBag, size S, zipper lock) containing 3 Å molecular sieves as desiccant. Solvent was then added to the salts in the glove bag to minimize exposure to ambient moisture. Once all solids were dissolved, the electrolyte samples were removed from the glove bag. Two methods of substrate addition were used. One involved adding substrate outside the glovebag quickly to the electrolyte, weighing the centrifuge tube before and after to get an accurate amount of added substrate. This procedure was done for the high-yield syntheses, since accurate masses were important for calculating yields, and small amounts of water would not have too large of an impact. A second variation minimized water exposure. A pre-made solution of substrate in the desired solvent could be made and stored over a few (~ 5 vol. %) sieves overnight with a known weight percentage of substrate. After making the electrolyte in the glovebag, the amount of substrate could be deduced from its known weight fraction and the mass of added electrolyte to the centrifuge tubes. This procedure was found to give better results for the low-conversion experiments, since these are more sensitive to small amounts of water. The accuracy of the substrate mass may have been compromised a little but was still good enough to get good mass balance closures. For experiments involving tertiary amines, the tertiary amine was added directly to the cell once the rest of electrolyte was added to minimize moisture exposure.

3 Cell Preparation and Cleaning

3.1 Silver Cathodes

Silver foils (25 mm x 25 mm) were polished for ~ 1.5 min using 400 G silicon carbide sandpaper (Norton, Blue-Bak T414) on a piece of aluminum foil on a cut piece of a polystyrene weigh boat trimmed to make a shallow dish (VWR, 85 x 85 x 24 mm, anti-static). MilliQ water was added to fully submerge the silver foils on the aluminum. The polishing time was apportioned as follows: 1 min with small circular polishes using one half of the sandpaper and 30 sec of unidirectional polishing to produce a visibly uniform finish using the other fresh half of the sandpaper. A fresh piece of sandpaper was used for each new electrode, and a new piece of aluminum was used after 3 – 4 polishes. A second layer of nitrile gloves was worn only during polishing to minimize possible silver contamination. After polishing, the electrodes were rinsed with acetone (10 – 15 seconds per rinse, both sides of the foil). Then, a section of a Kimwipe was wetted with acetone, and the active side of the foil was wiped with the wetted Kimwipe to remove remaining solid particulates. The foils were then submerged in 1 M HNO_3 for ~ 1 min seconds followed by rinsing with MilliQ water (10 – 15 sec, both sides of the foil). The foils were sonicated (VWR Symphony, 90 W, 35 kHz) for at least 2 min in MilliQ water followed by rinsing with acetone (10 – 15 sec) and then blown dry with house nitrogen. The foils were stored in a covered, vented glass dish in a drying oven (Binder FD forced convection oven) at 80 °C until they were ready to be used (at least 15 min).

3.2 Pt Anodes

A piece of platinum foil was used as the anode. Pt foils were washed with acetone and wiped with an acetone-wetted Kimwipe after each experiment. This was often sufficient to restore the visual quality of the Pt, but about once a week, they were stored in 10 wt.% HNO_3 overnight. Before each experiment, the Pt foils were dried at 80 °C until they were ready for use (at least 15 min).

3.3 Mg Anodes

Fresh magnesium anodes were dried at 80 °C before use. Each side of the magnesium anode was used once; further uses would often result in leaking likely due to the O-rings not making a perfect fit against the roughened surface of a used magnesium surface.

3.4 Cell Components

After each experiment, the PEEK cells (including the FEP-encapsulated silicone O-rings) were washed with acetone. Often, some insoluble particulates remained in the cell, which could be removed by wetting a Kimwipe with acetone and scrubbing the particulates off. The cells were dried at 80 °C before being submerged into 20 wt.% HNO_3. The cells were either (1) sonicated for > 5 min in 20 wt.% nitric acid or (2) stored in 20 wt.% HNO_3 overnight. The cells were then removed from the nitric acid, washed with copious amounts of MilliQ, and sonicated for > 3 min in MilliQ. The cells were then washed with acetone and dried at 80 °C for > 30 min or, ideally, overnight.

TEFZEL (ETFE) plugs and PTFE hex-head screws were soaked in acetone overnight and dried at 80 °C for > 15 min before use.

Daramic and Celgard separators were washed with acetone until no discoloration of the acetone was observed and then dried at 80 °C for > 15 min.

After extended use with vacuum grease, the PEEK cells, PTFE hex-head screws, and TEFZEL nuts were soaked in hexane at 65 °C for several hours to the grease. They were washed with acetone and dried at 80 °C for several hours.

3.5 Cell Assembly

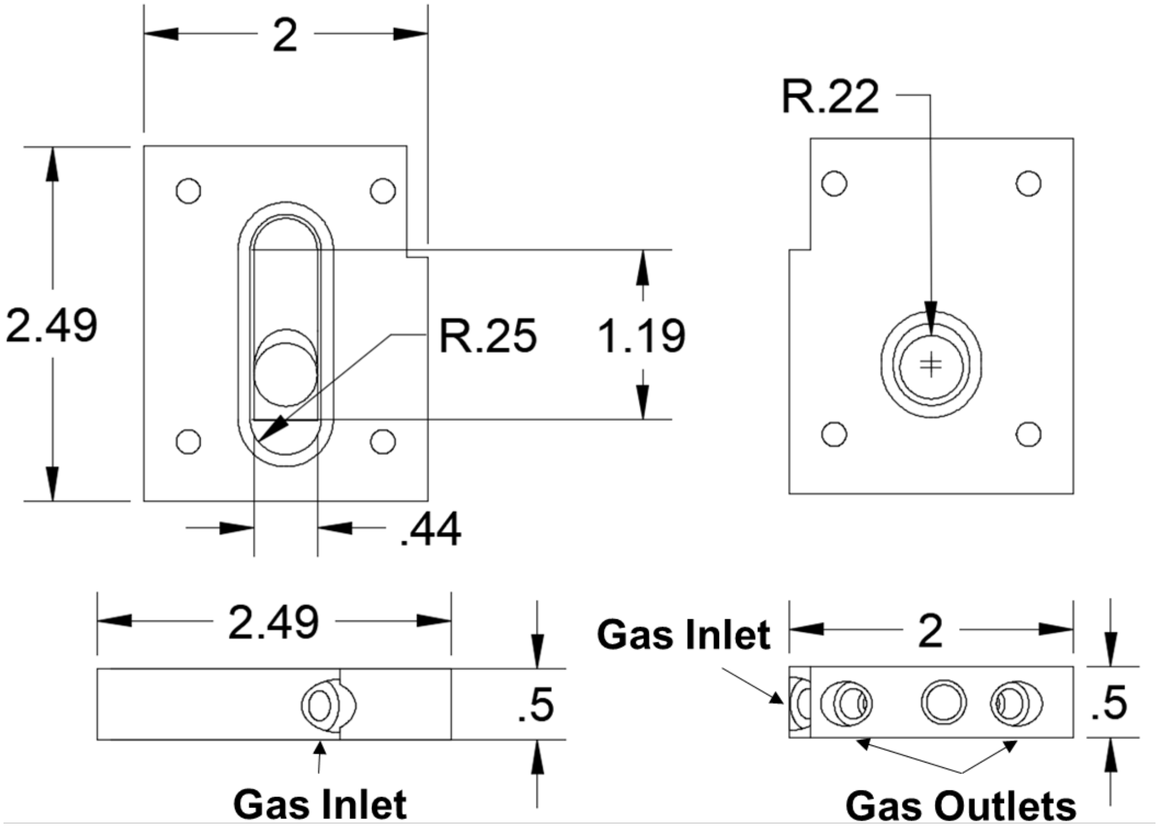

Figure S1. Schematics of the electrochemical cells used. Dimensions in inches are indicated along with where the gas inlet port is and the gas outlets. The gas inlet is positioned at an angle to bubble down into the electrolyte. Only one gas outlet port was used at a time, either connected to an in-line sampling GC, pressure equalizer, or to the atmosphere. The cathode-anode distance is 0.5 in.

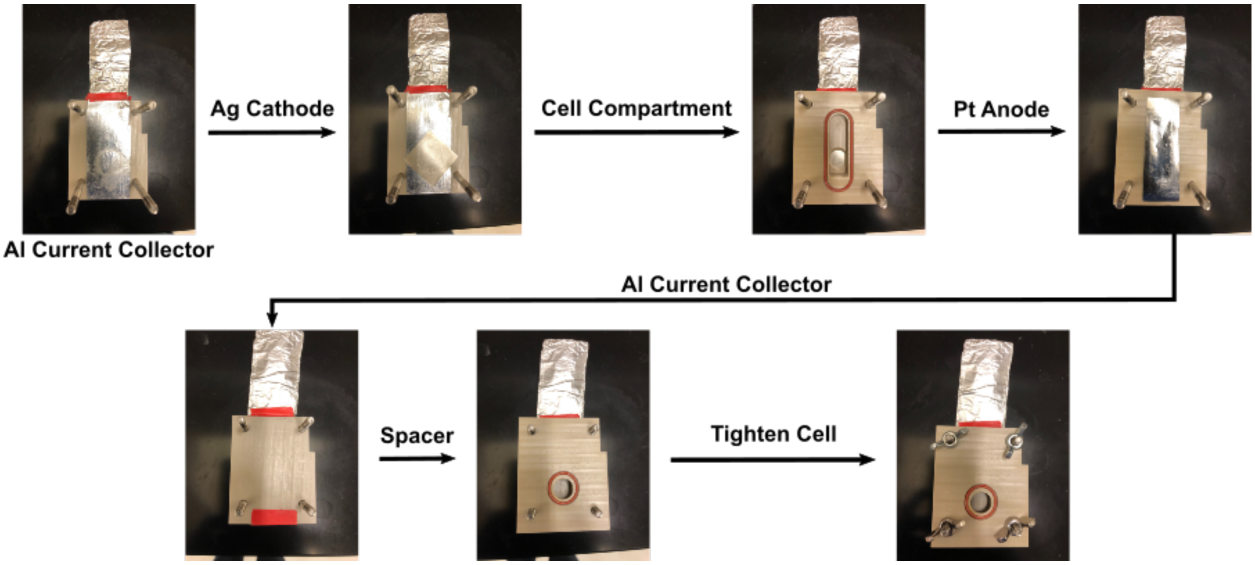

Figure S2. Assembly procedure for a one-compartment cell.

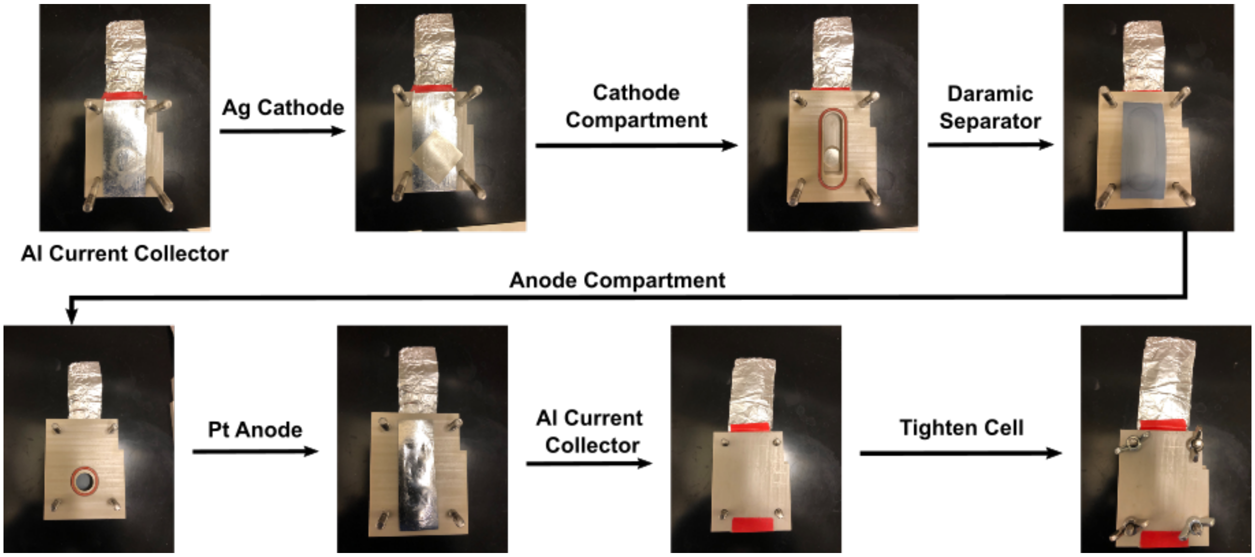

Figure S3. Assembly procedure for a two-compartment cell.

Electrochemical cells were assembled as shown in the above pictorial sequences. Cells were assembled with all components taken freshly out of the drying oven to minimize uptake of atmospheric water. Care was taken to ensure the O-rings made full contact with the electrodes (and separator if present). The screws were hand-tightened as much as possible to prevent leaks. To improve sealing, some vacuum grease was applied to the threads of the nuts used to connect the tubing to the cell inlets and outlets.

4 Electrochemical Experiments

After the cell was assembled, 20 sccm of dry CO_2 (Cole-Parmer Drierite 27068 L69GP gas purifier) was purged through the cathodic chamber (two-compartment cell) or entire cell (one-compartment cell) for > 2 min before electrolyte was added. The anodic compartment (two-

compartment cell) was either purged with CO_2 or N_2 (the flow rate for the anolyte was not always precisely controlled but was usually around 10 – 30 sccm). FEP tubing (Cole-Parmer) was used for gas flows; 1/16 in. OD was used for the gas inlet to the cell, and 1/8 in. OD otherwise. After addition of the electrolyte, the cell was purged for at least 10 min before electrolysis started. For experiments that measured CO and H_2, the outlet of the catholyte was sent to a gas sampling valve on an SRI 8610C gas chromatograph. The gas sampling occurred every 5 min. A BioLogic VMP3 potentiostat was used to control the current or voltage.

For experiments involving a reference electrode, a Pt wire (CH Instruments, CH112 with Pt wire) was immersed in a 10 mM solution of I_3^-/I^- (10 mM I_2, 20 mM TBAI) in DMF within a glass tube with a porous Teflon tip (CH Instruments). The reference electrode was inserted into a PTFE hex-head screw (3/8”-16 thread size, 1” long, McMaster Carr) with a hole drilled through the middle and carefully screwed into the cell. Vacuum grease could be applied to the threads to improve gas tightness. (Note: overtightening can place pressure on the glass reference electrode holder, causing it to break).

5 Product Quantification

5.1 General Workup Procedure for Two-Compartment, Low-Conversion Experiments with 1-Bromo-3-Phenylpropane (Procedure A)

The catholyte and anolyte were removed from the cell and combined in a 15 mL Corning polypropylene centrifuge tube. The cell was washed by adding 4 mL of a 1 M HBr solution to the cathode, transferred to the anode, and then combined with the anolyte and catholyte. The cell was then washed with 6 mL of Et_2O (4 mL to cathode, 2 mL to anode). 2 mL of the Et_2O from the cathode was added to the anode to equalize the washing volumes. Note that Et_2O does dissolve some of the polypropylene tube. This incompatibility is not a problem for quantification here, but it does introduce extra peaks on ^{1}H NMR spectra. Since bromine and tribromide can react with 1,3,5-trimethoxybenzene, some (typically 20 – 30 µL) 1 M sodium thiosulfate solution was added to the acid/Et_2O post-reaction mixture in an amount just enough to remove any discoloration (adding too much can result in a reaction with the brominated substrate, resulting in quantification errors). The Et_2O from the cell was combined with the reaction mixture, and the organic layer was extracted. Care should be taken during the first mixing step, as decent amounts of CO_2 gas can be liberated from the electrolyte upon addition of acid; the low surface tension of Et_2O can accelerate the rate of degassing. To help improve the quality of the phase separation, the extraction vials were centrifuged at 2,000 rpm for 10 seconds. Et_2O extraction was performed four more times on the aqueous fraction (two times with 4 mL, two times with 2 mL). After all organic fractions were combined, they were washed with 2 mL of 1 M HBr, and then this aqueous layer was extracted with Et_2O (2 mL) was to maximize product recovery. The combined organic fractions were dried with $MgSO_4$. 1,3,5-trimethoxybenzene (TMB) was weighed and added to the product solution as an internal standard. Product identification and quantification was performed respectively via gas chromatography-mass spectrometry (GC-MS) and GC-FID using TMB as an internal standard.

Product quantification via ^{1}H NMR could also be done by rotary evaporating (IKA RV 8, HB 10) the product solution (typically 2 – 3 min at room temperature to remove most of the diethyl

ether) and adding deuterated solvent; however, this procedure often led to some evaporation of the hydrogenated product, so GC-FID was typically the preferred quantification method. ^1H NMR was used to quantify the ester since the ester has a very high boiling point and is not commercially available as a pure substance to create a calibration curve for GC-FID.

5.2 General Workup Procedure for Obtaining Isolated Yields of Acids in Presence of $MgBr_2$ or with a Sacrificial Anode (Procedure B)

This procedure was followed for experiments requiring isolated yields. EtOAc was used instead of Et_2O for the initial extractions to ensure good extracting power for a wide range of carboxylic acids. The electrolyte was removed from the cell and added to its original 15 mL polypropylene tube. The cell was washed out once with 1 M HBr (4 mL) followed by EtOAc (4 mL) (Note: 1 M HCl could be substituted here as a cheaper alternative instead of 1 M HBr). When using an Mg anode, the acid washes of the cell were kept brief to minimize dissolution of the Mg. The washes were combined with the electrolyte, slowly if an Mg anode was used to prevent foaming from hydrogen gas release. For experiments using a Pt anode, discoloration was removed by adding 1 M sodium thiosulfate solution (typically 100 µL for experiments using TBA-Br and 600 µL for experiments involving TBA-I), and the organic layer was extracted. Another cell wash with EtOAc (4 mL) was repeated, followed by another organic layer extraction. Finally, a third EtOAc extraction (1.75 mL) was performed. The combined organic layers were rotary evaporated at 45 °C in a 20 mL glass vial until 80 – 90% of the liquid had evaporated. 3.5 mL of 1 M $NaHCO_3$ was added to basify the solution; for some of the more volatile acids, the organic layers were basified before rotary evaporation in the previous step. The remaining solution, now the aqueous layer, was extracted 2x with hexane (3.5 mL). Then, the aqueous layer was acidified carefully with 1.75 mL of 4 M HCl to avoid excessive degassing of CO_2. The acidified aqueous layer was extracted 3x with Et_2O (2x 3.5 mL, 1x 1.75 mL). The organic layers were combined in a 15 mL Pyrex centrifuge tube (Corning, PTFE-lined phenolic screw caps) and washed 3x with MilliQ water (3x 3.5 mL). These washes were combined in a glass centrifuge tube and extracted once with Et_2O (1.75 mL). The combined organic layers were washed once more with MilliQ water (1.75 mL), dried over $MgSO_4$, and then gravity filtered through VWR qualitative filter paper 413 (5.5 cm) into a 20 mL glass vial. The solution was rotary evaporated at room temperature until the bulk of the solvent was gone and then further concentrated under vacuum for at least 40 min. For some of the more volatile carboxylic acids (b.p. < ~ 240 °C), the last vacuum drying step was skipped to minimize losses due to the very small amounts of product.

^1H and ^{13}C NMR were performed to identify and quantify products. Either ethylene carbonate or 1,3,5-trimethoxybenzene was used as an internal standard for quantification depending on peak overlaps.

5.2.1 Quantification of Ester Yields

The combined hexane layers were washed once with MilliQ, dried with $MgSO_4$, rotary evaporated at 65 °C, and then dried in a vacuum oven for 40 – 60 min. Esters could be quantified via ^1H NMR using an appropriate internal standard (ethylene carbonate or TMB). Any ester not in the hexane would end up in the final isolated carboxylate product and could be added to the amount found in the hexane if needed.

5.3 General Workup Procedure for Experiments without any Magnesium Source (Procedure C)

This workup procedure is similar to **Procedure B** except that the acid was not separated from the other non-polar side products. EtOAc was often used as the initial extraction solvent to ensure maximal acid recovery, although in a few cases, Et_2O was instead to reduce the total number of workup steps.

5.3.1 Workup Procedure for EtOAc (Procedure C1)

The electrolyte was removed from the cell and added to its original 15 mL polypropylene tube. The cell was washed out once with 1 M HBr (4 mL) followed by EtOAc (4 mL) (Note: 1 M HCl could be substituted here as a cheaper alternative instead of 1 M HBr). The washes were combined with the electrolyte, and discoloration was removed by adding 1 M sodium thiosulfate solution (typically 100 µL for experiments using TBA-Br and 600 µL for experiments involving TBA-I), and the organic layer was extracted. Another cell wash with EtOAc (4 mL) was repeated, followed by another organic layer extraction. Finally, a third EtOAc extraction (1.75 mL) was performed. The combined organic layers were rotary evaporated at 45 °C in a 20 mL glass vial until 80 – 90% of the liquid had evaporated. 3.5 mL MilliQ was added, and this aqueous layer was extracted 3x with Et_2O (2x 3.5 mL, 1x 1.75 mL). The organic layers were combined in a 15 mL Pyrex centrifuge tube (Corning, PTFE-lined phenolic screw caps) and washed 3x with MilliQ water (3x 3.5 mL). These washes were combined in a glass centrifuge tube and extracted once with Et_2O (1.75 mL). The combined organic layers were washed once more with MilliQ water (1.75 mL), dried over $MgSO_4$, and then gravity filtered through VWR qualitative filter paper 413 into a 20 mL glass vial. The solution was rotary evaporated at room temperature until the bulk of the solvent was gone and then further concentrated under vacuum for at least 40 min. For some of the more volatile carboxylic acids (b.p. < ~ 240 °C), the last vacuum drying step was skipped to minimize losses due to the very small amounts of product.

5.3.2 Workup Procedure for Et_2O (Procedure C2)

The electrolyte was removed from the cell and added to its original 15 mL polypropylene tube. The cell was washed out once with 1 M HBr (4 mL) followed by Et_2O (4 mL) (Note: 1 M HCl could be substituted here as a cheaper alternative instead of 1 M HBr). The washes were combined with the electrolyte, and discoloration was removed by adding 1 M sodium thiosulfate solution (typically 100 µL for experiments using TBA-Br and 600 µL for experiments involving TBA-I), and the organic layer was extracted. Another cell wash with Et_2O (4 mL) was repeated, followed by another organic layer extraction. Finally, three more Et_2O extractions (3x 1.75 mL) were performed. The organic layers were combined in a 15 mL Pyrex centrifuge tube (Corning, PTFE-lined phenolic screw caps) and washed 3x with MilliQ water (3x 3.5 mL). These washes were combined in a glass centrifuge tube and extracted once with Et_2O (1.75 mL). The combined organic layers were washed once more with MilliQ water (1.75 mL), dried over $MgSO_4$, and then gravity filtered through VWR qualitative filter paper 413 into a 20 mL glass vial. The solution was rotary evaporated at room temperature until the bulk of the solvent was gone and then further concentrated under vacuum for at least 40 min. For some of the more volatile carboxylic acids (b.p. < ~ 240 °C), the last vacuum drying step was skipped to minimize losses due to the very small amounts of product.

^1H and ^{13}C NMR were performed to identify and quantify products. Since the goal of most of these experiments was to determine the ratio of acid to other side products, an internal standard was not always used.

5.4 NMR Analysis

^1H (500.34 MHz), ^{13}C (125.81 MHz), and ^{19}F (470.79 MHz) NMR spectra were collected on a Bruker Avance Neo spectrometer. ^1H NMR spectra were referenced to tetramethylsilane (0 ppm) in $CDCl_3$ and residual deuterated solvent in DMSO-d_6 (2.50 ppm). ^{13}C NMR spectra were referenced to $CDCl_3$ (77.16 ppm) or DMSO-d_6 (39.52 ppm). Quantitative ^1H NMR spectra were typically acquired with 16 scans with an 8 sec acquisition time followed by a 9 sec interscan delay in $CDCl_3$. ^{13}C spectra were acquired with 512 scans, and both ^{13}C and ^{19}F spectra were acquired using a spin-echo procedure to remove interference from the prodigy cryoprobe fluoropolymer of the spectrometer. A full listing of parameters is shown below.

^1H

```
F2 - Acquisition Parameters
Date_              20201110
Time                  20.25 h
INSTRUM              Avance
PROBHD     Z150364_0011 (
PULPROG                zg45
TD                   131072
SOLVENT               CDCl3
NS                       16
DS                        2
SWH                8196.722 Hz
FIDRES             0.125072 Hz
AQ                7.9953918 sec
RG                       64
DW                   61.000 usec
DE                    15.35 usec
TE                    298.1 K
D1               9.00000000 sec
TD0                       1
SFO1            500.3430020 MHz
NUC1                     1H
P1                    11.82 usec
PLW1            11.04599953 W

F2 - Processing parameters
SI                    65536
SF              500.3400000 MHz
WDW                      EM
SSB        0
LB         0 Hz
GB         0
PC                     1.00
```

^{13}C

```
F2 - Acquisition Parameters
Date_              20201110
Time                   1.31 h
INSTRUM              Avance
PROBHD     Z150364_0011 (
PULPROG              zgdepg
TD                    32768
SOLVENT                DMSO
NS                      512
DS                       16
SWH               30120.482 Hz
FIDRES             1.838408 Hz
AQ                0.5439488 sec
RG                      101
DW                   16.600 usec
DE                     6.50 usec
TE                    298.2 K
D1               0.45605120 sec
D11              0.03000000 sec
D21              0.00100000 sec
TD0                       1
SFO1            125.8231741 MHz
NUC1                    13C
CNST18           30.0000000
P0                     3.16 usec
P1                     9.49 usec
P13                 2000.00 usec
PLW1            67.92500305 W
SPNAM[5]        Crp60comp.4
SPOAL5                0.500
SPOFFS5    0 Hz
SPW5             9.34459972 W
SFO2            500.3420014 MHz
NUC2                     1H
CPDPRG[2            waltz65
PCPD2                 80.00 usec
PLW2            11.04599953 W
PLW12            0.24853000 W
PLW13            0.12481000 W
GPNAM[1]          SMSQ10.100
GPZ1                  37.00 %
P16                 1000.00 usec

F2 - Processing parameters
SI                    32768
SF              125.8105931 MHz
WDW                      EM
SSB        0
LB                     1.00 Hz
GB         0
PC                     1.40
```

^{19}F

```
F2 - Acquisition Parameters
Date_              20201110
Time                  20.29 h
INSTRUM              Avance
PROBHD     Z150364_0011 (
PULPROG                zgde
TD                   131072
SOLVENT               CDCl3
NS                       16
DS                        4
SWH              108695.648 Hz
FIDRES             1.658564 Hz
AQ                0.6029312 sec
RG                      101
DW                    4.600 usec
DE                     6.50 usec
TE                    298.2 K
D1               1.00000000 sec
D21              0.00300000 sec
TD0                       1
SFO1            470.7428960 MHz
NUC1                    19F
CNST18           30.0000000
P0                     5.00 usec
P1                    15.00 usec
P13                  120.00 usec
PLW1            10.55300045 W
SPNAM[5]   Bip720,100,10.1
SPOAL5     0
SPOFFS5    0 Hz
SPW5            10.55300045 W

F2 - Processing parameters
SI                    65536
SF              470.7899746 MHz
WDW                      EM
SSB        0
LB                     1.50 Hz
GB         0
PC                     1.00
```

Figure S4. Acquisition parameters for ^1H, ^{13}C, and ^{19}F NMR spectra.

5.5 In-Line Gas Chromatography for CO and H_2 Quantification

An 8610C SRI MultiGas 5 gas chromatograph with in-line sampling capabilities was used to quantify carbon monoxide (CO) and hydrogen (H_2). House N_2 was used as the carrier gas at a flow rate of ~40 mL/min (13 psig setpoint). The sampled gas was injected via a 1 mL sample loop and passed through a 6-foot Haysep D column held at a constant temperature of 85 °C. H_2 was quantified via a thermal conductivity detector (TCD), while CO was quantified with a flame ionization detector (FID) with a pre-methanizer. A stop-flow valve (B) was used to prevent CO_2

from contacting the methanizer. Further sequence details and calibration curves are shown below.

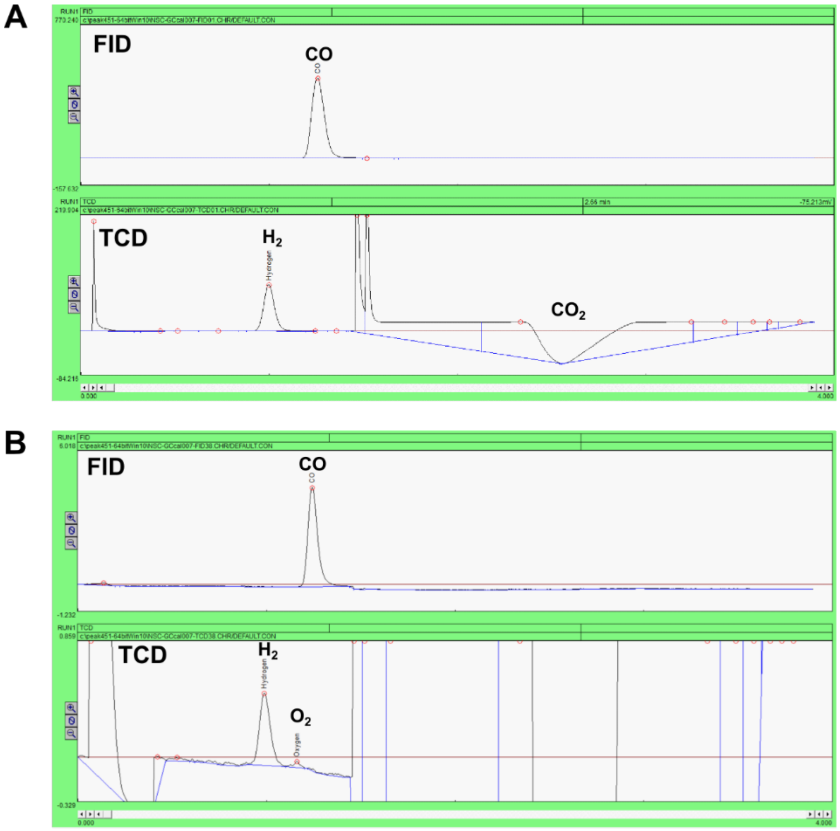

C

FID Events

Time	Event
0.000	ZERO
0.050	F ON (ValveRotate)
1.450	B ON (StopFlow)
1.500	F OFF (ValveRotate)
3.900	B OFF (StopFlow)

TCD Events

Time	Event
0.000	ZERO
0.400	ZERO

Temperature Program

Init temp	Hold	Ramp
85.00	3.900	0.000

Figure S5. Images of FID and TCD chromatograms for (A) 10,000 ppm and (B) 60 ppm. Note that baselines have not been corrected in these examples. (C) Left to right: FID events, TCD events, temperature program.

Calibration curves for CO and H_2 were generated by diluting a standard gas mixture of 10,000 ppm CO, H_2, and CH_4 in CO_2 (Airgas) (the methane signal was cut off by the stop-flow valve) with pure CO_2 at appropriate ratios. Two repeated, consecutive measurements were performed at each dilution level. The data were divided by ppm order of magnitude, and calibration curves were fit by minimizing the sum of squared percent errors. We chose to regress based on percent (or relative) errors rather than absolute errors because the impact of an absolute error depends on the magnitude of the measurement; a 5 ppm error at 1,000 ppm is less harmful (0.5%) than a 5 ppm error at 60 ppm (8.33%). Dividing the data into groups by order of magnitude also helps to minimize percent error, assuming there are enough data points within each order of magnitude for accurate regression.

To convert measured gas concentrations (in ppm) to partial current densities, the following equation was used:

$$j_i = \frac{nFy_i\dot{N}_{tot}}{A_{elec}}$$

where j_i is the partial current density of species i (CO or H_2), n is the number of electrons transferred to form species i ($n = 2$ for both CO and H_2), F is Faraday's constant (96,485 C/mol), y_i is the mole fraction (derived from ppm) for species i measured by the GC, $\dot{N}_{tot}$ is the total molar flow rate of gas set by the mass flow controller, and A_{elec} is the geometric area of the cathode (always 1 cm^2). Faradaic efficiencies were calculated by taking the ratio of the partial current density to the total current density:

$$FE_i = \frac{j_i}{J_{tot}} \times 100\%$$

where FE_i is the Faradaic efficiency of species i and J_{tot} is the total current density.

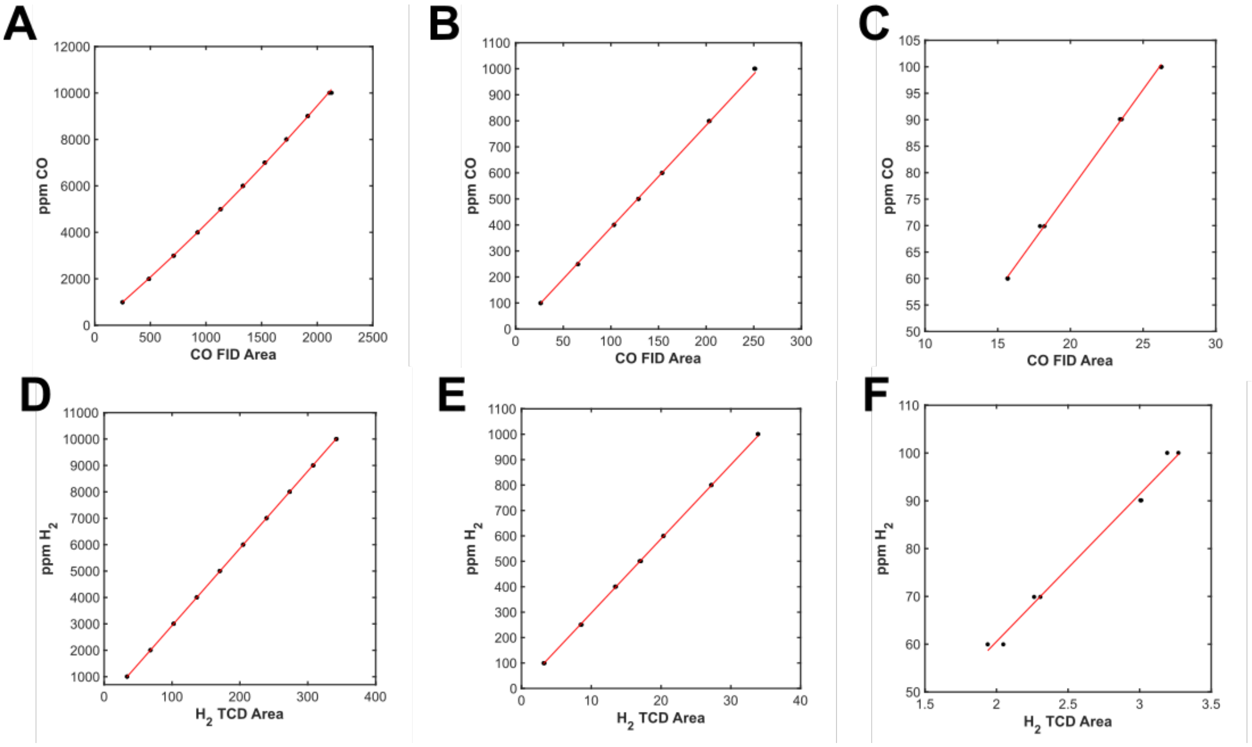

Figure S6. Calibration curves for (A) 1,000 to 10,000 ppm CO, (B) 100 to 1,000 ppm CO, (C) 60 to 100 ppm CO, (D) 1,000 to 10,000 ppm H_2, (E) 100 to 1,000 ppm H_2, (F) 60 to 100 ppm H_2.

Table S1. Calibration equations for H_2 TCD area. Equations are of the form ppm = m × Area + b.

ppm Range	60 to 100	100 to 1,000	1,000 to 10,000
**m**	30.8271	29.1648	29.2455
**b**	-1.1086	5.2508	9.3928

**RMSE (ppm)**	1.56	3.55	6.91
**Max. Absolute Error (ppm)**	2.72	6.67	14.5
**RRMSE[a] (%)**	2.02	0.98	0.106
**Max. Absolute Percent Error (%)**	3.32	2.67	0.229
**R^2**	0.9903	0.9998	1.0000
**No. Points**	8	14	20
**Area Lower Bound[b]**	1.9823	3.2314	33.8905
**Area Upper Bound[c]**	3.2314	33.8905	341.6118

[a] Relative root mean square error, defined as $\sqrt{\frac{\sum_{i=1}^{N}\left(\frac{ppm_{fit}-ppm_{true}}{ppm_{true}}\right)^2}{N}} \times 100\%$, where ppm_{fit} is the ppm predicted from the calibration equation and ppm_{true} is the ppm determined by the dilution of calibration gas. [b] Lowest TCD area corresponding to this calibration equation. [c] Highest TCD area corresponding to this calibration equation.

Table S2. Calibration equations for CO FID area. Equations are of the form $\text{ppm} = \text{a} \times \text{Area}^2 + \text{m} \times \text{Area} + \text{b}$.

**ppm Range**	60 to 100	100 to 1,000	1,000 to 10,000
**a**	0	0	3.4968E-4
**m**	3.7934	3.9331	4.0458
**b**	0.8560	-3.8229	-40.1330
**RMSE (ppm)**	0.504	6.75	39.8
**Max. Absolute Error (ppm)**	1.13	18.04	151.1
**RRMSE[a] (%)**	0.69	0.94	0.49
**Max. Absolute Percent Error (%)**	1.62	1.80	1.51
**R^2**	0.9990	0.9994	0.9998
**No. Points**	8	14	20
**Area Lower Bound[b]**	15.5910	26.2414	251.1167
**Area Upper Bound[c]**	26.2414	251.1167	2,188.4

[a] Relative root mean square error, defined as $\sqrt{\frac{\sum_{i=1}^{N}\left(\frac{ppm_{fit}-ppm_{true}}{ppm_{true}}\right)^2}{N}} \times 100\%$, where ppm_{fit} is the ppm predicted from the calibration equation and ppm_{true} is the ppm determined by the dilution of calibration gas. [b] Lowest TCD area corresponding to this calibration equation. [c] Highest TCD area corresponding to this calibration equation.

5.6 Gas Chromatography-Mass Spectrometry (GC-MS)

GC-MS was performed using a DB-Wax column on a 7890B Agilent GC-MS. A flame ionization detector was used to quantify products. Calibration curves were constructed by making a solution in Et_2O of known composition of the desired compounds and 1,3,5-trimethoxybenzene (TMB). A second Et_2O solution with a known amount of TMB was used to dilute the first solution to generate various ratios of compounds to TMB. Calibration curves were fit by using the FID area and moles of each compound relative to TMB; the high volatility of Et_2O, the solvent used when preparing samples, can introduce errors in the absolute concentrations, so an internal standard needed to be used.

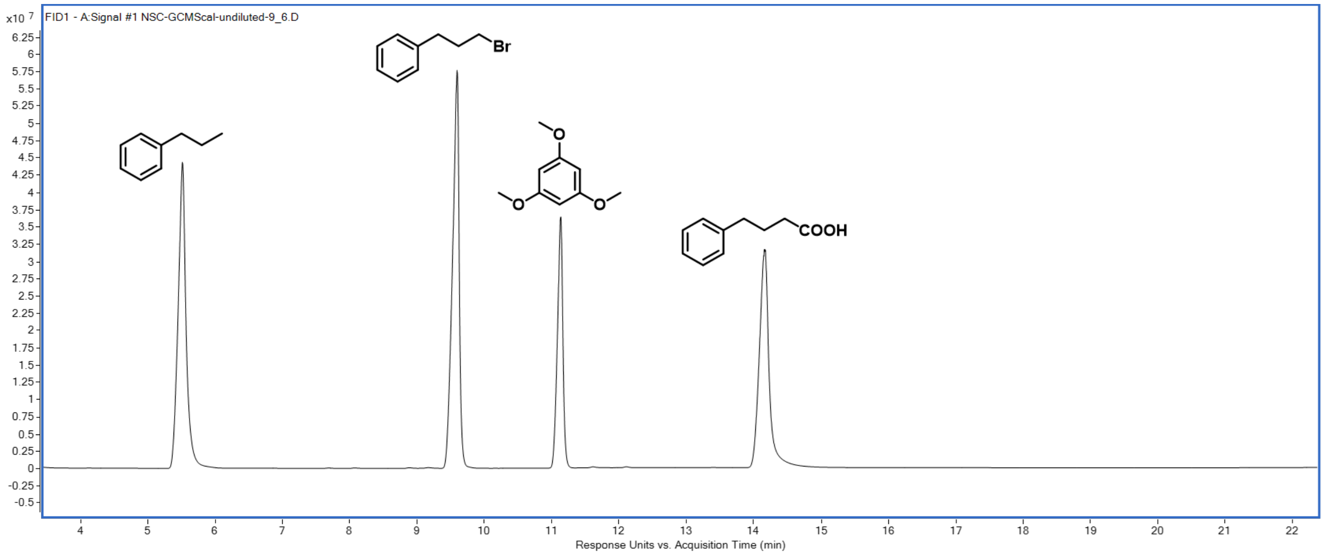

Figure S7. Representative GCMS spectra of a calibration solution of n-propylbenzene, 1-bromo-3-phenylpropane, and 4-phenylbutyric acid with 1,3,5-trimethoxybenzene as the internal standard. Concentrations of species are between 15 – 23 mM.

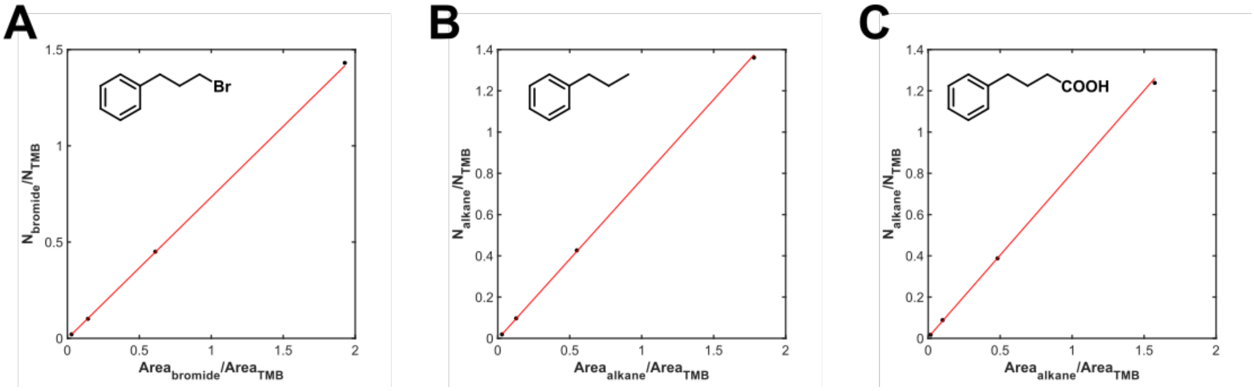

Figure S8. GCMS FID calibration equations for (A) 1-bromo-3-phenylpropane, (B) n-propylbenzene, and (C) 4-phenylbutyric acid.

Table S3. Calibration equations for GCMS FID areas. The equations are of the form $\left(\frac{N_i}{N_{TMB}}\right) = m\left(\frac{Area_i}{Area_{TMB}}\right) + b$.

**Compound**	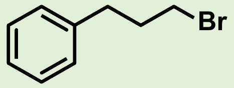	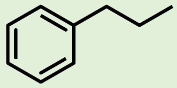	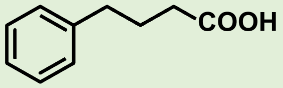
**m**	0.735	0.774	0.798
**b**	-1.10E-3	-3.28E-3	4.65E-3
**RRMSE[a] (%)**	1.11	0.72	1.61
**Max. Absolute Percent Error (%)**	1.87	1.05	2.57

[a] Relative root mean square error, defined as $\sqrt{\frac{\sum_{i=1}^{N}\left(\frac{ppm_{fit}-ppm_{true}}{ppm_{true}}\right)^2}{N}} \times 100\%$, where ppm_{fit} is the ppm predicted from the calibration equation and ppm_{true} is the ppm determined by the dilution of calibration gas.

6 Syntheses of Carboxylate and TBA Salts

6.1 Tetra-n-butylammonium 4-Phenylbutyrate

4-Phenylbutyric acid was weighed into a 5 mL glass vial, and an amount of 1 M TBAOH in methanol was added to give equimolar amounts of acid and base. The solution was shaken and allowed to react for 3 hours. The solvent was removed under reduced pressure by rotary evaporation, and complete drying was achieved in a vacuum oven (VWR 6291 vacuum oven, HFS VP2200 vacuum pump). The final product was an amber-colored, thick gel.

6.2 Magnesium 4-Phenylbutyrate

4-Phenylbutyric acid was weighed into a 20 mL vial, and then 1.2 eq of $MgCO_3$ and ~5 mL of water were added. The mixture was stirred at 700 – 800 rpm at 60 – 70 °C for 3 hours. The water was then boiled off under similar stirring at 120 °C. The residue was dissolved in methanol and centrifuged at 2,000 rpm (VWR Clinical 200). The solution was carefully transferred to another vial to avoid taking up any undissolved solids. The methanol was boiled off at 70 °C under stirring, and the residue was allowed to dry overnight at 80 °C in air. We note that rotary evaporation was avoided since it resulted in significant foaming and bumping.

7 Additional Figures

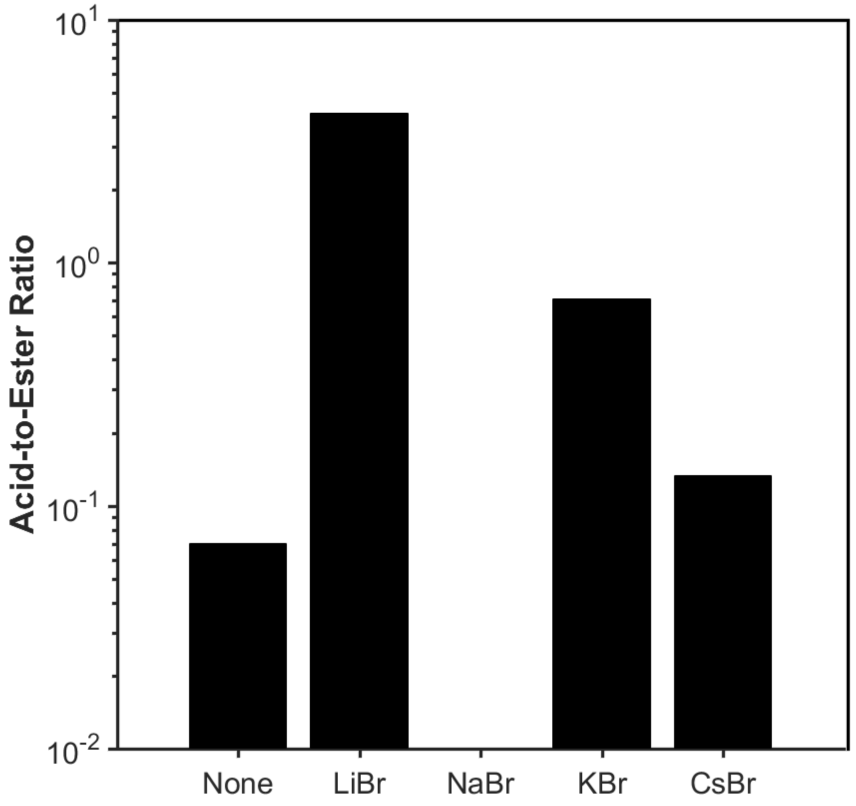

Figure S9. Effect of various alkali metal bromide salts on the acid-to-ester ratio during carboxylation of **1b** in a divided cell. The amount of salt added was enough to make a 0.1 M solution assuming the salt completely dissolved. For comparison, the ratio without any inorganic salts is provided. In the case of NaBr, the voltage quickly reached the potentiostat's limits, suggesting that precipitated Na_2CO_3 was blocking the electrode. KBr and CsBr are limited by their solubility in DMF. Both $MgBr_2$ and $AlBr_3$ resulted in no detectable ester during short-term (1 hr) experiments (i.e. an infinite acid-to-ester ratio).

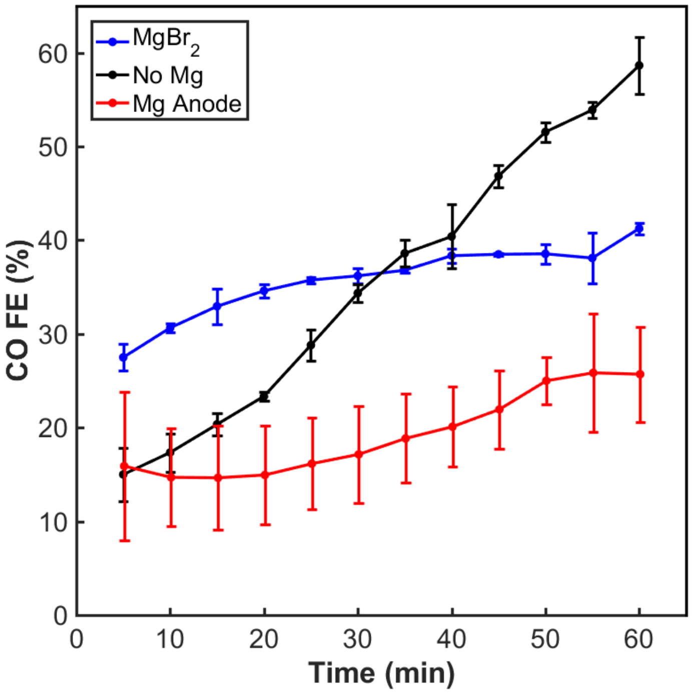

Figure S10. Average CO Faradaic efficiencies over time during low-conversion electrolysis of **1b** with CO_2 at -5 mA/cm^2. Averages involved two data points from independent experiments. Error bars represent sample standard deviations. The increase in CO FE over time may be attributable to depletion of the substrate and diffusion through the Daramic separator for experiments in a two-compartment cell. The larger increase over time for the <u>No Mg</u> experiment likely stems from greater depletion of the substrate as a result of homogeneous nucleophilic reactions. Reaction conditions correspond to those in **Figures 1B** and **1C**.

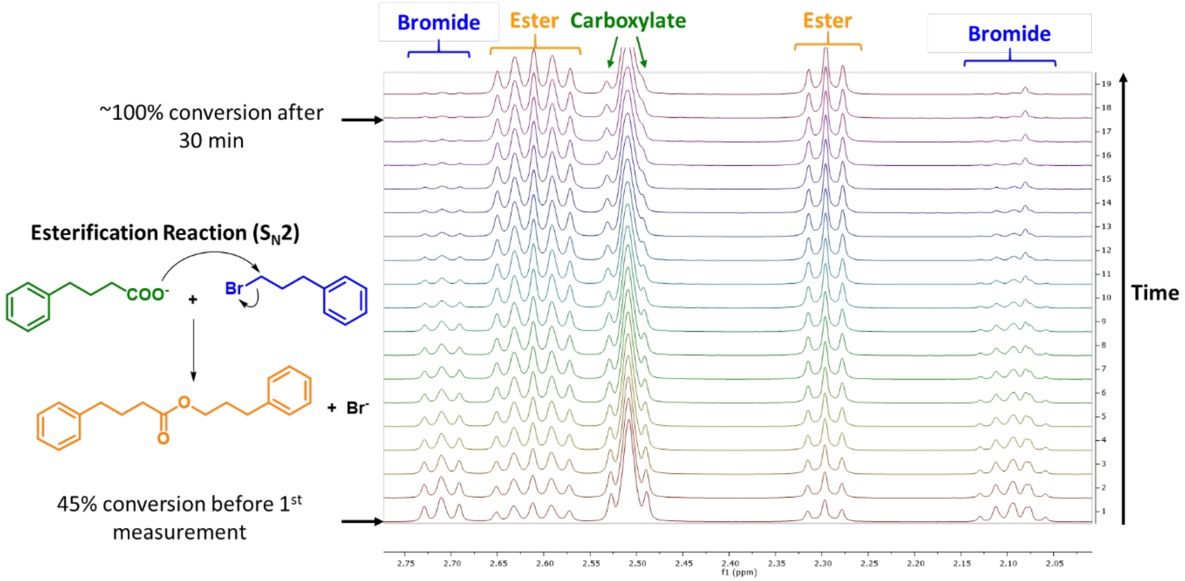

Figure S11. ^{1}H NMR esterification study between **1b** and the TBA salt of **1a** in DMSO-d$_6$. To minimize time between sample prep and the first scan, a blank sample of DMSO-d$_6$ was shimmed, tuned, and locked first. The reaction sample was quickly mixed well in a vial, transferred to an NMR tube, and then inserted, after which scans were immediately started. The first nine experiments were conducted at roughly 58 second intervals, after which the interval was increased to 150 seconds. Since the limiting reactant was the brominated substrate **1b**, all conversions are referenced to the amount of converted **1b**.

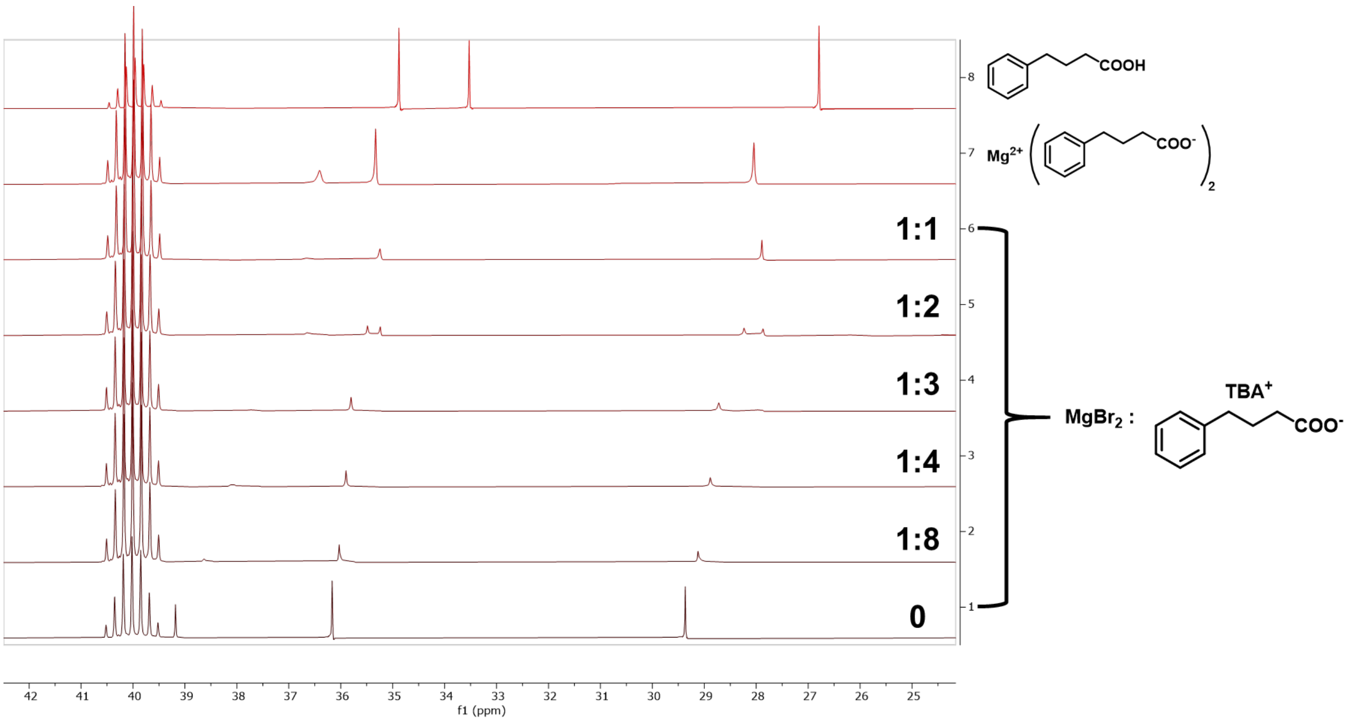

Figure S12. ^{13}C NMR spectra of the aliphatic carbons in 4-phenylbutyrate with various cations. Lower spectra show how the chemical shifts for various ratios of $MgBr_2$ to TBA-4-phenylbutyrate (77 mM). The upper two spectra show the magnesium salt and acidic forms. Spectra were acquired in DMSO-d_6.

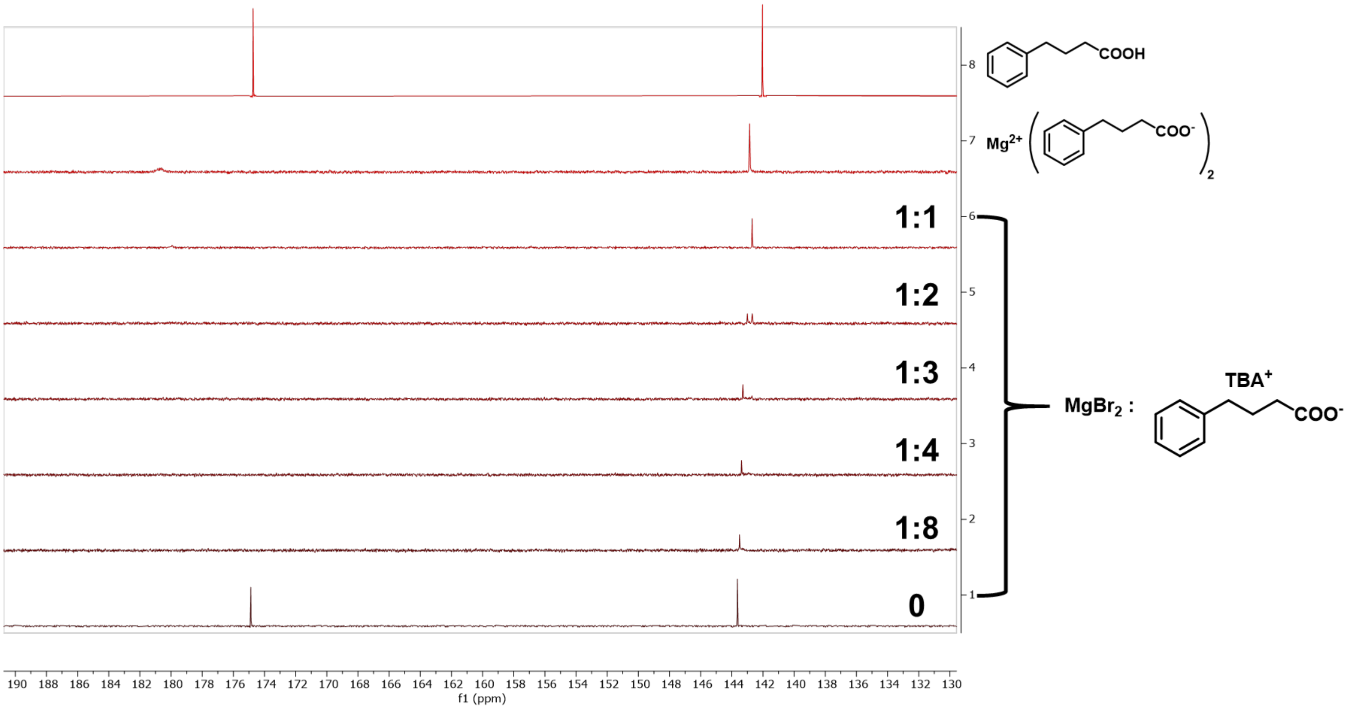

Figure S13. ^{13}C NMR spectra of the carboxylate and tertiary carbons in 4-phenylbutyrate with various cations. Lower spectra show how the chemical shifts for various ratios of $MgBr_2$ to TBA-4-phenylbutyrate (77 mM). The upper two spectra show the magnesium salt and acidic forms. Spectra were acquired in DMSO-d_6. The carboxylate carbon signal is very weak and shifted downfield when Mg^{2+} cations are present.

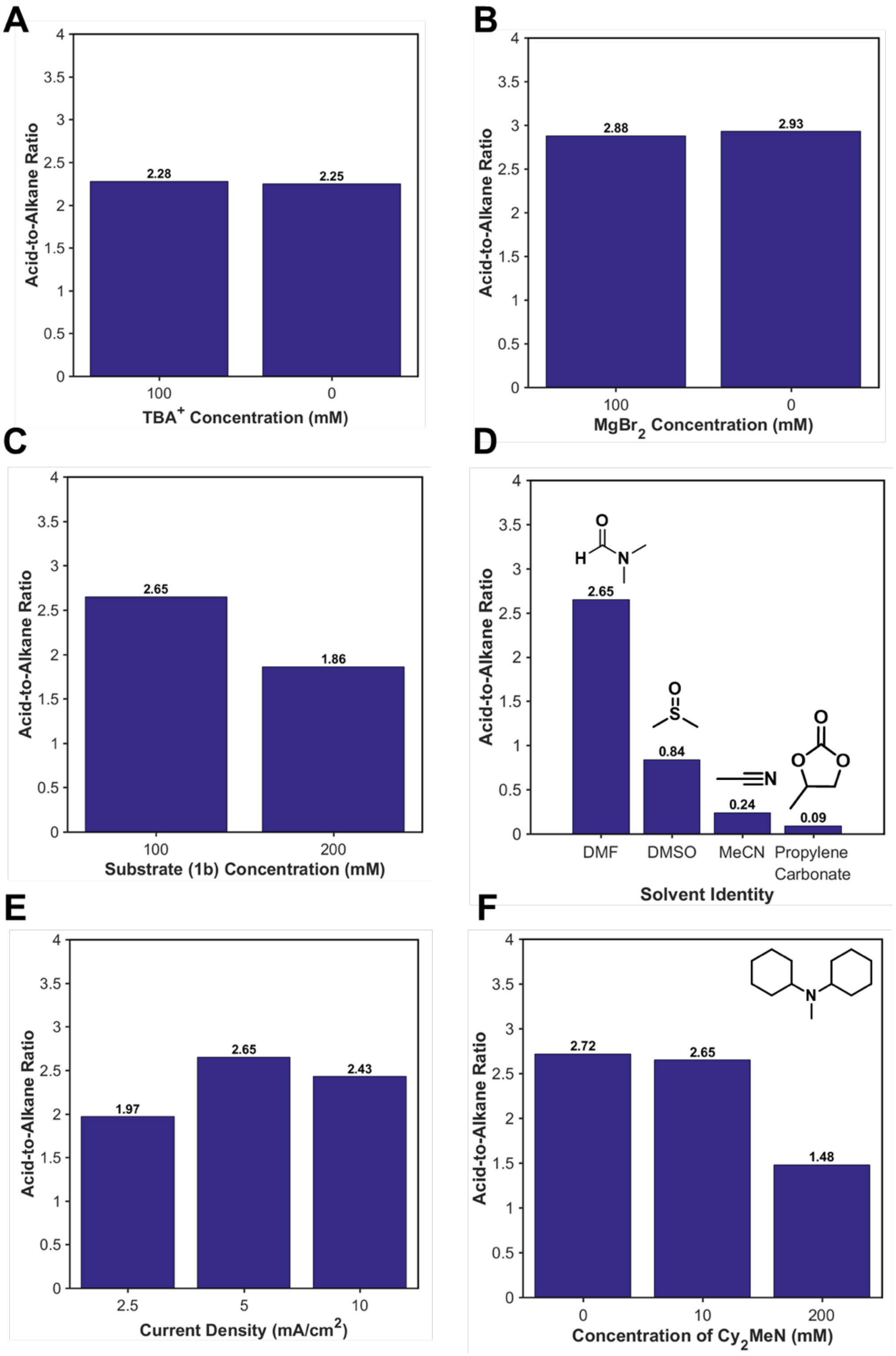

Figure S14. Optimization of the acid-to-alkane ratio (AAR) in two-compartment, sacrificial-anode-free cells under low substrate conversion. Note that these data were not all collected at consistent conditions, so comparisons should only be made within each figure. In particular, many preliminary experiments were conducted with a tertiary amine (N,N-dicyclohexylmethylamine, Cy_2MeN) present, which does decrease the AARs a bit. However, since the deviations across figures is small, the trends in each figure should generalize. (A) Effect of the concentration of

TBA-BF_4 in the catholyte. 70 mM Cy_2MeN was also present. (B) Effect of the $MgBr_2$ concentration in the catholyte. Note that ester was also included into the acid total as a measure of total carboxylation. (C) Effect of the substrate (**1b**) concentration in the catholyte. (D) Effect of the solvent identity. Note that the magnitude of the cell voltage increased noticeably for propylene carbonate. (E) Effect of applied current density, with total charge passed being held constant at 18 C. (F) Effect of Cy_2MeN concentration in the catholyte.

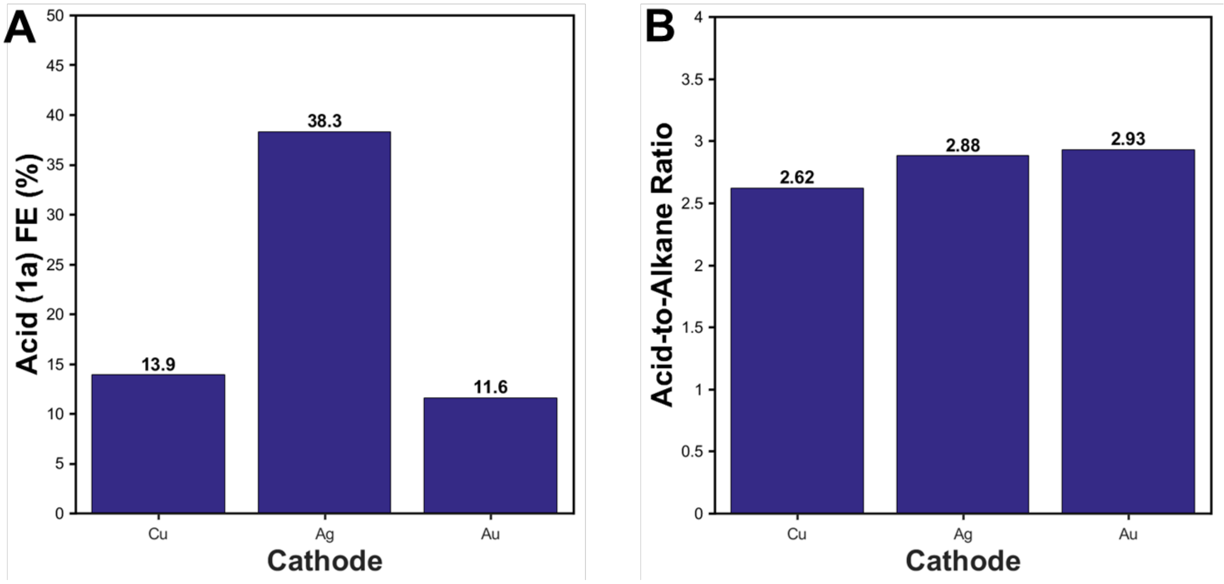

Figure S15. Effect of cathode material on (A) carboxylation Faradaic efficiency and (B) acid-to-alkane ratio. Reaction conditions: divided cell with Daramic separator, -5 mA/cm^2 for 1 hr. Catholyte: 0.1 M substrate (**1b**), 0.1 M $MgBr_2$, 0.1 M TBA-BF_4, 20 sccm CO_2, 2.2 mL DMF. Anolyte: 0.1 M TBA-Br, CO_2 purge, 2.2 mL DMF. While all three electrodes exhibit comparable acid-to-alkane ratios, silver shows a higher carboxylation Faradaic efficiency, making it the best choice for obtaining high yields of carboxylic acid.

Table S4. Results for an attempt at high-yield carboxylation in a divided cell with a Daramic separator.

**Compound**	**Amount in Catholyte (µmol)**	**Amount in Anolyte (µmol)**	**Total Amount (µmol)**
Substrate (**1b**)	2.6	28.7	31.3
Acid (**1a**)	57.1	12.4	69.5
Ester (**1e**)	22.5	6.0	28.5
Alkane (**1c**)	13.4	13.2	26.6
Alcohol (**1d**)	9.7	--	9.7
Substrate Conversion (%)		86	
Acid (**1a**) Yield (%)		31	

Catholyte: 0.1 M TBA-Br, 50 mM TBA-BF_4, 83 mM $MgBr_2$, 10 mM Cy_2MeN, 44.6 mg **1b**, 20 sccm CO_2, 2.2 mL DMF, Ag cathode.
Anolyte: 0.1 M TBA-Br, 50 mM TBA-BF_4, 83 mM $MgBr_2$, N_2 purging, 2.2 mL DMF, Pt anode.
-10 mA/cm^2 for 4 hours.

Table S5. Results for an attempt at high-yield carboxylation in a divided cell with a Celgard separator.

Compound	Amount in Catholyte (µmol)	Amount in Anolyte (µmol)	Total Amount (µmol)
Substrate (**1b**)	9.9	22.8	32.7
Acid (**1a**)	58.2	8.6	66.8
Ester (**1e**)	20.3	6.46	26.8
Alkane (**1c**)	12.3	13.3	25.6
Alcohol (**1d**)	8.6	--	9.7
Substrate Conversion (%)		85	
Acid (**1a**) Yield (%)		31	

Catholyte: 0.1 M TBA-Br, 50 mM TBA-BF_4, 83 mM $MgBr_2$, 10 mM Cy_2MeN, 43.5 mg **1b**, 20 sccm CO_2, 2.2 mL DMF, Ag cathode.
Anolyte: 0.1 M TBA-Br, 50 mM TBA-BF_4, 83 mM $MgBr_2$, N_2 purging, 2.2 mL DMF, Pt anode.
-10 mA/cm^2 for 4 hours.

For these divided cell experiments aimed at getting high carboxylic acid yields, extra electrolyte needed to be added to allow for enough conductivity to pass 10 mA without exceeding the voltage limits of the potentiostat. A small amount of tertiary amine was added to the catholyte, which should have only a small effect and not impact the overall trends. Despite having $MgBr_2$ in both compartments, significant amounts of ester were formed. Additionally, diffusion through the separator over 4 hr proved to be significant; the alkane side product was essentially evenly distributed within the cell. GCMS-FID and ^1H NMR were used to quantify products after workup.

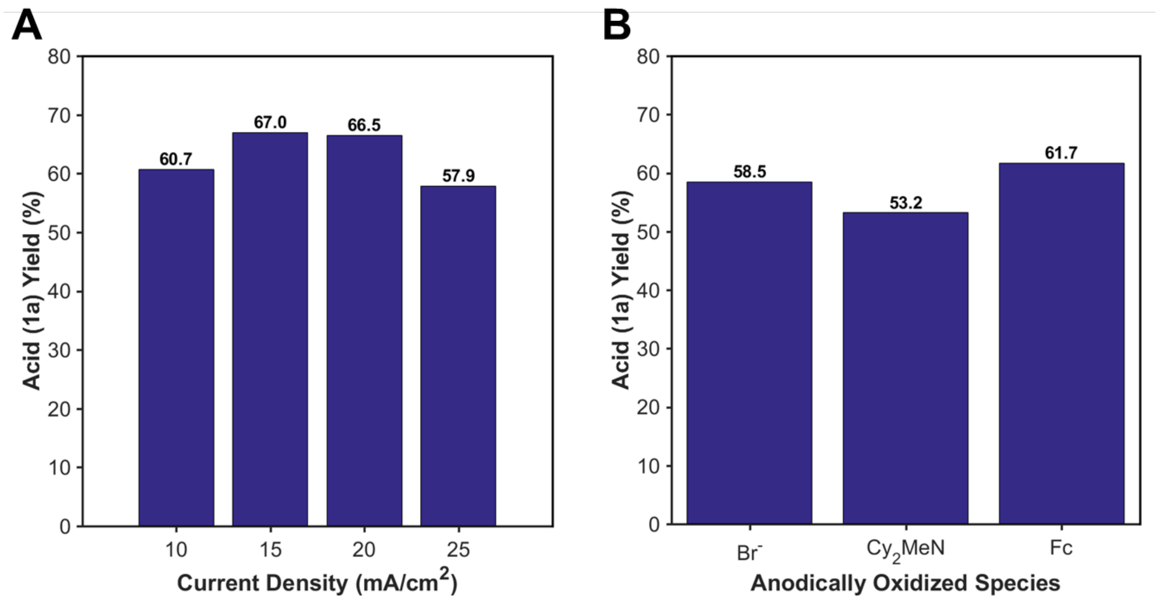

Figure S16. (A) Effect of applied current density on acid yield. Charge passed and substrate conversion for each experiment: 180 C, 95.6% (10 mA/cm^2); 243 C, 97.3% (15 mA/cm^2); 252 C, 97.3% (20 mA/cm^2); 270 C, 96.9 % (25 mA/cm^2). While the charge passed was not held constant, the substrate conversions were nearly all identical, indicating that the differences in charge passed would not amount to more acid yield. Reaction conditions: undivided cell, Ag cathode, Pt anode, 0.1 M TBA-Br, 20 sccm CO_2, 0.1 M **1b**, 83 mM $MgBr_2$, 2.2 mL DMF. The 25 mA/cm^2 experiment was at the voltage limits of the potentiostat. (B) Effect of the anodic oxidation reaction on acid yield. Three different anodic reaction were tested: bromide oxidation, Cy_2MeN oxidation, and ferrocene (Fc) oxidation. Reaction conditions: 10 mA/cm^2 for 4 hr, Ag cathode, Pt anode, 0.1 M **1b**, 83 mM $MgBr_2$, 10 mM Cy_2MeN, 0.1 M anodically oxidized species. For Br^- oxidation, 0.1 M TBA-Br was used, while 0.1 M TBA-BF_4 was used for Cy_2MeN and Fc oxidation. The oxidation of Cy_2MeN produces protons, which is likely why its acid yield was lower and its acid-to-alkane ratio was also lower at 1.76. Fc oxidation does appear to be the best for maximizing acid yield, but its oxidation caused the electrolyte to turn black with a dark precipitate that was difficult to fully remove from the cell. Based on these considerations, Br^- oxidation was chosen as the counter reaction. The discrepancy between **Figures S16A** and **S16B** for the 10 mA/cm^2 Br^- oxidation experiments is likely attributable to the small amount of Cy_2MeN used in the experiments in **Figure S16B**. Note that all yields shown in this figure were determined by GCMS-FID, so they are slightly higher than the finals yields upon further purification in **Scheme 2**.

Table S6. Acid yields for substrates where both a non-sacrificial-anode and sacrificial-anode carboxylation were performed. For benzyl bromide, a sacrificial-anode carboxylation was performed with 0.1 M $MgBr_2$ added to the electrolyte.

Substrate	$MgBr_2$ Pt Anode	Mg Anode	$MgBr_2$ Mg Anode
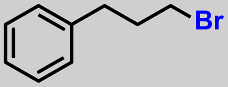	63	61	—
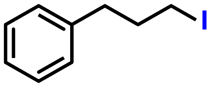	66	32	—
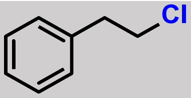	25	11	—
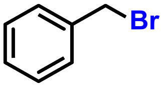	69	53	69
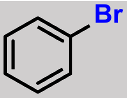	69	80	—

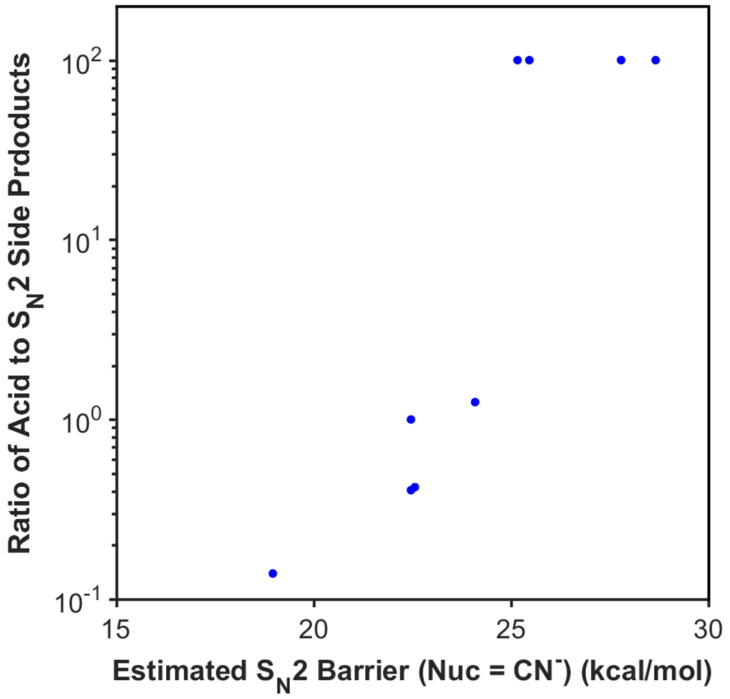

Figure S17. Correlation between estimated S_N2 reaction barriers with CN^- as the nucleophile and observed ratios of acid to S_N2 side products for various organic halides. S_N2 barriers were estimated by combining computational data from multiple sources[1,2] as detailed below. Substrates where no S_N2 products were detected without an Mg^{2+} source had ratios arbitrarily set to 100.

Procedure for estimating S_N2 reaction barriers

The computational S_N2 barriers from the literature did not encompass all of the tested substrates, so many of the barriers needed to be estimated based on barriers of other compounds. Rablen et al. tabulated barriers for various organic chlorides, while Vermeeren et al. tabulated barriers for halogen exchange for various ethyl halides. The data from Vermeeren et al. was used to estimate the change in barrier upon changing the halogen, which could then be combined with the data from Rablen et al. to convert the barrier for an organic chloride to a bromide or iodide. Below is a table showing the consistency of energy differences upon going from chloride to bromide or iodide from Vermeeren et al.

Table S7. Estimated of S_N2 energy barrier differences going from an organic chloride to a bromide or iodide.[2] All energies reported in kcal/mol. The top row corresponds to the nucleophile replacing the halide in the first column.

Organic Halide	Cl^-	Br^-	I^-
R-Cl	13.7	16.2	18.8
R-Br	8.6	11.1	13.3
R-I	5.6	7.4	9.7
Δ (Cl → Br)	-5.1	-5.1	-5.5
Δ (Cl → I)	-3.0	-3.7	-3.6

7.1 Electrochemical Impedance Spectroscopy

Galvanostatic electrochemical impedance spectroscopy (GEIS) was performed at an applied current density of -5 mA/cm^2. A reference electrode consisting of a platinum wire in a solution of 10 mM I_2/10 mM TBA-I_3^- in DMF was used. The reference electrode holder was a 4 mm O.D. glass tube with a porous Teflon frit (CH Instruments). These experiments were performed in undivided cells with a silver foil cathode and platinum foil anode. The electrolyte consisted of 0.1 M TBA-Br in DMF with a 20 sccm CO_2 purge.

GEIS was performed from 50 kHz to 20 Hz at eight points per decade (logarithmic spacing) using a 100 μA sinus amplitude. A waiting time of 0.1 of a sinus period was set before data acquisition. An average of 16 measures was calculated for each frequency. GEIS scans were performed every five minutes, with a constant current density of -5 mA/cm^2 passed in the interim periods.

During analysis of the GEIS spectra, impedances with positive imaginary components at the low frequency end were ignored during fitting (**Figure S18A**). While the source of these positive imaginary components is unknown, low-frequency impedances can be susceptible to fluctuations in mass transport (e.g. from bubbling) or non-steady state behavior, both of which were occurring in the system. These data also have increased levels of noise compared to other impedances of similar magnitude. Nonetheless, qualitative trends for solution resistances, capacitances, and charge-transfer resistances fitted from the truncated dataset should be trustworthy.

Data fitting was performed with the ZFit module as part of the EC-Lab software from Biologic. The randomize + simplex fitting algorithm was used with 5,000 iterations and weighting based on impedance (there was little difference in the fitted parameters if the impedance values were unweighted). The optimizer minimized the χ^2 value. Parameter uncertainties, denoted as “dev” in ZFit, can be viewed similarly to standard deviations.

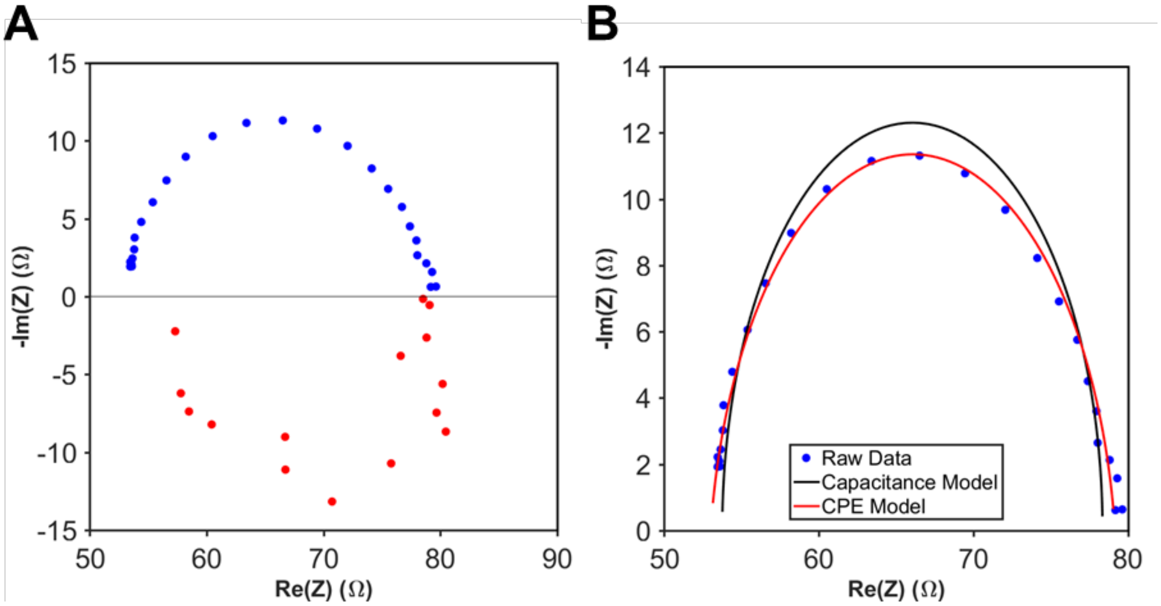

Figure S18. Data selection and model validation using the GEIS spectra at t = 0 with no added magnesium or carboxylate. (A) Depiction of the data points being kept for analysis with Im(Z) < 0 (blue) and of the points excluded from fitting with Im(Z) > 0 (red). (B) Comparison of model fits on the selected data points using an ideal capacitor (black) or a constant phase element (CPE) (red).

**Ideal capacitor model ($R_1 + R_2/C_2$)**							
	Fitted Values			"Dev"			
R_s (Ω)	C_{dl} (F)	R_{ct} (Ω)	R_s (Ω)	C_{dl} (F)	R_{ct} (Ω)	χ^2	$\frac{\chi^2}{\sqrt{N}}$
53.75	5.62 x 10^{-6}	24.61	0.3339	5.01 x 10^{-7}	0.375	19.83	0.8733

**CPE model ($R_s + R_{ct}/Q_{dl}$)**									
	Fitted Values				"Dev"				
R_s (Ω)	Q_{dl} (F·s^{a-1})	a_{dl}	R_{ct} (Ω)	R_s (Ω)	Q_{dl} (F·s^{a-1})	a_{dl}	R_{ct} (Ω)	χ^2	$\frac{\chi^2}{\sqrt{N}}$
53	1.18 x 10^{-5}	0.9098	26.17	0.4206	8.87 x 10^{-6}	0.745	0.929	5.796	0.4721

At the outset, two different types of models were evaluated to fit the data in **Figure S18**. Both models involved a resistor (modeling the electrolyte resistance, R_s) in series with a parallel combination of a resistor (resistance to charge transfer at the electrode, R_{ct}) and either a capacitor (C_{dl}) or a constant phase element (CPE, Q_{dl} & a_{dl}) to capture the double-layer capacitance. The analytical forms of the impedance of these models is given below where *f* is the applied frequency in Hz.

Ideal Capacitor Model
$$Z = R_s + \frac{R_{ct}}{1 + 2\pi f R_{ct} C_{dl} j} \quad (1)$$

CPE Model
$$Z = R_s + \frac{R_{ct}}{1 + R_{ct} Q_{dl} (2\pi f j)^{a_{dl}}} \quad (2)$$

The CPE model replaces the capacitance C with two parameters Q and a. The capacitance of the CPE model can be calculated using the following equation[3]

$$C_{dl} = R_s^{(1-a)/a} Q^{1/a} \tag{3}$$

where R_s is the solution resistance. The calculated C_{dl} value for the CPE model using Eq. 3 is 5.69×10^{-6} F, in good agreement with the value of 5.62×10^{-6} F obtained from the ideal capacitor model.

The CPE model does provide a better fit to the data than does the ideal capacitor model (**Figure S18B**), but the extracted resistances and capacitances are nearly identical between the models. Moreover, the uncertainties in Q_{dl} and a_{dl} are rather large, whereas the uncertainty in C_{dl} is low. Given these observations, we opted to use the simpler ideal capacitor model since it provides equivalent information for our purposes with fewer model parameters.

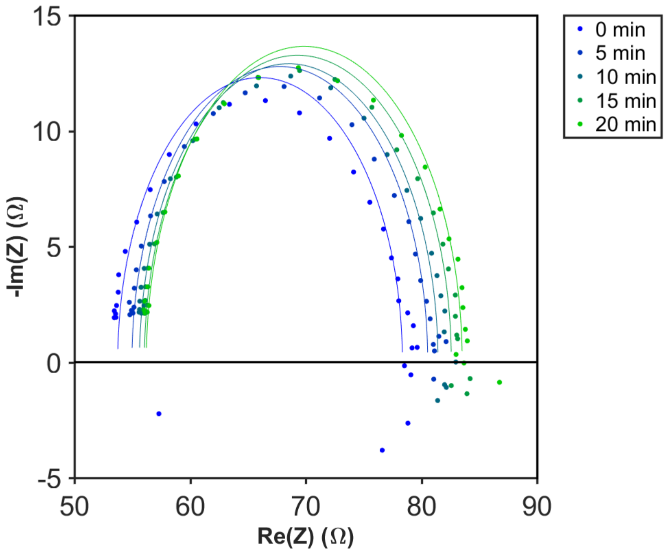

Figure S19. GEIS spectra at 5 min intervals at -5 mA/cm^2 during electrolysis with no added magnesium or carboxylate. Points represent experimental data, and solid lines represent fits to the data using the ideal capacitor model $R_s + R_{ct}/C_{dl}$. As indicated earlier, data points with positive imaginary impedances were ignored during fitting.

**Fitted parameters for R_s+R_{ct}/C_{dl} model during electrolysis without Mg or carboxylate**								
Time (min)	Fitted Values			"Dev"			χ^2	$\frac{\chi^2}{\sqrt{N}}$
	R_s (Ω)	C_{dl} (F)	R_{ct} (Ω)	R_s (Ω)	C_{dl} (F)	R_{ct} (Ω)		
0	53.75	5.62×10^{-6}	24.61	0.3339	5.01×10^{-7}	0.375	19.83	0.8733
5	54.98	5.13×10^{-6}	25.57	0.3299	4.28×10^{-7}	0.3809	23	0.9063
10	55.63	5.07×10^{-6}	25.81	0.3382	4.47×10^{-7}	0.3271	14.67	0.7818
15	56.05	5.07×10^{-6}	26.54	0.3351	4.26×10^{-7}	0.3546	15.39	0.7847
20	56.22	5.13×10^{-6}	27.31	0.3289	4.00×10^{-7}	0.3814	31.81	1.066

The ideal capacitor model $R_s + R_{ct}/C_{dl}$ was used to fit the GEIS spectra from electrolysis without any added magnesium or carboxylate. During this electrolysis, the main cathodic reaction would be CO_2 reduction, with possibly some bromine reduction over time as it transports from the

anode. The circuit parameters indicate only small changes occur over the timespan of 20 min. Small increases in the solution resistance and charge-transfer resistance occur, and the capacitance remains mostly constant. These data serve as a background to compare the effects of adding a magnesium salt and carboxylate salt.

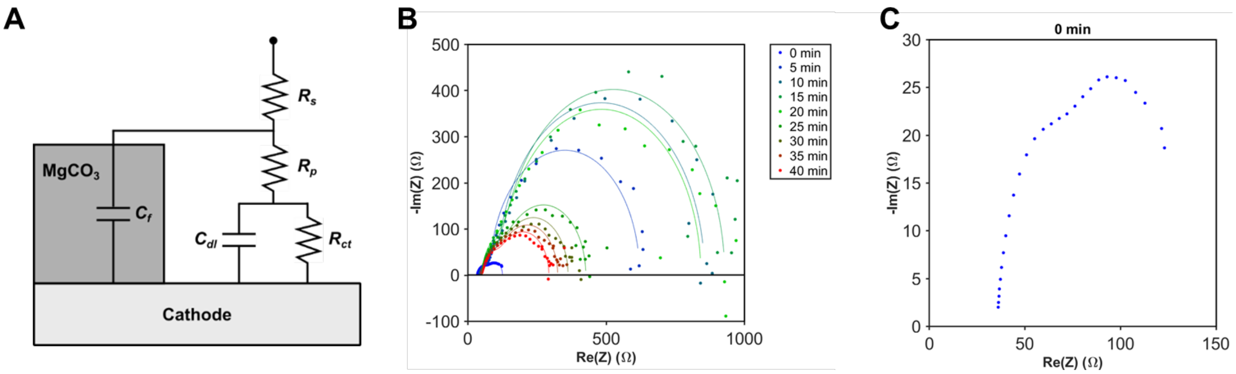

Figure S20. (A) Equivalent circuit model of a partially blocked electrode. (B) GEIS spectra (points) and model fits (lines) during electrolysis with 0.1 M $MgBr_2$ in the electrolyte. After 20 min, 50-100 mM TBA 4-phenylbutyrate was added to the electrolyte. (C) Zoom-in of the initial GEIS spectra showing two arcs are present in the spectra.

Fitted parameters for surface passivation model												
Time (min)	Fitted Values					"Dev"						
	R_s (Ω)	C_f (F)	R_p (Ω)	C_{dl} (F)	R_{ct} (Ω)	R_s (Ω)	C_f (F)	R_p (Ω)	C_{dl} (F)	R_{ct} (Ω)	χ^2	$\frac{\chi^2}{\sqrt{N}}$
0	37.09	2.59 x 10^{-6}	42.58	3.87 x 10^{-5}	45.94	0.3295	2.22 x 10^{-7}	2.713	5.95 x 10^{-6}	2.941	262.9	3.064
5	43.95	2.32 x 10^{-6}	51.77	1.40 x 10^{-5}	525.8	0.3252	1.34 x 10^{-7}	1.803	1.03 x 10^{-7}	1.840	1.61 x 10^{4}	21.14
10	49.07	2.11 x 10^{-6}	101.8	8.38 x 10^{-6}	705.4	0.3257	7.13 x 10^{-8}	3.187	5.61 x 10^{-8}	3.249	4.61 x 10^{4}	36.29
15	51.41	1.76 x 10^{-6}	152.8	4.97 x 10^{-6}	724.8	0.3237	4.67 x 10^{-8}	5.458	4.77 x 10^{-8}	5.517	5.36 x 10^{4}	38.59
20	51.67	1.84 x 10^{-6}	173.6	4.60 x 10^{-6}	617.8	0.3224	4.73 x 10^{-8}	7.245	7.22 x 10^{-8}	7.302	1.04 x 10^{4}	53.62
25	52.00	1.97 x 10^{-6}	142.5	8.44 x 10^{-6}	232.4	0.324	6.07 x 10^{-8}	6.401	4.61 x 10^{-7}	6.438	1.28 x 10^{4}	19.15
30	52.01	2.08 x 10^{-6}	124.4	9.32 x 10^{-6}	186.3	0.3248	7.40 x 10^{-8}	6.480	6.53 x 10^{-7}	6.516	7.41 x 10^{3}	14.55
35	52.37	2.06 x 10^{-6}	110.0	9.68 x 10^{-6}	162.9	0.3251	8.15 x 10^{-8}	6.169	7.47 x 10^{-7}	6.213	5.71 x 10^{3}	12.6
40	52.84	2.04 x 10^{-6}	101.4	1.05 x 10^{-5}	139.3	0.3252	8.70 x 10^{-8}	5.914	9.35 x 10^{-7}	5.959	1.02 x 10^{4}	16.79

Adding 0.1 M $MgBr_2$ causes significant changes in the GEIS spectra over time (**Figure S20**). Notably, after the addition of TBA 4-phenylbutyrate after 20 min, the over impedance decreases. To model these spectra, a partially blocked electrode model was adapted from the literature.[4] The necessity of the additional parameters is also justified based off of the presence of two arcs in the initial GEIS spectra (**Figure S20C**). The impedance of the equivalent circuit in **Figure S20A** is given below:

$$Z = R_s + \frac{R_{ct} + R_p(1 + 2\pi f C_{dl} R_{ct} i)}{1 + 2\pi f C_{dl} R_{ct} i + 2\pi f C_f i \left(R_{ct} + R_p(1 + 2\pi f C_{dl} R_{ct} i)\right)} \quad (4)$$

The model fitting is somewhat worse than in the case without Mg or carboxylate, possibly due to the buildup of $MgCO_3$ during the measurements. There is also a visible disappearance of the

second circular arc after 15 min. Given these limitations of the data and the model, we have only interpreted the high- and low-frequency intercepts.

7.2 Fourier Transform Infrared Spectroscopy (FTIR)

FTIR experiments were performed on a Bruker Alpha II FTIR spectrometer with a diamond ATR crystal. Solid samples were pressed against the ATR crystal by a pressure arm. Spectra were collected from 400 to 4,000 cm^{-1} with 32 scans. Background spectra were collected before solids were applied to the crystal.

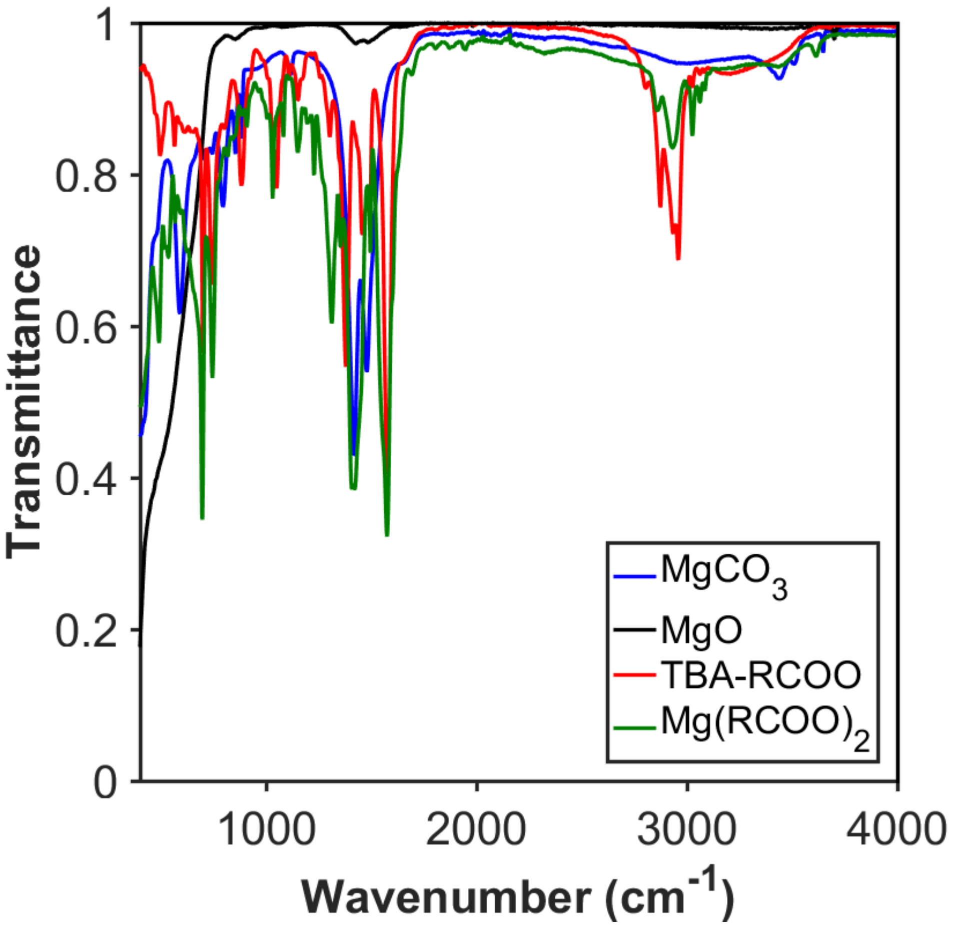

Figure S21. FTIR spectra of standard compounds. R = 3-phenylpropyl.

Compound	Observed Peak Locations (cm^{-1})
$MgCO_3$	589, 794, 853, 884, 1117, 1417, 1478, 3438, 3510, 3648
MgO	853, 1427, 1482, 3702
TBA-RCOO	495, 565, 699, 744, 882, 1030, 1049, 1150, 1303, 1379, 1457, 1490, 1577, 2873, 2935, 2960
$Mg(RCOO)_2$	491, 697, 744, 1030, 1150, 1228, 1311, 1408, 1420, 1496,1575, 2930, 3026

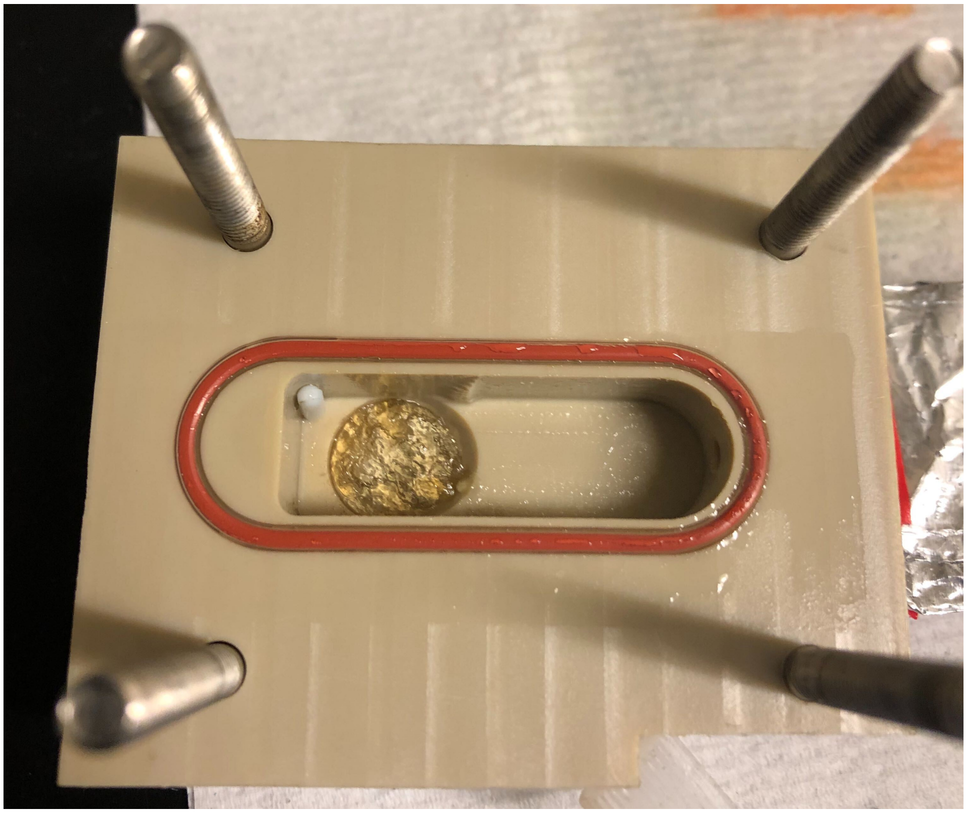

Figure S22. Picture of the cell and cathode after electrocarboxylation with a magnesium anode

8 Scanning Electron Microscopy (SEM)

The silver foils were attached to SEM pins with carbon tape. The pins were inserted into an 8-pin stub multiholder. The samples were imaged using a Zeiss Merlin High-Resolution SEM. A working distance of approximately 6 mm was used for imaging. A gun beam voltage and current of 1.3 kV and 25 pA were used, respectively. The beam parameters were chosen to minimize charging of the relatively non-conductive residual material on the electrode. For each sample, several locations to image were selected at low magnification (50x – 250x), after which progressively higher magnification images were taken, up to the highest magnification at which features could be well resolved (~40,000x). The imaged features were found over the entire sample area and are representative of the entire sample surface.

9 Carboxylation Mechanistic & Control Experiments

9.1 Divided Cell with a Magnesium Anode

To avoid complications from oxidized products while still observe products that form in the absence of a protecting cation, a two-compartment experiment using a Daramic separator and a sacrificial anode was performed. While the sacrificial anode would produce protecting magnesium cations, the separator would delay their transport into the catholyte, allowing nucleophilic reactions to occur for a period of time.

Both compartments contained the same electrolyte: 0.1 M benzyl bromide, 0.2 M TBA-BF_4, and 2.2 mL DMF. 20 sccm CO_2 was bubbled into the catholyte, while N_2 was purged into the anolyte for 10 min and then turned off to minimize convective transport of Mg cations to the catholyte. 15 mA of current was passed until voltage limits (V < -10 V) were reached; afterwards, the cell was held at -10 V for about an hour. The electrolyte from both compartments was removed, and 5.25 mL of MilliQ was added. 3.5 mL of Et_2O was then added, and the organic layer was extracted. Then, ~ 100 µL of 3 M NaOH was added to the aqueous layer. This basification step was performed to ensure capture of any amine side products (which would normally be missed by the workups with an acidic aqueous layer. We do note that later experiments revealed adding base can form amines from DMF decomposition); this step occurred after the first organic extraction to minimize contact of benzyl bromide with hydroxide which could lead to undesired alcohol formation. Once basified, the aqueous layer was extracted 4x with Et_2O (1x 3.5 mL, 3x 1.75 mL). The combined organic layers were washed 3x with 3.5 mL MilliQ. The combined aqueous washes were extracted once with 1.75 mL Et_2O, then the combined organic layers were washed one last time with 1.75 mL of MilliQ. The organic layers were rotavapped until about 2 – 2.5 mL remained, dried over $MgSO_4$, and analyzed by GC-MS. To prepare the sample for NMR, the GC-MS sample was combined with the rest of the crude, gravity filtered through VWR qualitative filter paper, and rotavapped until ~ 5 min had passed once the solvent appeared visibly gone (~ 8 – 10 min in total). We avoided vacuum drying to minimize the loss of more volatile products seen on the GC-MS.

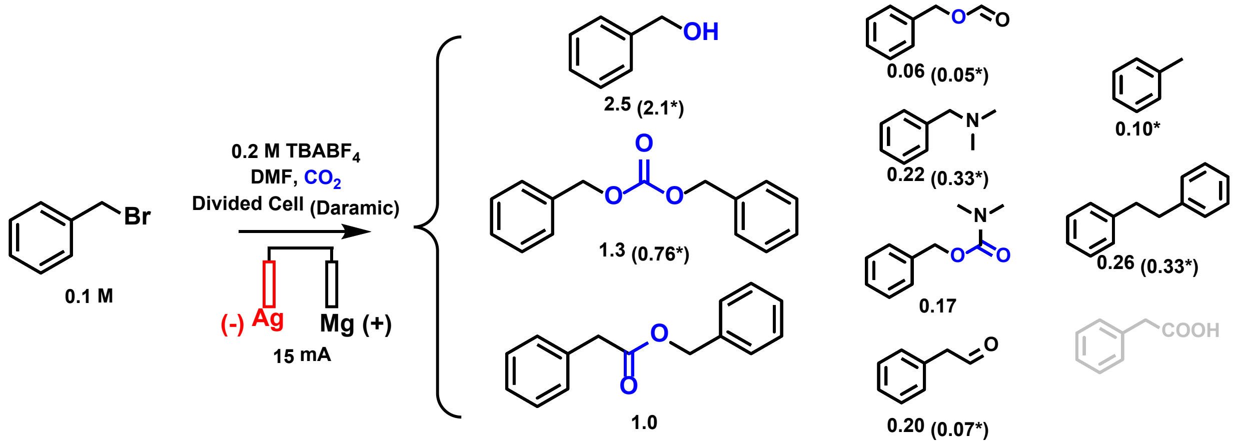

Figure S23. Product distribution from benzyl bromide carboxylation in a divided cell with an Mg anode. Values are normalized to the amount of ester. Unmarked values correspond to those determined by ^{1}H NMR; values with an asterisk are those determined by GC-MS-FID.

The carboxylation of benzyl bromide in this setup yielded a wide variety of products as shown in **Figure S23**. The alcohol, carbonate, and ester were the major products from nucleophilic reactions with benzyl bromide. Several side products were observed that are likely from reactions with DMF. Bibenzyl can form via radical-radical coupling (non-nucleophilic), or a nucleophilic attack of a benzyl anion on benzyl bromide; toluene forms via a hydrogenation reaction most likely involving the solvent. Phenylacetic acid is grayed out because it could have been present but would not have been extracted from the basic aqueous layer. Ratios relative to the ester are displayed from both ^{1}H NMR (unmarked) and GC-MS-FID (marked); both values

are indicated because while ^{1}H NMR is more accurate, some of the more volatile products are lost during rotary evaporation. Specific product identification details are presented below. There are some unassigned NMR and GC-MS peaks, so there could be additional side products.

Benzyl 2-phenyl acetate: ^{1}H NMR δ 3.68 (s, 2H), 5.14 (s, 2H), 1 : 1.02 area ratio. ^{1}H – ^{13}C HSQC (3.68, 41.30), (5.14, 66.45).[5] MS (*m/z*) 226 (M^+).

Dibenzyl carbonate: ^{1}H NMR δ 5.17 (s, 4H). ^{13}C NMR δ 155.11. ^{1}H – ^{13}C HSQC (5.17, 69.58).[5] No reference MS spectra, could not detect M^+ ion.

Benzyl alcohol: ^{1}H NMR δ 4.70 (s, 2H), ^{1}H – ^{13}C HSQC (4.70, 65.28). MS (*m/z*) 108 (M^+).

Benzyl formate: ^{1}H NMR δ 8.15 (t, J = 0.9 Hz, 1H), 5.21 (d, J = 0.9 Hz, 2H), 1 : 2.1 area ratio.[6] ^{1}H – ^{1}H COSY can barely detect a resonance at (8.15, 5.21). MS (*m/z*) 136 (M^+).

While the proton chemical shifts are not exactly in agreement, their splitting patterns, coupling constants, and area ratios support the presence of benzyl formate.

N,N-Dimethylbenzylamine: ^{1}H NMR δ 3.45 (s, 2H), 2.25 (s, 6H), 1 : 3.0 area ratio. ^{1}H – ^{13}C HSQC (2.24, 45.39), (3.45, 64.31).[7] MS (*m/z*) 135 (M^+).

Benzyl N,N-dimethylcarbamate: ^{1}H NMR δ 5.13 (s, 2H), 2.94 (s, 6H), 1 : 2.7 area ratio. ^{1}H – ^{13}C HSQC (2.94, 36.03).[8] MS (*m/z*) 179 (M^+).

The ^{1}H NMR peaks for this compound are very close to other peaks (bibenzyl, ester), and the GCMS peak is very overlapped by bibenzyl. Nonetheless, there appears to be enough spectral information to suggest the presence of this compound. There are two ^{1}H singlets at 2.94 and 2.93 ppm, one for the carbamate and one for bibenzyl. Additionally, the MS spectrum for bibenzyl does not contain *m/z* 179.

Phenylacetaldehyde: ^{1}H NMR δ 9.76 (t, J = 2.4 Hz, 1H), 3.69 (d, J = 2.4 Hz, 2H), 1.8 : 1 area ratio. ^{1}H – ^{1}H COSY (9.75, 3.69). ^{1}H – ^{13}C HSQC (9.75, 199.74), (3.69, 50.46).[9] MS (*m/z*) 120 (M^+).

The GCMS peak is overlapped by benzyl bromide, but there is no peak at *m/z* 120 for benzyl bromide, which supports the presence of phenylacetaldehyde.

Toluene: MS (*m/z*) 92 (M^+).

The relatively high volatility of toluene coupled with rotary evaporation likely greatly reduced is presence in NMR. There is a HSQC coupling that matches well with the expected shifts for toluene, but it is more likely to be from BHT based on integration area. GCMS provides fairly definitive confirmation for toluene, however.

Bibenzyl: ^{1}H NMR δ 2.93 (s, 4H). ^{1}H – ^{13}C HSQC (2.93, 37.98).[10] MS (*m/z*) 182 (M^+).

As discussed above, the ^{1}H NMR and GCMS peaks for bibenzyl are very close to ones for the benzyl carbamate. However, the spectral information here enables unique identification of both species.

^{1}H NMR

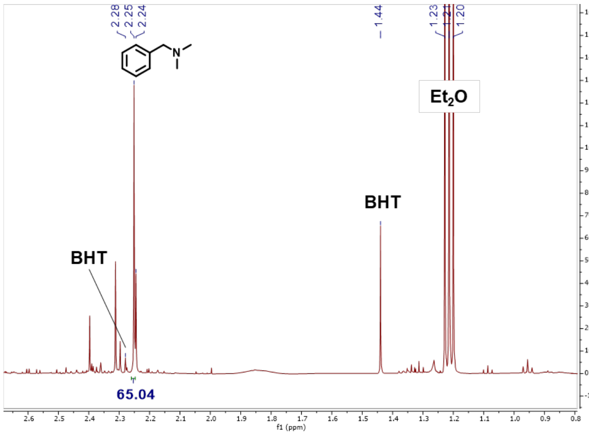
2.28
2.25
2.24
1.44
1.23
1.20
Et₂O
BHT
BHT
65.04
f1 (ppm)

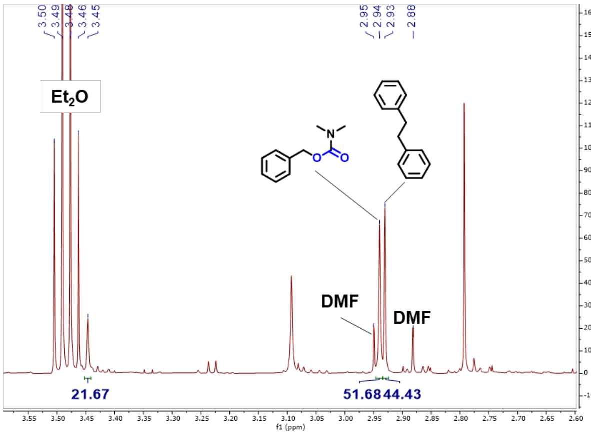
3.50
3.49
3.46
3.45
2.95
2.94
2.93
2.88
Et₂O
DMF
DMF
21.67
51.68
44.43
f1 (ppm)

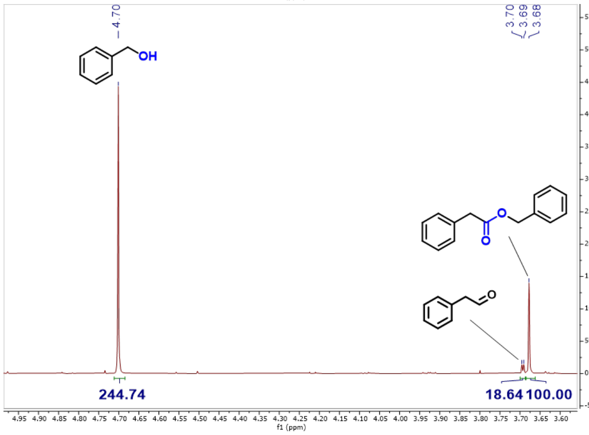
4.70
3.70
3.69
3.68
244.74
18.64
100.00
f1 (ppm)

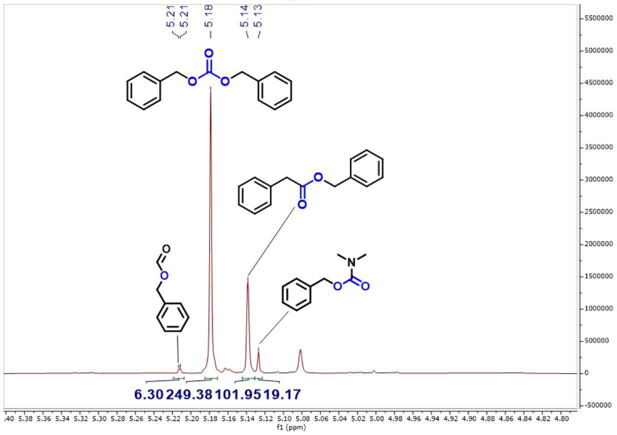
5.21
5.21
5.18
5.14
5.13
6.30
249.38
101.95
19.17
f1 (ppm)

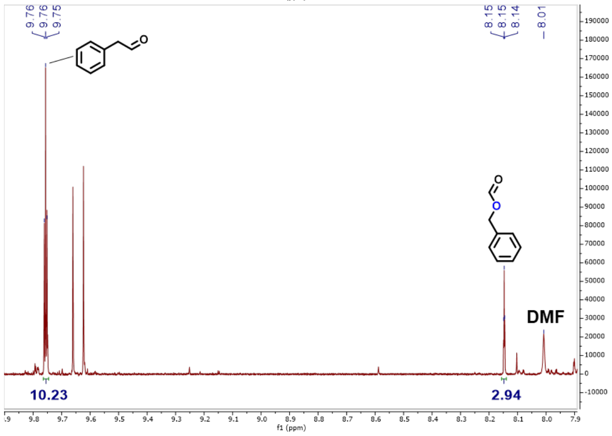
9.76
9.76
9.75
8.15
8.15
8.14
8.01
DMF
10.23
2.94
f1 (ppm)

$^{1}H - ^{13}C$ NMR

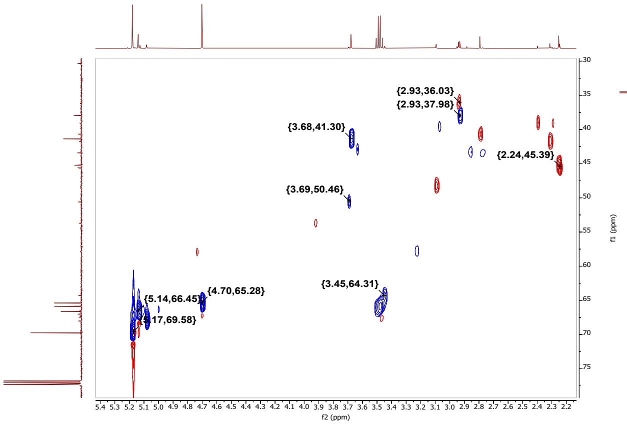

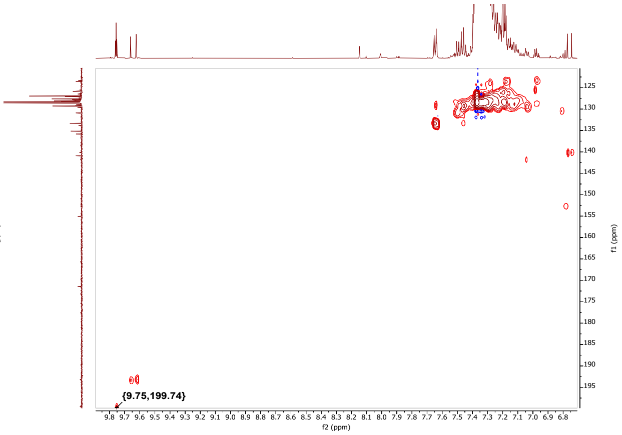

$^{1}H - ^{1}H$ NMR

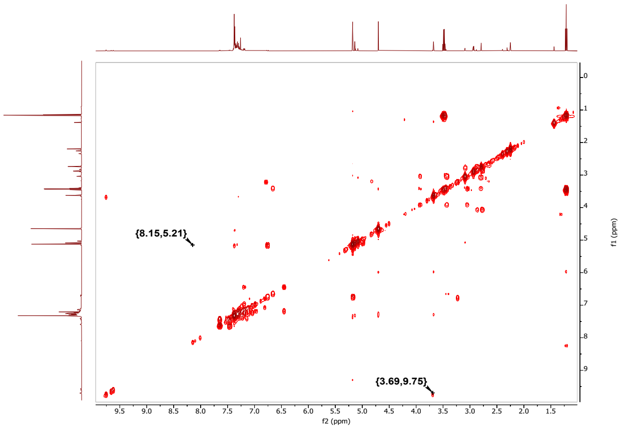

9.2 $^{13}CO_2$ Experiment, Divided Cell, Mg Anode

A repeat of the divided cell experiment using an Mg anode and benzyl bromide was performed with $^{13}CO_2$ with the goal of determining which side products incorporated CO_2. To save on -$^{13}CO_2$, the bubbling flow rate was reduced to 5 sccm. Overall, the same types of side products were found, but some of the relative ratios were different, so the goal of this experiment is just to identify which side products incorporate CO_2 into them. Below, evidence from GCMS and NMR is presented to affirm whether the side product incorporated CO_2.

Phenylacetic acid

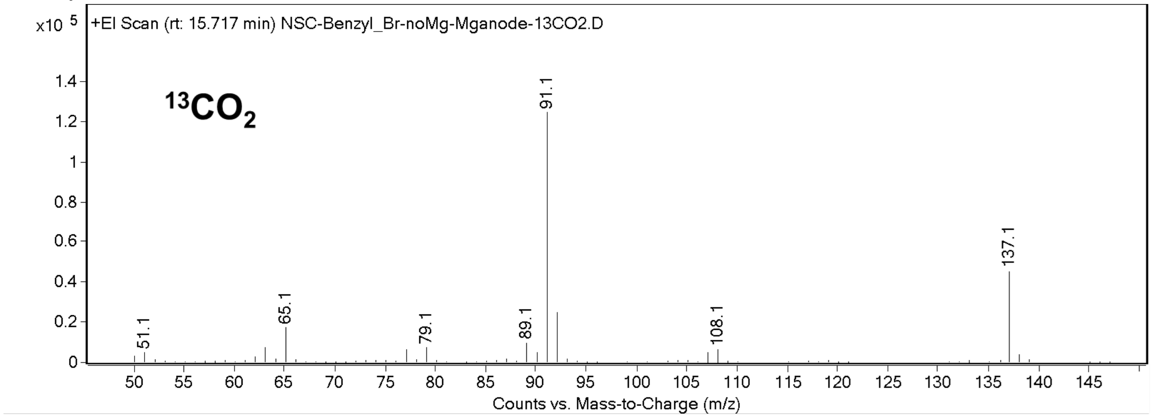

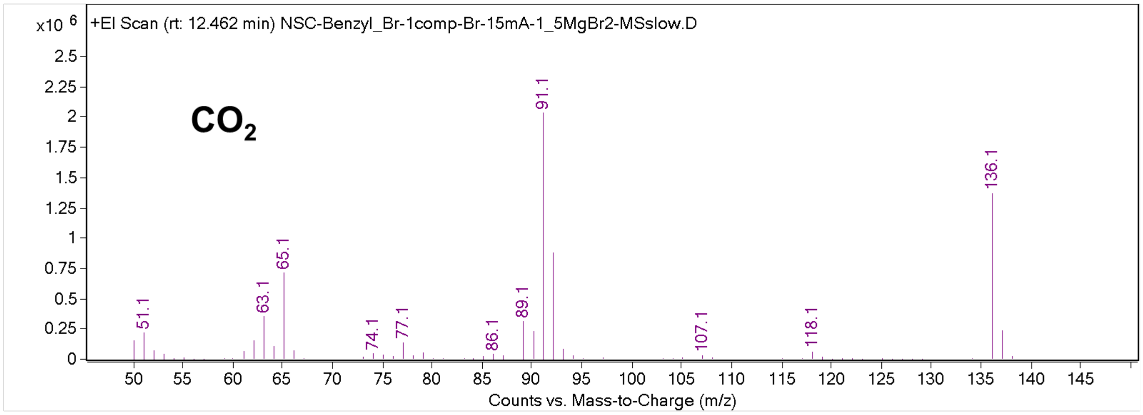

Mass spectrum: *m/z* at 137.1, the expected mass of $^{13}CO_2$ phenylacetic acid.
^{1}H NMR: Doublet at 3.66 and 5.15 ppm (normally a singlet).
^{13}C NMR: Enhanced peaks at 173 ppm.
^{1}H – ^{13}C HSQC: Coupling between 3.66 ppm and 173 ppm.

Benzyl-2-phenylacetate

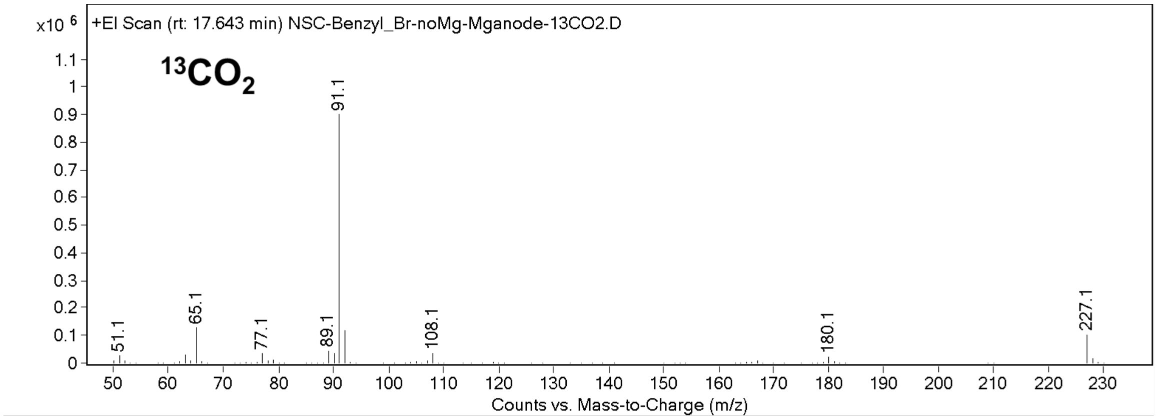

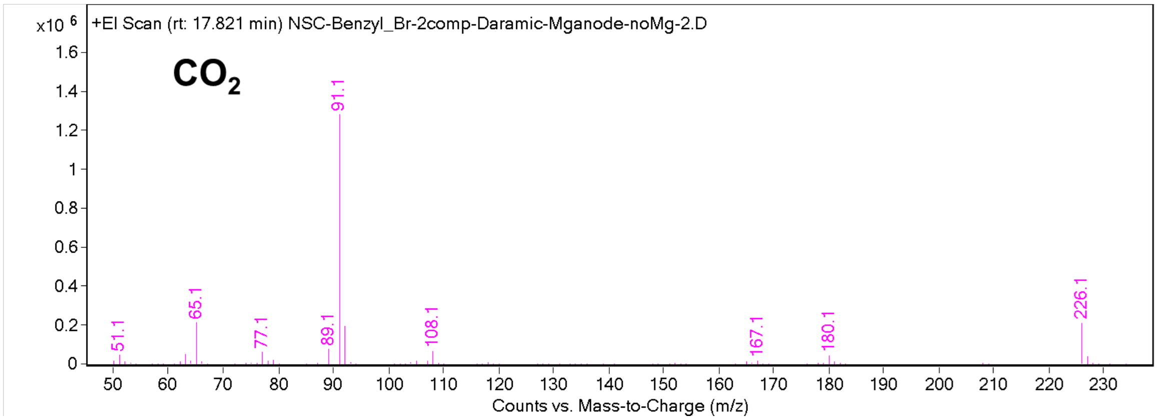

Mass spectrum: *m/z* at 227.1, the expected mass of $^{13}CO_2$ benzyl-2-phenylacetate.
^{1}H NMR: Doublets at 3.69 ppm (normally singlets).
^{13}C NMR: Enhanced peak at 171.4 ppm.
^{1}H – ^{13}C HSQC: Coupling between 3.69, 5.15 ppm and 171.7 ppm.

Dibenzyl Carbonate

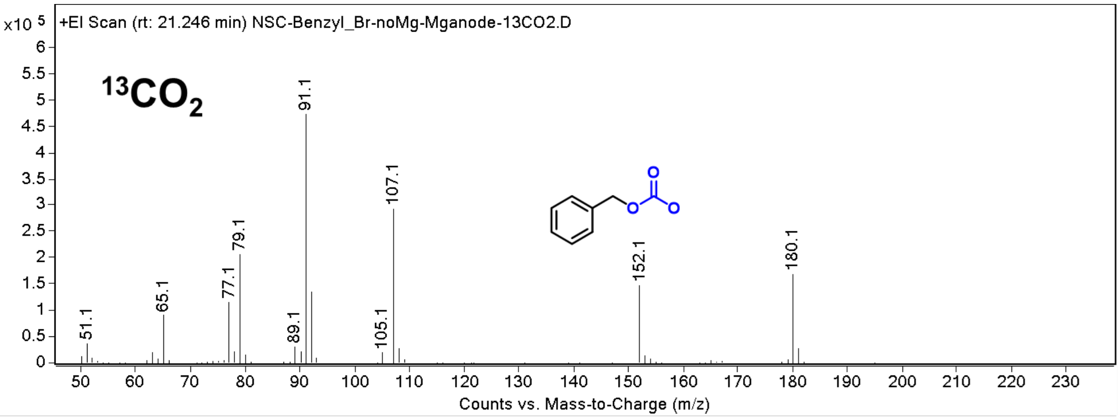

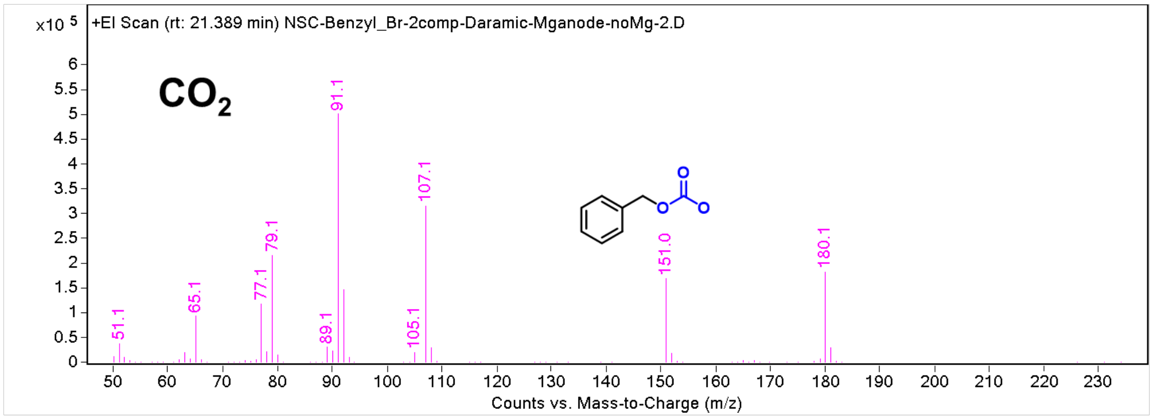

Mass spectrum: *m/z* at 152.1, the expected mass of a ^{13}C carbonate fragment.
^{1}H NMR: Doublet at 5.19 ppm (normally a singlet).
^{13}C NMR: Enhanced peak at 155.1 ppm.
^{1}H – ^{13}C HSQC: Coupling between 5.20 ppm and 155.3 ppm.

Benzyl formate

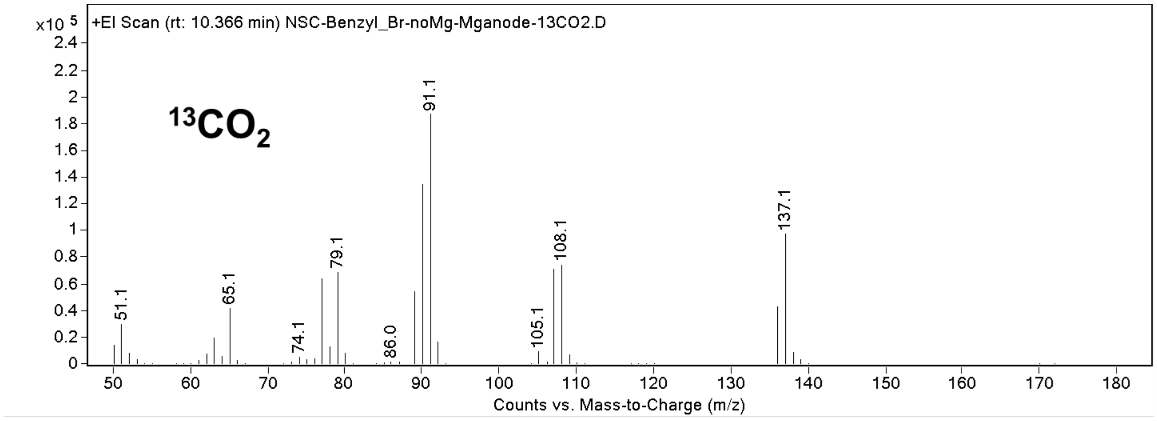

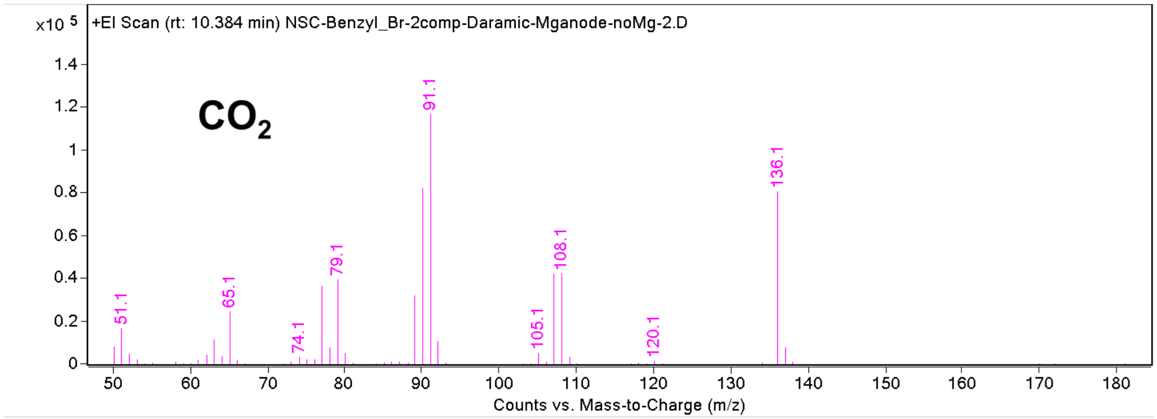

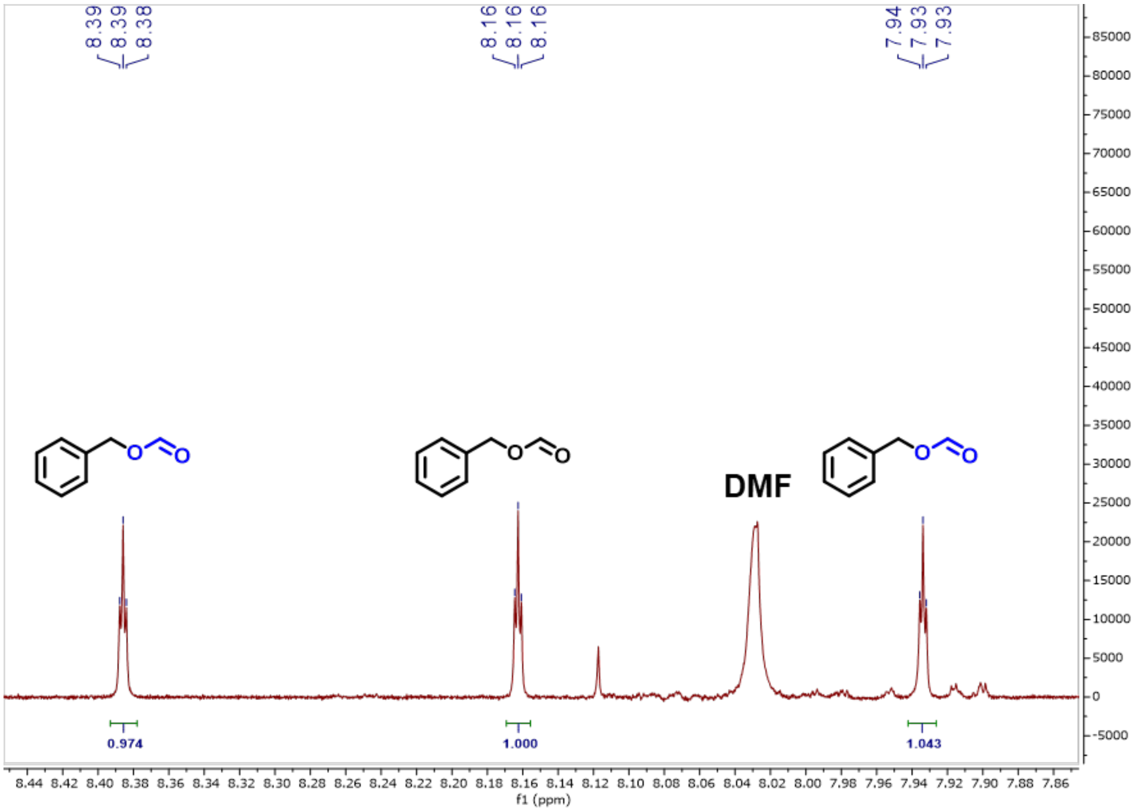

Mass spectrum: *m/z* at 137.1, the expected mass of benzyl formate with ^{13}C in the formyl group.
^{1}H NMR: Triplet of triplets at 8.16 ppm (J = 0.9, 115 Hz) (normally a triplet with J = 0.9 Hz), doublet of triplets at 5.23 ppm (J = 0.9, 2.8 Hz) (normally a doublet with J = 0.9 Hz).
^{13}C NMR: Enhanced peak at 160.8 ppm (formyl carbon).
^{1}H – ^{13}C HSQC: Coupling between 8.16 ppm and 160.7 ppm.

The presence of a noticeable peak at 136.1 *m/z* in the mass spectrum and the presence of triplets rather than doublets in the ^{1}H NMR suggests that there is only partial incorporation of $^{13}CO_2$ into the formyl group. Based on the ratio of peaks at 8.16 ppm, the ratio of ^{13}C:^{12}C is about 2:1. As discussed in sections below, benzyl formate can be detected without the passage of current or presence of CO_2, maybe as a result of the aqueous workup solvent. However, higher amounts have been detected from experiments passing current. A possibility exists that two pathways to benzyl formate exist.

Phenylacetaldehyde

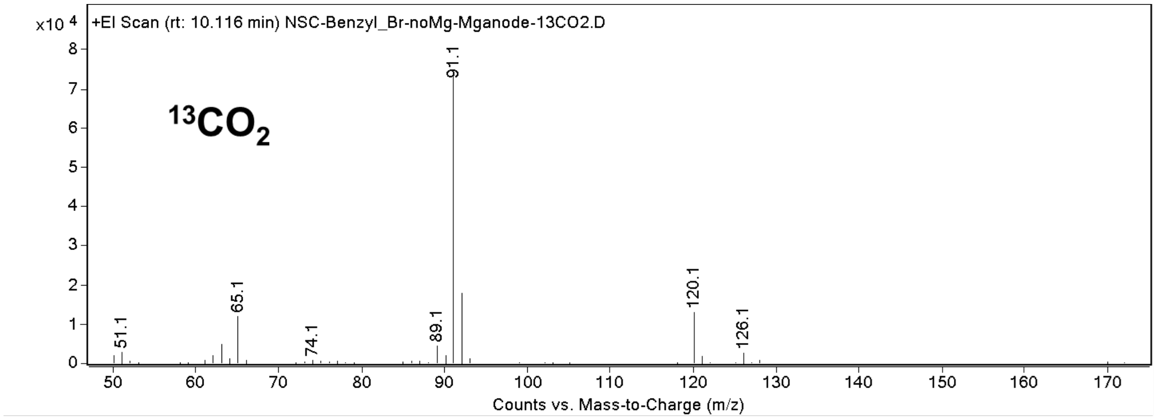

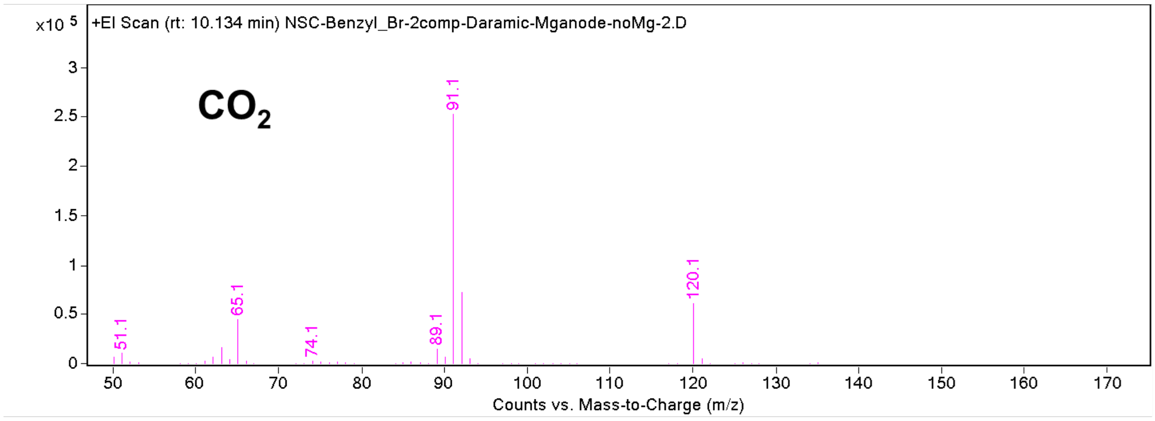

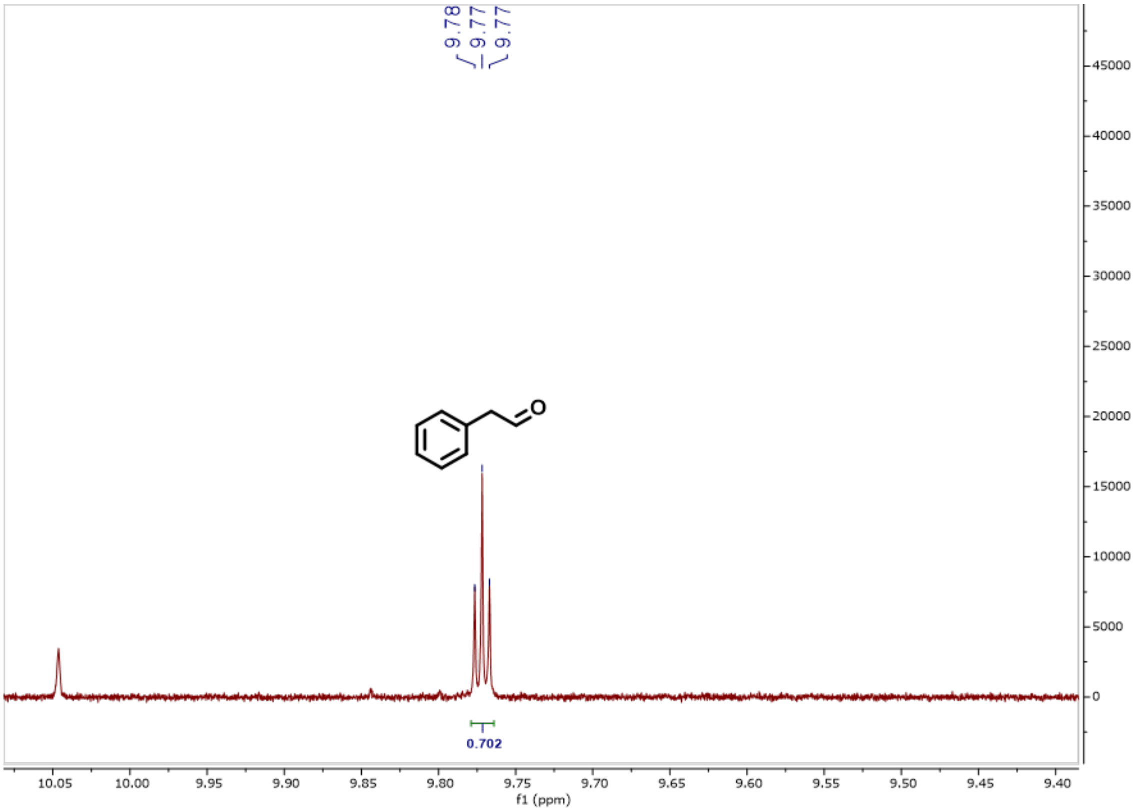

Mass spectrum: *m/z* at 120.1, the expected mass of phenylacetaldehyde with no ^{13}C substitution. ^{1}H NMR: Triplet at 9.77 ppm (J = 2.4 Hz), no additional splitting from ^{13}C. Doublet at 3.71 ppm overlapped by peak of benzyl-2-phenylacetate.

There appears to be no ^{13}C incorporation into phenylacetaldehyde, so it is a product by reaction with DMF. The analogous Beauveault achieves formylation of Grignard reagents by reaction with N,N-disubstituted formamides.

Benzyl N,N-dimethylcarbamate

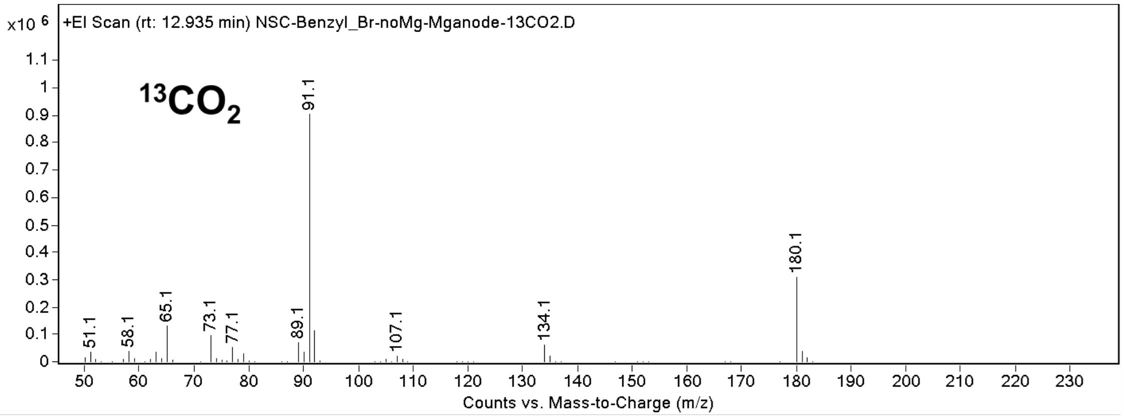

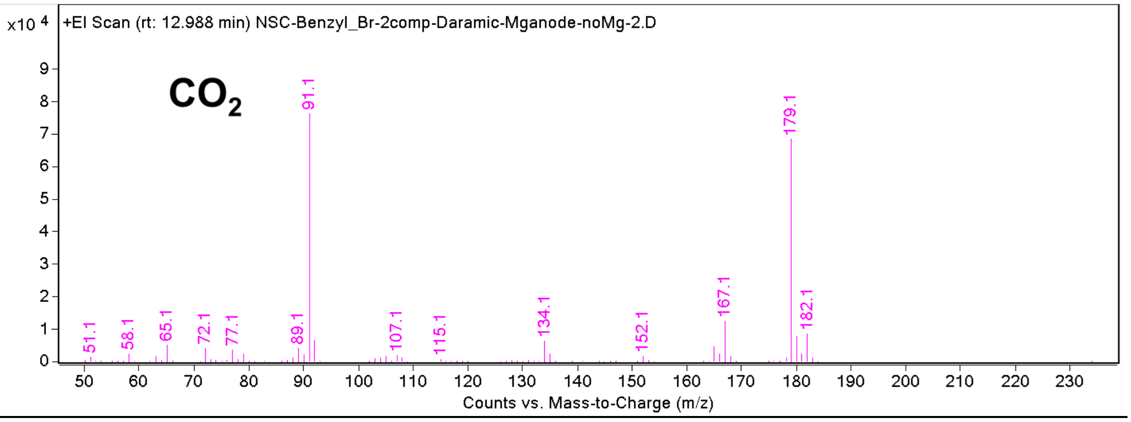

Mass spectrum: *m/z* at 180.1, the expected mass with ^{13}C in the carbamate group. There is some overlap with bibenzyl which has an *m/z* of 182.
^{1}H NMR: Doublet at 5.14 ppm (normally singlet)
^{13}C NMR: Enhanced peak at 157.5 ppm (formyl carbon).
^{1}H – ^{13}C HSQC: Coupling between 2.96 ppm and both 36.3 & 156.7 ppm. Coupling between 5.14 ppm and 156.7 ppm.

N,N-Dimethylbenzylamine

Based on control experiments described in subsequent sections, N,N-dimethylbenzylamine can be readily formed during workup if NaOH is added. In this $^{13}CO_2$ experiment, H_2O was used to generate the aqueous phase, and no N,N-dimethylbenzylamine was detected. In a similar experiment with N_2 purging, N,N-dimethylbenzylamine was detected with H_2O as the aqueous phase during workup, suggesting this compound could form, but overall, it is only a very minor side product under carboxylation conditions.

NMR Spectra

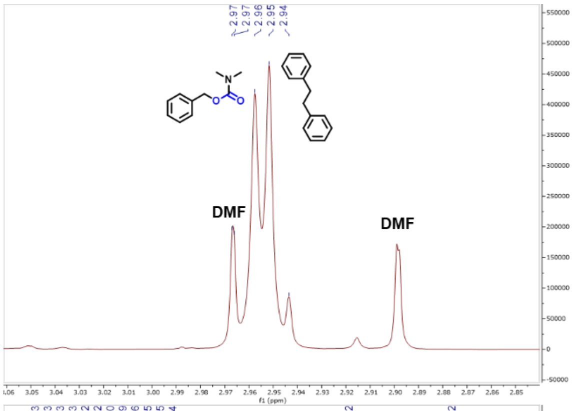
2.97
2.97
2.96
2.95
2.94
DMF
DMF
f1 (ppm)

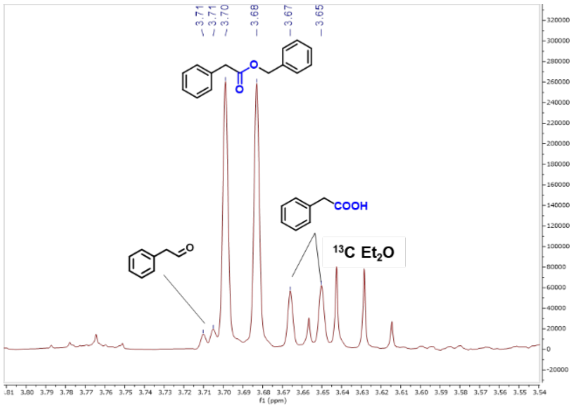
3.71
3.71
3.70
3.68
3.67
3.65
COOH
^{13}C Et$_2$O
f1 (ppm)

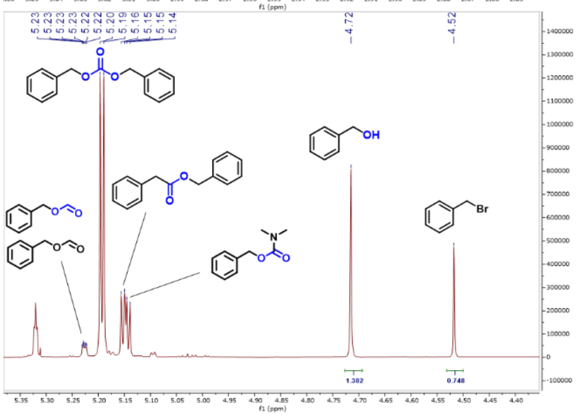
5.23
5.23
5.23
5.23
5.22
5.22
5.20
5.20
5.19
5.16
5.15
5.15
5.14
4.72
4.52
OH
Br
1.382
0.748
f1 (ppm)

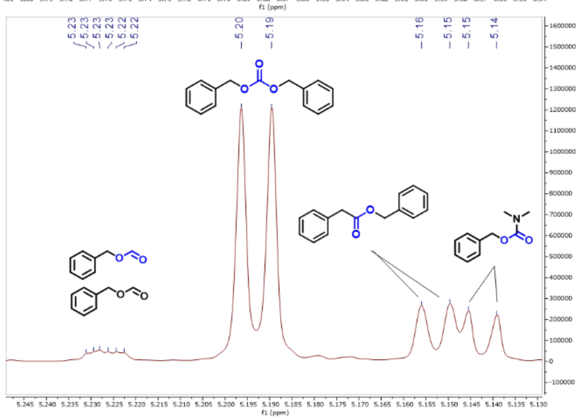
5.23
5.23
5.23
5.23
5.22
5.22
5.20
5.19
5.16
5.15
5.15
5.14
f1 (ppm)

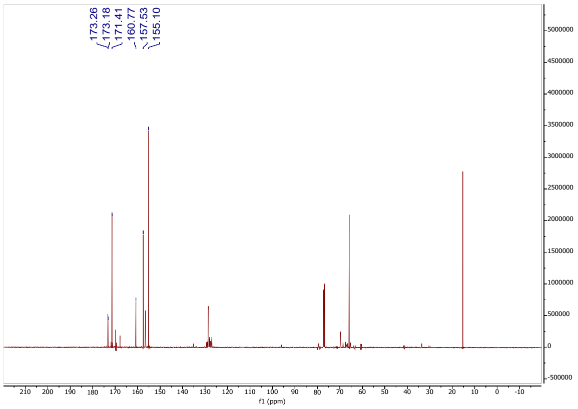
173.26
173.18
171.41
160.77
157.53
155.10
f1 (ppm)

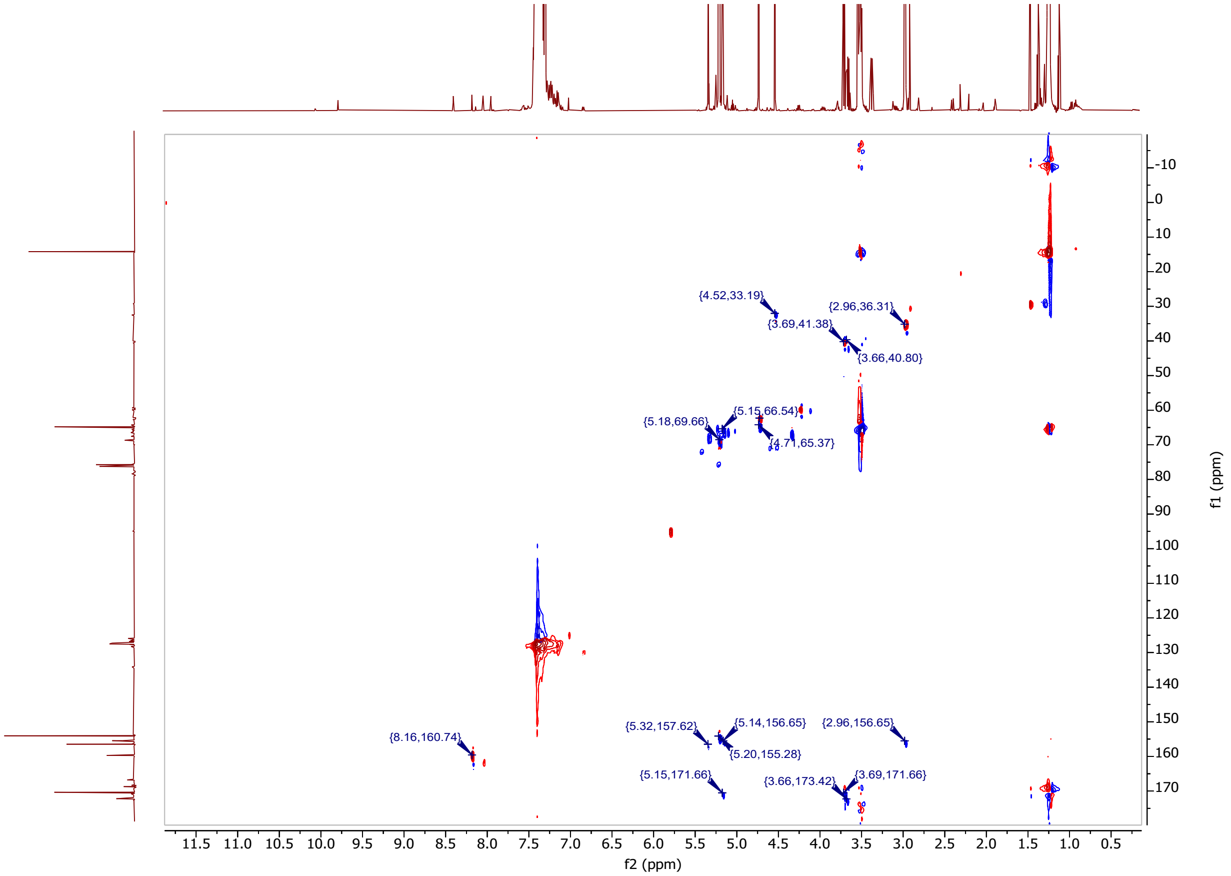

9.3 Deuterated Solvent Experiment

To test the hypothesis that the solvent is responsible for hydrogenation, carboxylation was performed in DMSO-d_6 with **1b** as the substrate to improve the ease of detection of the alkane side product. DMSO-d_6 was used as a cheaper alternative to DMF-d_7.

Reaction conditions: 0.1 M 1-bromo-3-phenylpropane (**1b**), 0.1 M TBA-Br, 0.1 M $MgBr_2$ (not all of it was soluble), 2.2 mL DMSO-d_6, undivided cell, Ag cathode, Pt anode, -20 mA/cm^2, 1 hr.

Workup: 3.5 mL 1 M HBr, 3x Et_2O extraction (2x 3.5 mL, 1x 1.75 mL), MilliQ wash on combined organics, dried organic layer with $MgSO_4$.

As shown below, the mass spectrum of the alkane shows deuterium incorporation at the tail end of the alkyl chain. The deuterium incorporation is also verified by the additional peak splitting in ^{1}H NMR and presence of a peak at 0.94 ppm in ^{2}H NMR.

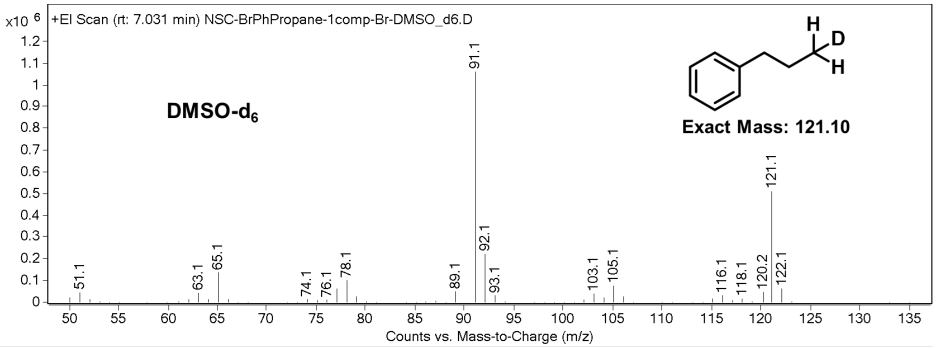
+EI Scan (rt: 7.031 min) NSC-BrPhPropane-1comp-Br-DMSO_d6.D
DMSO-d6
Exact Mass: 121.10
Counts vs. Mass-to-Charge (m/z)

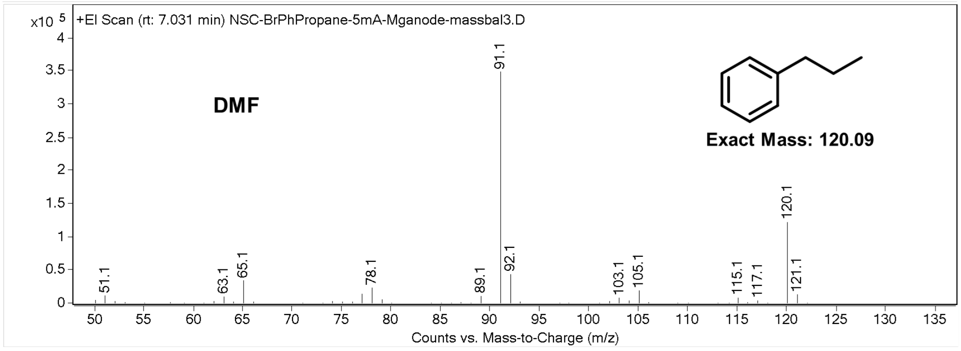
+EI Scan (rt: 7.031 min) NSC-BrPhPropane-5mA-Mganode-massbal3.D
DMF
Exact Mass: 120.09
Counts vs. Mass-to-Charge (m/z)

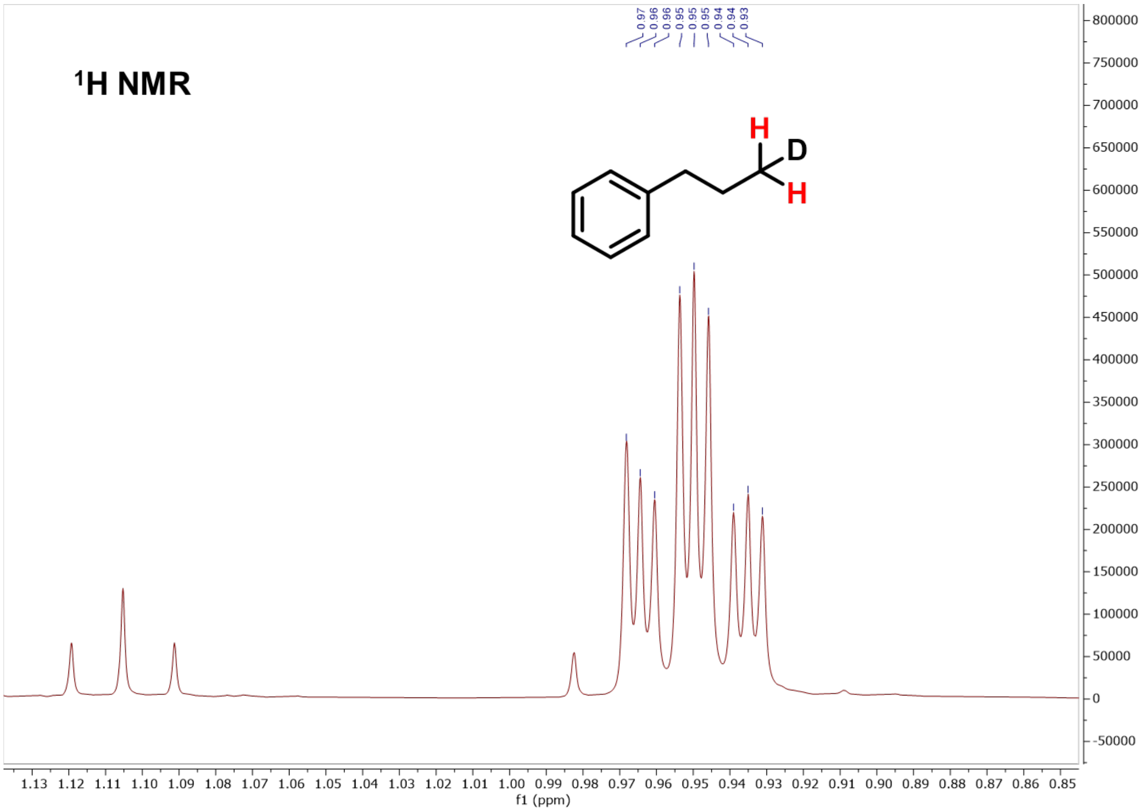

1H NMR
H
D
H
0.97
0.96
0.96
0.95
0.95
0.95
0.94
0.94
0.93
f1 (ppm)

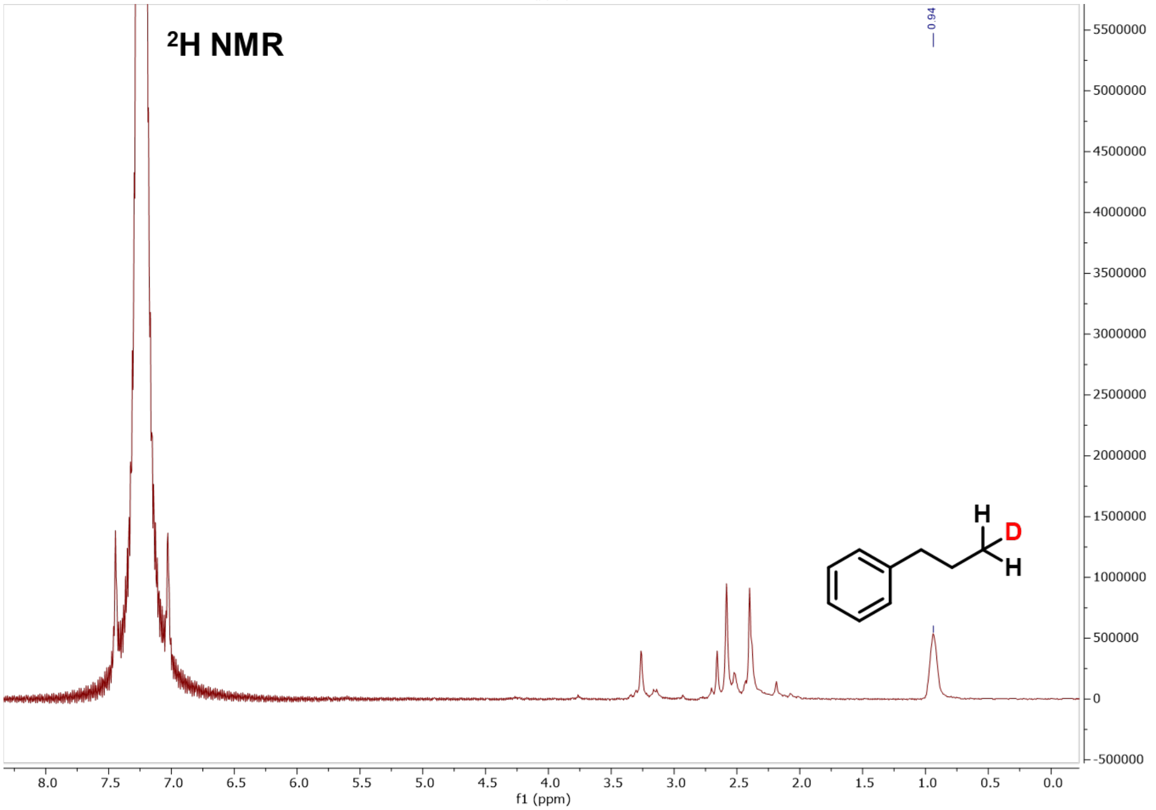

2H NMR
H
D
H
0.94
f1 (ppm)

9.4 Control Experiments without Current

A series of controls were performed to evaluate if the workup procedure could result in reactions between the substrate and solvent. Specifically, benzyl bromide was investigated since it is the most susceptible to side reactions. These experiments involved performing workup immediately on a 0.1 M benzyl bromide solution in 1 mL DMF using four different workup solvent combinations: (1) hexane, (2) MilliQ water + Et_2O, (3) 0.1 M HBr + Et_2O, (4) 1 M HBr + Et_2O. 1.75 mL of each solvent was added, and three organic layer extractions (3x 1.75 mL) were performed. The combined organic layers were washed with 1.75 mL MilliQ, and the sample was dried with $MgSO_4$. GCMS-FID was used to identify and qualitatively compare compounds. The small amount of BHT present in the Et_2O was used as a rough internal standard since roughly the same amount of Et_2O was used for all samples (except hexane). The results were also scaled by the volume of Et_2O used to enable comparison to experiments using different workup procedures.

Table S8. Relative amounts of compounds from various workup procedures on 1 mL solution of 0.1 M benzyl bromide in DMF. Values are derived from FID areas from GCMS-FID. Workups involving Et_2O used the BHT stabilizer as a rough internal standard, so all of the values are normalized to the BHT area and incorporate the Et_2O volume.

**Compound**	**Workup Solvents**			
	Hexane	MilliQ + Et_2O	0.1 M HBr + Et_2O	1 M HBr + Et_2O
Benzaldehyde	3.19×10^5	471.0	469.0	2256.8
Benzyl bromide	4.30×10^8	4.360×10^5	4.718×10^5	4.840×10^5
Benzyl formate	1.71E x 10^5	564.1	535.4	687.5
Formate/Bromide Ratio	3.42×10^{-4}	1.29×10^{-3}	1.13×10^{-3}	1.42×10^{-3}

The primary products detected aside from benzyl bromide were benzaldehyde, benzyl formate, toluene, and bibenzyl. The latter two were formed in small amounts and are known to form via electrochemical and homogeneous reactions already, so they are not discussed further. Benzaldehyde is an oxidation product of benzyl bromide, which could arise from exposure to oxygen over time. Benzyl formate is a potential nucleophilic side product. Based on the ratios above, benzyl formate seems to form in roughly equal amounts during aqueous and acidic workups, while the workup involving just hexane found significantly less. The water could be hydrolyzing the DMF to a small extent and reacting with the benzyl bromide. Benzyl formate has been detected by ^1H NMR, but it could also be an artifact from heating during GCMS-FID. Nonetheless, these values provide a baseline to confirm if electrolysis can enhance the production of benzyl formate.

Another series of controls were performed by bubbling either CO_2 or N_2 for over an hour into a 0.1 M benzyl bromide solution in DMF to see if gases and/or residence time would lead to observed side products. A variety of workup solvents were also used. Using NaOH as a workup solvent led to significant amounts of N,N-dimethylbenzylamine, on par with the amount of

benzyl bromide measured. NaOH also seemed to suppress the formation of benzyl formate. The presence of CO_2 did not lead to an increase in benzyl formate formation.

Table S9. Relative amounts of compounds from bubbling either CO_2 or N_2 into a 0.1 M benzyl bromide solution in DMF for several hours. Aqueous workup layers are indicated. Workup procedures for each gas are detailed below. Values are derived from GCMS-FID areas scaled to the amount of BHT and volume of Et_2O used.

Compound	Experiment			
	N_2 0.1 M HBr	CO_2 0.1 M HBr	N_2 0.5 M NaOH	CO_2 0.5 M NaOH
Benzaldehyde	198.0	432.1	601.1	-
Benzyl bromide	1.617×10^5	1.754×10^5	1.010×10^5	184.1
Benzyl formate	930.7	584.9	21.9	-
Dimethylbenzylamine	-	0	5.665×10^4	117.9
Formate/Bromide Ratio	5.76×10^{-3}	3.33×10^{-3}	2.17×10^{-4}	-

CO_2 workup: Three workups were done sequentially: (1) hexane (3x 1.75 mL), (2) 0.1 M HBr (1.75 mL) + Et_2O (2x 1.75 mL), (3) 0.5 M NaOH (1.75 mL) + Et_2O (2x 1.75 mL).
N_2 workup: Solution was divided roughly equally, and two parallel workups were done: (1) 0.1 M HBr (1.75 mL) + Et_2O (3x 1.75 mL), (2) 0.5 M NaOH (1.75 mL) + Et_2O (3x 1.75 mL).

To test what the effect of trace amounts of water would be, 0.1 M 1-bromo-3-phenylpropane was added to 0.1 M TBA-OH in DMF with methanol from the 1 M TBA-OH stock. Either CO_2 or N_2 was bubbled through the solution for a few hours, followed by extraction into diethyl ether. GCMS-FID was used to obtain qualitative information regarding the side products formed. For both experiments, the elimination product allyl benzene was formed in greater quantities than the alcohol; the elimination product was more prominent in the N_2 case due to CO_2 reacting with the hydroxide to form bicarbonate. At least in the case of 1-bromo-3-phenylpropane (benzyl bromide cannot form an elimination product), this experiment confirms that the reduction of trace amounts of water at the cathode to form hydroxide does not account for the observed alcohol.

9.5 Control Experiments Adding Substrate After Passing Current

A series of experiments were performed by passing current under CO_2. Once the current was stopped, benzyl bromide was added and allowed to mix for 1 – 1.5 hr. These controls were aimed at seeing which nucleophilic products would form without electrochemical reduction of the substrate. Experiments with CO_2 bubbling throughout revealed that benzyl alcohol and dibenzyl carbonate were detected, with no phenylacetic acid or benzyl phenylacetate detected. Moreover, if the bubbling was switched to N_2 after stopping current but before adding benzyl bromide, dibenzyl carbonate was not detected, and the amount of alcohol decreased by at least an order of magnitude. These experiments confirm the alcohol and carbonate side products can originate from a nucleophilic attack of carbonate anions on the substrate. More benzyl formate and benzaldehyde were also detected during the experiments flowing CO_2, suggesting these products could be formed by electrolysis.

9.6 Carbon monoxide as an Intermediate in Carboxylation

A possibility exists that carbon monoxide (CO) could be an intermediate for carboxylation. CO can be used as a source of carboxylate groups in the Koch reaction and carboxylation reactions.

A control experiment to assess whether CO in the bulk electrolyte could react with the organic halide substrate to form the carboxylate was performed. A third species is needed to supply another oxygen to form the carboxylate group such as CO_3^{2-} or DMF:

$$R - X + CO + CO_3^{2-} \rightarrow R - COO^- + CO_2 + X^-$$

To maximize the possibility of forming a carboxylate from CO, carbonate anions were first produced in the electrolyte, after which the current was stopped and the substrate and CO were added.

1) In an undivided cell with a Ag cathode, Pt anode, 0.1 M TBA-Br in 2.2 mL DMF electrolyte, and 20 sccm CO_2, -10 mA/cm^2 was applied for 30 min to generate CO_3^{2-} species.
2) The current was stopped, then 0.1 M 1-bromo-3-phenylpropane (**1b**) was added to the cell.
3) The gas flow was switched to 10 sccm CO for 30 min without current.
4) The gas flow was switched to 20 sccm CO_2 for 10 min without current as a safety precaution to flush out CO before disassembling the cell.

After this experiment, workup was performed to isolate the products. Both ^{1}H NMR and GCMS-FID analyses failed to identify any carboxylate products (either the acid or the ester). This experiment confirms that CO in the bulk electrolyte is not responsible for carboxylation.

9.7 Lack of Interaction Between $MgBr_2$ and R-Br

We performed ^{1}H and ^{13}C NMR on 50 mM 1-bromo-3-phenylpropane in DMSO-d_6 with and without 50 mM $MgBr_2$ to see if there is any interaction between Mg^{2+} and the alkyl bromide. After normalizing to TMS (^{1}H, 0 ppm) and DMSO-d_6 (^{13}C, 39.52 ppm), no significant shifts were observed in either the proton or carbon signals. These data suggest $MgBr_2$ does not interact or activate the substrate for carboxylation.

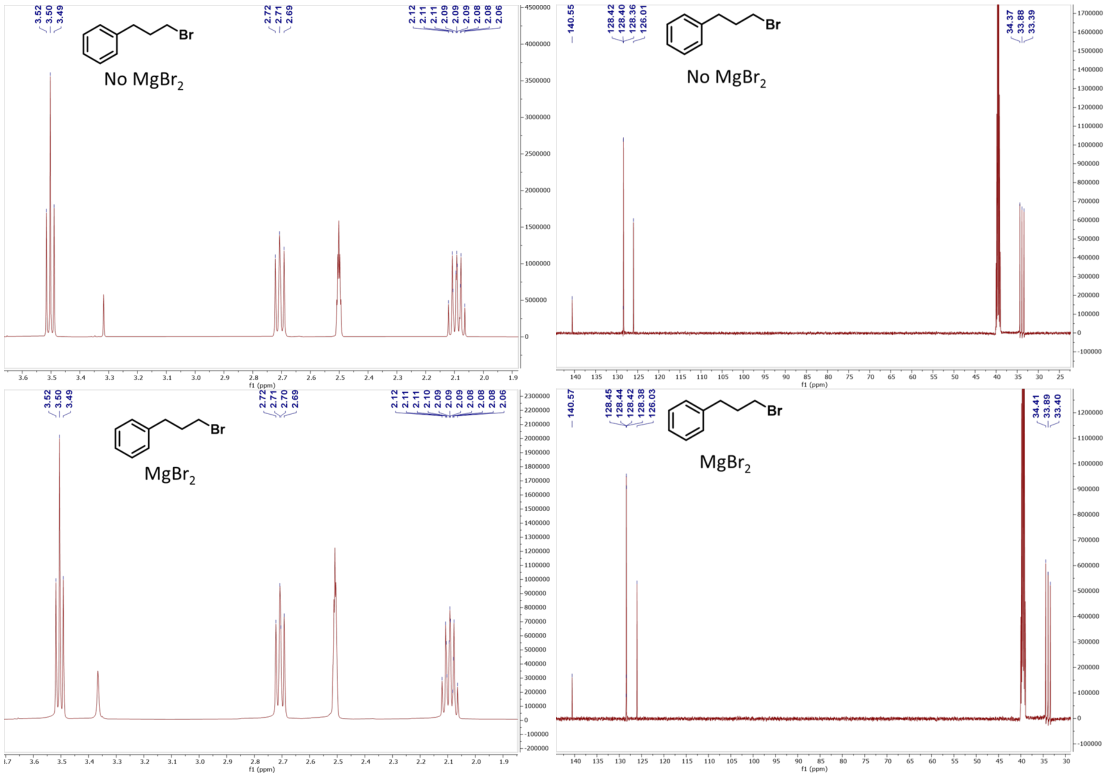

10 Grignard Carboxylation Reactions

A few reactions of a Grignard reagent with CO_2 were performed to assess how well the electrochemical protocol developed in this work can tolerate functional groups that are known to react with Grignard reagents.

<u>Bromobenzene</u>

The purpose of this experiment was to establish a reasonable protocol for Grignard carboxylation using bromobenzene as a model substrate.

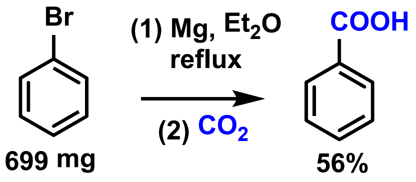

A 25 mL Schlenk flask and stir bar were dried at 80 °C. Magnesium turnings (129 mg) was added to the flask, which was subsequently cycled onto a Schlenk line. Et_2O (6 mL, distilled to remove BHT, dried over molecular sieves) was added to the flask and stirred. Bromobenzene (699 mg) in Et_2O (2 mL) was slowly added to the flask, during which the heat was increased to gently reflux the Et_2O. After ~ 1.5 hr, the solution had become a cloudy brown. At this stage, the flask was closed and removed from the Schlenk line. CO_2 was purged at a rate of 200 sccm through a long needle into the solution for 20 min. The product was purified by acidification with 4 M HCl (being careful about the H_2 evolution from Mg) and extracted 3x with Et_2O. The combined organic layers were dried with $MgSO_4$, filtered, and rotary evaporated. A yield of 56%

of benzoic acid was obtained via ^{1}H NMR with 29.4 mg 1,3,5-trimethoxybenzene as an internal standard.

1-Bromo-5-chloropentane

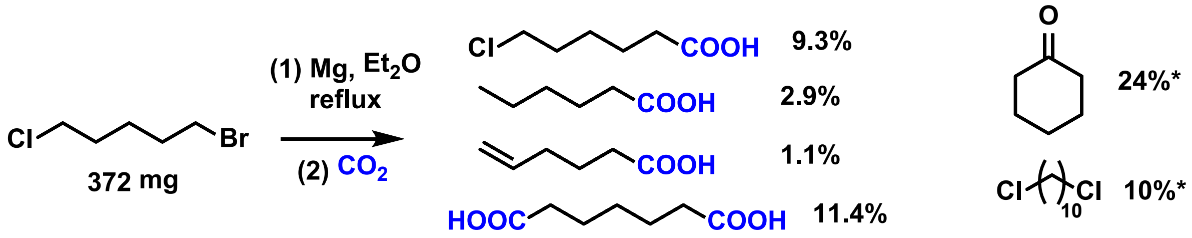

A 25 mL Schlenk flask and stir bar were dried at 80 °C. Magnesium turnings (129 mg) was added to the flask, which was subsequently cycled onto a Schlenk line. Et_2O (6 mL, distilled to remove BHT, dried over molecular sieves) was added to the flask and stirred. 1-Bromo-5-chloropentane (372 mg) in Et_2O (2 mL) was slowly added to the flask, during which the heat was increased to gently reflux the Et_2O. Because it appeared that the reaction was slow to start, a small amount of iodine was added. After ~ 2 hr at reflux, the solution was slightly cloudy and the heating was removed. At this stage, the flask was closed and removed from the Schlenk line. CO_2 was purged at a rate of 200 sccm through a long needle into the solution for 20 min. The product was purified by acidification with 4 M HCl (being careful about the H_2 evolution from Mg) and extracted 3x with Et_2O. The combined organic layers were dried with $MgSO_4$, filtered, and rotary evaporated. A yield of 9.3% of 6-chlorohexanoic acid was obtained via ^{1}H NMR with 13 mg ethylene carbonate as an internal standard. Notably, many additional carboxylic acids were formed, and other side products not observed from electrochemical carboxylation were made during during the Grignard formation reaction (yields estimated via GCMS-FID, indicated by an asterisk). Cyclopentane, a result of ring closure, was unable to be detected due to its low boiling point. This experiment revealed that the electrochemical carboxylation is much more tolerant to alkyl chlorides than Grignard carboxylation.

Methyl (4-bromomethyl)benzoate

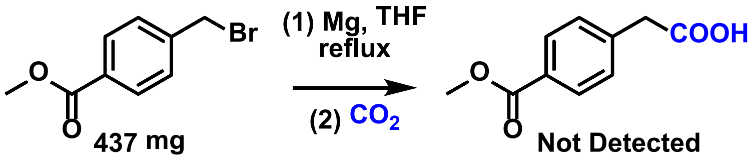

A 25 mL Schlenk flask, stir bar, and 108 mg magnesium turnings were air dried at 80 °C. The flask was then cycled onto the Schlenk line while sitting in a water bath at 75 °C. Dry THF (6 mL) was added to the flask via syringe while stirring. Methyl 4-(bromomethyl)benzoate (437 mg) was dissolved in 2 mL THF along with a small amount of 1,2-dibromoethane (for Mg activation) and added all at once to the flask; previous experiments had indicated this substrate was difficult to convert into a Grignard reagent, so adding all the reagent at once would not lead to overheating. The solution was stirred under reflux for 5 hr, after which it turned a dark green color. CO_2 was bubbled into the solution at ambient conditions at 30 sccm for 30 min. Workup

proceeded by removing the THF from the flask into a collection tube, washing the flask with 2.5 mL 1.5 M pH 2 phosphate buffer (and adding ~ 1 mL of this buffer to the collection tube), and then washing the Schlenk flask with 3.5 mL Et_2O. All washes were combined with the THF, and the organic layer was extracted. Two more Et_2O extractions were performed (1.75 mL). The combined organic layers were dried with $MgSO_4$, filtered, and rotavapped at room temperature and then 45 °C. The residue was concentrated under vacuum. The ^{1}H and ^{13}C NMR of this crude product showed no traces of carboxylic acid. The major products appeared to be the hydrogenated substrate as well as some polymeric material appearing as broad peaks in the ^{1}H NMR spectrum. Additionally, the Schlenk flask and Mg turnings were coated with an insoluble material after the reaction.

11 Experimental Details and Characterization Data of Products

This section contains the synthetic conditions and product characterization data for all carboxylation reactions, separated according to whether $MgBr_2$, a magnesium anode, or no magnesium source was used. The cathode area in all cases is 1 cm^2, so the reported currents also correspond to their current densities.

11.1 Notes about Impurities and Internal Standards in NMR Spectra

In many instances, residual solvents (Et_2O, EtOAc, DMF) appear in NMR spectra due to incomplete or lack of vacuum drying. Internal standards are also present to perform accurate quantification. To obtain accurate internal standard masses below 10 mg, an amount greater than 20 mg was weighed followed by a weighing of deuterated solvent. This solution was then divided approximately evenly between several NMR samples, weighing each transfer. The amount of standard in each sample could then be estimated with the mass fraction of the solution added and the initial mass of the standard with an error of about 1%.

Below is a list of these compounds and their associated chemical shifts. These shifts are not explicitly indicated on spectra. In most cases, the presence of these peaks does not interfere with identification or quantification of the product of interest.

- 1,3,5-Trimethoxybenzene (internal standard)
  ^{1}H NMR (500 MHz, $CDCl_3$) δ 6.09 (s, 3H), 3.76 (s, 9H)
  ^{13}C NMR (126 MHz, $CDCl_3$) δ 161.6, 93.1, 55.5
- Ethylene carbonate (internal standard)
  ^{1}H NMR (500 MHz, $CDCl_3$) δ 4.47 (s, 4H)
  ^{13}C NMR (126 MHz, $CDCl_3$) δ 155.6, 64.7
- Diethyl ether (Et_2O, workup solvent)
  ^{1}H NMR (500 MHz, $CDCl_3$) δ 3.49 (q, J = 7.03 Hz, 4H), 1.20 (t, J = 7.01 Hz, 6H).
  ^{13}C NMR (126 MHz, $CDCl_3$) δ 66.0, 15.3
- Ethyl acetate (EtOAc, workup solvent)
  ^{1}H NMR (500 MHz, $CDCl_3$) δ 4.12 (q, J = 7.14 Hz, 2H), 2.04 (s, 3H), 1.25 (t, J = 7.14 Hz, 3H).
  ^{13}C NMR (126 MHz, $CDCl_3$) δ 171.6, 60.6, 21.2, 14.3

- N,N-Dimethylformamide (DMF, carboxylation solvent)
  ^{1}H NMR (500 MHz, $CDCl_3$) δ 8.00 (s, 1H), 2.95 (s, 3H), 2.88 (s, 3H)
- Acetic acid (from EtOAc hydrolysis during acidic workup steps)
  ^{1}H NMR (500 MHz, $CDCl_3$) δ 2.08 (s, 3H)
  ^{13}C NMR (126 MHz, $CDCl_3$) δ 177.7, 20.9
- Butylated hydroxyl toluene (BHT, stabilizer in Et_2O)
  ^{1}H NMR (500 MHz, $CDCl_3$) δ 6.98 (s, 2H), 2.27 (s, 3H), 1.43 (s, 18H)
- N-Methyl pyrrolidinone (contamination from vacuum oven)
  ^{1}H NMR (500 MHz, $CDCl_3$) δ 3.45 – 3.40 (m, 2H), 2.90 (s, 3H), 2.53 – 2.44 (m, 2H), 2.09 – 2.03 (m, 2H).

11.2 Carboxylation Using $MgBr_2$ and a Non-Sacrificial Anode

4-Phenylbutyric acid (1a)[11]

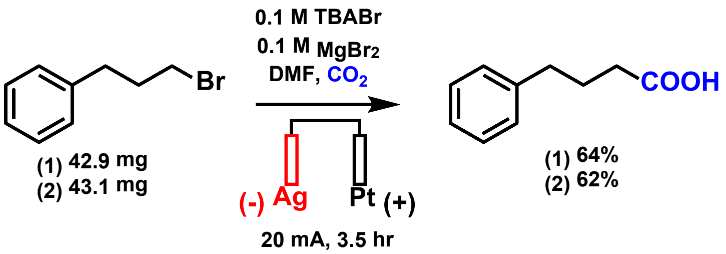

<u>Experiment (1)</u>: Following **Procedure B**, 0.22 mmol TBABr, 0.22 mmol $MgBr_2$, 2.2 mL DMF, and 42.9 mg 1-bromo-3-phenylpropane were combined and subjected to 20 mA for 3.5 hr under 20 sccm CO_2. The product was purified according to **Procedure B** to give **1a** in 64% yield by ^{1}H NMR with 6.2 mg of 1,3,5-trimethoxybenzene as the internal standard.

^{1}H NMR (500 MHz, $CDCl_3$) δ 11.35 (br, s), 7.31 – 7.25 (m, 2H), 7.21 – 7.14 (m, 3H), 2.67 (t, J = 7.6 Hz, 2H), 2.37 (t, J = 7.45 Hz, 2H), 1.97 (dt, J = 7.67, 7.00 Hz, 2H).

^{13}C NMR (126 MHz, $CDCl_3$) δ 180.13, 141.29, 128.61, 128.55, 126.18, 35.11, 33.45, 26.32.

MS (*m/z*) 164 (M^+, 46.9%), 146 (30.0), 105 (61.6), 104 (99.9), 91 (78.5), 65 (28.2)

<u>Experiment (2)</u>: A repeat of Experiment (1). Following **Procedure B**, 0.22 mmol TBABr, 0.22 mmol $MgBr_2$, 2.2 mL DMF, and 43.1 mg 1-bromo-3-phenylpropane were combined and subjected to 20 mA for 3.5 hr under 20 sccm CO_2. The product was purified according to **Procedure B** to give **1a** in 62% yield by ^{1}H NMR with 6.3 mg of ethylene carbonate as the internal standard.

^{1}H NMR (500 MHz, $CDCl_3$) δ 10.50 (br, s), 7.31 – 7.26 (m, 2H), 7.21 – 7.16 (m, 3H), 2.71 – 2.64 (m, 2H), 2.38 (t, J = 7.44 Hz, 2H), 2.03 – 1.92 (m, 2H).

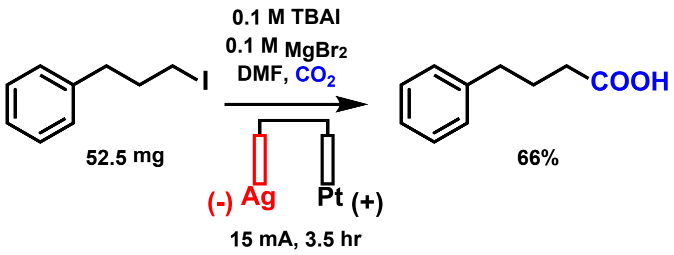

Following **Procedure B**, 0.22 mmol TBAI, 0.22 mmol $MgBr_2$, 2.2 mL DMF, and 52.5 mg 1-iodo-3-phenylpropane were combined and subjected to 15 mA for 3.5 hr under 20 sccm CO_2. The product was purified according to **Procedure B** to give **1a** in 66% yield by ^{1}H NMR with 6.4 mg of ethylene carbonate as the internal standard. A synthesis performed at 20 mA resulted in a lower yield of 46%.

^{1}H NMR (500 MHz, $CDCl_3$) δ 10.31 (br, s), 7.32 – 7.25 (m, 2H), 7.22 – 7.15 (m, 3H), 2.69 – 2.66 (m, 2H), 2.37 (t, *J* = 7.44 Hz, 2H), 2.03 – 1.89 (m, 2H).
^{13}C NMR (126 MHz, $CDCl_3$) δ 180.00, 141.30, 128.58, 128.52, 126.15, 35.09, 33.43, 26.31.

3-Phenylpropanoic acid (2a)[12]

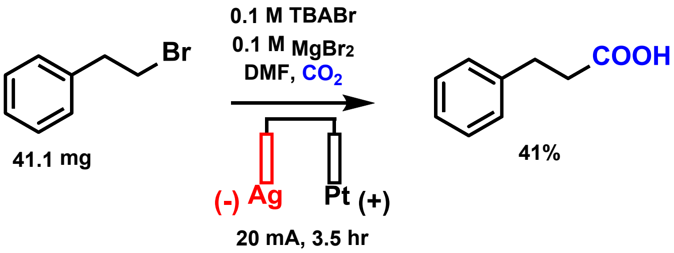

Following **Procedure B**, 0.22 mmol TBABr, 0.22 mmol $MgBr_2$, 2.2 mL DMF, and 41.1 mg (2-bromoethyl)benzene were combined and subjected to 20 mA for 3.5 hr under 20 sccm CO_2. The product was purified according to **Procedure B** to give **2a** in 41% yield by ^{1}H NMR with 4.8 mg of 1,3,5-trimethoxybenzene as the internal standard.

^{1}H NMR (500 MHz, $CDCl_3$) δ 11.08 (br, s), 7.32 – 7.26 (m, 2H), 7.23 – 7.18 (m, 3H), 2.96 (t, *J* = 7.82 Hz, 2H), 2.78 – 2.62 (m, 2H).

^{13}C NMR (126 MHz, $CDCl_3$) δ 179.35, 140.24, 128.70, 128.39, 126.52, 35.72, 30.69.

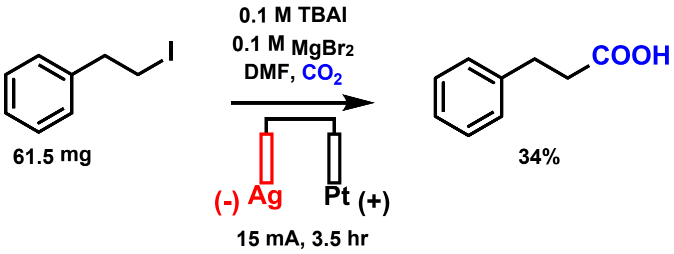

Following **Procedure B**, 0.22 mmol TBAI, 0.22 mmol $MgBr_2$, 2.2 mL DMF, and 61.5 mg (2-iodoethyl)benzene were combined and subjected to 15 mA for 3.5 hr under 20 sccm CO_2. The

product was purified according to **Procedure B** to give **2a** in 34% yield by ^{1}H NMR with 7.3 mg of ethylene carbonate as the internal standard.

^{1}H NMR (500 MHz, $CDCl_3$) δ 9.82 (br, s), 7.34 – 7.26 (m, 2H), 7.24 – 7.18 (m, 3H), 2.96 (t, *J* = 7.79 Hz, 2H), 2.70 – 2.67 (m, 2H).

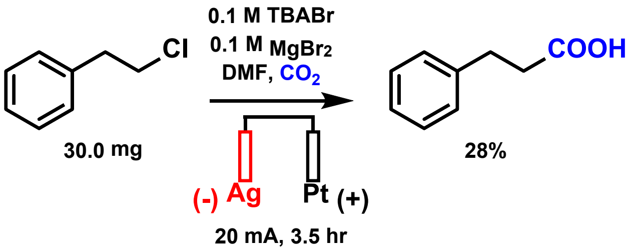

Following **Procedure B**, 0.22 mmol TBABr, 0.22 mmol $MgBr_2$, 2.2 mL DMF, and 30.0 mg (2-chloroethyl)benzene were combined and subjected to 20 mA for 3.5 hr under 20 sccm CO_2. The product was purified according to **Procedure B** to give **2a** in 28% yield by ^{1}H NMR with 8.6 mg ethylene carbonate as the internal standard.

^{1}H NMR (500 MHz, $CDCl_3$) δ 8.16 (br, s), 7.32 – 7.27 (m, 2H), 7.23 – 7.19 (m, 3H), 2.96 (t, *J* = 7.80 Hz, 2H), 2.77 – 2.64 (m, 2H).

^{13}C NMR (126 MHz, $CDCl_3$) δ 178.80, 140.23, 128.68, 128.38, 126.50, 35.63, 30.68.

4-(Benzyloxy)butanoic acid (3a)[13]

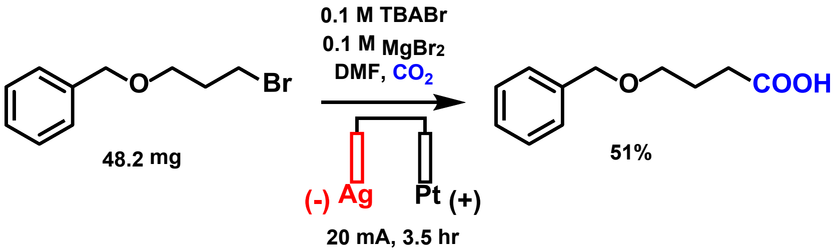

Following **Procedure B**, 0.22 mmol TBABr, 0.22 mmol $MgBr_2$, 2.2 mL DMF, and 48.2 mg benzyl 3-bromopropyl ether were combined and subjected to 20 mA for 3.5 hr under 20 sccm CO_2. The product was purified according to **Procedure B** to give **3a** in 51% yield by ^{1}H NMR with 6.2 mg of 1,3,5-trimethoxybenzene as the internal standard.

^{1}H NMR (500 MHz, $CDCl_3$) 10.87 (br, s), δ 7.37 – 7.25 (m, 5H), 4.50 (s, 2H), 3.53 (t, *J* = 6.11 Hz, 2H), 2.48 (t, *J* = 7.33 Hz, 2H), 1.94 (dt, *J* = 7.30, 6.09 Hz, 2H).

^{13}C NMR (126 MHz, $CDCl_3$) δ 179.67, 138.32, 128.51, 127.75, 73.05, 69.12, 31.07, 24.88.

6-Chlorohexanoic acid (4a) and side products

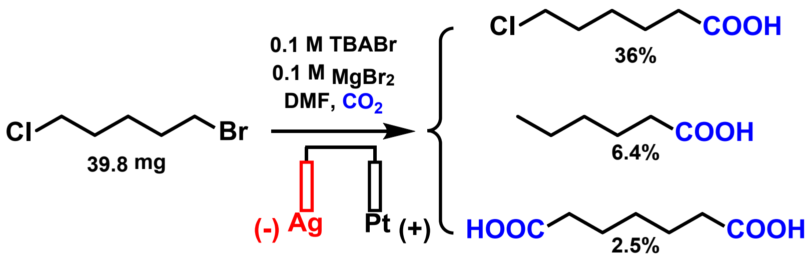

Following **Procedure B**, 0.22 mmol TBABr, 0.22 mmol $MgBr_2$, 2.2 mL DMF, and 39.8 mg 1-bromo-5-chloropentane were combined and subjected to 20 mA for 3.5 hr under 20 sccm CO_2. The product was purified according to **Procedure B** to give **4a** in 36% yield by ^{1}H NMR with 6.2 mg of ethylene carbonate as the internal standard. Additionally, the side products hexanoic acid and 1,7-heptanedioic acid were obtained with yields of 6.4% and 2.5% respectively. A full deconvolution of the spectra for assignments and quantification is provided with the spectra.

Characterization data for 6-chlorohexanoic acid (**4a**)[14]

^{1}H NMR (500 MHz, $CDCl_3$) δ 9.90 (br, s), 3.54 (t, *J* = 6.63 Hz, 2H), 2.38 (t, *J* = 7.42 Hz, 2H), 1.88 – 1.75 (m, 2H), 1.74 – 1.62 (m, 2H), 1.56 – 1.46 (m, 2H).

The triplet at 2.38 and multiplet at 1.74 – 1.62 are overlapped by hexanoic acid and 1,7-heptanedioic acid.

^{13}C NMR (126 MHz, $CDCl_3$) δ 179.82, 44.84, 33.93, 32.31, 26.41, 24.02.

MS (converted to 5-hexenoic acid on column) (*m/z*) 114 (M^+, 3.2%), 87 (21.7), 73 (54.8), 68 (21.6), 60 (99.9), 55 (42.3)

Characterization data for hexanoic acid[13]

^{1}H NMR (500 MHz, $CDCl_3$) δ 9.90 (br, s), 2.35 (t, 2H) 1.74 – 1.62 (m, 2H), 1.36 – 1.29 (m, 4H), 0.92 – 0.89 (m, 3H)

Triplet at 2.35 and multiplet at 1.74 – 1.62 are overlapped by **4a** and 1,7-heptanedioic acid.

^{13}C NMR (126 MHz, $CDCl_3$) δ 180.35, 34.13, 31.30, 24.46, 22.40, 13.98

MS (*m/z*) 87 (38.8%), 73 (78.1), 61 (18.4), 60 (99.9), 55 (27.6)

Could not observe M^+ peak, although it is also missing from NIST reference spectrum.

Characterization data for 1,7-heptanedioic acid[15]

^{1}H NMR (500 MHz, $CDCl_3$) δ 9.90 (br, s), 2.36 (4H) 1.74 – 1.62 (m, 4H), 1.44 – 1.38 (m, 2H)

Triplet at 2.35 and multiplet at 1.74 – 1.62 are overlapped by **4a** and hexanoic acid.

^{13}C NMR (126 MHz, $CDCl_3$) δ 179.92, 33.88, 28.48, 24.35

MS (*m/z*) 114 (82.0%), 101 (55.6), 83 (73.4), 60 (67.9), 55 (99.9)

Could not observe M^+ peak, although it is also missing from NIST reference spectrum.

Pentanoic Acid (16a)[16]

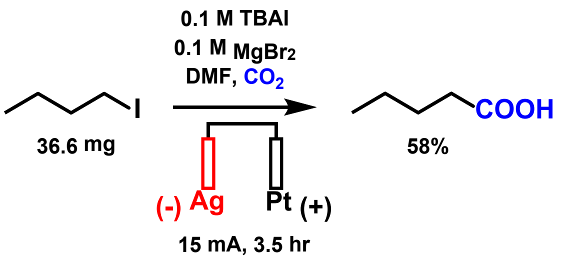

Following **Procedure B**, 0.22 mmol TBAI, 0.22 mmol $MgBr_2$, 2.2 mL DMF, and 36.6 mg 1-iodobutane were combined and subjected to 15 mA for 3.5 hr under 20 sccm CO_2. The product was purified according to **Procedure B** with two modifications. (1) Before rotary evaporating the EtOAc, 1.75 mL of 1 M $NaHCO_3$ was added to convert the acid to basic form to minimize evaporation. The remaining 1.75 mL 1 M $NaHCO_3$ was added after rotary evaporation. (2) The last vacuum drying step was avoided to minimize the risk of product loss. This afforded **16a** in 58% yield by ^{1}H NMR with 4.7 mg 1,3,5-trimethoxybenzene as the internal standard.

^{1}H NMR (500 MHz, $CDCl_3$) δ 2.36 (t, J = 7.54 Hz, 2H), 1.63 (tt, J = 7.69, 6.49 Hz, 2H), 1.42 – 1.33 (m, 2H), 0.93 (t, J = 7.37 Hz, 3H).

^{13}C NMR (126 MHz, $CDCl_3$) δ 179.66, 33.88, 26.89, 22.30, 13.78.

Cyclohexanecarboxylic acid (5a)[12]

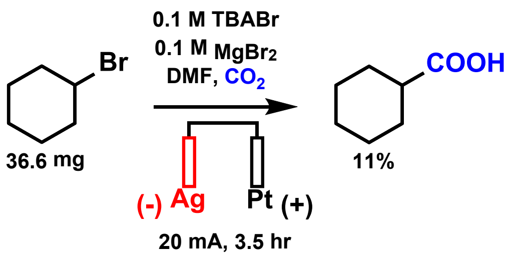

Following **Procedure B**, 0.22 mmol TBABr, 0.22 mmol $MgBr_2$, 2.2 mL DMF, and 36.6 mg bromocyclohexane were combined and subjected to 20 mA for 3.5 hr under 20 sccm CO_2. The product was purified according to **Procedure B** with two modifications. (1) Before rotary evaporating the EtOAc, 1.75 mL of 1 M $NaHCO_3$ was added to convert the acid to basic form to minimize evaporation. The remaining 1.75 mL 1 M $NaHCO_3$ was added after rotary evaporation. (2) The last vacuum drying step was avoided to minimize the risk of product loss given the low yield. This afforded **5a** in 11% yield by ^{1}H NMR with 6.7 mg ethylene carbonate as the internal standard. Since some peaks on the ^{1}H NMR were not perfectly clean, we used the multiplet at 1.95 ppm for quantification. GC-MS indicated the presence of small amounts of pentanoic and butyric acids which could contaminate the tt peak at 2.33 ppm.

^{1}H NMR (500 MHz, $CDCl_3$) The cleanliness of the spectrum is not ideal, with the tt peak at 2.33 ppm contaminated by at least one impurity peak. Nonetheless, there are enough peaks to help confirm the identity of the product **5a** and get a good estimate of the yield.

MS (*m/z*) 128 (M^+, 48.8%), 83 (71.2), 73 (85.7), 68 (48.7), 55 (99.9)

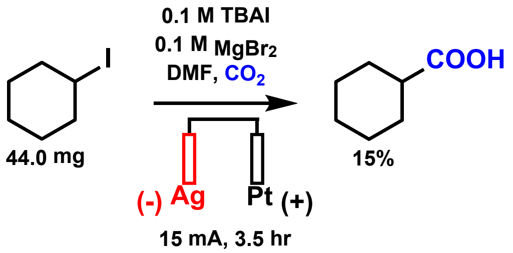

Following **Procedure B**, 0.22 mmol TBAI, 0.22 mmol $MgBr_2$, 2.2 mL DMF, and 44.0 mg iodocyclohexane were combined and subjected to 15 mA for 3.5 hr under 20 sccm CO_2. The product was purified according to **Procedure B** with two modifications. (1) Before rotary evaporating the EtOAc, 1.75 mL of 1 M $NaHCO_3$ was added to convert the acid to basic form to minimize evaporation. The remaining 1.75 mL 1 M $NaHCO_3$ was added after rotary evaporation. (2) The last vacuum drying step was avoided to minimize the risk of product loss given the low yield. This afforded **5a** in 15% yield by ^1H NMR with 7.3 mg ethylene carbonate as the internal standard.

^1H NMR (500 MHz, $CDCl_3$) δ 2.31 (tt, J = 11.24, 3.67 Hz, 1H), 1.98 – 1.88 (m, 2H), 1.82 – 1.72 (m, 2H), 1.65 (m, 1H), 1.53 – 1.39 (m, 2H). Multiplet from 1.37 – 1.18 ppm is obscured by Et_2O and EtOAc peaks.

MS (*m/z*) 128 (M^+, 99.9%), 110 (62.3), 83 (87.6), 73 (88.0), 55 (71.0)

2-Methyl-3-phenylpropanoic acid (6a) and 2-phenylbutyric acid

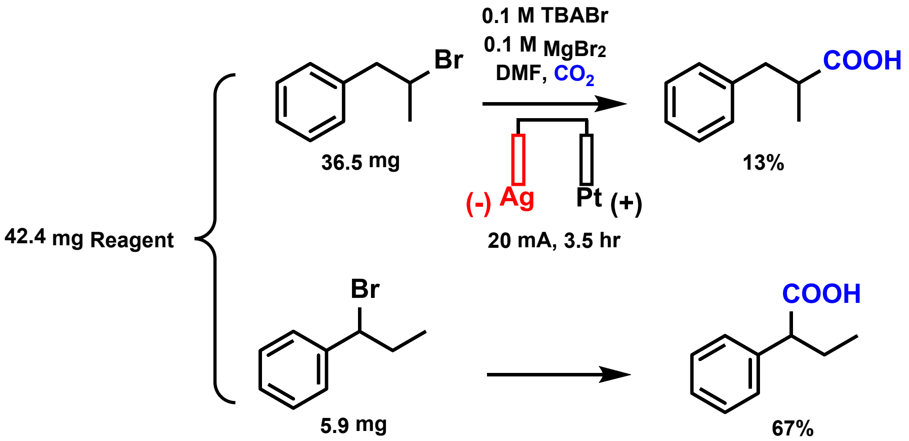

Following **Procedure B**, 0.22 mmol TBABr, 0.22 mmol $MgBr_2$, 2.2 mL DMF, and 42.4 mg 2-bromo-1-phenylpropane reagent were combined and subjected to 20 mA for 3.5 hr under 20 sccm CO_2. The product was purified according to **Procedure B** to give **6a** in 13% yield by ^1H NMR with 7.1 mg of ethylene carbonate as the internal standard. Additionally, the side product 2-phenylbutyric acid was obtained with 67% yield (by initial impurity amount) due to the presence of (1-bromopropyl)benzene impurity in the reagent.

<u>Characterization data for 2-methyl-3-phenylpropanoic acid (**6a**)</u>[17]

^{1}H NMR (500 MHz, $CDCl_3$) δ 8.37 (br, s), 7.35 – 7.26 (m, 3H), 7.24 – 7.16 (m, 2H), 3.07 (dd, *J* = 13.52, 6.52 Hz, 1H), 2.77 (dp, *J* = 7.99, 6.85 Hz, 1H), 2.67 (dd, *J* = 13.52, 8.00 Hz, 1H), 1.18 (d, *J* = 6.92 Hz, 3H).

^{13}C NMR (126 MHz, $CDCl_3$) δ 182.21, 139.08, 129.10, 128.54, 126.55, 41.28, 39.39, 16.60.

MS (*m/z*) 164 (M^+, 38.0%), 118 (17.6), 92 (21.2), 91 (99.9), 65 (21.0)

Characterization data for 2-phenylbutyric acid[18]

^{1}H NMR (500 MHz, $CDCl_3$) δ 8.37 (br, s), 7.35 – 7.26 (m, 3H), 7.24 – 7.16 (m, 2H), 3.47 (t, *J* = 7.69 Hz, 1H), 2.20 – 2.06 (m, 1H), 1.92 – 1.73 (m, 1H), 0.92 (t, *J* = 7.37 Hz, 2H).

^{13}C NMR (126 MHz, $CDCl_3$) δ 180.03, 138.46, 128.77, 128.20, 127.57, 53.37, 26.41, 16.60.

MS (*m/z*) 164 (M^+, 67.3%), 119 (71.4), 91 (99.9), 79 (17.6), 77 (20.7)

Analysis of the 2-bromo-1-phenylpropane reagent

^{1}H NMR analysis indicates that the 2-bromo-1-phenylpropane reagent is actually 86.0% 2-bromo-1-phenylpropane and 14.0% (1-bromopropyl)benzene. These values are used to correct the initial mass of substrate for yield calculations. Note that for the characterization data below, the aromatic peaks cannot be distinguished for each compound.

Characterization data for 2-bromo-1-phenylpropane[19]

^{1}H NMR (500 MHz, $CDCl_3$) δ 4.34 (ddt, *J* = 13.74, 7.09, 6.64 Hz, 1H), 3.26 (dd, *J* = 13.98, 7.01 Hz, 1H), 3.11 (dd, *J* = 13.97, 7.26 Hz, 1H), 1.73 (d, *J* = 6.62 Hz, 3H).

^{13}C NMR (126 MHz, $CDCl_3$) δ 138.64, 129.34, 128.58, 126.98, 50.66, 47.66, 25.81.

Characterization data for (1-bromopropyl)benzene[20]

^{1}H NMR (500 MHz, $CDCl_3$) δ 4.92 (dd, *J* = 8.08, 6.74 Hz, 1H), 2.33 (ddq, *J* = 14.52, 8.00, 7.30 Hz, 1H), 2.25 – 2.16 (m, 1H), 1.04 (t, *J* = 7.26 Hz, 3H).

^{13}C NMR (126 MHz, $CDCl_3$) δ 142.27, 128.77, 128.39, 127.42, 57.72, 33.41, 13.14.

2,2-Dimethyl butyric acid (7a)

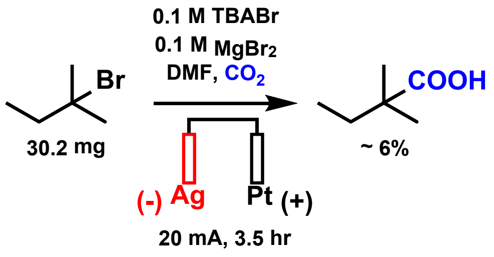

Following **Procedure B**, 0.22 mmol TBABr, 0.22 mmol $MgBr_2$, 2.2 mL DMF, and 30.2 mg 2-bromo-2-methylbutane were combined and subjected to 20 mA for 3.5 hr under 20 sccm CO_2. **Procedure B** was followed for purification, but workup was shortened since product analysis

was only done via GCMS and GC-FID. EtOAc instead of Et_2O was used for the first three organic extractions. The combined organic layers were washed three times with 3.5 mL water, and then these water layers were extracted once with 3.5 mL Et_2O. The combined organic layers were dried with $MgSO_4$, and 22.1 mg 1,3,5-trimethoxybenzene was added as internal standard. An estimate of the yield was performed from GC-FID to give around ~6% of **7a**. The yield is low likely due to hydrogenation being predominant (see details for **8a**). Because the yield was low, further isolation was not attempted.

MS (*m/z*) 101 (6.3%), 88 (25.3), 73 (14.2), 72 (6.7), 71 (99.9), 70 (7.7), 59 (9.1), 57 (5.0), 55 (21.0), 53 (4.7)

1-Adamantane carboxylic acid (8a)

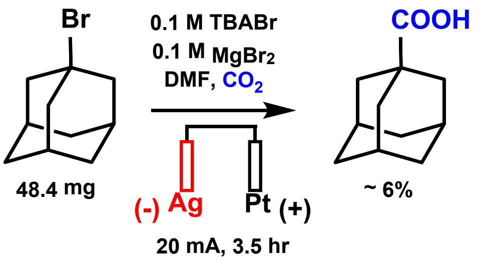

Following **Procedure B**, 0.22 mmol TBABr, 0.22 mmol $MgBr_2$, 2.2 mL DMF, and 48.4 mg 1-bromoadamantane were combined and subjected to 20 mA for 3.5 hr under 20 sccm CO_2. **Procedure B** was followed for purification, but workup was shortened since product analysis was only done via GCMS and GC-FID. EtOAc instead of Et_2O was used for the first three organic extractions. The combined organic layers were washed three times with 3.5 mL water, and then these water layers were extracted once with 3.5 mL Et_2O. The combined organic layers were dried with $MgSO_4$, and 20.9 mg 1,3,5-trimethoxybenzene was added as internal standard. An estimate of the yield was performed from GC-FID to give around ~6% of **8a**. The yield is low mostly due to predominant hydrogenation but also from incomplete substrate conversion. Because the yield was low, further isolation was not attempted.

MS (*m/z*) 180 (M^+, 9.1%), 136 (13.8), 135 (99.9), 107 (11.1), 93 (20.9), 91 (11.3), 81 (5.7), 79 (23.6), 77 (11.7), 67 (7.0)

Phenylacetic acid (9a)[21]

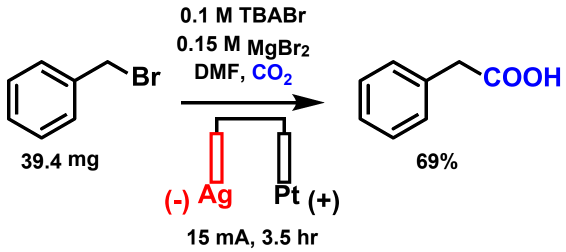

Following **Procedure B**, 0.22 mmol TBABr, 0.33 mmol $MgBr_2$, 2.2 mL DMF, and 39.4 mg benzyl bromide were combined and subjected to 15 mA for 3.5 hr under 20 sccm CO_2. The product was purified according to **Procedure B** to give **9a** in 69% yield by ^{1}H NMR with 4.9 mg

ethylene carbonate as the internal standard. A synthesis carried out at 20 mA resulted in a lower yield of 57%. At 10 mA, the acid yield was also lower due to increased formation of bibenzyl, possibly due to a less negative cathodic voltage slowing down the rate of benzyl radical reduction to a benzyl anion. A lower bubbling rate of 10 sccm CO_2 resulted in a 59% yield.

^{1}H NMR (500 MHz, $CDCl_3$) δ 10.8 (br, s), 7.36 – 7.30 (m, 2H), 7.30 – 7.25 (m, 3H) 3.64 (s, 2H).

^{13}C NMR (126 MHz, $CDCl_3$) δ 178.12, 133.36, 129.52, 128.79, 127.50, 41.19.

MS (*m/z*) 136 (M^+, 67.2%), 92 (43.0), 91 (99.9), 65 (35.0), 63 (17.4)

2-([1,1'-Biphenyl]-3-yl)acetic acid (11a)[21]

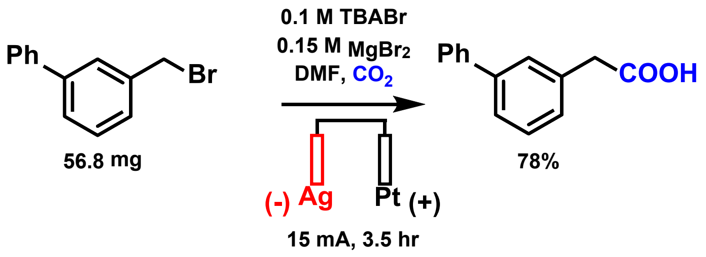

Following **Procedure B**, 0.22 mmol TBABr, 0.33 mmol $MgBr_2$, 2.2 mL DMF, and 56.8 mg 3-phenyl benzyl bromide were combined and subjected to 15 mA for 3.5 hr under 20 sccm CO_2. The product was purified according to **Procedure B** to give **11a** in 78% yield by ^{1}H NMR with 7.0 mg ethylene carbonate as the internal standard. Another synthesis where the reaction mixture was allowed to sit in the cell for ~ 45 min without CO_2 bubbling resulted in a lower yield of 49%.

^{1}H NMR (500 MHz, $CDCl_3$) δ 9.05 (br, s), 7.59 – 7.54 (m, 2H), 7.52 – 7.49 (m, 2H), 7.44 – 7.37 (m, 4H), 7.36 – 7.31 (m, 1H), 7.26 (dt, *J* = 7.6, 1.4 Hz, 1H), 3.71 (s, 2H).

^{13}C NMR (126 MHz, $CDCl_3$) δ 178.04, 141.84, 140.92, 133.80, 129.21, 128.90, 128.43, 127.55, 127.34, 126.38, 41.25.

2-(3-Cyanophenyl)acetic acid (12a)[22]

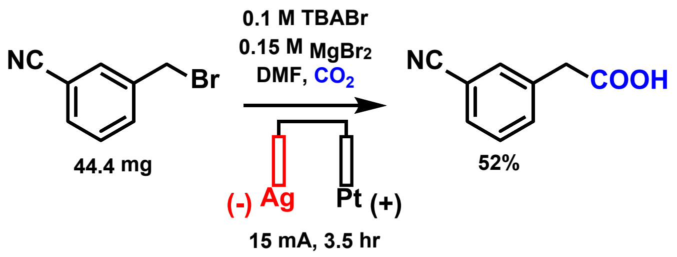

Following **Procedure B**, 0.22 mmol TBABr, 0.33 mmol $MgBr_2$, 2.2 mL DMF, and 44.4 mg 3-cyano benzyl bromide were combined and subjected to 15 mA for 3.5 hr under 20 sccm CO_2. The product was purified according to **Procedure B** to give **12a** in 52% yield by ^{1}H NMR with 6.7 mg ethylene carbonate as the internal standard.

^{1}**H NMR** (500 MHz, $CDCl_3$) δ 9.72 (br, s), 7.62 – 7.56 (m, 2H), 7.56 – 7.52 (m, 1H), 7.46 (td, *J* = 7.70, 0.69 Hz 1H), 3.71 (s, 2H).

^{13}**C NMR** (126 MHz, $CDCl_3$) δ 176.68, 134.70, 134.12, 133.12, 131.30, 129.59, 118.57, 112.92, 40.46.

2-(*m*-Tolyl)acetic acid (10a)[22]

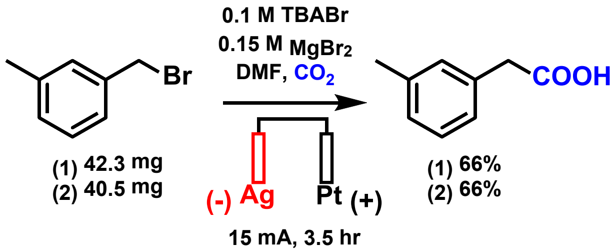

Following **Procedure B**, 0.22 mmol TBABr, 0.33 mmol $MgBr_2$, 2.2 mL DMF, and 42.3 mg 3-methyl benzyl bromide were combined and subjected to 15 mA for 3.5 hr under 20 sccm CO_2. The product was purified according to **Procedure B**. This afforded **10a** in 66% yield by ^{1}H NMR with 7.3 mg ethylene carbonate as the internal standard. A repetition of this experiment resulted in an identical yield of 66%.

^{1}**H NMR** (500 MHz, $CDCl_3$) δ 10.8 (br, s), 7.21 (t, 1H), 7.10 – 7.06 (m, 3H), 3.60 (s, 2H), 2.33 (s, 3H).

^{13}**C NMR** (126 MHz, $CDCl_3$) δ 178.16, 138.43, 133.24, 130.21, 128.63, 128.20, 126.47, 41.09, 21.41.

MS (*m/z*) 150 (M^+, 72.8%), 106 (34.1), 105 (99.9), 91 (42.1), 77 (36.7)

4-Tolylacetic acid (19a)[23]

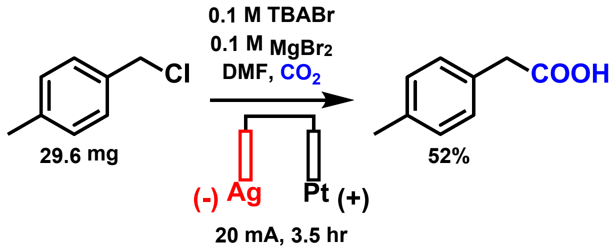

Following **Procedure B**, 0.22 mmol TBABr, 0.22 mmol $MgBr_2$, 2.2 mL DMF, and 29.6 mg 4-methyl benzyl chloride were combined and subjected to 20 mA for 3.5 hr under 20 sccm CO_2. The product was purified according to **Procedure B**. This afforded **10a** in 52% yield by ^{1}H NMR with 7.1 mg ethylene carbonate as the internal standard.

^{1}**H NMR** (500 MHz, $CDCl_3$) δ 7.19 – 7.10 (m, 4H), 3.59 (s, 2H), 2.32 (s, 3H).

^{13}**C NMR** (126 MHz, $CDCl_3$) δ 178.23, 137.10, 130.30, 129.42, 129.32, 40.69, 21.15.

2-(4-(Methoxycarbonyl)phenyl)acetic acid (13a)[13]

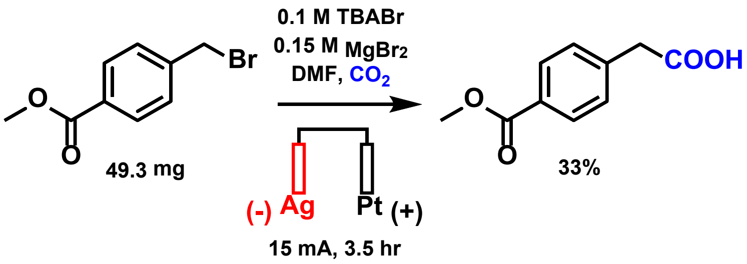

Following **Procedure B**, 0.22 mmol TBABr, 0.33 mmol $MgBr_2$, 2.2 mL DMF, and 49.3 methyl 4-(bromomethyl)benzoate were combined and subjected to 15 mA for 3.5 hr under 20 sccm CO_2. The electrolyte solution was first vacuum distilled to remove DMF. Then, the residue was mixed with 1.75 mL 1 M $NaHCO_3$ and extracted 2x with 3.5 mL Et_2O. The basic aqueous layer was acidified with a 1.5 M pH 2 phosphate buffer (the pH ranges were chosen to avoid hydrolysis of the ester functionality). The acidic aqueous solution was extracted 3x with 3.5 mL Et_2O, washed with MilliQ, and dried with $MgSO_4$. The organic layers were filter, solvent was removed by rotary evaporation, and the resulting residue was dried under vacuum. This afforded **13a** in 33% yield by ^{1}H NMR with 9.5 mg ethylene carbonate as the internal standard.

^{1}H NMR (500 MHz, $CDCl_3$) δ 8.00 (d, J = 8.3 Hz, 2H), 7.40 – 7.33 (m, 2H), 3.91 (s, 3H), 3.71 (s, 2H).

^{13}C NMR (126 MHz, $CDCl_3$) δ 176.81, 167.00, 138.46, 130.06, 129.61, 129.39, 52.31, 41.04.

2-Phenylpropanoic acid (14a)[21]

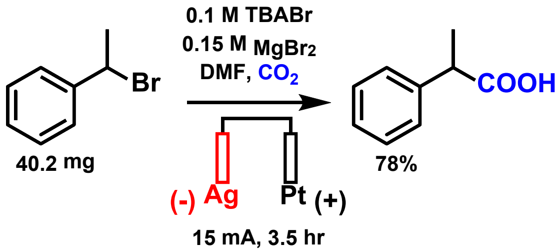

Following **Procedure B**, 0.22 mmol TBABr, 0.33 mmol $MgBr_2$, 2.2 mL DMF, and 40.2 mg (1-bromoethyl)benzene were combined and subjected to 15 mA for 3.5 hr under 20 sccm CO_2. The product was purified according to **Procedure B** to give **14a** in 78% yield by ^{1}H NMR with 6.8 mg ethylene carbonate as the internal standard.

^{1}H NMR (500 MHz, $CDCl_3$) δ 11.06 (br, s), 7.34 – 7.30 (m, 4H), 7.28 – 7.24 (m, 1H), 3.73 (q, J = 7.17 Hz, 1H), 1.51 (d, J = 7.17 Hz, 3H).

^{13}C NMR (126 MHz, $CDCl_3$) δ 180.92, 139.86, 128.78, 127.71, 127.50, 45.47, 18.20.

MS (*m/z*) 150 (M^+, 58.4%), 105 (99.9), 103 (29.1), 79 (35.5), 77 (38.9)

2-(4-Fluorophenyl)propanoic acid (16a)[22]

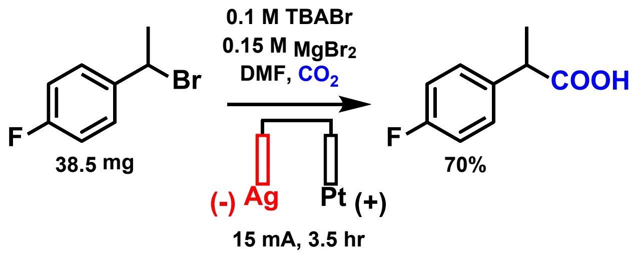

Following **Procedure B**, 0.22 mmol TBABr, 0.33 mmol $MgBr_2$, 2.2 mL DMF, and 38.5 mg 1-(1-bromoethyl)-4-fluorobenzene were combined and subjected to 15 mA for 3.5 hr under 20 sccm CO_2. The product was purified according to **Procedure B** to give **16a** in 70% yield by ^1H NMR with 6.8 mg ethylene carbonate as the internal standard.

^{1}H NMR (500 MHz, $CDCl_3$) δ 9.80 (br, s), 7.31 – 7.26 (m, 2H), 7.04 – 6.98 (m, 2H), 3.72 (q, *J* = 7.19 Hz, 1H), 1.50 (d, *J* = 7.21 Hz, 3H).

^{13}C NMR (126 MHz, $CDCl_3$) δ 180.66, 162.20 (d, *J* = 245.56 Hz), 135.54, 129.30 (d, *J* = 8.21 Hz), 115.61 (d, *J* = 21.92 Hz), 44.70, 18.31.

^{19}F NMR (471 MHz, $CDCl_3$) δ -115.30 (td, *J* = 8.62, 4.51 Hz).

MS (*m/z*) 168 (M^+, 54.1%), 124 (22.2), 123 (99.9), 103 (67.1), 77 (26.8)

2-(4-Isobutylphenyl)propanoic acid (15a)[22]

Synthesis of 1-(1-bromoethyl)4-isobutylbenzene

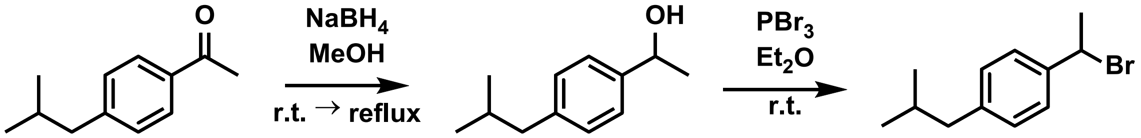

1-(1-Bromoethyl)-4-isobutylbenzene was synthesized using a procedure from the literature.[22] Sodium borohydride ($NaBH_4$, 320 mg) was added to a stirred solution of 4-isobutylacetophenone (1.04 mL) in methanol (7 mL). The solution was refluxed for 1 hr and then cooled to room temperature. 1 M HCl (5 mL) was added, and then the MeOH was distilled off. The resulting aqueous acid phase was extracted 3x with 5 mL EtOAc. The combined organic phases were washed 2x with 15 mL brine and dried with Na_2SO_4. Rotavapping the EtOAc afforded 1-hydroxy-1-(4-isobutylphenyl)ethane, as confirmed by GCMS.

The alcohol was added to 20 mL Et_2O, and then 600 µL of PBr_3 was added. The solution was stirred for 1 hr at room temperature. The resulting solution was partially rotavapped and treated with H_2O. Three Et_2O extractions were performed on the aqueous layer, and the combined organic layers were washed with brine, dried with $MgSO_4$, filtered, and rotavapped. The resulting residue was concentrated under vacuum to afford 1-(1-bromoethyl)-4-isobutylbenzene.

^{1}H NMR[24] (500 MHz, $CDCl_3$) δ 7.34 – 7.31 (m, 2H), 7.11 – 7.07 (m, 2H), 5.21 (q, *J* = 6.9 Hz, 1H), 2.45 (d, *J* = 7.2 Hz, 2H), 2.03 (d, *J* = 6.9 Hz, 3H), 1.96 – 1.75 (m, 1H), 0.89 (d, *J* = 6.6 Hz, 6H).

^{13}C NMR (126 MHz, $CDCl_3$) δ 142.13, 140.66, 129.47, 126.67, 50.01, 45.22, 30.25, 26.96, 22.50.

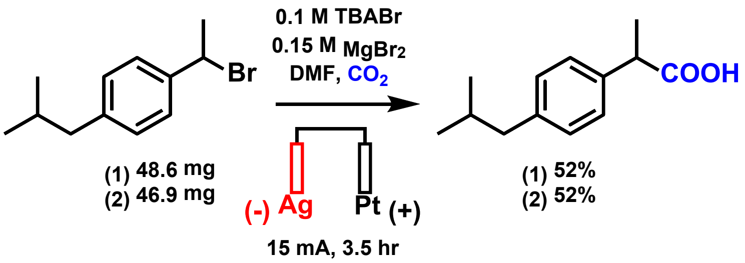

Following **Procedure B**, 0.22 mmol TBABr, 0.33 mmol $MgBr_2$, 2.2 mL DMF, and 48.6 mg 1-(1-bromoethyl)-4-isobutylbenzene were combined and subjected to 15 mA for 3.5 hr under 20 sccm CO_2. The product was purified according to a modified form of **Procedure B**, where Et_2O was used instead of EtOAc and hexane. The initial extractions with EtOAc were replaced with 4x 3.5 mL Et_2O extractions (using only glass centrifuge tubes). The last set of MilliQ washes were condensed to just one 1.75 mL wash before drying with $MgSO_4$. These steps gave **15a** in 52% yield by ^{1}H NMR with 10.4 mg ethylene carbonate as the internal standard. This experiment was repeated a second time and resulted in a yield of 52%.

^{1}H NMR (500 MHz, $CDCl_3$) δ 7.25 – 7.15 (m, 2H), 7.16 – 7.05 (m, 2H), 3.70 (q, J = 7.1 Hz, 1H), 2.44 (d, J = 7.2 Hz, 2H), 1.85 (dh, J = 13.4, 6.7 Hz, 1H), 1.49 (d, J = 7.2 Hz, 3H), 0.89 (d, J = 6.6 Hz, 6H).

^{13}C NMR (126 MHz, $CDCl_3$) δ 180.86, 140.95, 137.10, 129.48, 127.37, 45.12, 45.04, 30.25, 22.48, 18.19.

2-Methyl-2-phenylpropanoic acid (17a)

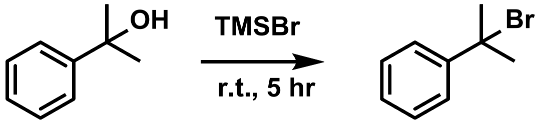

(2-bromopropan-2-yl)benzene was synthesized according to a procedure from the literature.[25] 2-Phenylpropan-2-ol (335 mg) was stirred with 244 µL of bromotrimethylsilane (TMSBr) at room temperature for 5 hr. The resulting mixture was rotavapped at 35 °C and furthered concentrated under vacuum.

^{1}H NMR[25] (500 MHz, $CDCl_3$) δ 7.74 – 7.54 (m, 2H), 7.37 – 7.31 (m, 2H), 7.29 – 7.26 (m, 1H), 2.20 (s, 6H).

^{13}C NMR (126 MHz, $CDCl_3$) δ 146.88, 128.40, 127.87, 125.85, 64.22, 35.60.

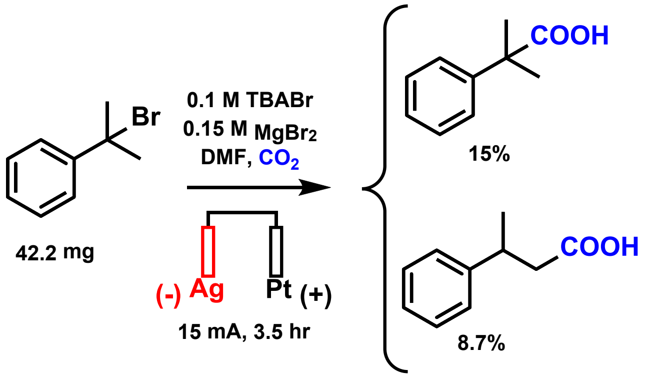

Following **Procedure B**, 0.22 mmol TBABr, 0.33 mmol $MgBr_2$, 2.2 mL DMF, and 42.2 mg (2-bromopropan-2-yl)benzene were combined and subjected to 15 mA for 3.5 hr under 20 sccm CO_2. The product was purified according to a modified form of **Procedure B**, where Et_2O was used instead of EtOAc and hexane. The initial extractions with EtOAc were replaced with 4x 3.5 mL Et_2O extractions (using only glass centrifuge tubes). The last set of MilliQ washes were condensed to just one 1.75 mL wash before drying with $MgSO_4$. These steps gave **17a** in 15% yield by ^{1}H NMR with 11.1 mg ethylene carbonate as the internal standard. Additionally, 3-phenylbutyric acid was made in 8.7% yield. A second experiment at 20 mA and 0.2 M TBAI resulted in a lower yield of 6.8%.

<u>2-Methyl-2-phenylpropanoic acid</u>[26]

^{1}H NMR (500 MHz, $CDCl_3$) δ 1.72 (s, 6H) (aromatic peaks are overlapped)

^{13}C NMR (126 MHz, $CDCl_3$) δ 180.41, 142.02, 128.83, 127.53, 125.74, 48.07, 23.56.

<u>3-Phenylbutyric acid</u>[27]

^{1}H NMR (500 MHz, $CDCl_3$) δ 3.30 – 3.22 (m, 1H), 2.66 (dd, J = 15.5, 6.8 Hz, 1H), 2.57 (dd, J = 15.5, 8.2 Hz, 1H), 1.31 (d, J = 7.0 Hz, 3H).

^{13}C NMR (126 MHz, $CDCl_3$) δ 176.41, 145.61, 128.63, 126.79, 126.54, 43.41, 36.23, 21.94.

Single peak on GCMS with *m/z* 164, suggesting either (1) these two acids are not easily separated or (2) conversion of 2-methyl-2-phenylpropanoic acid to 3-phenylbutyric acid in the GC.

Benzoic acid (18a)[28]

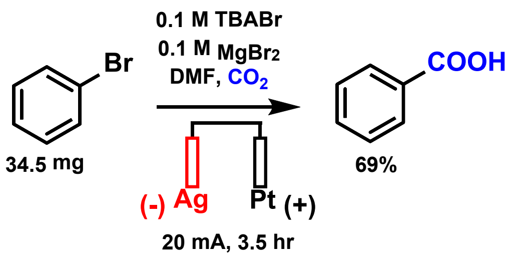

Following **Procedure B**, 0.22 mmol TBABr, 0.22 mmol $MgBr_2$, 2.2 mL DMF, and 34.5 mg bromobenzene were combined and subjected to 20 mA for 3.5 hr under 20 sccm CO_2. The

product was purified according to **Procedure B** to give **18a** in 69% yield by ^{1}H NMR with 4.4 mg ethylene carbonate as the internal standard.

^{1}H NMR (500 MHz, $CDCl_3$) δ 10.26 (br, s), 8.15 – 8.10 (m, 2H), 7.64 – 7.59 (m, 1H), 7.51 – 7.45 (m, 2H).

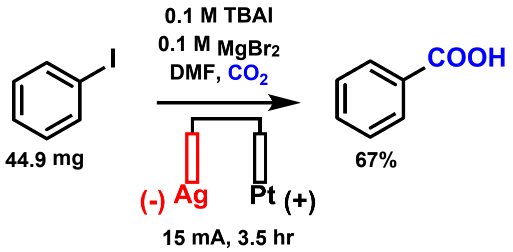

Following **Procedure B**, 0.22 mmol TBAI, 0.22 mmol $MgBr_2$, 2.2 mL DMF, and 44.9 mg iodobenzene were combined and subjected to 15 mA for 3.5 hr under 20 sccm CO_2. The product was purified according to **Procedure B** to give **18a** in 67% yield by ^{1}H NMR with 7.3 mg ethylene carbonate as the internal standard.

^{1}H NMR (500 MHz, $CDCl_3$) δ 11.45 (br, s), 8.17 – 8.09 (m, 2H), 7.67 – 7.59 (m, 1H), 7.52 – 7.45 (m, 2H).

^{13}C NMR (126 MHz, $CDCl_3$) δ 172.44, 133.94, 130.31, 129.42, 128.59.

MS (*m/z*) 122 (M^+, 91.2%), 105 (99.9), 77 (71.7), 51 (31.0), 50 (19.9)

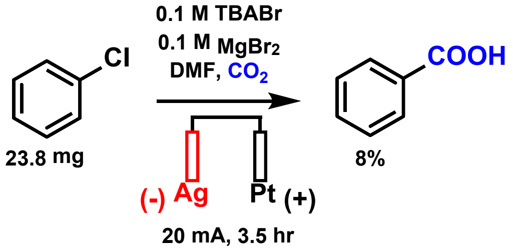

Following **Procedure B**, 0.22 mmol TBABr, 0.22 mmol $MgBr_2$, 2.2 mL DMF, and 23.8 mg chlorobenzene were combined and subjected to 20 mA for 3.5 hr under 20 sccm CO_2. The crude yield of **18a** was found to be 8% yield by ^{1}H NMR. Given the low yield and previous purifications of benzoic acid, isolation of the benzoic acid was not attempted.

11.3 Carboxylation with Sacrificial Anodes

4-Phenylburytic acid (1a)

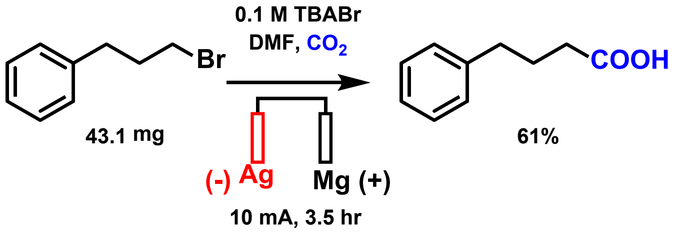

Following **Procedure B**, 0.22 mmol TBABr, 2.2 mL DMF, and 43.1 mg 1-bromo-3-phenylpropane were combined and subjected to 10 mA until the cell voltage exceeded the potentiostat limit (< -10 V) under 20 sccm CO_2. The product was purified according to **Procedure B** to give **1a** in 61% yield by ^{1}H NMR with 7.3 mg of ethylene carbonate as the internal standard.

^{1}H NMR (500 MHz, $CDCl_3$) δ 10.71 (br, s), 7.32 – 7.25 (m, 2H), 7.22 – 7.14 (m, 3H), 2.77 – 2.59 (m, 2H), 2.37 (t, J = 7.45 Hz, 2H), 1.97 (p, J = 7.52 Hz, 2H).

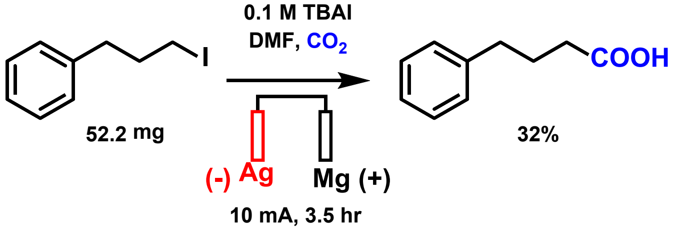

Following **Procedure B**, 0.22 mmol TBAI, 2.2 mL DMF, and 52.2 mg 1-iodo-3-phenylpropane were combined and subjected to 10 mA until the cell voltage exceeded the potentiostat limit (< -10 V) under 20 sccm CO_2. The product was purified according to **Procedure B** to give **1a** in 32% yield by ^{1}H NMR with 6.6 mg of ethylene carbonate as the internal standard.

^{1}H NMR (500 MHz, $CDCl_3$) δ 8.75 (br, s), 7.32 – 7.26 (m, 2H), 7.22 – 7.15 (m, 3H), 2.72 – 2.64 (m, 2H), 2.38 (t, J = 7.45 Hz, 2H), 1.97 (p, J = 7.51 Hz, 2H).

3-Phenylpropanoic acid (2a)

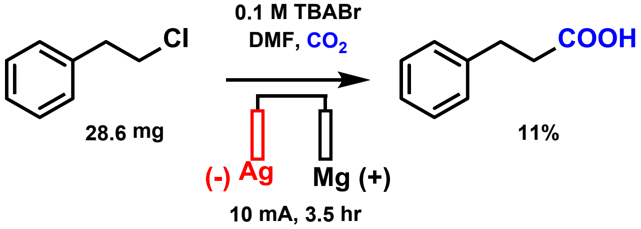

Following **Procedure B**, 0.22 mmol TBABr, 2.2 mL DMF, and 28.6 mg (2-chloroethyl)benzene were combined and subjected to 10 mA until the cell voltage exceeded the potentiostat limit (< -10 V) under 20 sccm CO_2. Since this occurred about 90 min into the run, additional current was passed at -10 V for another 2 hours. The current at -10 V dropped in magnitude below 1 mA/cm^2 after about 30 min. The product was purified according to **Procedure B** to give **2a** in 11% yield by ^{1}H NMR with 6.7 mg of ethylene carbonate as the internal standard.

^{1}H NMR (500 MHz, $CDCl_3$) δ 7.32 – 7.27 (m, 2H), 7.23 – 7.19 (m, 3H), 2.97 (t, J = 7.81 Hz, 2H), 2.71 – 2.66 (m, 2H).

2-Methyl-3-phenylpropanoic acid (6a) and 2-phenylbutyric acid

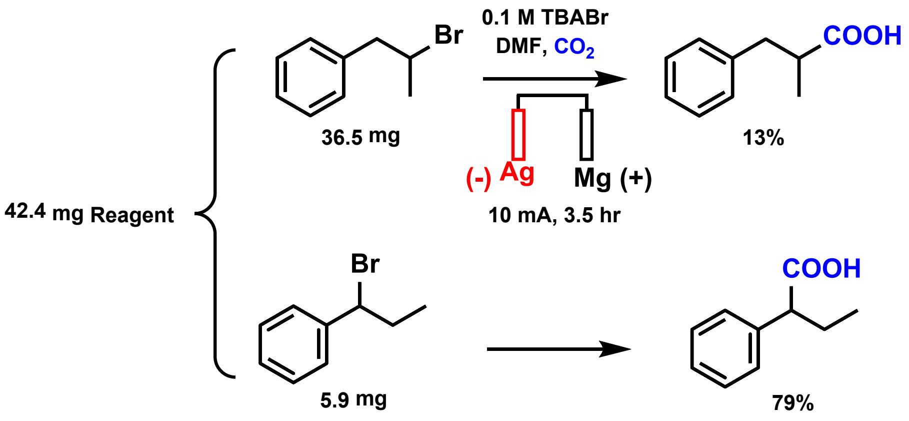

Following **Procedure B**, 0.22 mmol TBABr, 2.2 mL DMF, and 42.4 mg 2-bromo-1-phenylpropane were combined and subjected to 10 mA until the cell voltage exceeded the potentiostat limit (< -10 V) under 20 sccm CO_2. The product was purified according to **Procedure B** to give **6a** in 13% yield by ^{1}H NMR with 7.3 mg of ethylene carbonate as the internal standard. Additionally, the side product 2-phenylbutyric acid was formed in 79% yield (based on initial amount of impurity) due to the presence of (1-bromopropyl)benzene as an impurity in the reagent.

Characterization data for 2-methyl-3-phenylpropanoic acid (**6a**)

^{1}H NMR (500 MHz, $CDCl_3$) δ 9.34 (br, s), 7.36 – 7.26 (m, 3H), 7.23 – 7.15 (m, 2H), 3.06 (dd, *J* = 13.49, 6.47 Hz, 1H), 2.76 (dp, *J* = 8.08, 6.87 Hz, 1H), 2.66 (dd, *J* = 13.48, 7.99 Hz, 1H), 1.17 (d, *J* = 6.94 Hz, 3H).

Characterization data for 2-phenylbutyric acid

^{1}H NMR (500 MHz, $CDCl_3$) δ 9.34 (br, s), 7.36 – 7.26 (m, 3H), 7.23 – 7.15 (m, 2H), 3.46 (t, *J* = 7.82 Hz, 1H), 2.17 – 2.05 (m, 1H), 1.82 (dp, *J* = 13.62, 7.45 Hz, 1H), 0.91 (t, *J* = 7.37 Hz, 3H).

Phenylacetic acid (9a)

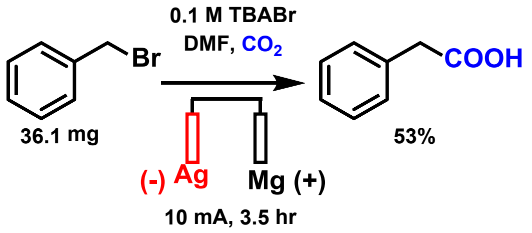

Following **Procedure B**, 0.22 mmol TBABr, 2.2 mL DMF, and 36.1 mg benzyl bromide were combined and subjected to 10 mA until the cell voltage exceeded the potentiostat limit (< -10 V) under 20 sccm CO_2. The product was purified according to **Procedure B** to give **9a** in 53% yield by ^{1}H NMR with 7.1 mg of ethylene carbonate as the internal standard.

^{1}H NMR (500 MHz, $CDCl_3$) δ 7.95 (br, s), 7.35 – 7.31 (m, 2H), 7.30 – 7.26 (m, 3H), 3.64 (s, 2H).

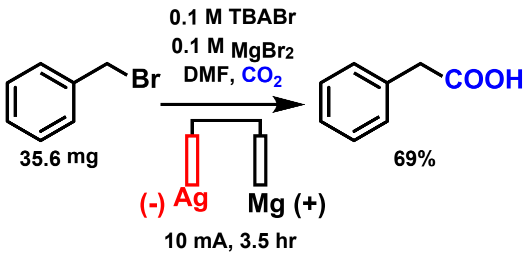

Following **Procedure B**, 0.22 mmol TBABr, 0.22 mm $MgBr_2$, 2.2 mL DMF, and 35.6 mg benzyl bromide were combined and subjected to 10 mA until the cell voltage exceeded the potentiostat limit (< -10 V) under 20 sccm CO_2. The product was purified according to **Procedure B** to give **9a** in 69% yield by ^{1}H NMR with 5.5 mg of ethylene carbonate as the internal standard.

^{1}H NMR (500 MHz, $CDCl_3$) δ 7.39 – 7.33 (m, 2H), 7.33 – 7.28 (m, 3H), 3.67 (s, 2H).

Benzoic acid (18a)

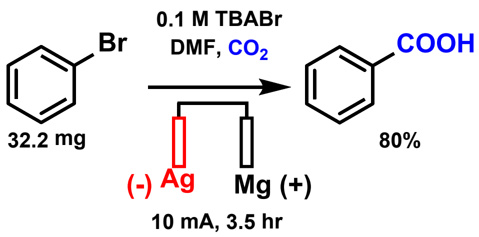

Following **Procedure B**, 0.22 mmol TBABr, 2.2 mL DMF, and 32.2 mg bromobenzene were combined and subjected to 10 mA until the cell voltage exceeded the potentiostat limit (< -10 V) under 20 sccm CO_2. The product was purified according to **Procedure B** to give **18a** in 80% yield by ^{1}H NMR with 2.5 mg of ethylene carbonate as the internal standard.

^{1}H NMR (500 MHz, $CDCl_3$) δ 10.93 (br, s), 8.17 – 8.08 (m, 2H), 7.62 (ddt, J = 8.76, 7.07, 1.33 Hz, 1H), 7.53 – 7.43 (m, 2H).

^{13}C NMR (126 MHz, $CDCl_3$) δ 172.58, 133.96, 130.38, 129.45, 128.62.

11.4 Carboxylation without Magnesium

1-Bromo-3-phenylpropane

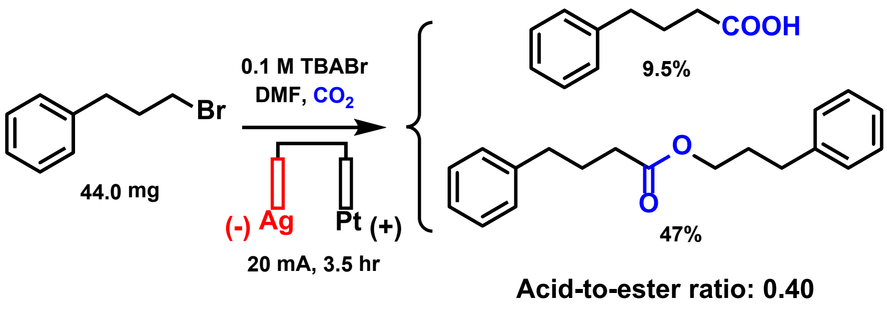

Following **Procedure C1**, 0.22 mmol TBABr, 2.2 mL DMF, and 44.0 mg 1-bromo-3-phenylpropane were combined and subjected to 20 mA for 3.5 hr under 20 sccm CO_2. The product was purified according to **Procedure C1** to give **1a** in 9.5% yield and the ester **1e** in 47% yield by ^{1}H NMR with 19.9 mg of 1,3,5-trimethoxybenzene as the internal standard.

The ester peak in the initial ^{1}H NMR spectrum (4.09 ppm, t)[29] was overlapped by EtOAc, so a further purification was done to isolate the acid from the ester and remove EtOAc. Most of the $CDCl_3$-d_1 was rotavapped, and then 1.75 mL hexane and 1.75 mL 1 M $NaHCO_3$ were added. The hexane layer was extracted, followed by a second 1.75 mL hexane extraction. The combined organic layers were dried over $MgSO_4$, rotavapped, and concentrated under vacuum. Since the TMB internal standard should have remained entirely in the organic fraction, quantification of the ester could be performed after this workup. The amount of ester after additional workup agreed well with the amount found in the initial spectrum containing both acid and ester. The total amount of acid and ester was found from the initial spectrum by integrating the pair of overlapping triplets at 2.36 and 2.32 ppm. These two results enabled the calculation of the acid-to-ester ratio. A similar acid-to-ester ratio of 0.42 was estimated from GCMS-FID. GCMS indicated that the acid, ester, and alkane were the major products with a small amount of substrate. Small amounts of carbonate and alcohol were seen on the ^{1}H NMR, but these are estimated to be a few percent of the ester and difficult to quantify, so they were not included here.

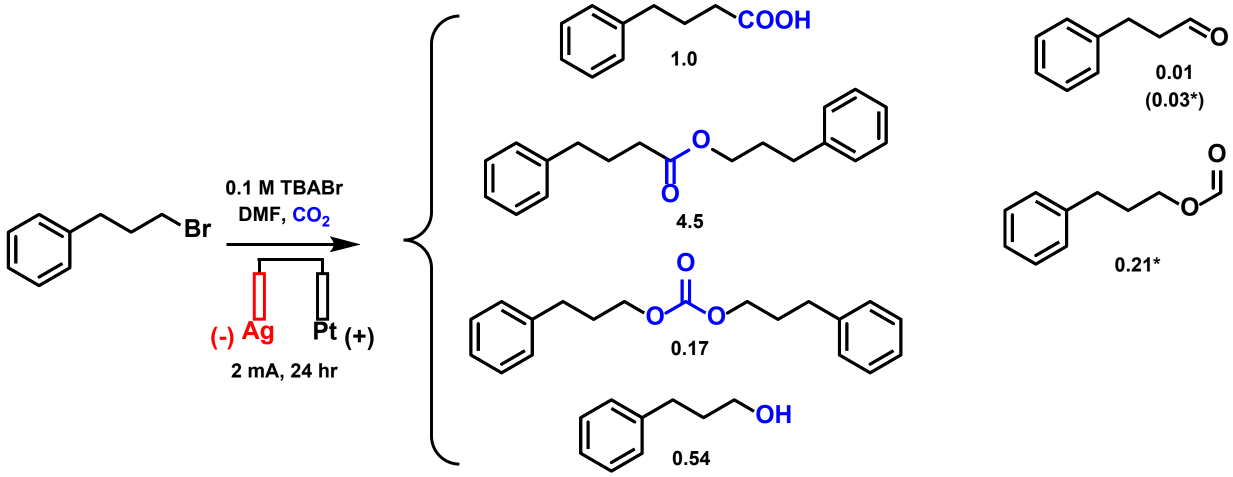

Following **Procedure C1**, 0.22 mmol TBABr, 2.2 mL DMF, and 43.2 mg 1-bromo-3-phenylpropane were combined and subjected to 2 mA for 24 hr under 20 sccm CO_2. The product was purified according to **Procedure C1**. Product ratios were determined via ^{1}H NMR, but absolute quantification was not performed. Compound identification was performed with a combination of ^{1}H & ^{13}C NMR and GCMS. Values with an asterisk denote estimations from GCMS-FID.

<u>^{1}H NMR peaks used for quantification:</u>
Carboxylic acid **1a**: 2.37 ppm (t, J = 7.4 Hz, 2H)[11]
Ester **1e**: 4.09 ppm (t, J = 6.5 Hz, 2H)[29]. Area of triplet at 2.33 ppm also agreed with this area.
Carbonate **1f**: 4.15 ppm (t, J = 6.5 Hz, 4H)[30]. Used integration of fitted peaks due to noisy

baseline.
Alcohol **1d**: 3.68 ppm (t, J = 6.4 Hz, 2H)[11]. Used edited sum integration[31] to remove contribution from singlet overlapping with edge of triplet.
Aldehyde: 9.81 ppm (t, J = 1.4 Hz, 1H)[32]

As noted below for the products from carboxylation of 1-iodo-3-phenylpropane without a magnesium source, there is an additional aldehyde peak at 9.75 ppm (t, J = 1.7 Hz, 1H), which could be from 4-phenylbutanal.[33] There is a peak in for *m/z* 148 around 9.69 min, but it is heavily overlapped by BHT. There are peaks at 8.07 ppm, which could be from the formate ester as detected by GCMS.[34] There are also numerous peaks downfield of the ester peak at 4.09 ppm which could be from various unidentified oxidation products (see discussion for benzyl bromide).

<u>^{13}C NMR peaks used for identification:</u>
Carboxylic acid **1a**: 178.52, 35.11, 33.21, 26.34 ppm
Ester **1e**: 173.73, 141.51, 141.31, 63.86, 35.28, 33.76, 32.33, 30.35, 26.66 ppm
Carbonate **1f**: 67.38 ppm
Alcohol **1d**: 62.43 ppm

GCMS was able to identify the alcohol, carboxylic acid, aldehyde, and formate ester. The ester is likely the large peak around 20 min, but its mass spectrum is not readily available for comparison. The carbonate was probably too heavy to be detected within the time of the experimental run.

1-Iodo-3-phenylpropane

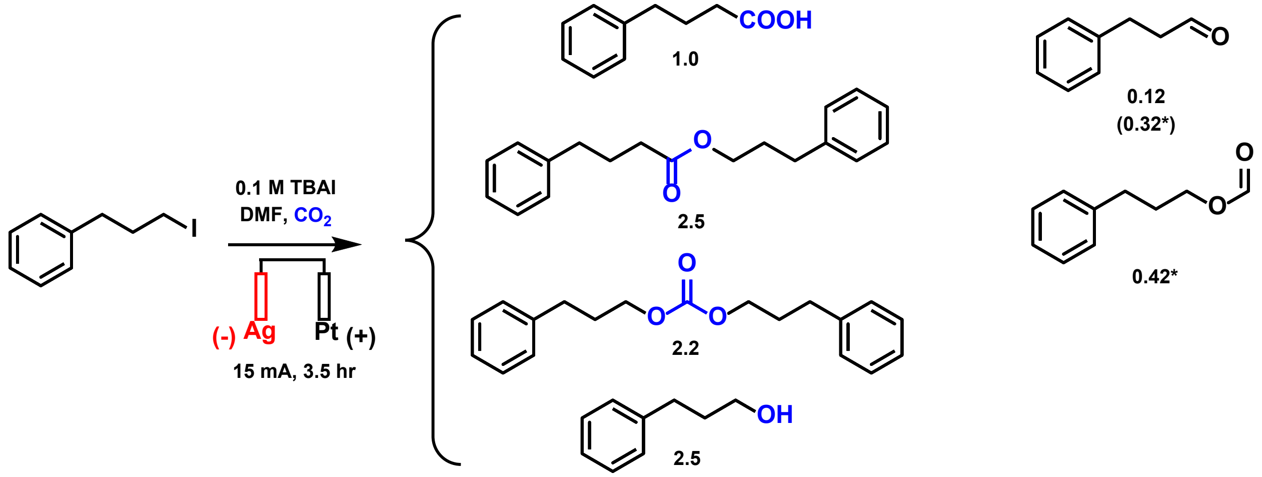

Following **Procedure C1**, 0.22 mmol TBAI, 2.2 mL DMF, and 50.7 mg 1-iodo-3-phenylpropane were combined and subjected to 15 mA for 3.5 hr under 20 sccm CO_2. The product was purified according to **Procedure C1**. Product ratios were determined via ^{1}H NMR, but absolute quantification was not performed. Compound identification was performed with a combination of ^{1}H & ^{13}C NMR and GCMS. Values with an asterisk denote estimations from GCMS-FID.

^{1}H NMR peaks used for quantification:
Carboxylic acid **1a**: 2.37 ppm (t, J = 7.4 Hz, 2H)[11]
Ester **1e**: 2.33 ppm (t, J = 7.5 Hz, 2H)[29]
Carbonate **1f**: 4.15 ppm (t, J = 6.5 Hz, 4H)[30]
Alcohol **1d**: 3.68 ppm (t, J = 6.4 Hz, 2H)[11]
Aldehyde: 9.81 ppm (t, J = 1.4 Hz, 1H)[32]

We did not use the triplet at 4.09 ppm to quantify the ester because it appeared to be too large, contaminated by a smaller peak. There are peaks at 8.08 ppm, which support the presence of the formate ester as detected by GCMS.[34] Moreover, we consistently detected the analogous formate ester when benzyl bromide was the substrate. We also note the presence of a second aldehyde peak at 9.75 ppm (t, J = 1.7 Hz, 1H), which could be from 4-phenylbutanal.[33] While there is an ion peak at *m/z* 148 in the GCMS spectrum at 9.64 min, it is too close to the BHT peak at 9.7 min to reliably distinguish.

^{13}C NMR peaks used for identification:
Carboxylic acid **1a**: 178.59 ppm
Ester **1e**: 173.78, 63.88 ppm
Carbonate **1f**: 155.46, 67.39 ppm
Alcohol **1d**: 62.45 ppm

We also note that there are 4 prominent peaks between 141 and 142 ppm, corresponding to the carboxylic acid, ester (2 peaks), and carbonate.

GCMS was able to identify the alcohol, carboxylic acid, aldehyde, and formate ester. The ester does appear, but it does not have a readily comparable standard MS spectrum. The carbonate did not appear, likely due to an elution time longer than the acquisition time.

In addition to the ester and carbonate, there are numerous other small peaks in the region 4.35 – 4.05 ppm in the ^{1}H NMR spectrum. Most of these are shifted downfield of the ester and carbonate, so they likely represent oxidized side products (see discussion for benzyl bromide). Subtracting off the areas of the carbonate and ester, the remaining area in this region is 2.2 x the area of the carboxylic acid, implying more side reactions are present in the absence of an Mg source. At the same time, there are several smaller peaks in the GCMS spectrum that are not easily identified, which are likely related to these unidentified peaks in the ^{1}H NMR spectrum. There are also two smaller ^{13}C NMR peaks (66.76, 65.05 ppm) which are unassigned.

(2-Chloroethyl)benzene

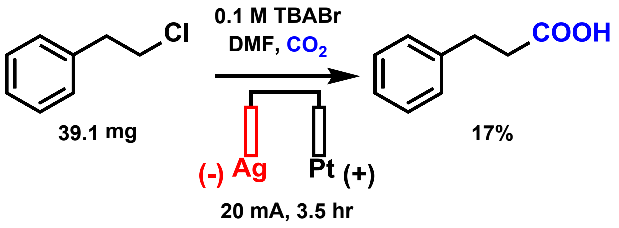

Following **Procedure C1**, 0.22 mmol TBABr, 2.2 mL DMF, and 39.1 mg bromobenzene were combined and subjected to 20 mA for 3.5 hr under 20 sccm CO_2. The product was purified according to **Procedure C1** to give **2a** in 17% yield by ^1H NMR with 20.9 mg of 1,3,5-trimethoxybenzene as the internal standard.

^1H NMR, ^{13}C NMR, and GCMS indicate that the two main detectable species are the substrate and carboxylic acid **2a**. There may be some ester, which has a triplet at 4.28 ppm,[35] but this amount is estimated to be ≤ 3% of the acid based on the area of a small set of peaks around 4.28 ppm in the ^1H NMR spectrum.

Bromocyclohexane

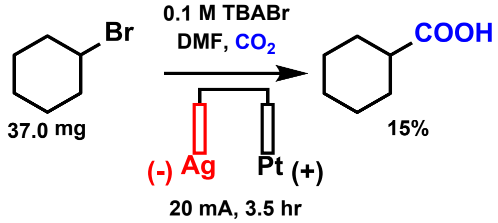

Following **Procedure C1**, 0.22 mmol TBABr, 2.2 mL DMF, and 37.0 mg bromocyclohexane were combined and subjected to 20 mA for 3.5 hr under 20 sccm CO_2. The product was purified according to **Procedure C1** to give **5a** in 15% yield by ^1H NMR with 20.6 mg of 1,3,5-trimethoxybenzene as the internal standard.

Based on ^1H NMR and GCMS, the majority species derived from the starting material is the carboxylic acid **5a**; small amounts of substrate and other side products could be present. The amount of ester, which has a multiplet at 4.74 ppm,[36] formed is ≤ 1% of the acid based on the integration of this region in the ^1H NMR spectrum.

Iodocylcohexane

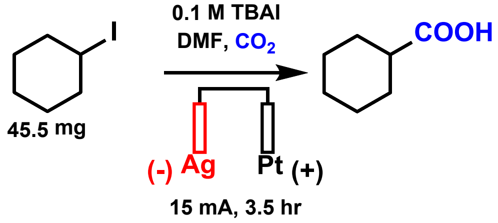

Following **Procedure C1**, 0.22 mmol TBAI, 2.2 mL DMF, and 45.5 mg iodocyclohexane were combined and subjected to 15 mA for 3.5 hr under 20 sccm CO_2. The product was purified according to **Procedure C1** to give **5a**. No peaks in the ^1H NMR spectrum were detected at 4.74 ppm where the ester would be,[36] so the ester yield should be < 1%. GCMS also indicates no presence of ester, with the major product being the carboxylic acid.

1-Bromo-5-chloropentane

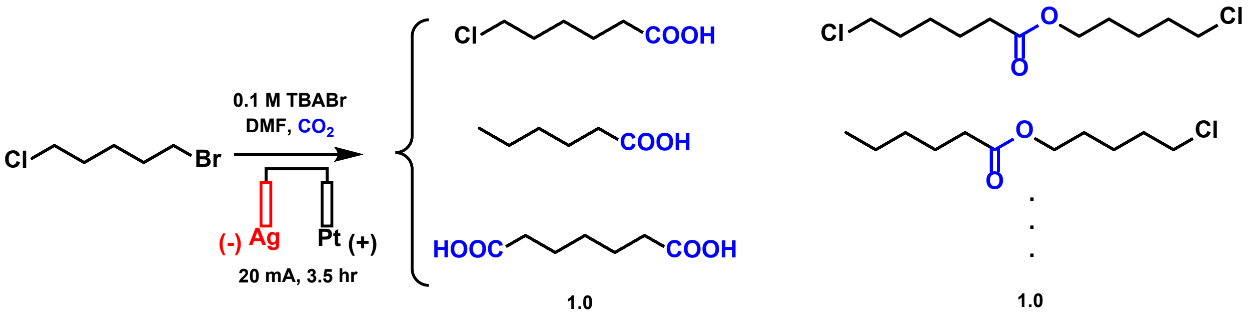

Following **Procedure C1**, 0.22 mmol TBABr, 2.2 mL DMF, and 39.3 mg 1-bromo-5-chloropentane were combined and subjected to 20 mA for 3.5 hr under 20 sccm CO_2. The product was purified according to **Procedure C1**. Due to the large number of products, we only calculated the acid-to-ester ratio using ^{1}H NMR. The presence of two potential carboxylation sites on the substrate could lead to polyester products as well. The acid-to-ester ratio calculated here is actually a ratio of carboxylation events that did not lead to an ester relative to carboxylation events that did lead to an ester. 1,3,5-Trimethoxybenzene was added as an internal standard, but we did not attempt to quantify yields. We also note some N-methyl pyrrolidinone contamination occurred during vacuum drying.

^{1}H NMR peaks for quantification

Esters: Pair of triplets at 4.08 and 4.09 ppm (J = 6.6 Hz, 2H for both)
Carboxylic acids + Esters: Peaks from 2.38 to 2.28 ppm (2H). We actually integrated the region from 2.45 to 2.28 ppm and subtracted the area from NMP contamination.
NMP contamination: 2.85 ppm (s, 3H)
Chlorides: Pair of triplets at 3.54 and 3.53 ppm (2H for both)

Based on the chloride area, the fraction of terminal carbons with a chlorine attached is 76%.

^{13}C NMR peaks for identification

Carboxylic acids: 3 total peaks at 178.67, 178.61, 178.42
Esters: 2 total peaks at 173.76 and 173.66, 2 total peaks at 64.17, 64.11
Chlorides: 2 total peaks at 44.85, 44.82

^{13}C NMR suggests there are 3 distinct carboxylic acids, 2 distinct esters, and 2 distinct carbon chloride bonds present in the mixture, in agreement with what is found in ^{1}H NMR.

GCMS was able to identify two carboxylic acids (**4a** and hexanoic acid); 1,7-heptanedioic acid is inferred as a potential product from carboxylation in the presence of $MgBr_2$. However, the chlorinated esters were not readily identifiable since they do not have known mass spectra (or even NMR spectra). Thus, we can only report the ratio of total acids to total esters without knowing the precise nature of the esters present, although the ones shown above are believed to be most likely based on the distribution of carboxylic acids from carboxylation with $MgBr_2$.

We also looked for ε-caprolactone from an intramolecular carboxylate nucleophilic attack, but GCMS and ^{1}H NMR could not confirm the presence of this product. This result is not too surprising given the low susceptibility of alkyl chlorides to carboxylate nucleophilic attack.

2-Bromo-1-phenylpropane

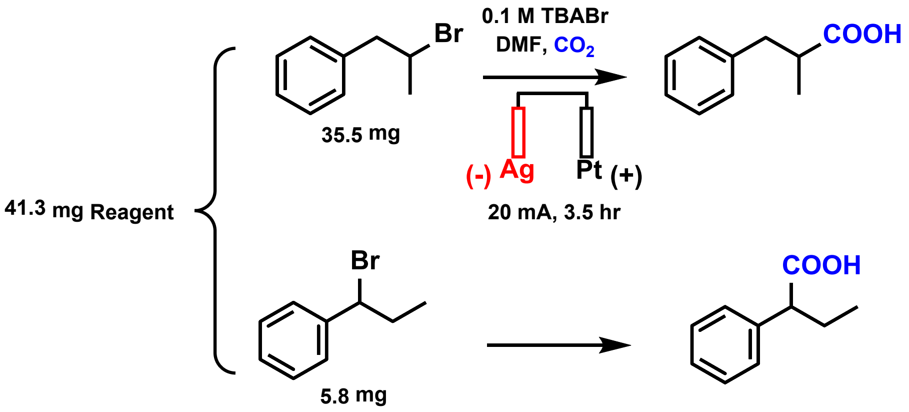

Following **Procedure C1**, 0.22 mmol TBABr, 2.2 mL DMF, and 41.3 mg 2-bromo-1-phenylpropane were combined and subjected to 20 mA for 3.5 hr under 20 sccm CO_2. The product was purified according to **Procedure C1**. Only the two carboxylic acids were detected by ^{1}H and ^{13}C NMR. There were no discernable peaks in the ^{1}H spectrum region 5 – 6 ppm where both esters should have peaks, and there were also no peaks in the ^{13}C NMR (aside from $CDCl_3$) from 90 to 60 ppm.

(1-Bromoethyl)benzene

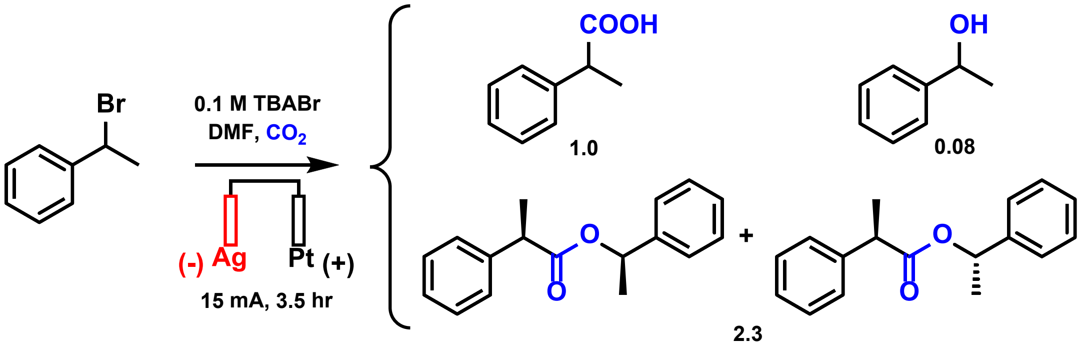

Following **Procedure C1**, 0.22 mmol TBABr, 2.2 mL DMF, and 39.0 mg (1-bromoethyl)benzene were combined and subjected to 15 mA for 3.5 hr under 20 sccm CO_2. The product was purified according to **Procedure C1**. Product ratios were determined by ^{1}H NMR. The presence of ester enantiomers is confirmed by ^{13}C NMR. Their ratio appears to be close to 1:1, although the *syn* peaks are consistently taller than the *anti* peaks.

<u>^{1}H NMR peaks for quantification</u>
Carboxylic acid + Esters: 3.77 – 3.71 ppm (1H) (acid is found by subtracting ester area from this)[22,37]
Esters: 5.86 ppm (p, J = 6.6 Hz, 1H)[37]
Alcohol: 4.91 ppm (q, J = 6.6 Hz, 1H)[38]

We can also identify five groups of doublets between 1.55 and 1.40 ppm belonging to the carboxylic acid (1.52 ppm, d, J = 6.6 Hz, 3H) and the esters (*syn*: 1.48 ppm, d, J = 7.2 Hz, 3H; 1.42 ppm, d, J = 6.6 Hz, 3H; *anti*: 1.50 ppm, d, J = 7.2 Hz, 3H; 1.49 ppm, d, J = 6.6 Hz, 3H).

<u>^{13}C NMR peaks for identification</u>
Carboxylic acid **13a**: 180.18, 139.91, 45.41, 18.27
syn Ester: 173.95, 141.75, 140.66, 72.73, 45.79, 22.11, 18.49
anti Ester: 173.76, 141.78, 140.54, 72.63, 45.87, 22.46, 18.44
Alcohol: 70.67

To discern the ester peaks, we followed the relative ordering of peaks from the literature[37]. Subsequently, we noticed that the easily discernable peaks were always taller for the *syn* ester than those for the *anti* ester, so for the more closely spaced peaks, we assigned the taller peak to the *syn* ester. There is also some contamination from maleic acid, which was intended to be used as an internal standard but was not soluble enough in $CDCl_3$ to be useful.

The GCMS spectrum shows three closely spaced peaks, the shortest of which is clearly identified as the carboxylic acid **5a**, while the other two larger ones are presumably the two ester enantiomers.

4-Methyl benzyl chloride

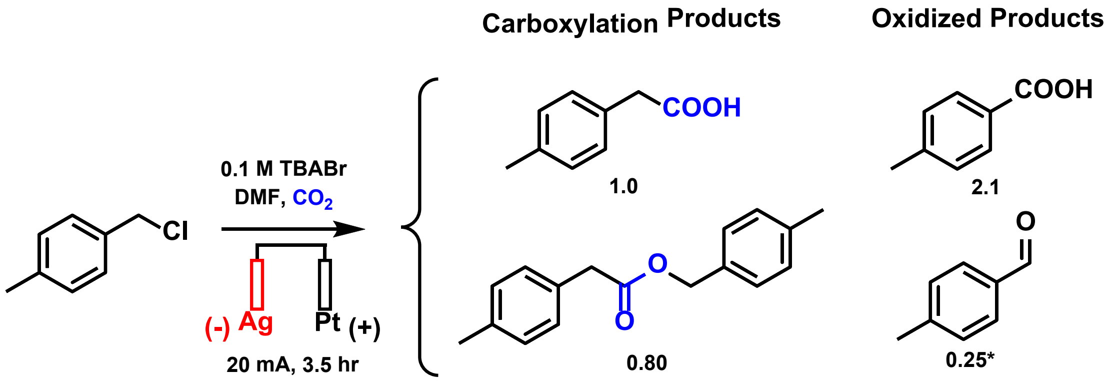

Following **Procedure C1**, 0.22 mmol TBABr, 2.2 mL DMF, and 29.7 mg 4-methyl benzyl chloride were combined and subjected to 20 mA for 3.5 hr under 20 sccm CO_2. The product was purified according to **Procedure C1**. Product ratios were determined by ^{1}H NMR and for the aldehyde, GCMS-FID (indicated by an *).

<u>^{1}H NMR peaks used for quantification</u>
Carboxylic acid + ester: singlets at 3.61 (ester, 2H) and 3.62 ppm (acid, 2H)[22]. Attempting to integrate each peak separately also gave good agreement with the ester peak at 5.08 ppm.
Ester: 5.08 ppm (s, 2H). Could not find ^{1}H NMR in literature.
Carboxylic acid: 7.97 ppm (d, J = 8.2 Hz, 2H)[39]
Aldehyde: 9.95 ppm (s, 1H)[40]

The aldehyde amount was estimated from GCMS-FID, since it appeared that a significant fraction had evaporated during workup before NMR.

^{13}C NMR peaks used for identification
Carboxylic acid **10a**: 178.08, 41.06
Carboxylic acid and ester: 172.19 and 171.96 (cannot easily assign these two)
Ester: 66.73, 40.76

There are also 4 prominent peaks from 20 – 21 ppm, corresponding to the benzylic methyl carbons (1 for each acid, 2 for the ester).

GCMS can identify the aldehyde and both carboxylic acids. There is a large peak at 15.7 min that is likely the ester. The FID area for the aldehyde on the GCMS relative to carboxylic acid **10a** is about an order of magnitude larger than what is found on the ^{1}H NMR, suggesting a lot of it evaporated during rotary evaporation and vacuum drying.

All three characterization methods indicate the presence of smaller amounts of several side products, likely due to oxidation.

Bromobenzene

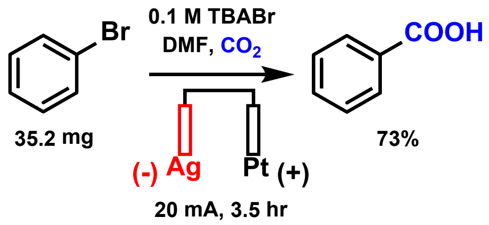

Following **Procedure C1**, 0.22 mmol TBABr, 2.2 mL DMF, and 35.2 mg bromobenzene were combined and subjected to 20 mA for 3.5 hr under 20 sccm CO_2. The product was purified according to **Procedure C1** to give **15a** in 73% yield by ^{1}H NMR with 24.6 mg of 1,3,5-trimethoxybenzene as the internal standard.

^{1}H NMR (500 MHz, $CDCl_3$) δ 9.29 (br, s), 8.15 – 8.09 (m, 2H), 7.61 (m, 1H), 7.52 – 7.45 (m, 2H)

^{13}C NMR (126 MHz, $CDCl_3$) δ 171.93, 133.83, 130.30, 129.54, 128.58.

11.5 Reactions Which Had Cell Leakage

For several substrates, the compression sandwich cells that were normally used leaked through the O-ring seals at both the cathode and anode. Additionally, this leakage was also often accompanied by some foaming out through the top of the cell. An electrolyte containing 0.1 M 1-bromohexane was allowed to sit in the cell for 1.5 hr, and similar leakage was observed as when passing current. This observation suggests the substrates, rather than their products, are responsible for the leakage. The chemical incompatibility likely arises from the PEEK cell material, as the O-rings are made of more chemically resistant FEP, and the metal electrodes should not be degraded, although there was not any visible damage to the PEEK after the experiments. A sandwich cell made of more chemically resistant material such as Teflon or a glass cell could possibly alleviate this issue.

We did attempt to carboxylate 1-bromohexane in a glass vial cell, but difficulties with our setup compromised a consistently wide enough anode-cathode distance. This distance is believed to be crucial to prevent too much anodically generated bromine from reducing at the cathode. We were able to obtain a 33% yield of heptanoic acid in a glass vial cell with 6 mL of 0.1 M 1-bromohexane electrolyte, but this yield is probably lower than what is inherently achievable by the cathodic chemistry. Since carboxylating these specific substrates was not crucial for our conclusions, we decided to not improve their yields further at this time but do want to note that in principle, they can be carboxylated electrochemically without a sacrificial anode.

5-Fluoropentanoic acid[41]

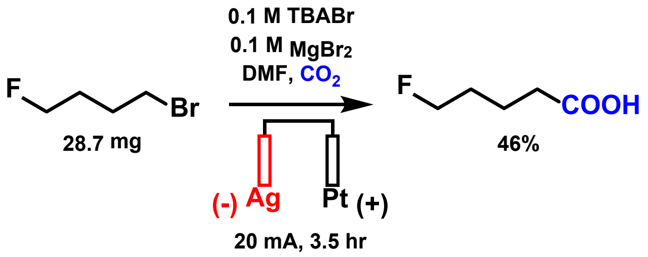

Following **Procedure B**, 0.22 mmol TBABr, 0.22 mmol $MgBr_2$, 2.2 mL DMF, and 28.7 mg 1-bromo-4-fluorobutane were combined and subjected to 20 mA for 3.5 hr under 20 sccm CO_2. The product was purified according to **Procedure B** to give 5-fluoropentanoic acid in 46% yield by ^{1}H NMR with 16.6 mg 1,3,5-trimethoxybenzene as the internal standard. Noticeable cell leakage occurred, so the observed yield is likely lower than the true yield.

^{1}H NMR (500 MHz, $CDCl_3$) δ 4.55 – 4.48 (m, 1H), 4.42 (t, J = 5.65 Hz, 1H), 2.49 – 2.38 (m, 2H), 1.84 – 1.70 (m, 3H).

^{13}C NMR (126 MHz, $CDCl_3$) δ 179.56, 83.55 (d, J = 165.0 Hz), 33.52, 29.66 (d, J = 19.9 Hz), 20.59 (d, J = 5.3 Hz).

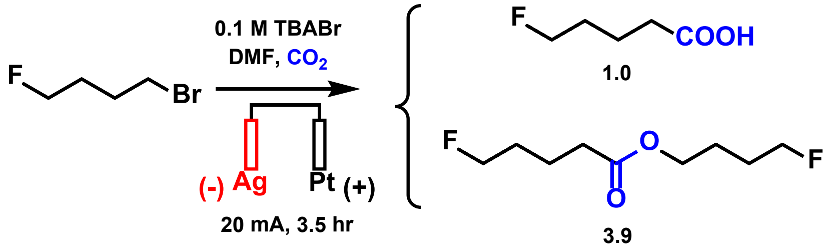

Following **Procedure C1**, 0.22 mmol TBABr, 2.2 mL DMF, and 30.5 mg 1-bromo-4-fluorobutane were combined and subjected to 20 mA for 3.5 hr under 20 sccm CO_2. The product was purified according to **Procedure C1**. Product ratios were determined from ^{1}H NMR. 1,3,5-Trimethoxybenzene was added as an internal standard but was not used for quantification due to electrolyte leakage. N-methyl pyrrolidinone was present as an impurity from vacuum drying.

^{1}H NMR peaks for quantification

Ester: 4.12 ppm (m, 2H) (Note that there is no literature spectra for the ester)

Carboxylic acid + ester: triplets from 2.34 to 2.44 ppm (2H for both). Subtracted NMP

contribution.
NMP: 3.40 ppm (m, 2H)

For consistency, the multiplets from 4.55 – 4.47, 4.44 – 4.39 and 1.89 – 1.65 ppm, which reflect the total amount of acid and ester, were also integrated and found to agree to within 10% with the results for the quantification peaks listed above.

^{13}C NMR peaks for identification
Carboxylic acid: 177.45, 83.69 (d, J = 165.0 Hz), 33.31, 29.79 (d, J = 20.2 Hz), 20.75 (d, J = 5.3 Hz)
Ester: 173.42, 83.71 (d, J = 165.0 Hz), 83.60 (d, J = 165.0 Hz), 63.96, 33.81, 29.88 (d, J = 19.7 Hz), 27.17 (d, J = 20.1 Hz), 24.79 (d, J = 5.1 Hz), 20.97 (d, J = 5.3 Hz).

^{13}C NMR can account for all carbon atoms in the ester and acid.

GCMS was performed on the products, but identification proved difficult because neither product has a reference spectrum. We were unable to detect any formation of any δ-valerolactone.

Heptanoic acid[42]

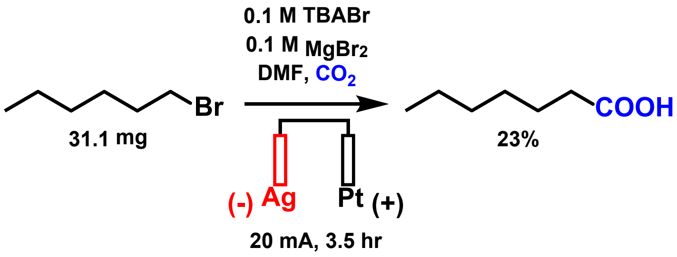

Following **Procedure B**, 0.22 mmol TBABr, 0.22 mmol $MgBr_2$, 2.2 mL DMF, and 31.1 mg 1-bromohexane were combined and subjected to 20 mA for 3.5 hr under 20 sccm CO_2. The product was purified according to **Procedure B** to give heptanoic acid in 23% yield by ^{1}H NMR with 6.0 mg 1,3,5-trimethoxybenzene as the internal standard. Noticeable cell leakage occurred, so the observed yield is likely lower than the true yield.

^{1}H NMR (500 MHz, $CDCl_3$) δ 2.35 (t, J = 7.53 Hz, 2H), 1.69 – 1.59 (m, 2H), 1.40 – 1.24 (m, 6H), 0.91 – 0.87 (m, 3H).

^{13}C NMR (126 MHz, $CDCl_3$) δ 180.09, 34.15, 31.56, 28.86, 24.77, 22.60, 14.15.

2-(4-Trifluoromethyl)phenylacetic acid[22]

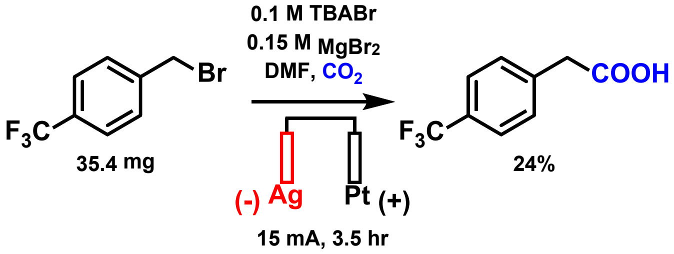

Following **Procedure B**, 0.22 mmol TBABr, 0.33 mmol $MgBr_2$, 2.2 mL DMF, and 35.4 mg 4-trifluoromethyl benzyl bromide were combined and subjected to 15 mA for 3.5 hr under 20 sccm CO_2. The product was purified according to **Procedure B** to give 2-(4-trifluoromethyl)phenylacetic acid in 24% yield by ^{1}H NMR with 7.0 mg ethylene carbonate as the internal standard. Noticeable cell leakage occurred, so the observed yield is likely lower than the true yield.

^{1}H NMR (500 MHz, $CDCl_3$) δ 7.60 (d, J = 8.05 Hz, 2H), 7.41 (d, J = 7.99 Hz, 2H), 3.73 (s, 2H).

^{13}C NMR (126 MHz, $CDCl_3$) δ 176.47, 137.27, 129.94, 126.69 (q, J = 3.85 Hz), 123.10, 40.76

12 Voltage-Time Traces for Non-Sacrificial and Sacrificial-Anode Syntheses

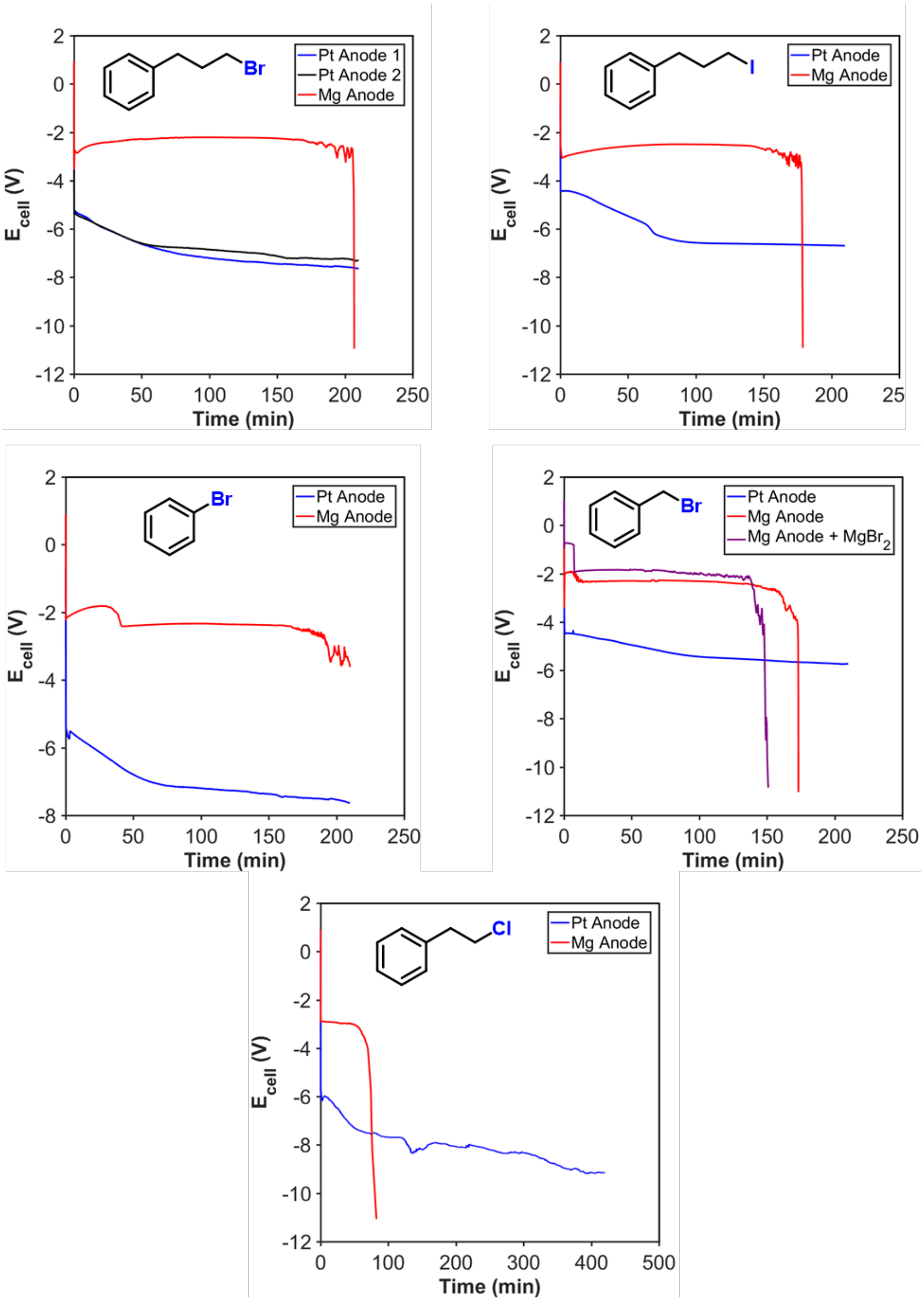

Figure S24. Cell voltage-time traces for carboxylation of several substrates with and without sacrificial anodes. For 1-bromo-3-phenylpropane, the non-sacrificial anode experiment was performed in duplicate. For benzyl bromide, an additional experiment with both 0.1 M $MgBr_2$ and an Mg anode was done. Except for bromobenzene, the sacrificial anode syntheses terminated early after a precipitous increase in cell voltage.

13 NMR and Mass Spectra

13.1 Reactions with $MgBr_2$ and a Non-Sacrificial Anode

4-Phenylbutyric acid (1a)

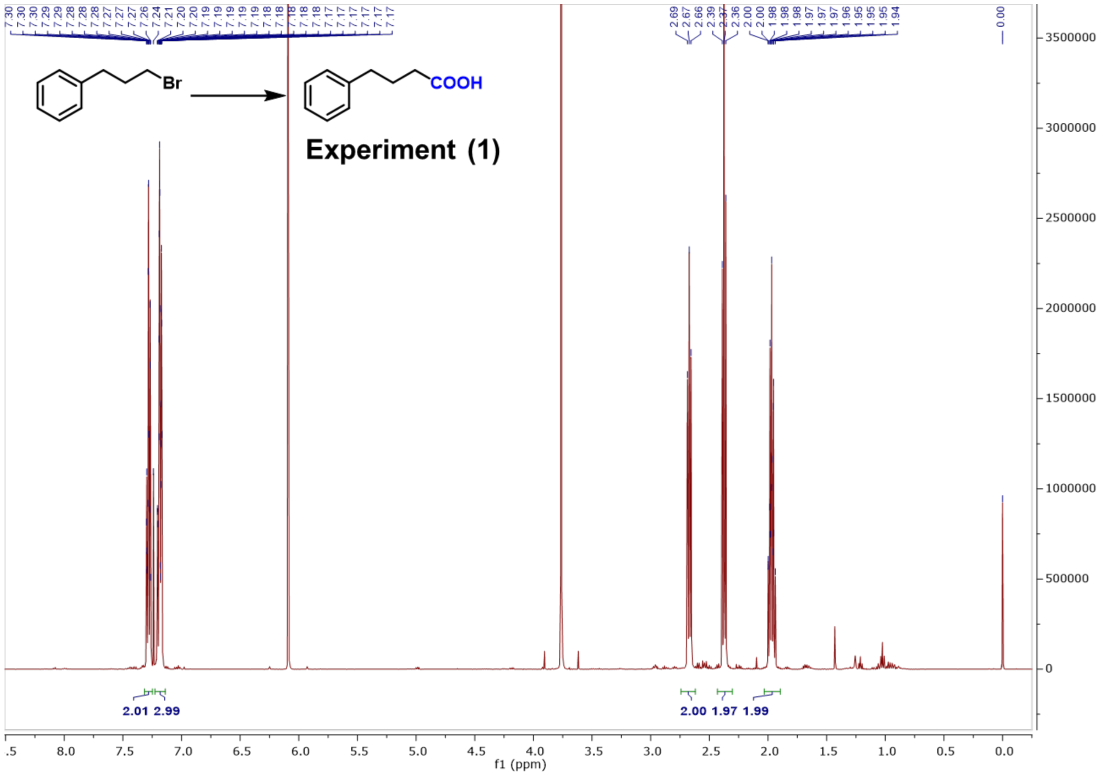

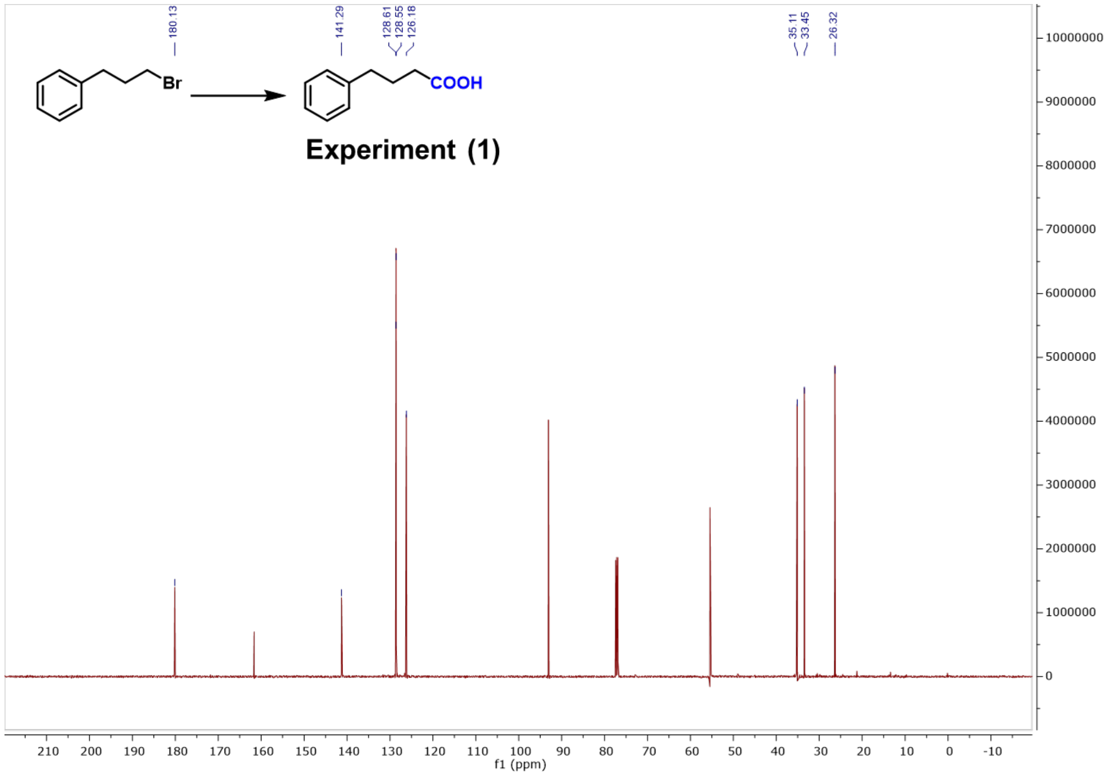

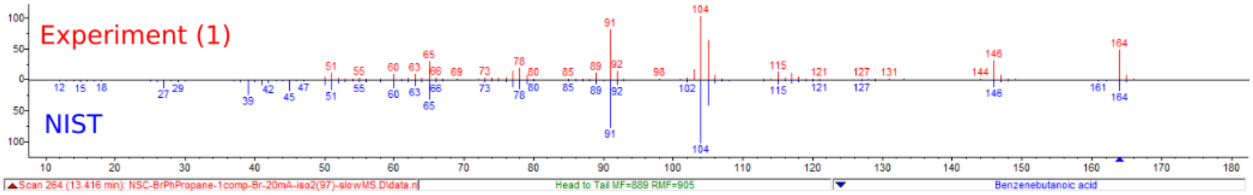
Experiment (1)
NIST
Scan 264 (13.416 min): NSC-BrPhPropane-1comp-Br-20mA-iso2(97)-slowMS.D\data.n
Head to Tail MF=889 RMF=905
Benzenebutanoic acid

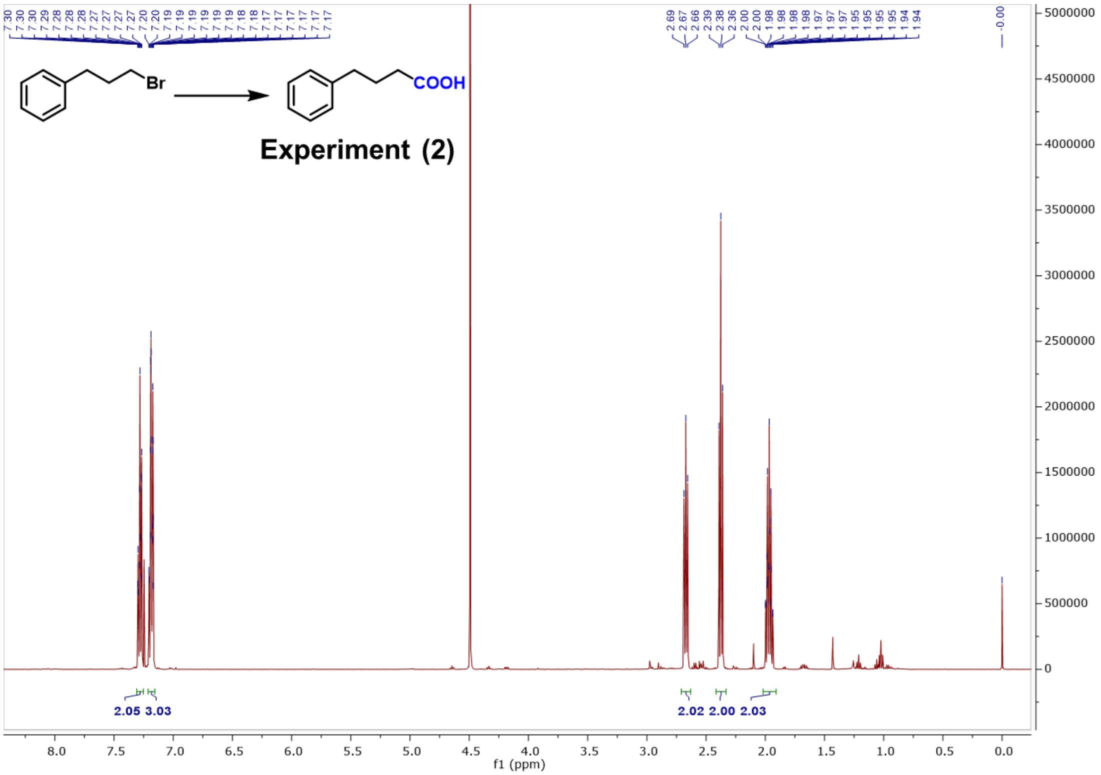
Br
COOH
Experiment (2)
f1 (ppm)
2.05 3.03
2.02 2.00 2.03

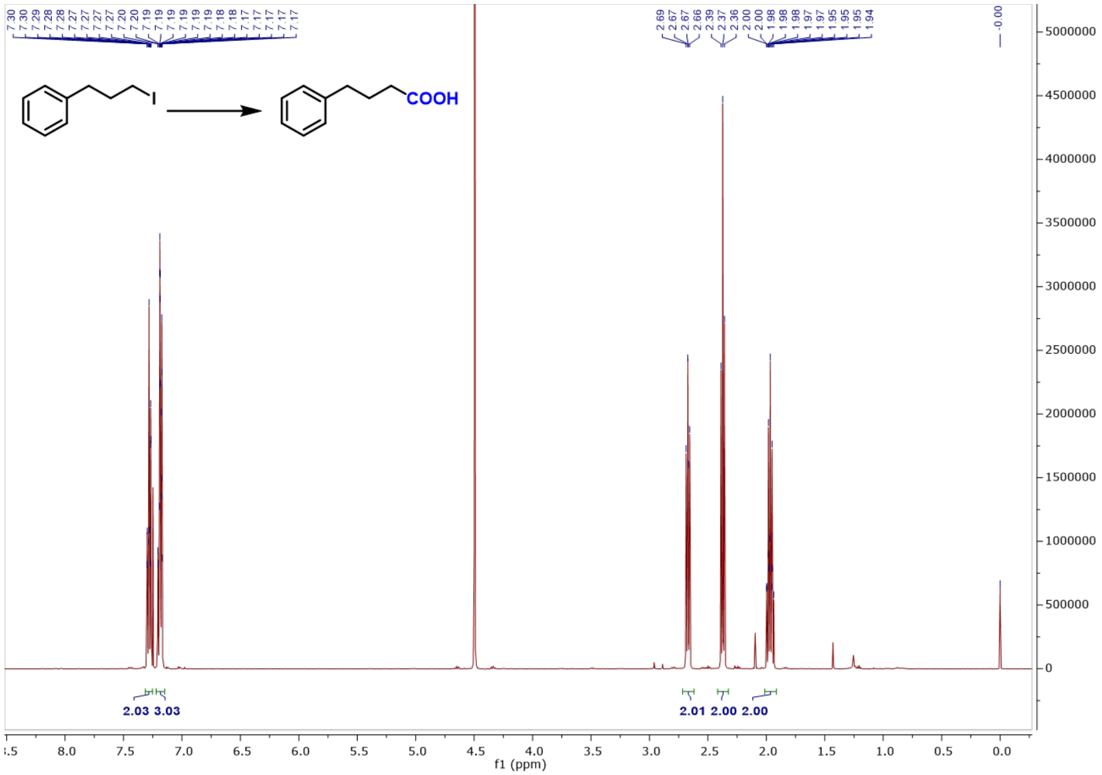
COOH
I
2.03 3.03
2.01 2.00 2.00
f1 (ppm)

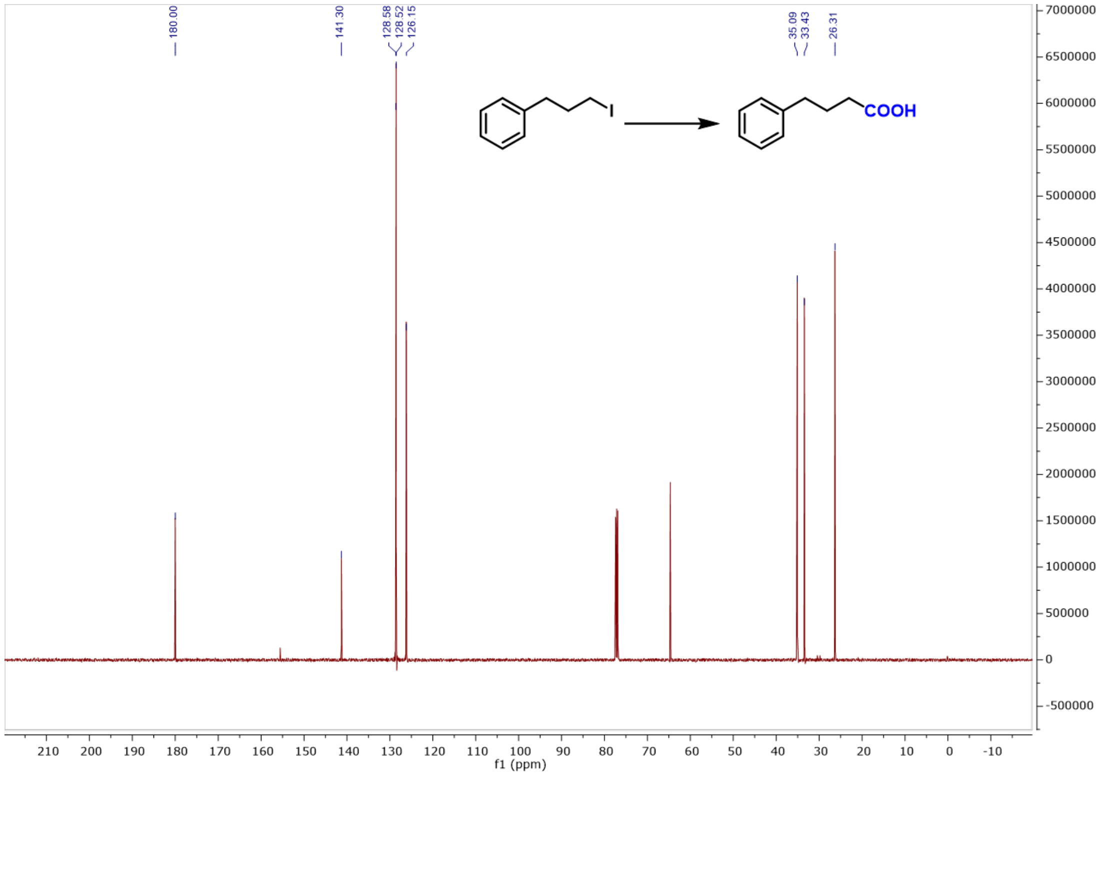
180.00
141.30
128.58
128.52
126.15
35.09
33.43
26.31
I
COOH
f1 (ppm)

3-Phenylpropanoic acid (2a)

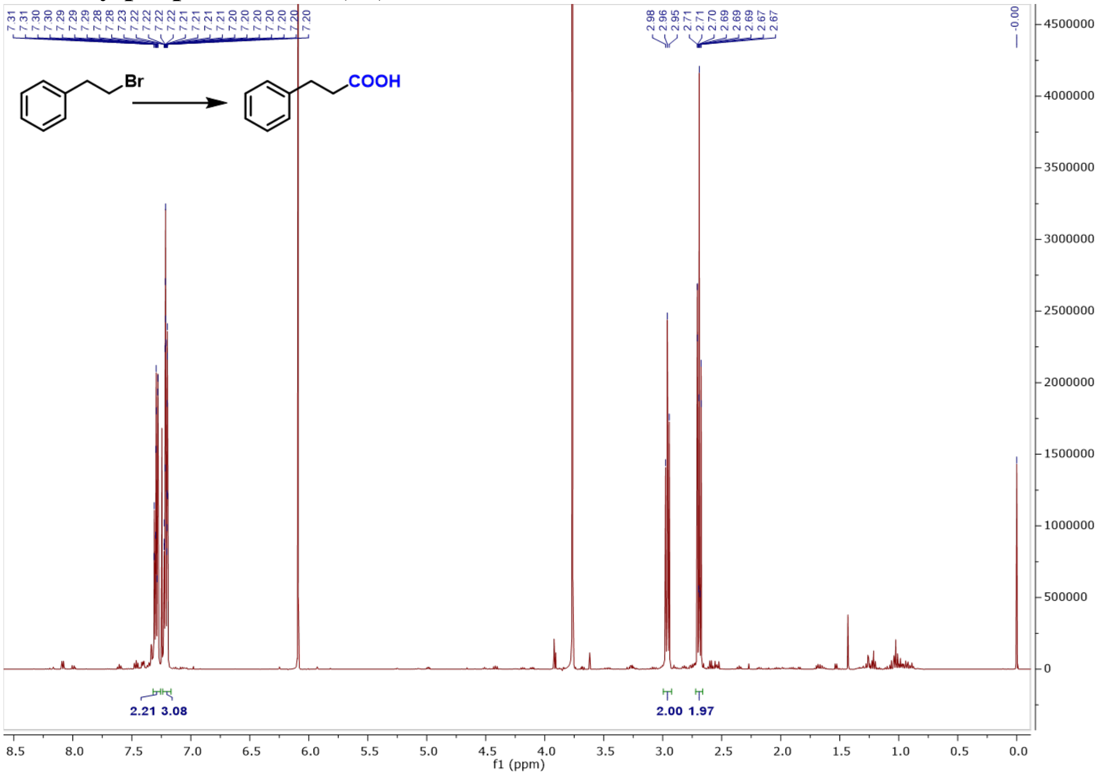

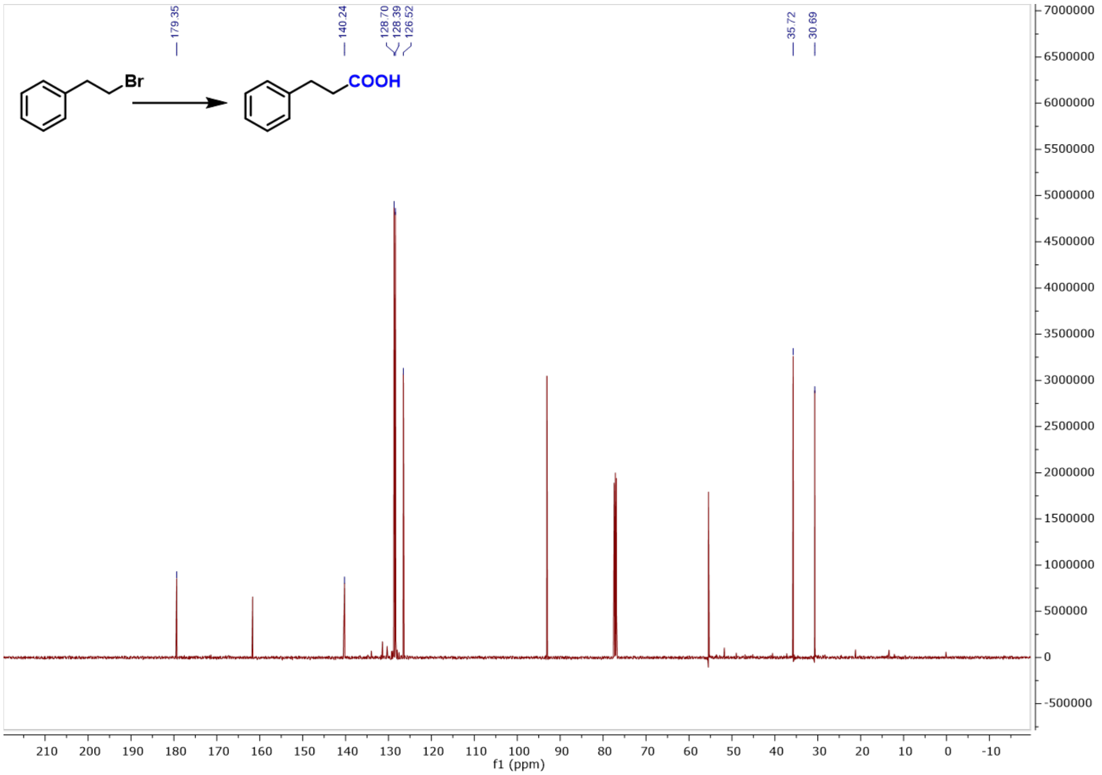

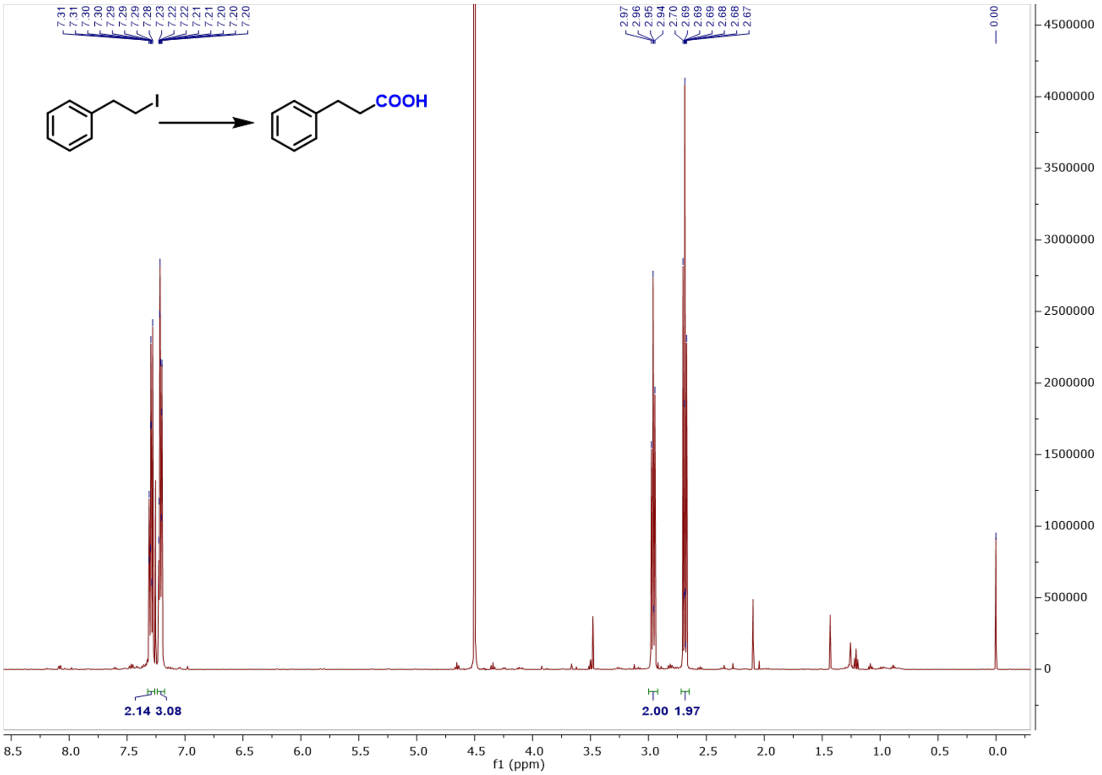
I
COOH
7.31
7.31
7.30
7.30
7.29
7.29
7.29
7.28
7.23
7.22
7.22
7.21
7.21
7.20
7.20
7.20
2.97
2.96
2.95
2.94
2.70
2.69
2.69
2.69
2.68
2.68
2.67
0.00
2.14 3.08
2.00 1.97
f1 (ppm)

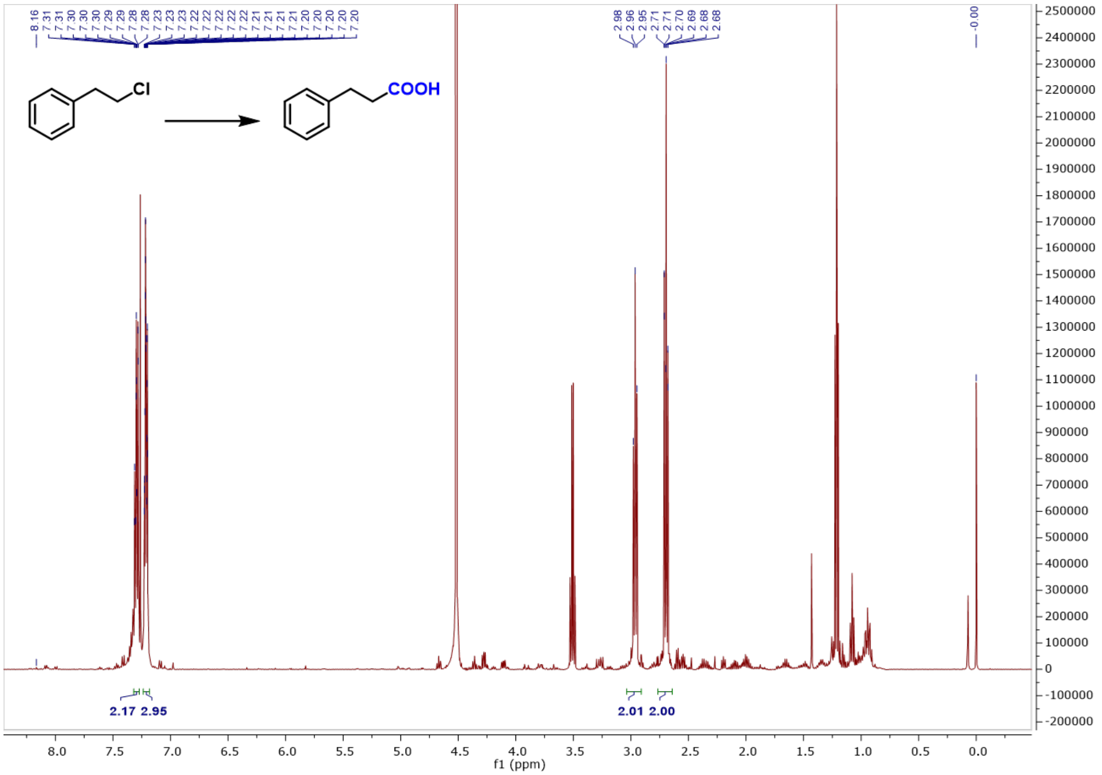
Cl
COOH
8.16
0.00
2.17 2.95
2.01 2.00
f1 (ppm)

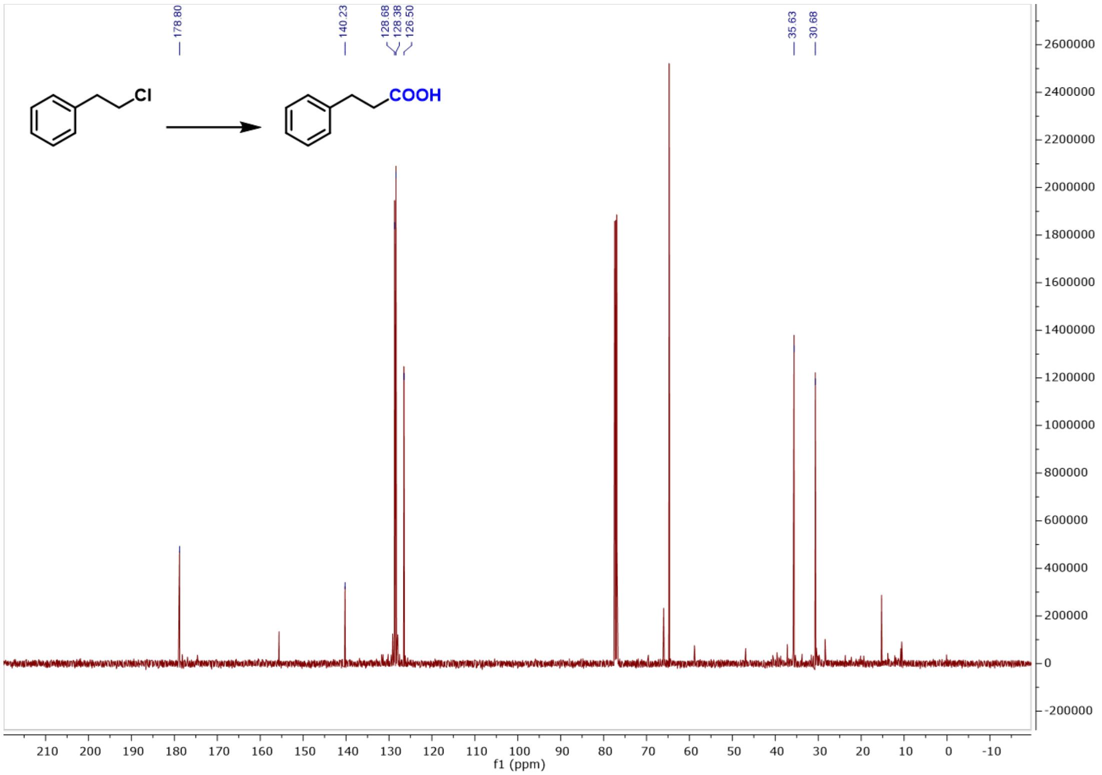

178.80
140.23
128.68
128.38
126.50
35.63
30.68
Cl
COOH
2600000
2400000
2200000
2000000
1800000
1600000
1400000
1200000
1000000
800000
600000
400000
200000
0
-200000
210 200 190 180 170 160 150 140 130 120 110 100 90 80 70 60 50 40 30 20 10 0 -10
f1 (ppm)

4-(Benzyloxy)butanoic acid (3a)

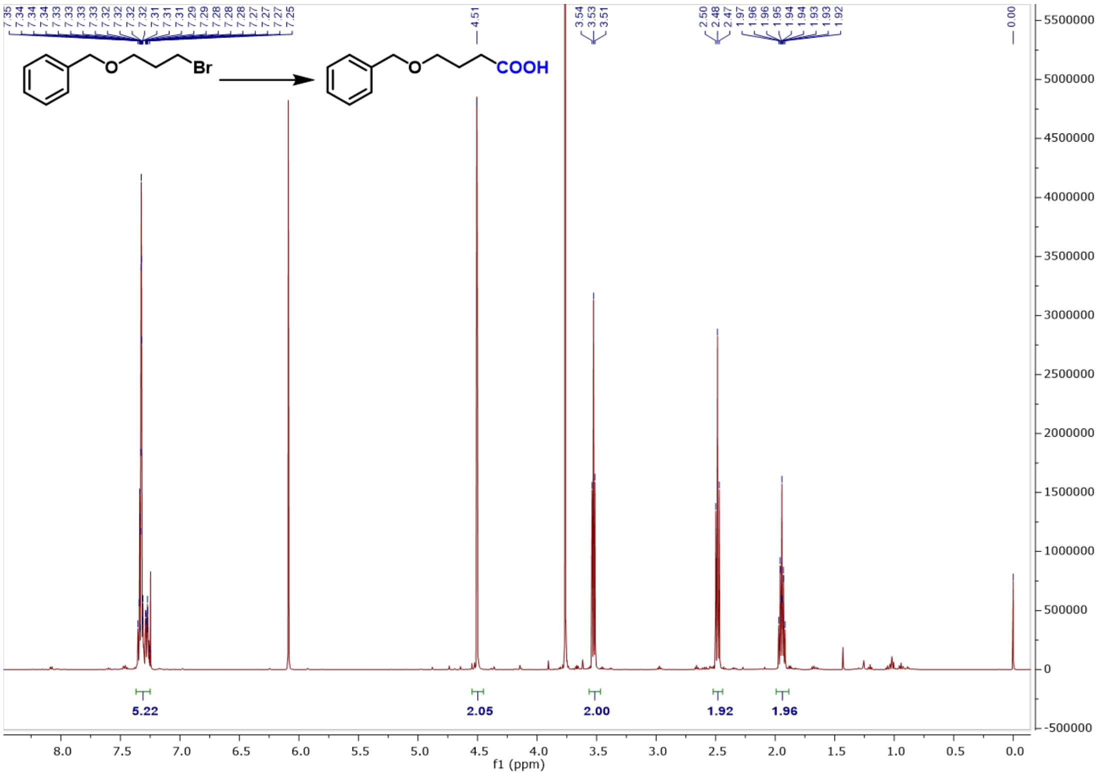

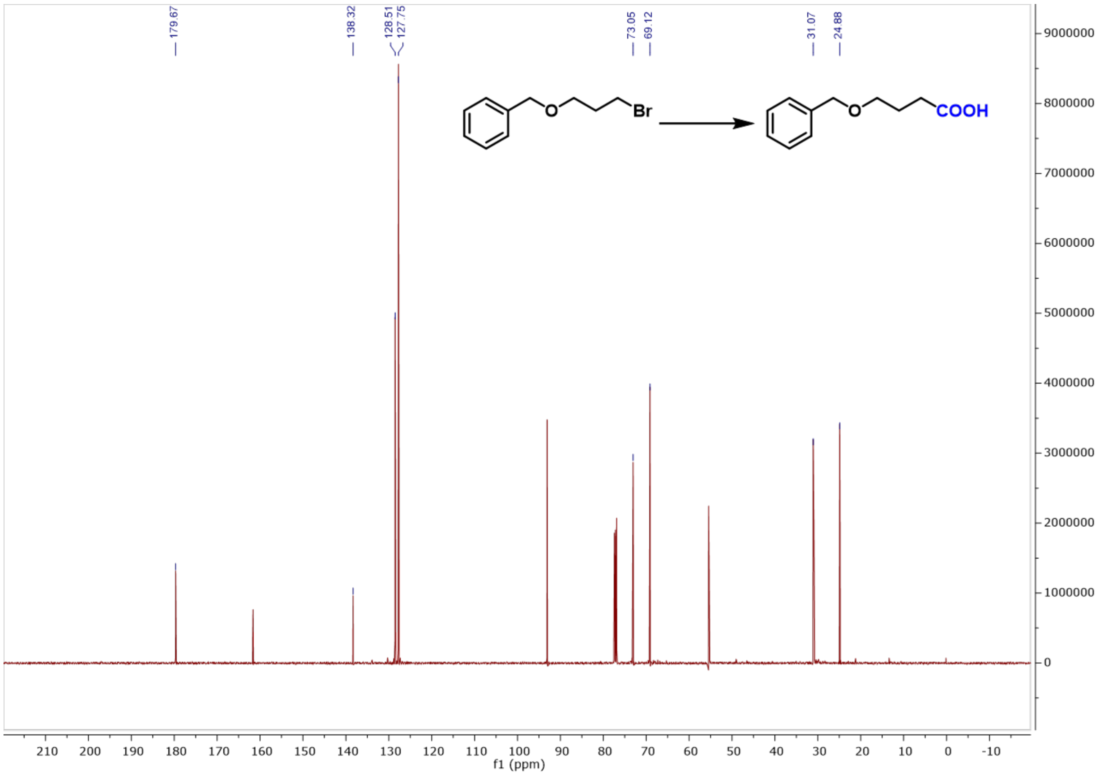

6-Chlorohexanoic acid (4a)

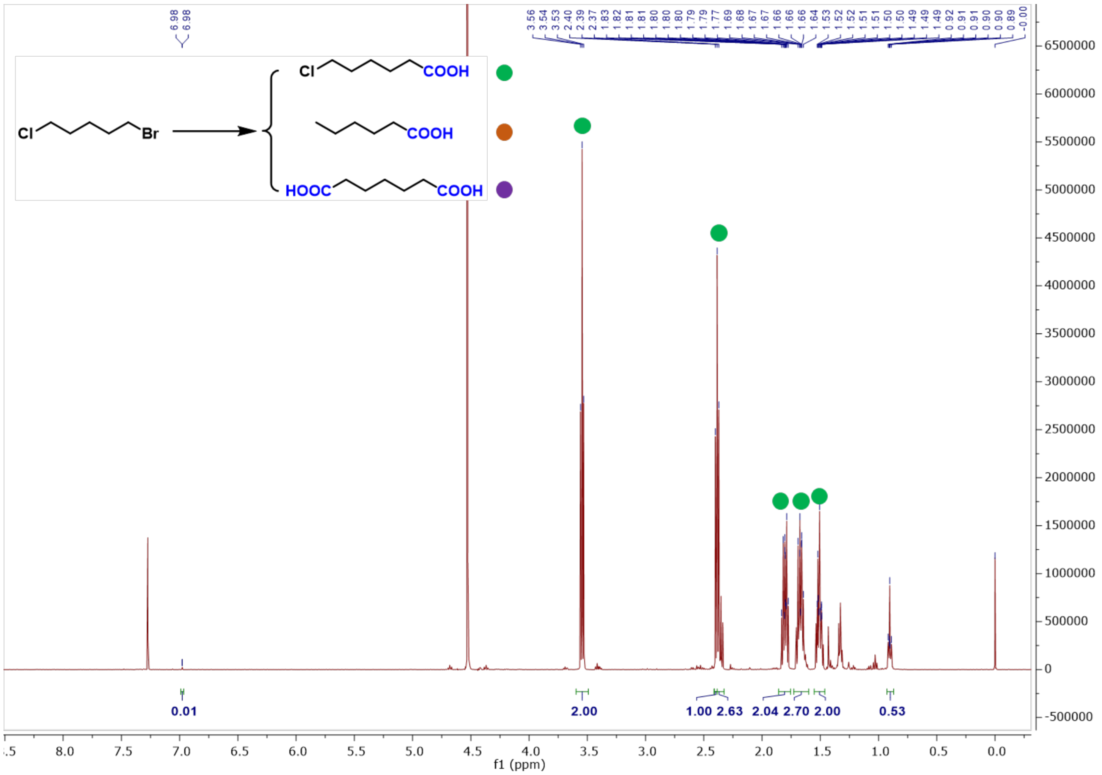

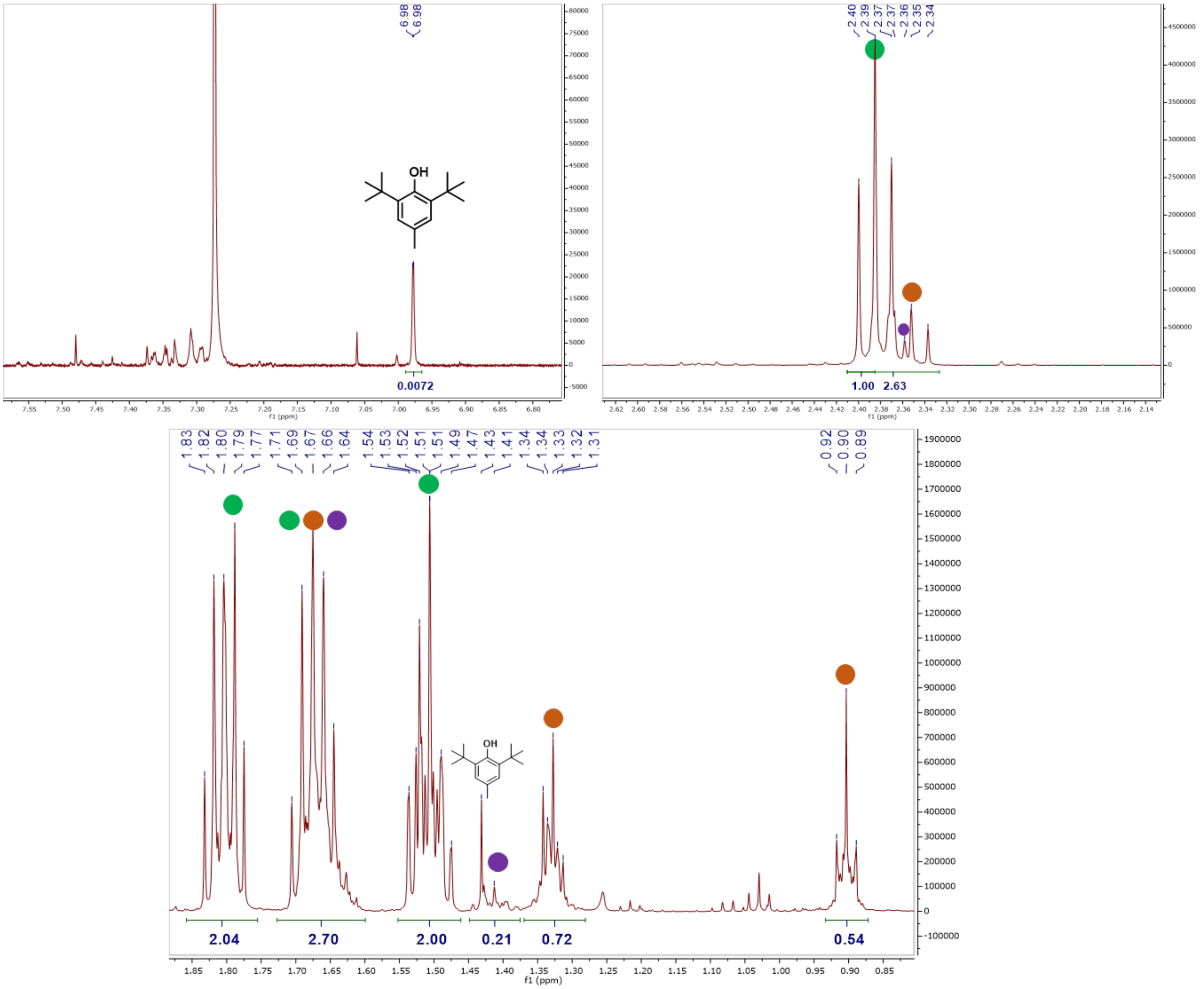

Table S10. Deconvolution of the peak areas from the ^{1}H NMR spectra of the reaction products from carboxylation of 1-bromo-5-chloropentane. The blue entries establish normalized areas for each compound, and the yellow entries verify these areas are consistent across all other observable peaks.

Peaks	4a	Hexanoic Acid	1,7-Heptanedioic Acid	BHT	Total	Actual Peak Area
3.54, t	2.00	-	-	-	2.00	2.00
0.90, m	-	0.54	-	-	0.54	0.54
6.98, s	-	-	-	0.0072	0.0072	0.0072
2.40 – 2.34	2.00	0.36	0.27	-	2.63	2.63
1.83 – 1.77	2.00	-	-	-	2.00	2.04

1.71 – 1.61	2.00	0.36	0.27	-	2.63	2.70
1.54 – 1.47	2.00	-	-	-	2.00	2.00
1.45 – 1.38	-	-	0.135	0.065	0.200	0.207
1.36 – 1.29	-	0.72	-	-	0.72	0.72

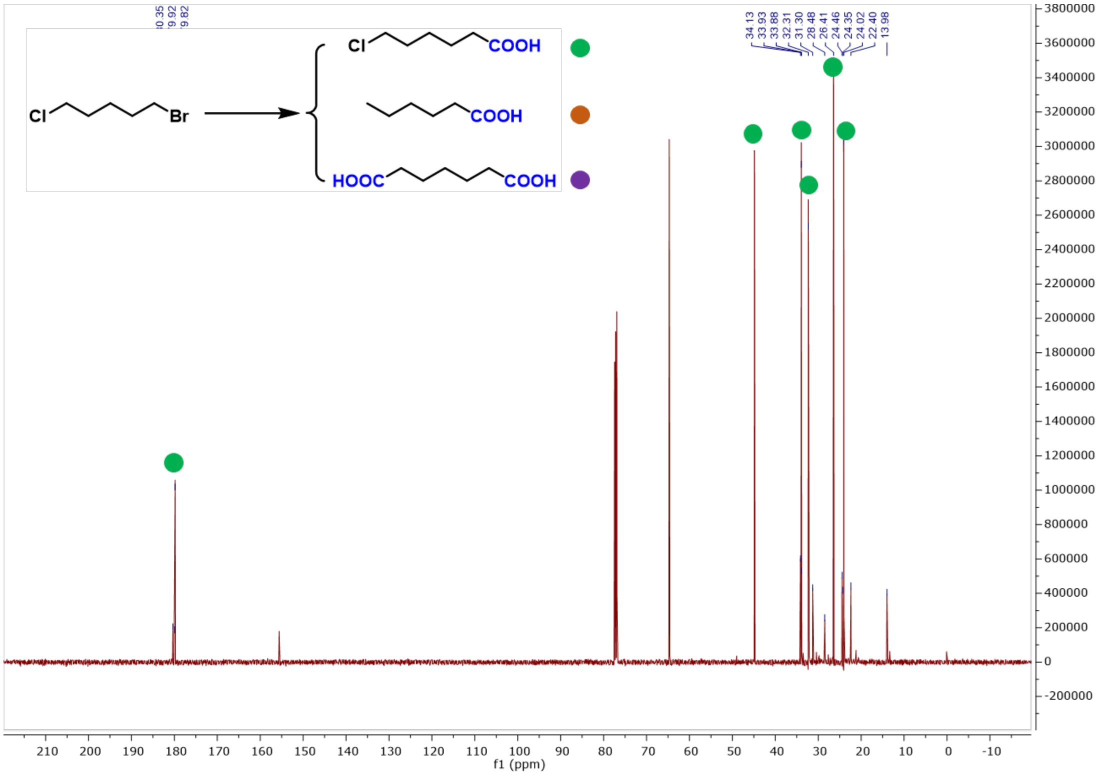

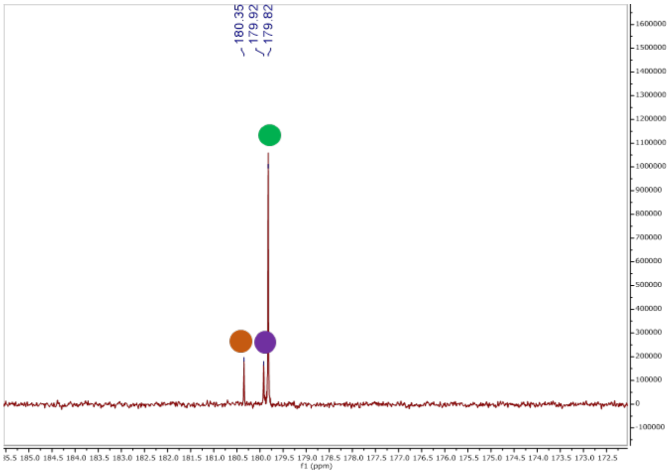
180.35
179.92
179.82
f1 (ppm)

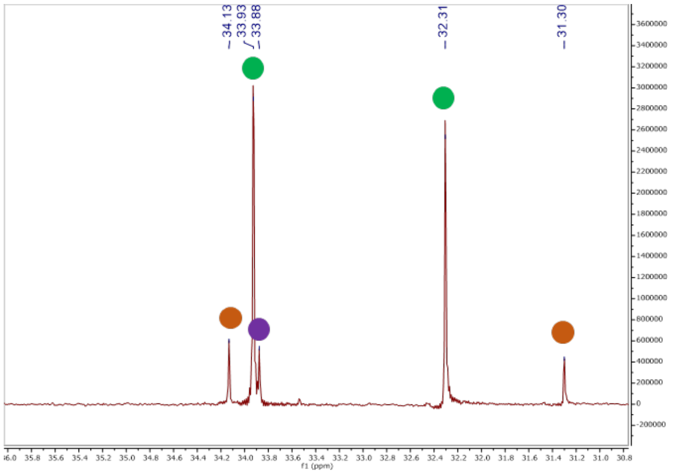
34.13
33.93
33.88
32.31
31.30
f1 (ppm)

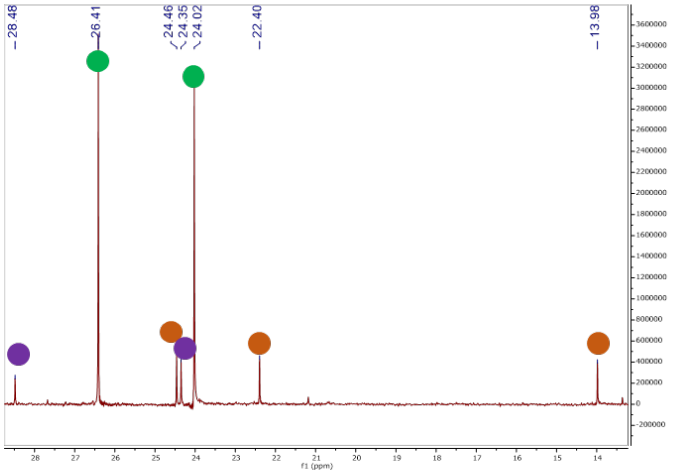
28.48
26.41
24.46
24.35
24.02
22.40
13.98
f1 (ppm)

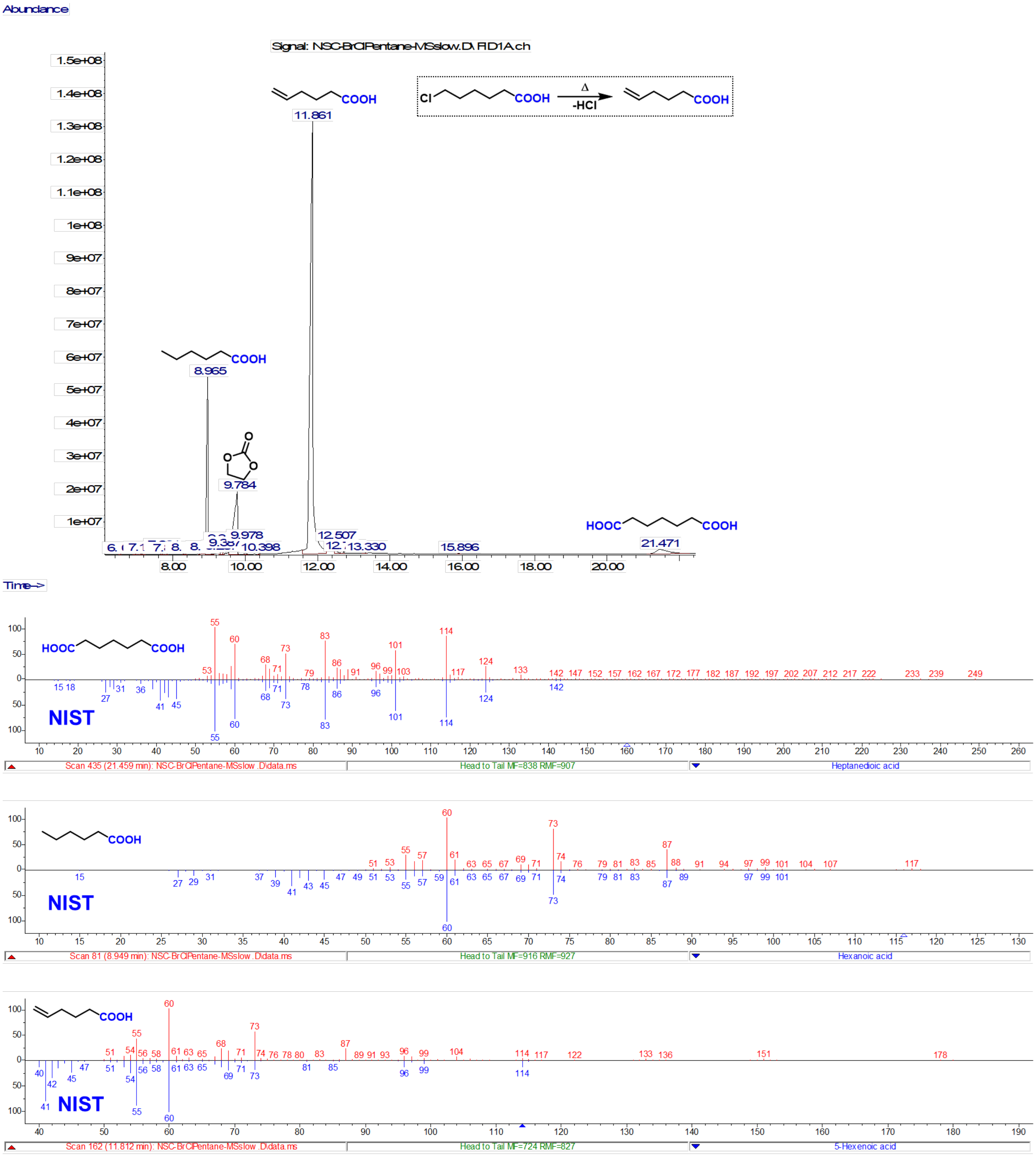

Abundance
Signal: NSC-BrClPentane-MSslow.D\ FID1A.ch
COOH
11.861
Cl
COOH
Δ
-HCl
COOH
COOH
8.965
9.784
9.978
10.398
12.507
13.330
15.896
HOOC
COOH
21.471
Time-->
HOOC
COOH
NIST
Scan 435 (21.459 min): NSC-BrClPentane-MSslow .D\data.ms
Head to Tail MF=838 RMF=907
Heptanedioic acid
COOH
NIST
Scan 81 (8.949 min): NSC-BrClPentane-MSslow .D\data.ms
Head to Tail MF=916 RMF=927
Hexanoic acid
COOH
NIST
Scan 162 (11.812 min): NSC-BrClPentane-MSslow .D\data.ms
Head to Tail MF=724 RMF=827
5-Hexenoic acid

Pentanoic acid (19a)

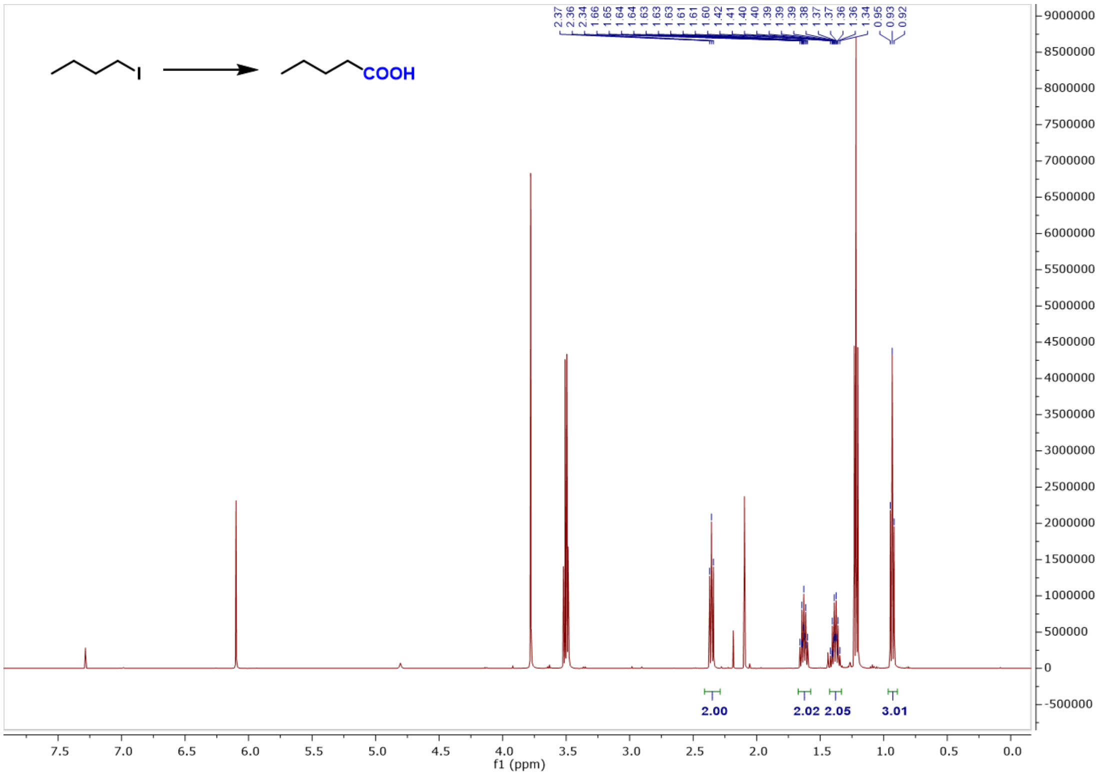

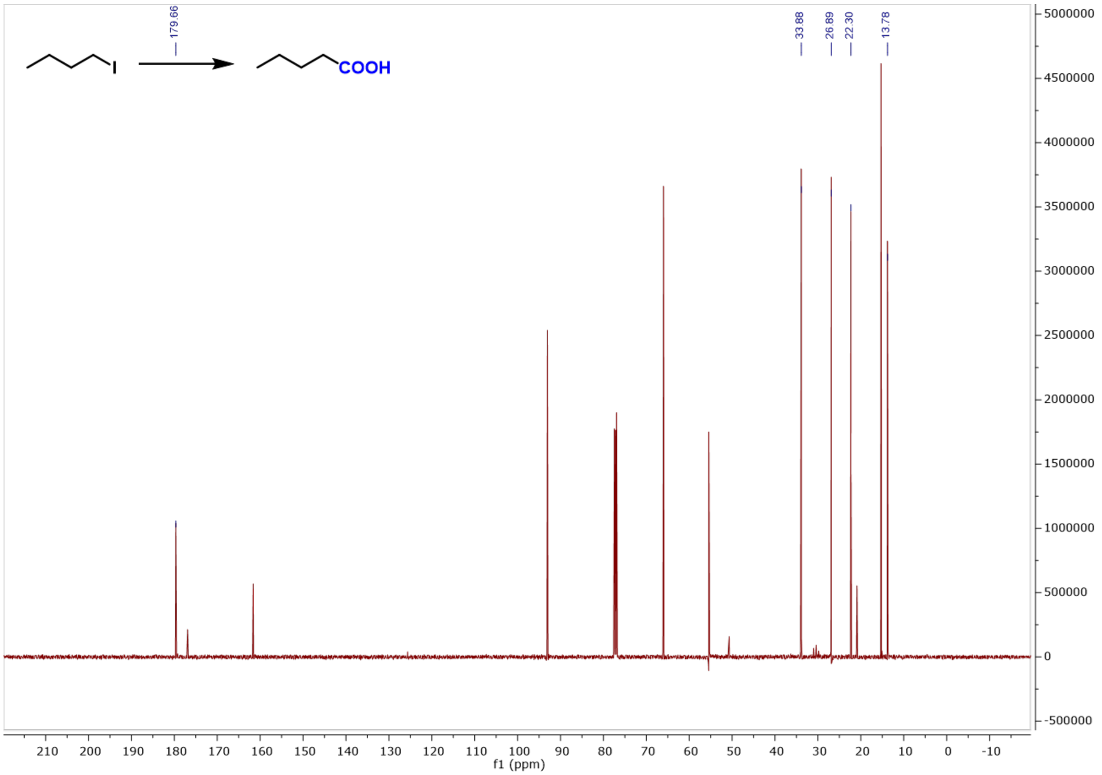

Cyclohexanecarboxylic acid (5a)

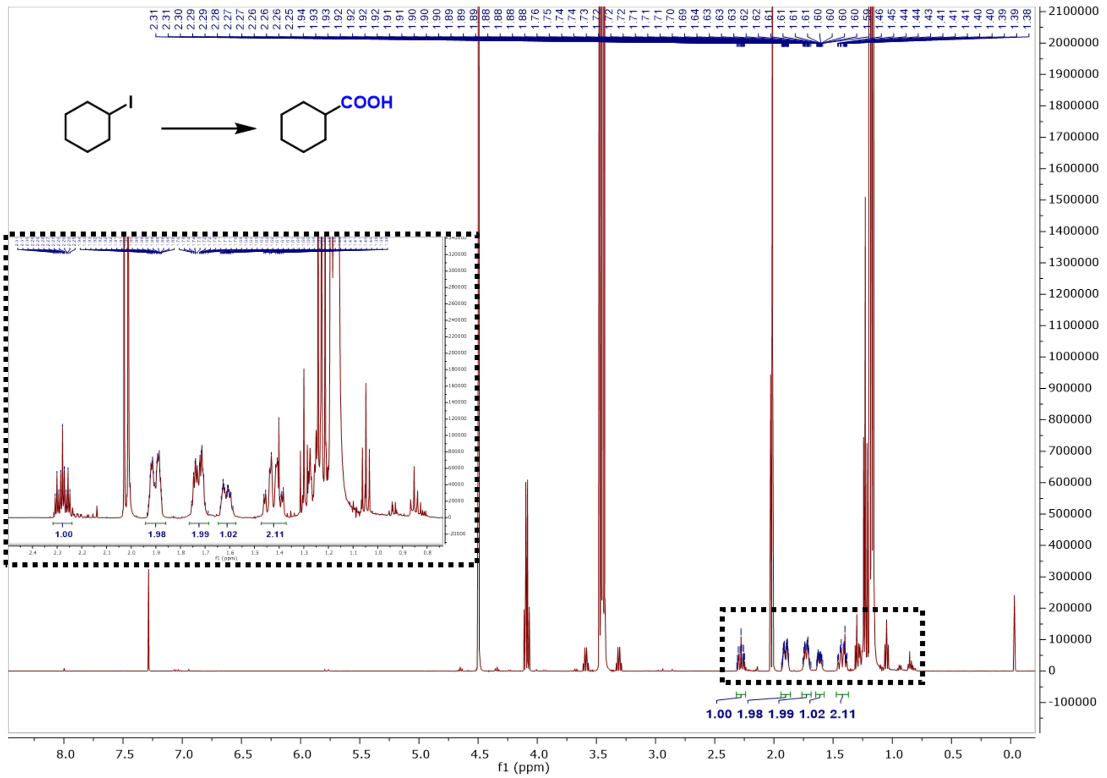

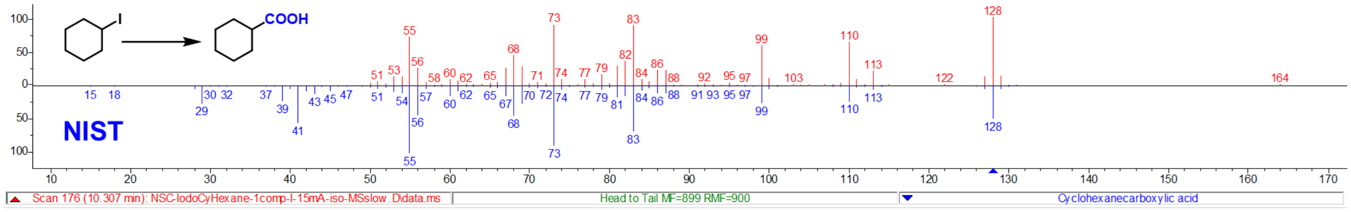

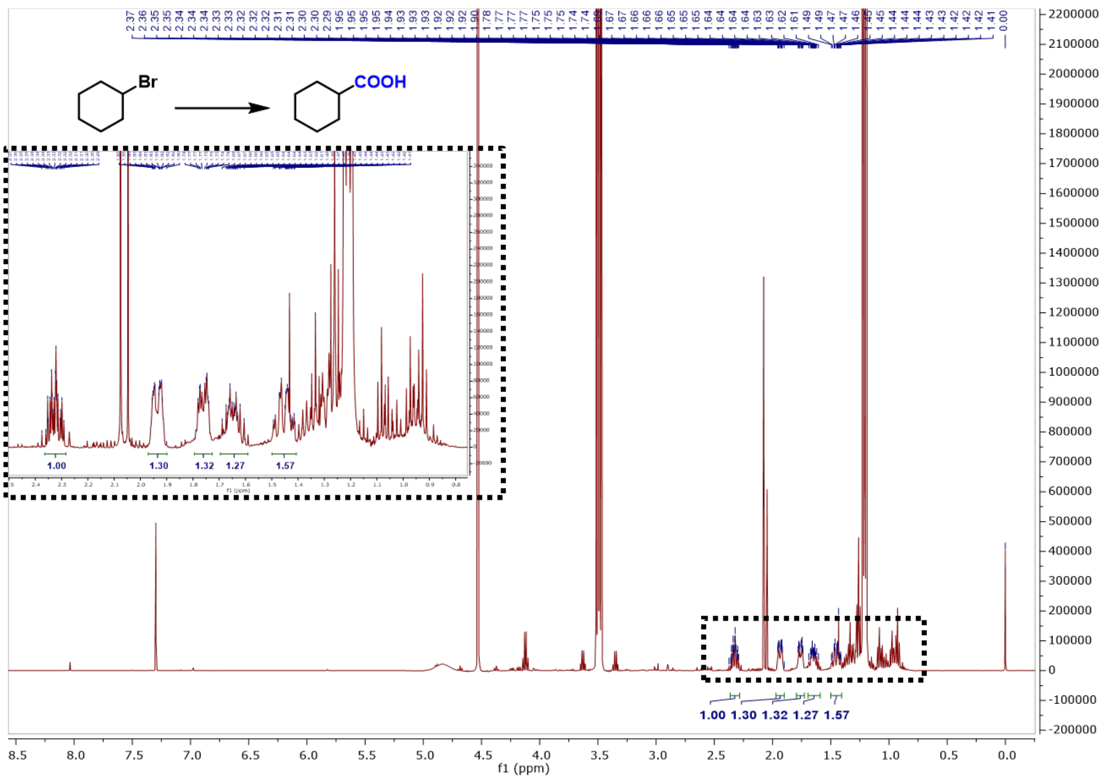

Br
COOH
1.00 1.30 1.32 1.27 1.57
f1 (ppm)

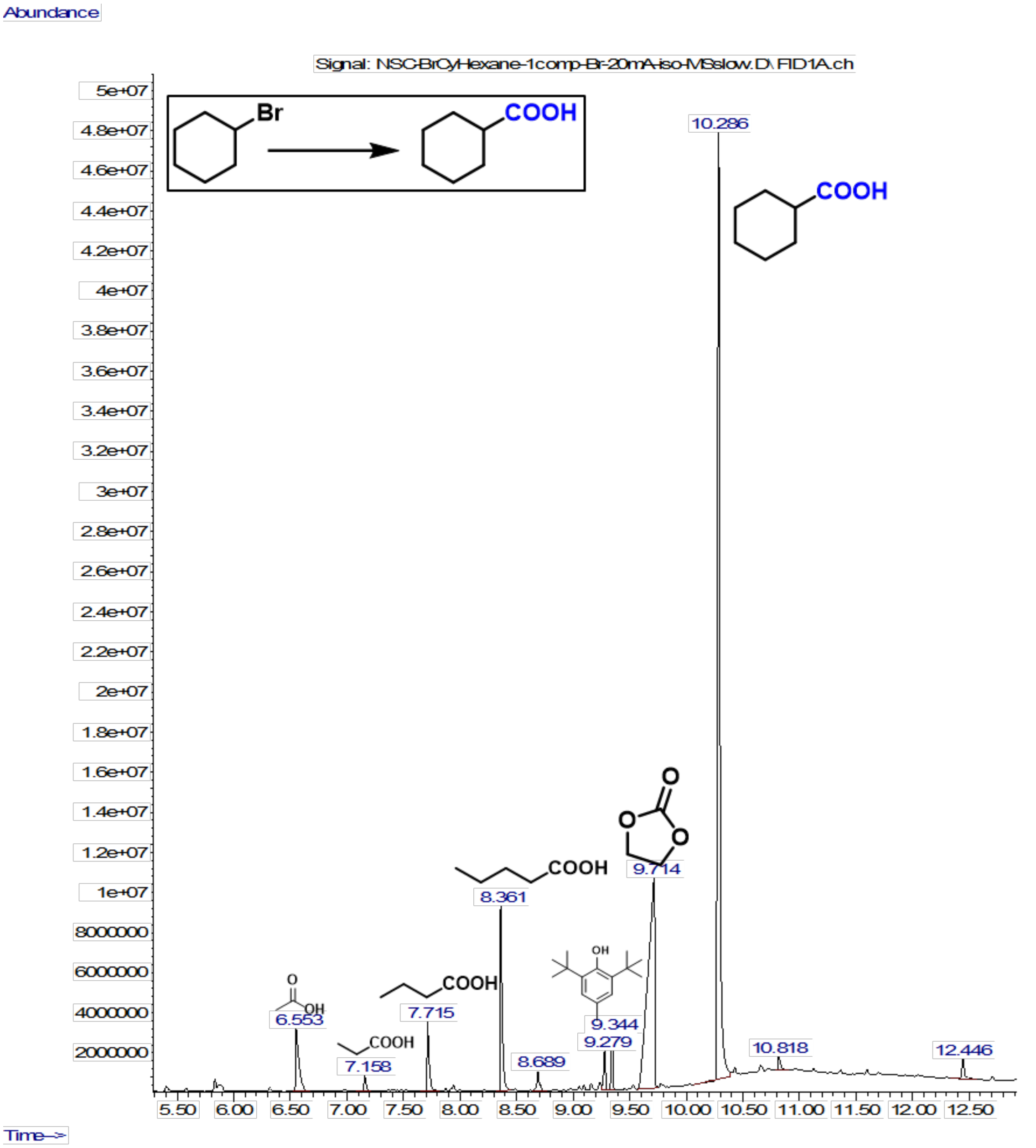
Abundance
Signal: NSC-BrCyHexane-1comp-Br-20mA-iso-MSslow.D\ FID1A.ch
Br
COOH
10.286
COOH
8.361
COOH
9.714
7.715
COOH
6.553
OH
9.344
9.279
COOH
7.158
8.689
10.818
12.446
Time-->

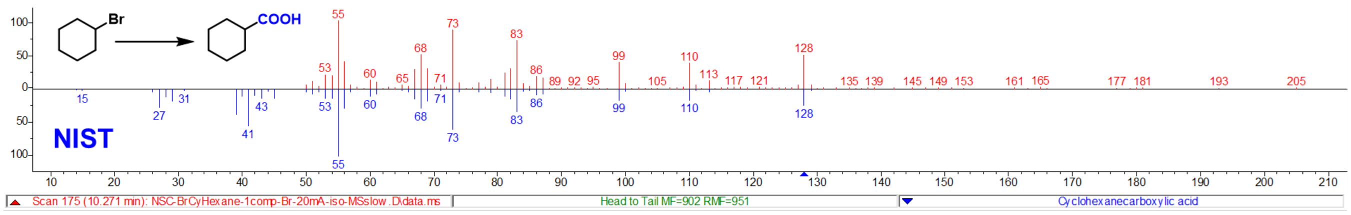
Br
COOH
NIST
Scan 175 (10.271 min): NSC-BrCyHexane-1comp-Br-20mA-iso-MSslow.D\data.ms
Head to Tail MF=902 RMF=951
Cyclohexanecarboxylic acid

2-Methyl-3-phenylpropanoic acid (6a) and 2-phenylbutyric acid

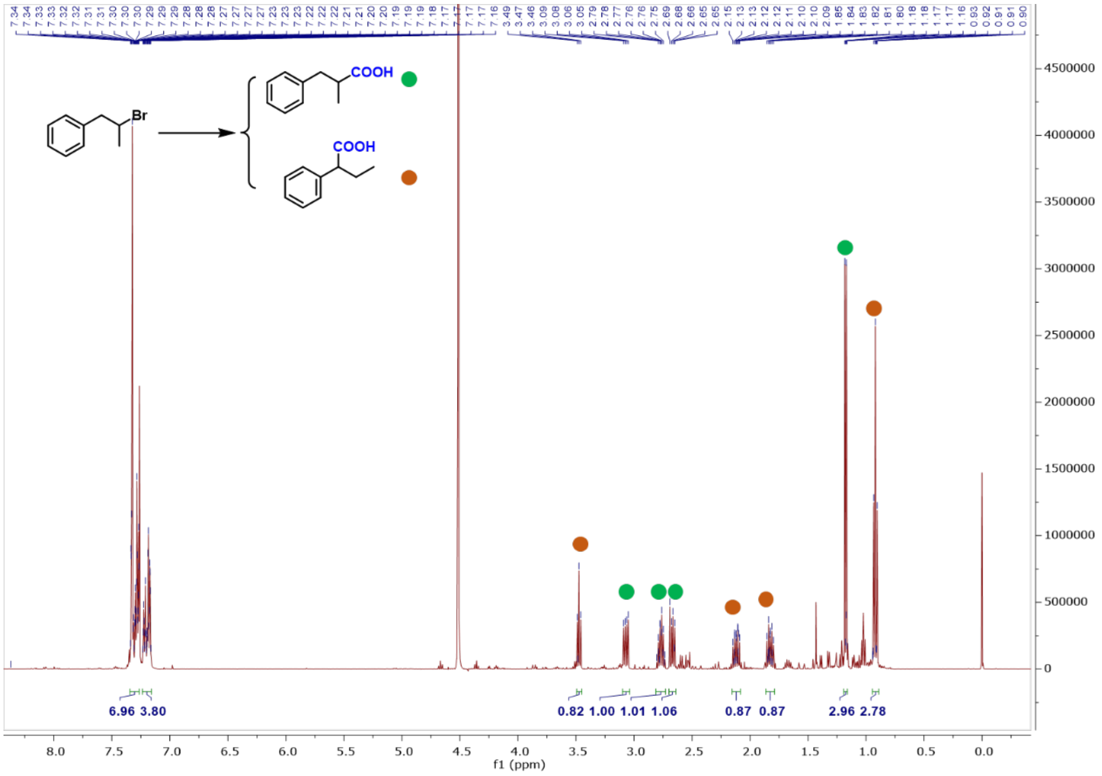

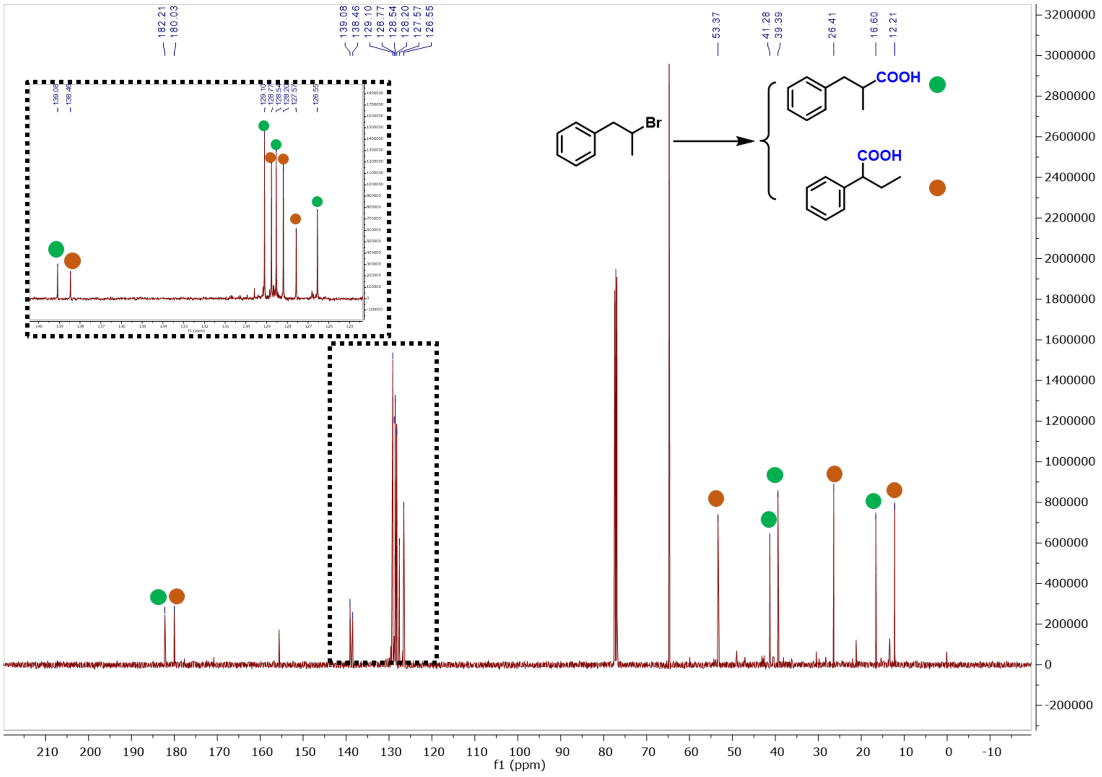

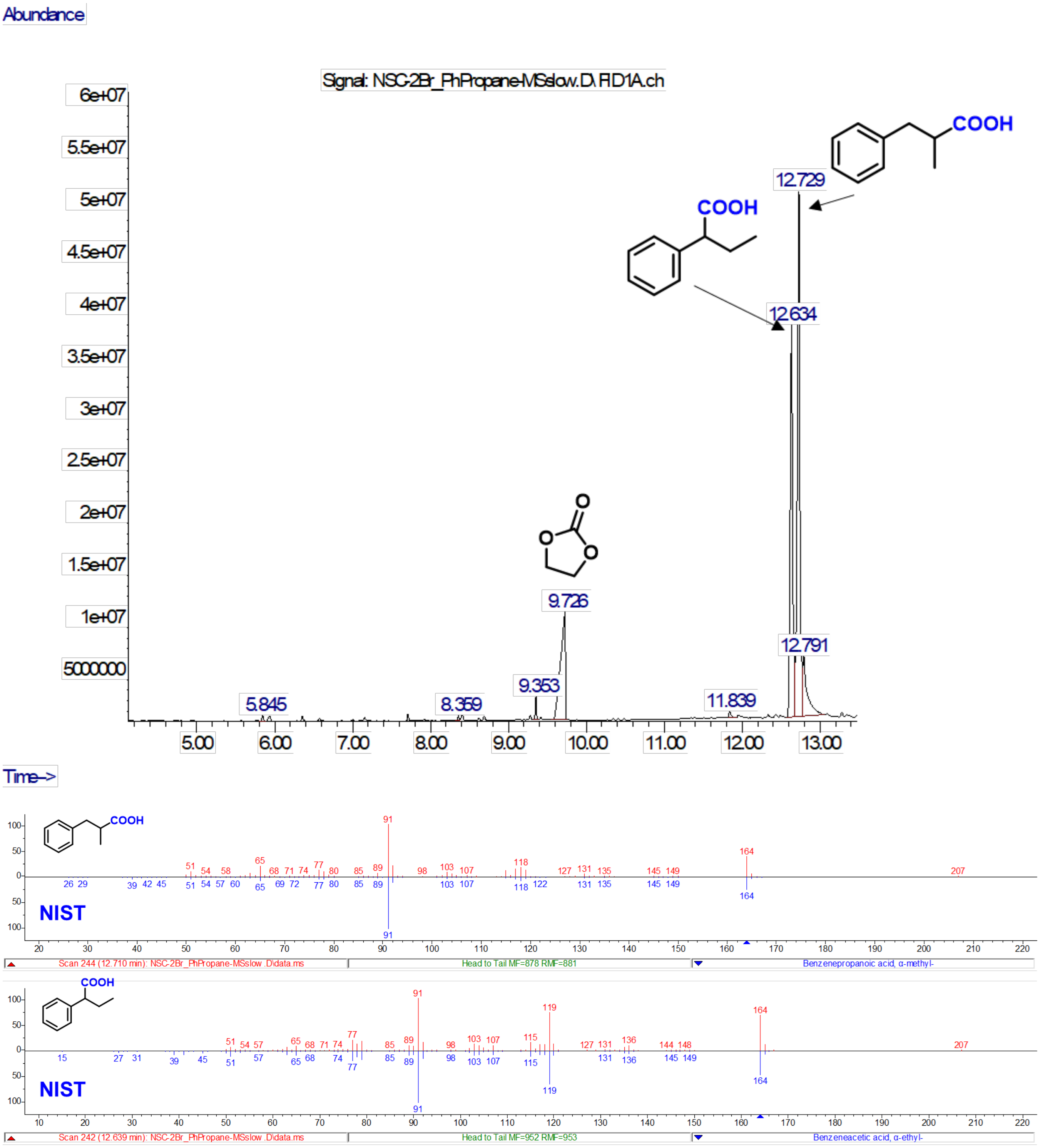
Abundance
Signal: NSC-2Br_PhPropane-MSslow.D\FID1A.ch
6e+07
5.5e+07
5e+07
4.5e+07
4e+07
3.5e+07
3e+07
2.5e+07
2e+07
1.5e+07
1e+07
5000000
COOH
COOH
12.729
12.634
9.726
12.791
9.353
11.839
5.845
8.359
5.00
6.00
7.00
8.00
9.00
10.00
11.00
12.00
13.00
Time-->
COOH
NIST
Scan 244 (12.710 min): NSC-2Br_PhPropane-MSslow.D\data.ms
Head to Tail MF=878 RMF=881
Benzenepropanoic acid, α-methyl-
COOH
NIST
Scan 242 (12.639 min): NSC-2Br_PhPropane-MSslow.D\data.ms
Head to Tail MF=952 RMF=953
Benzeneacetic acid, α-ethyl-

2-Bromo-1-phenylpropane reagent

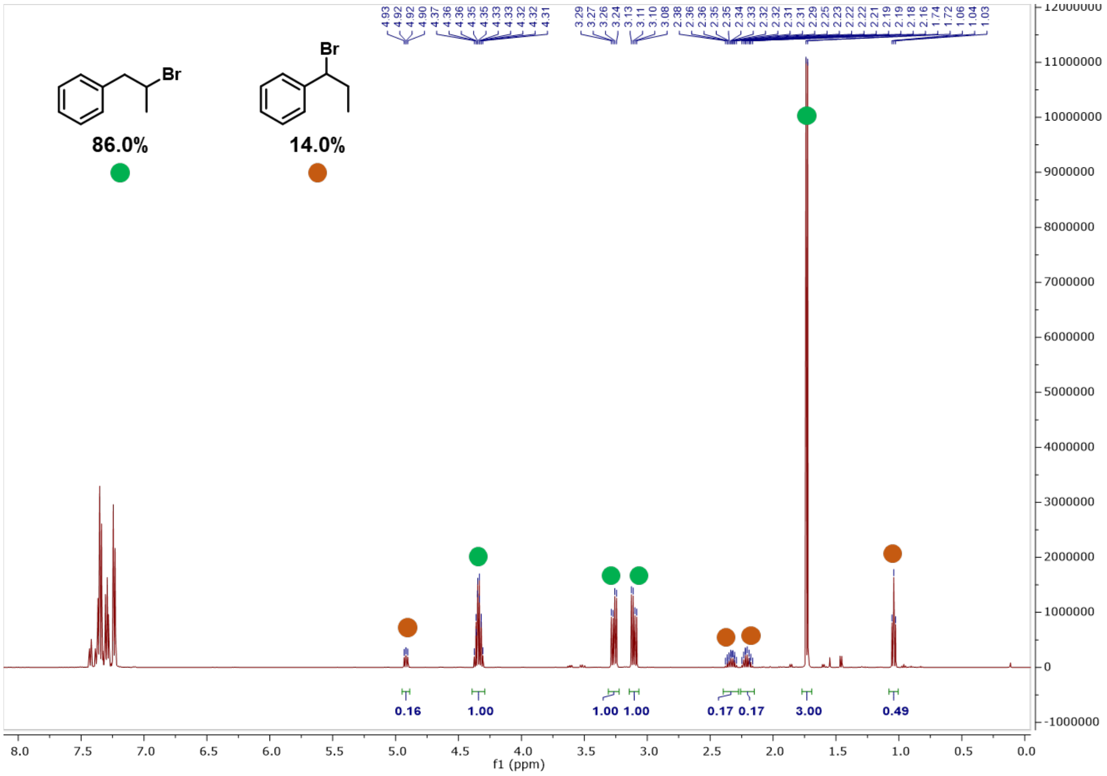

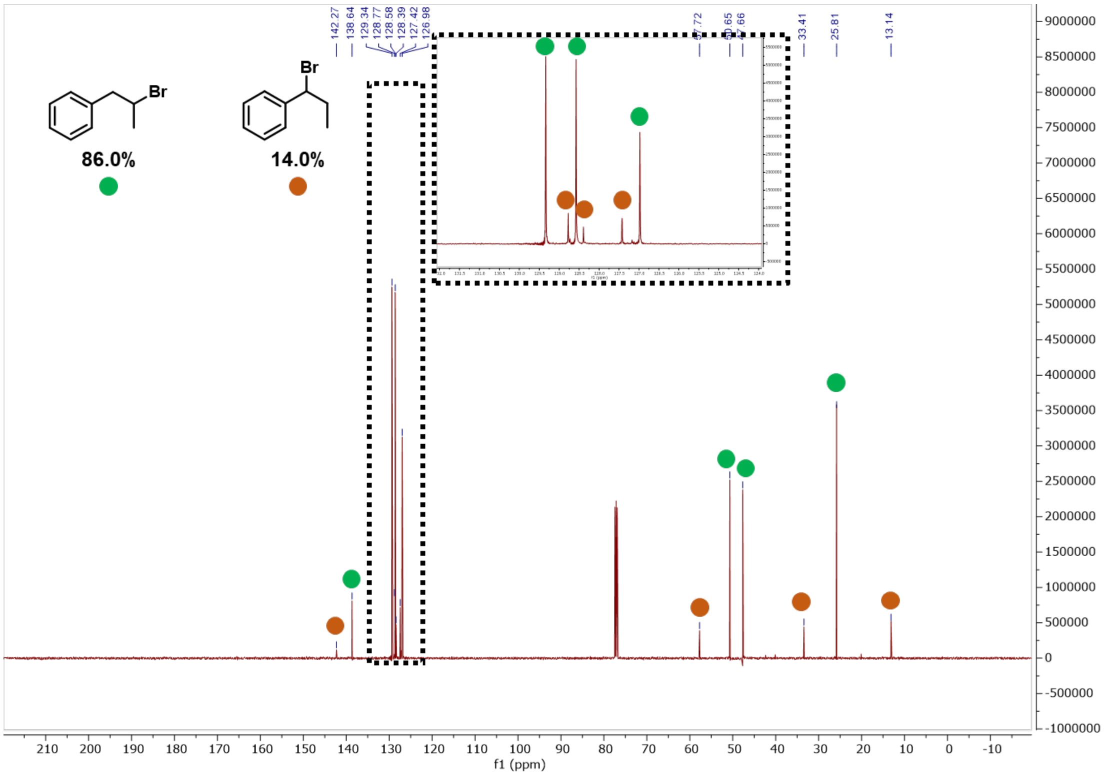

2,2-Dimethyl butyric acid (7a)

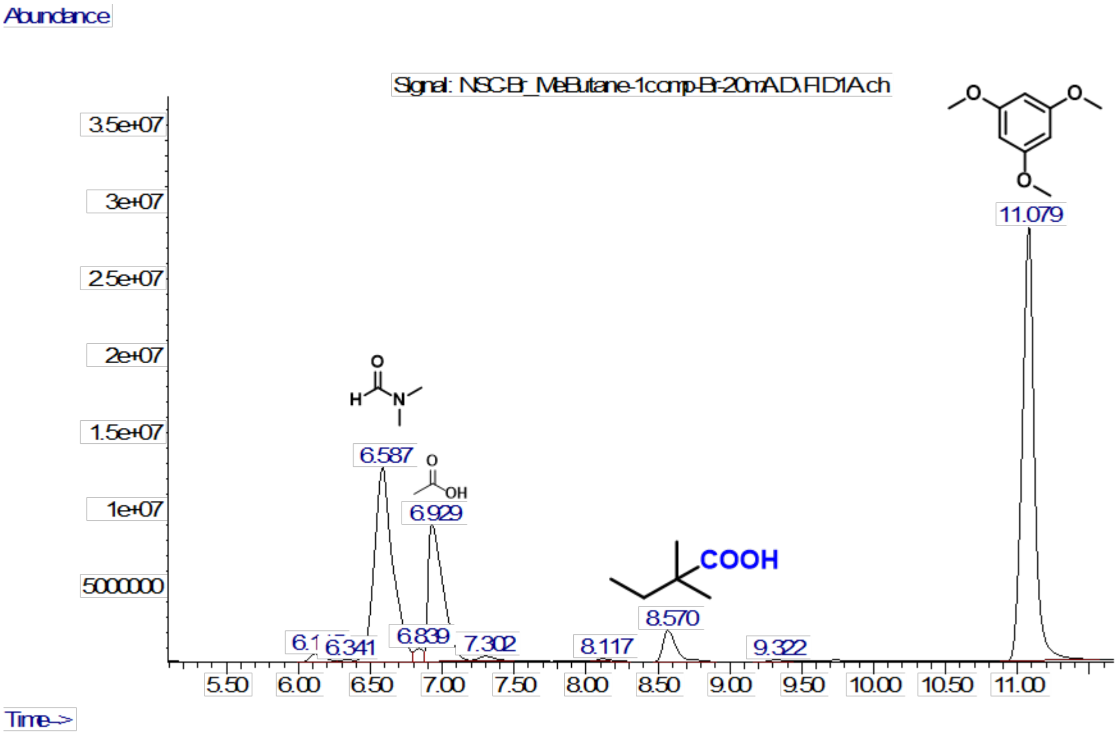

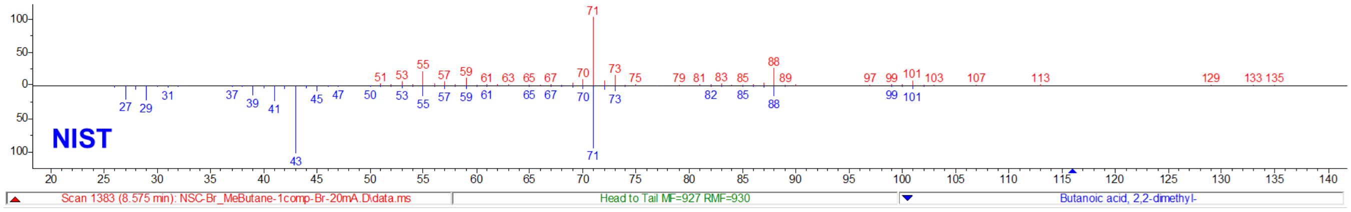

1-Adamantane carboxylic acid (8a)

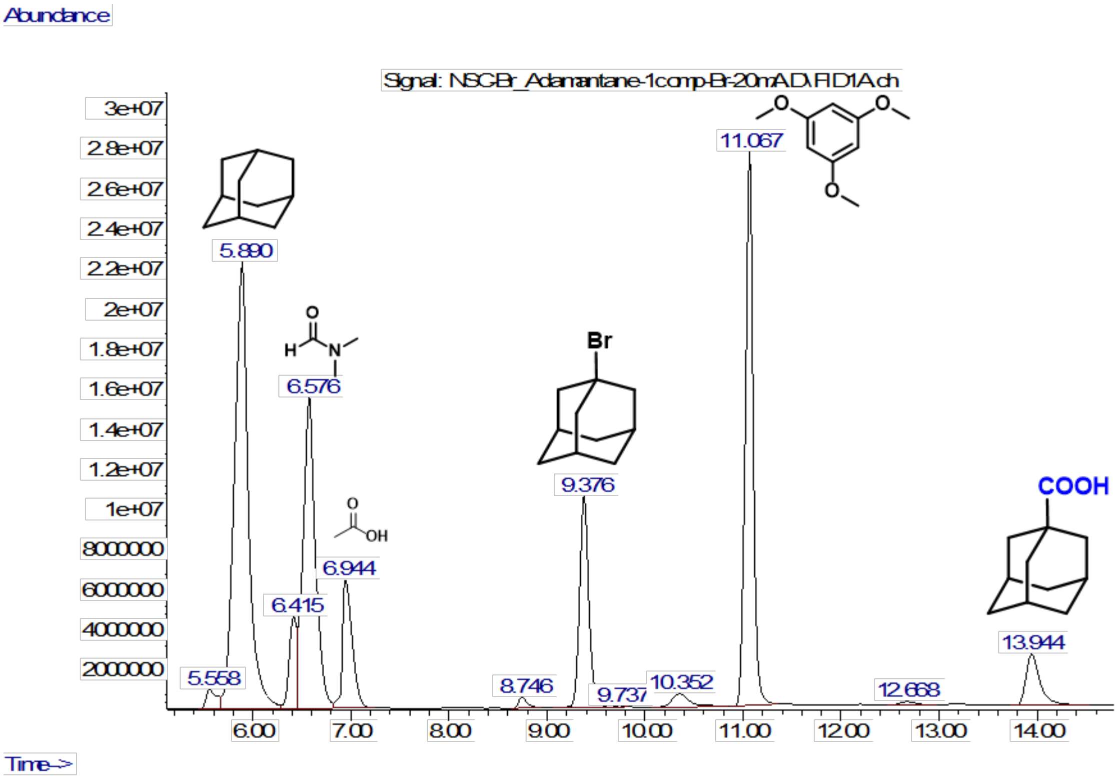

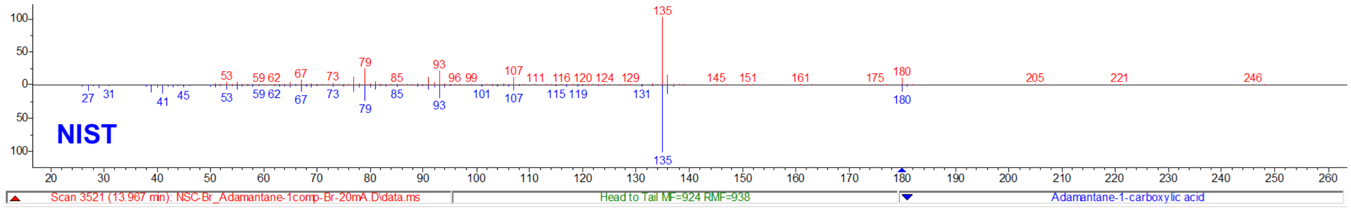

Phenylacetic acid (9a)

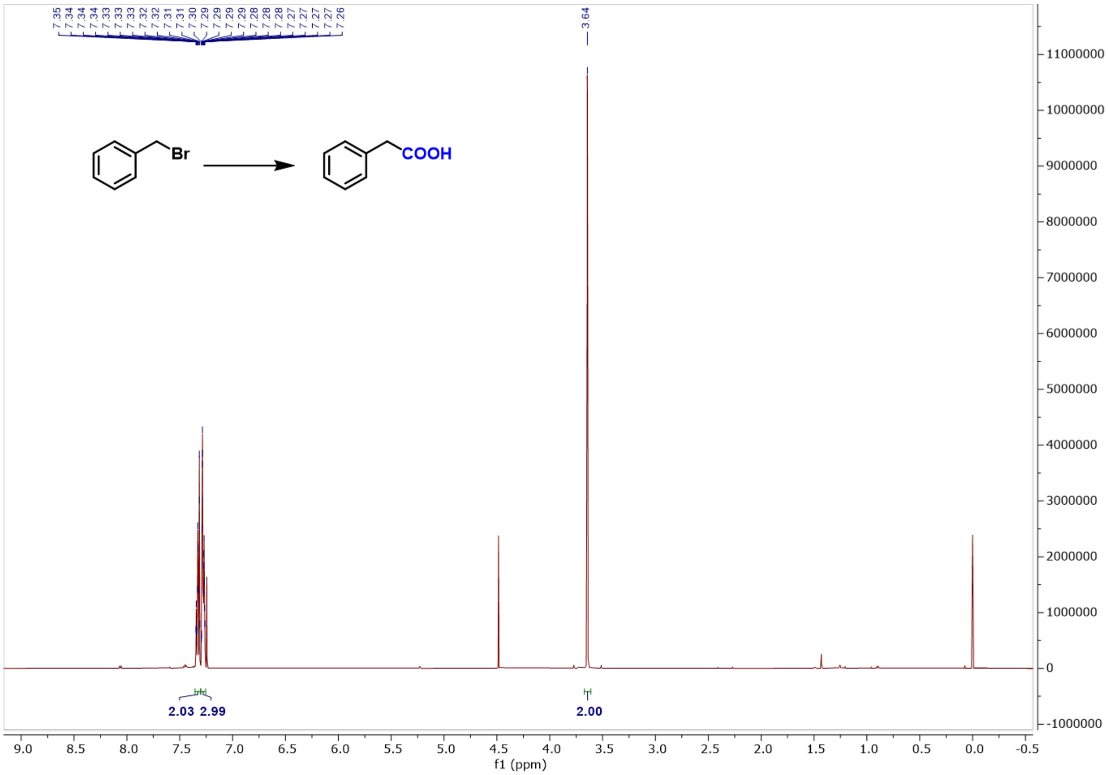

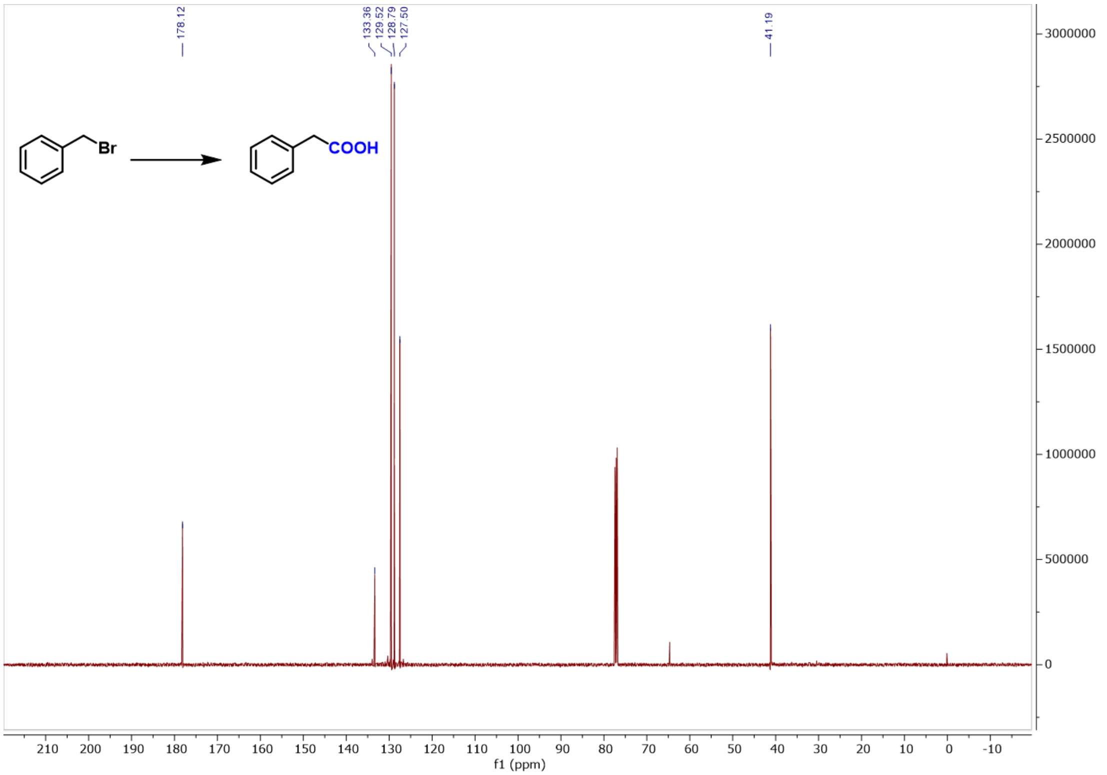

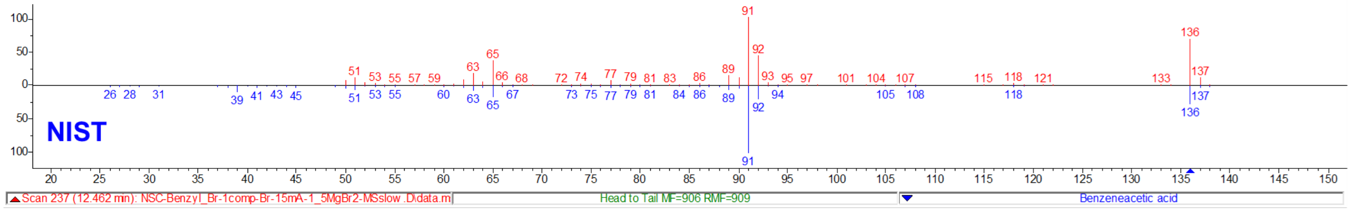
NIST
Scan 237 (12.462 min): NSC-Benzyl_Br-1comp-Br-15mA-1_5MgBr2-MSslow .D\data.ms
Head to Tail MF=906 RMF=909
Benzeneacetic acid

2-([1,1'-Biphenyl]-3-yl)acetic acid (11a)

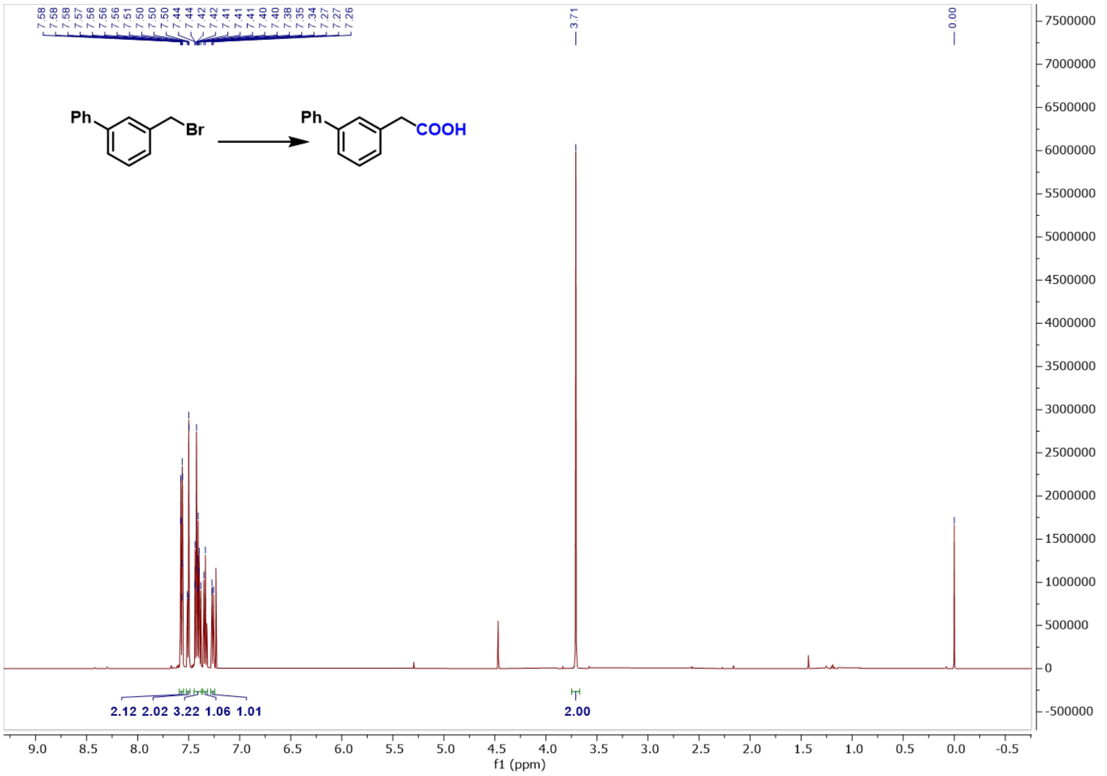

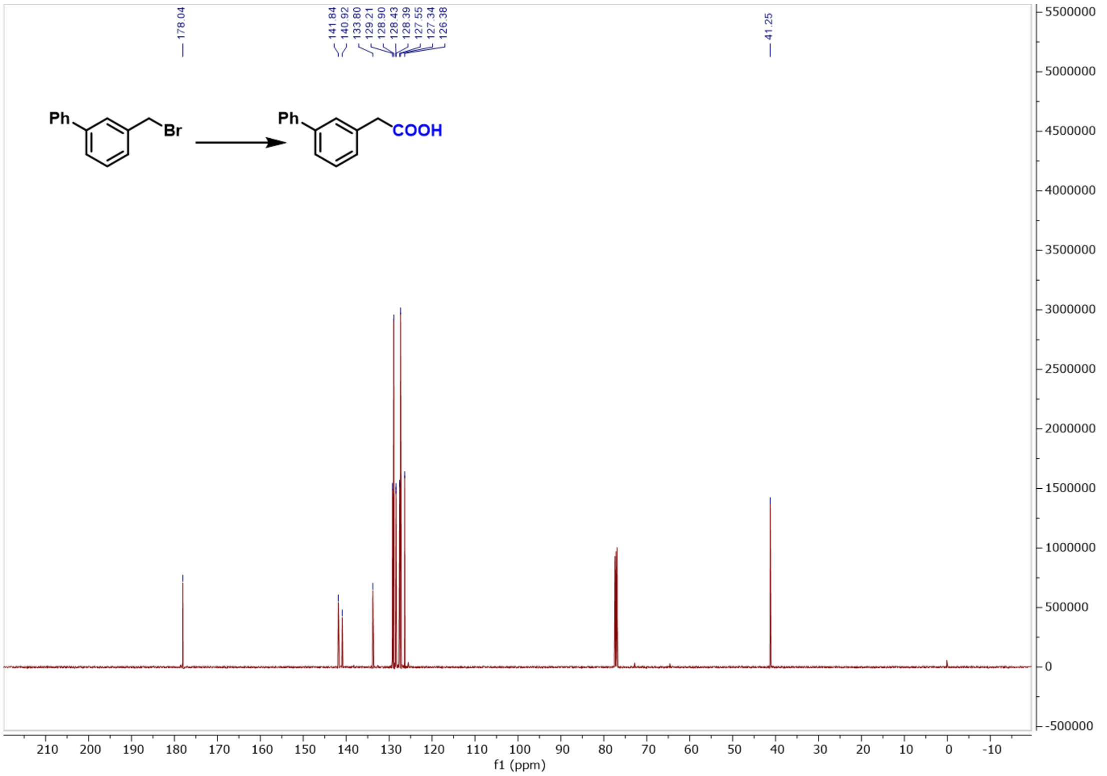

2-(3-Cyanophenyl)acetic acid (12a)

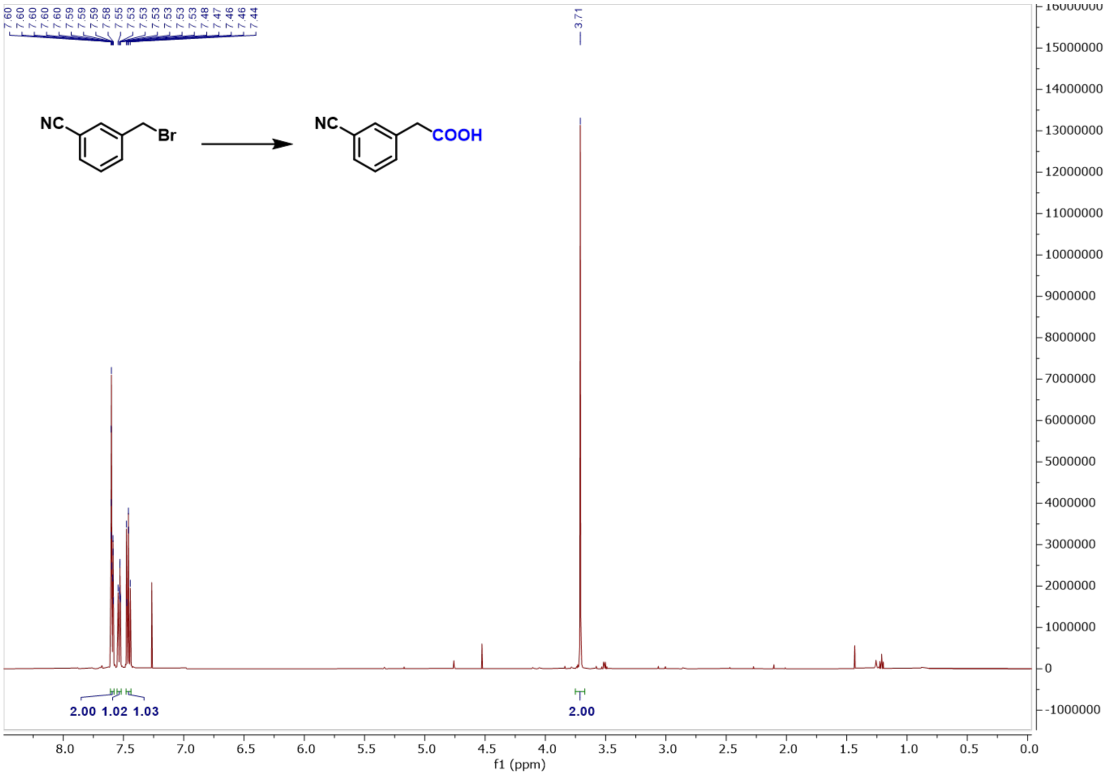

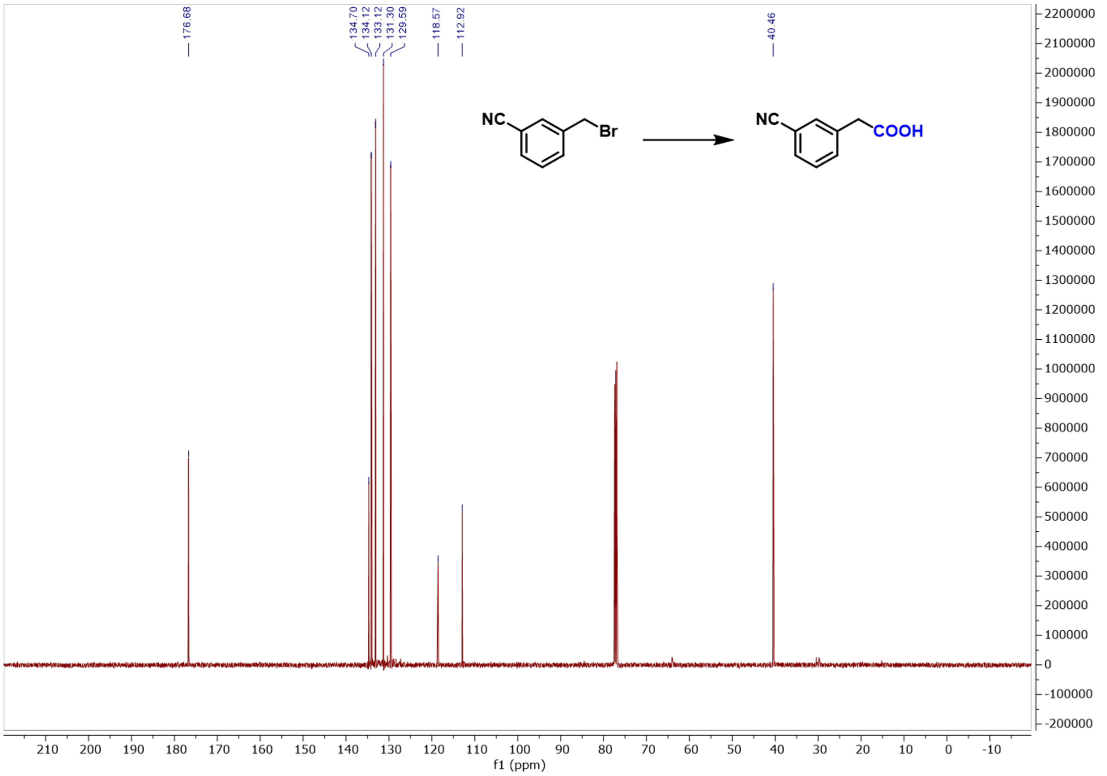

2-(*m*-Tolyl)acetic acid (10a)

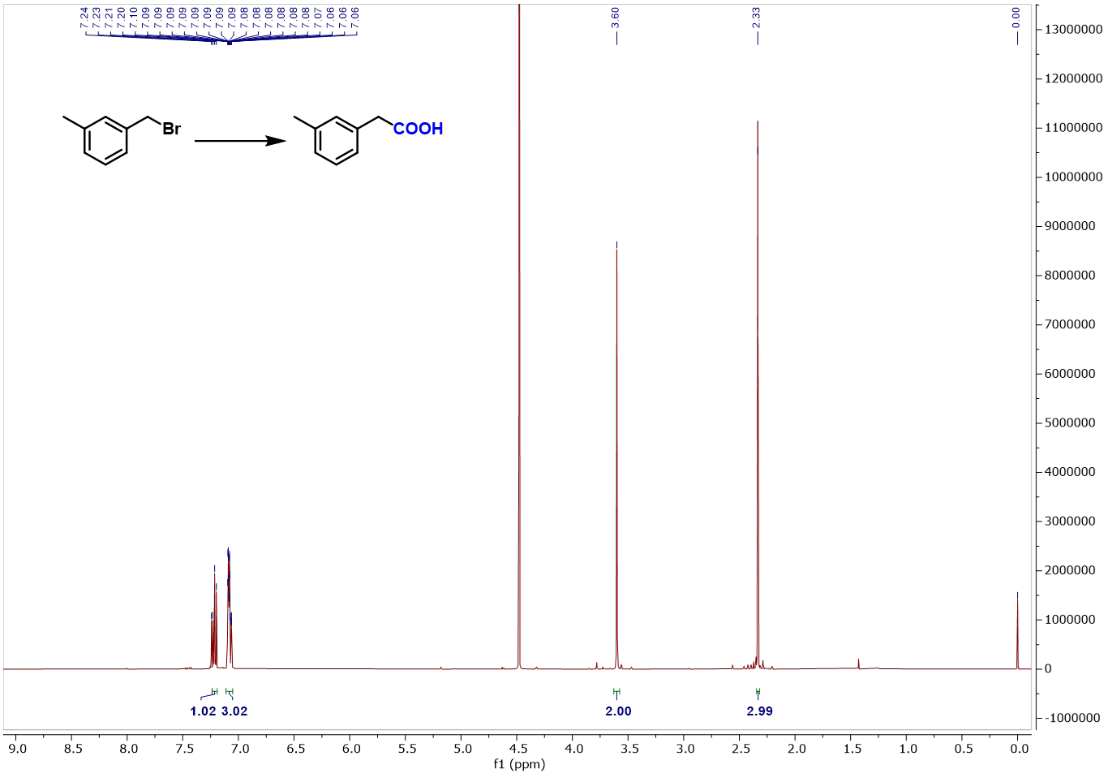

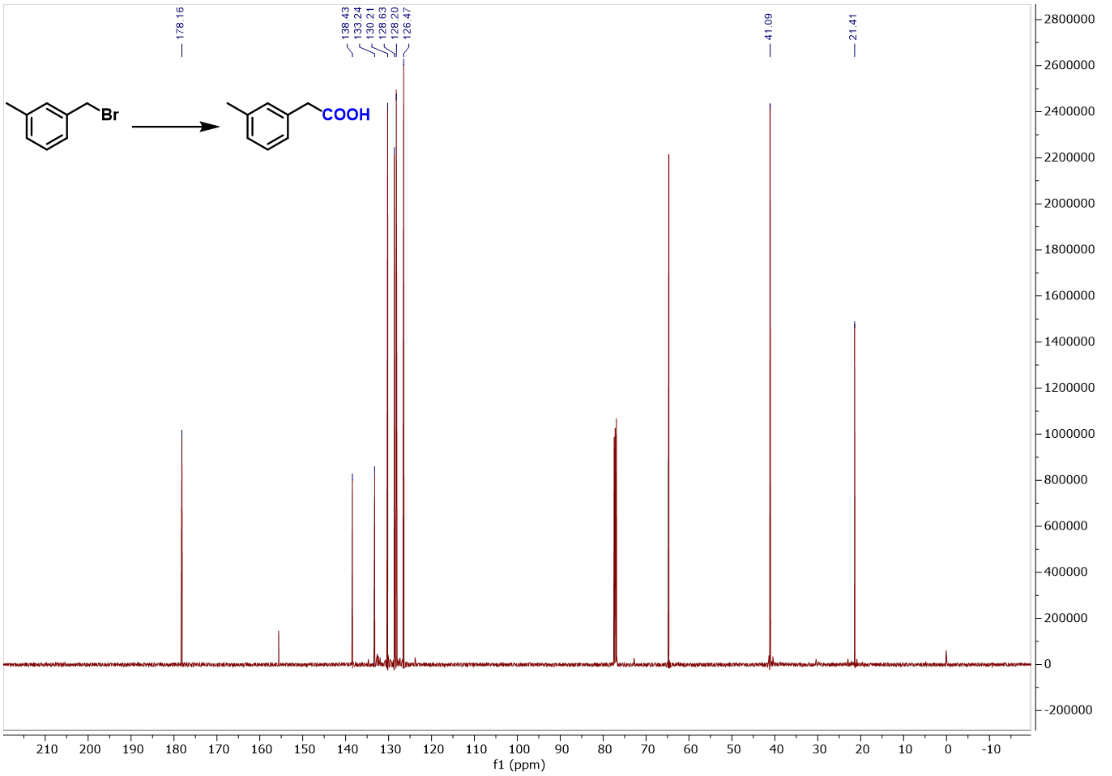

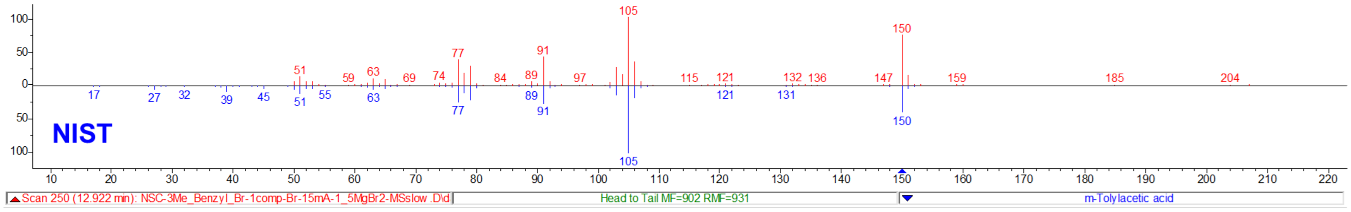

105
150
77
91
51
63
59
69
74
84
89
97
115
121
132
136
147
159
185
204
17
27
32
39
45
51
55
63
77
89
91
105
121
131
150
NIST
Scan 250 (12.922 min): NSC-3Me_Benzyl_Br-1comp-Br-15mA-1_5MgBr2-MSslow .D\d
Head to Tail MF=902 RMF=931
m-Tolylacetic acid

4-Tolylacetic acid (19a)

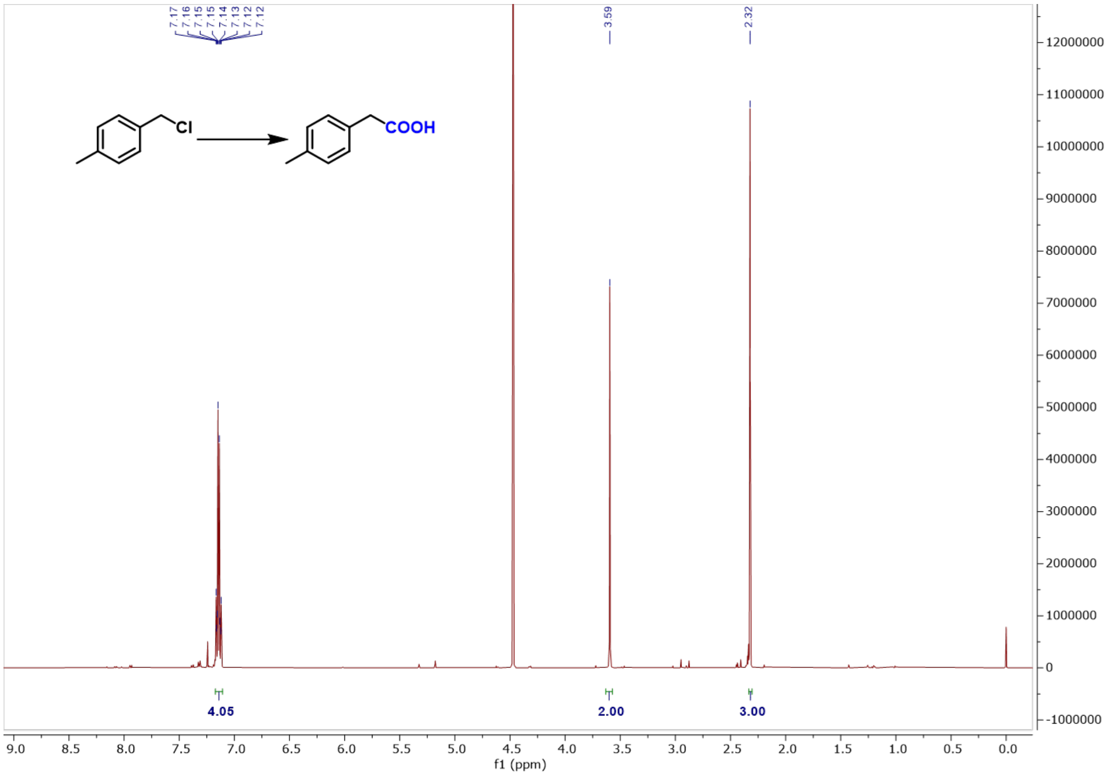

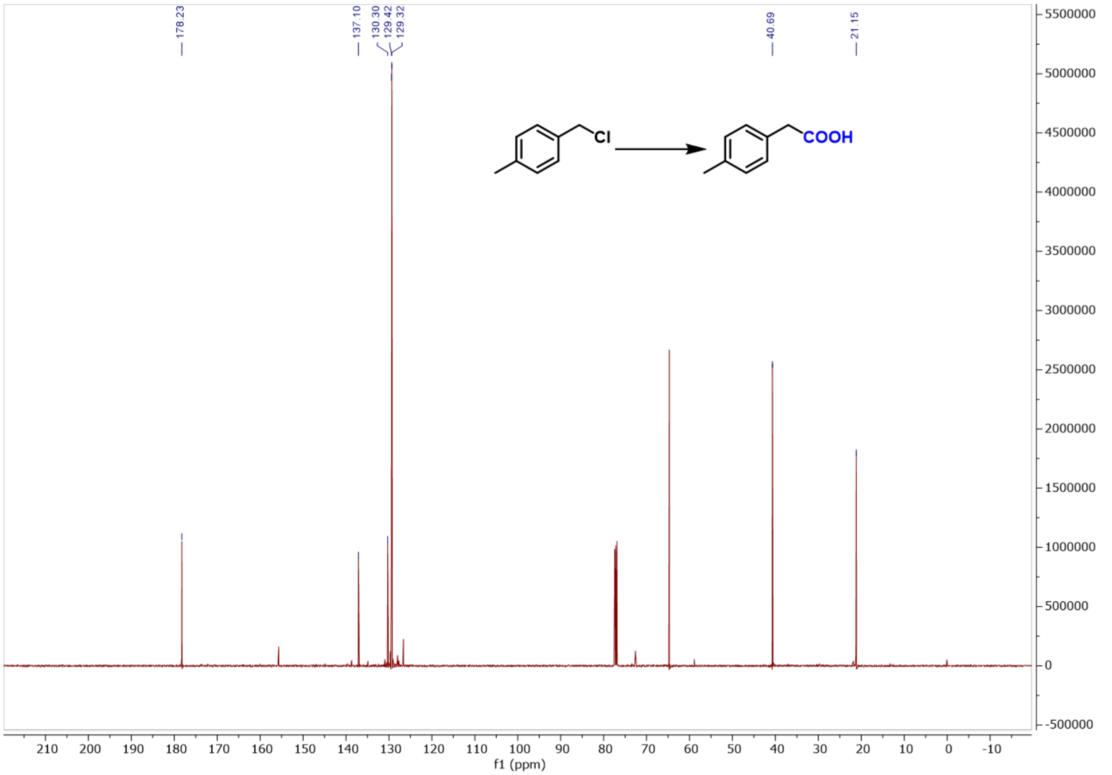

2-(4-(Methoxycarbonyl)phenyl)acetic acid (13a)

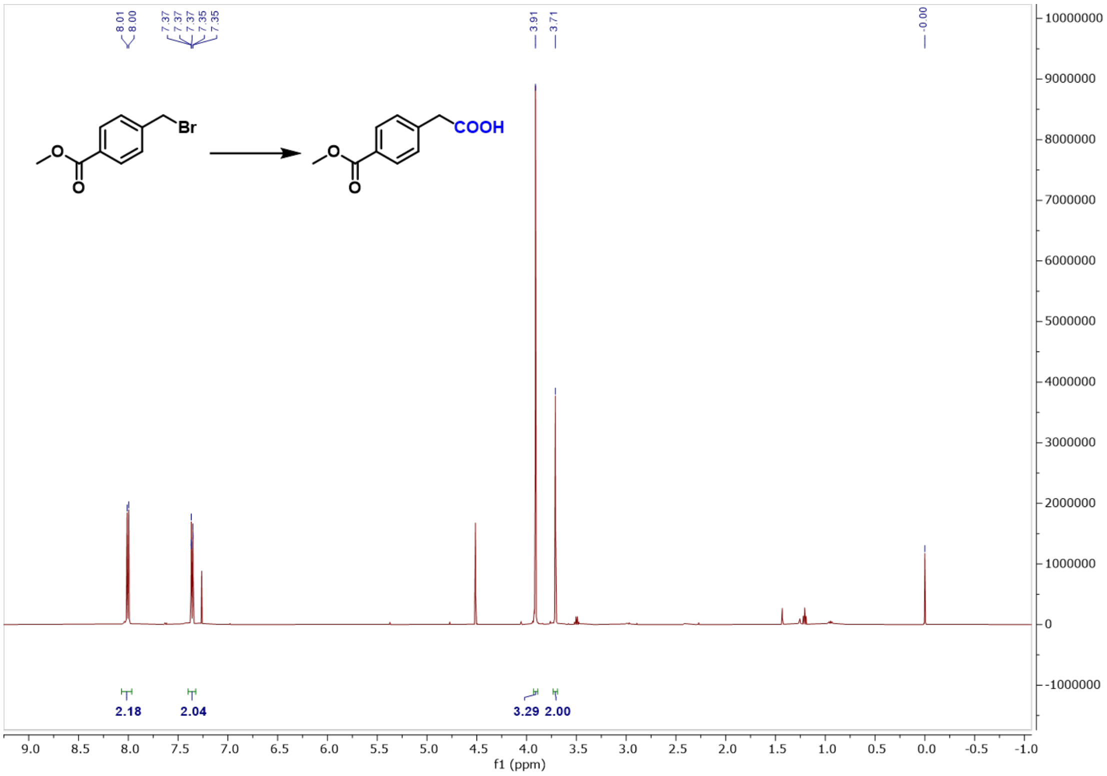

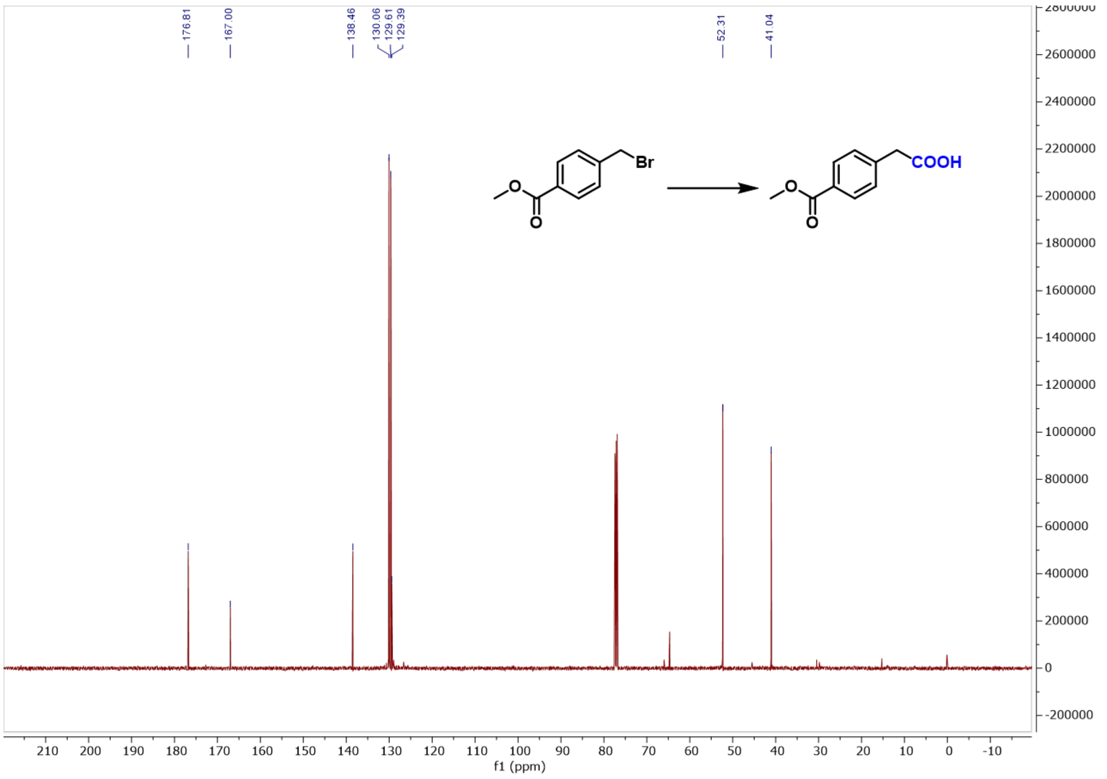

2-Phenylpropanoic acid (14a)

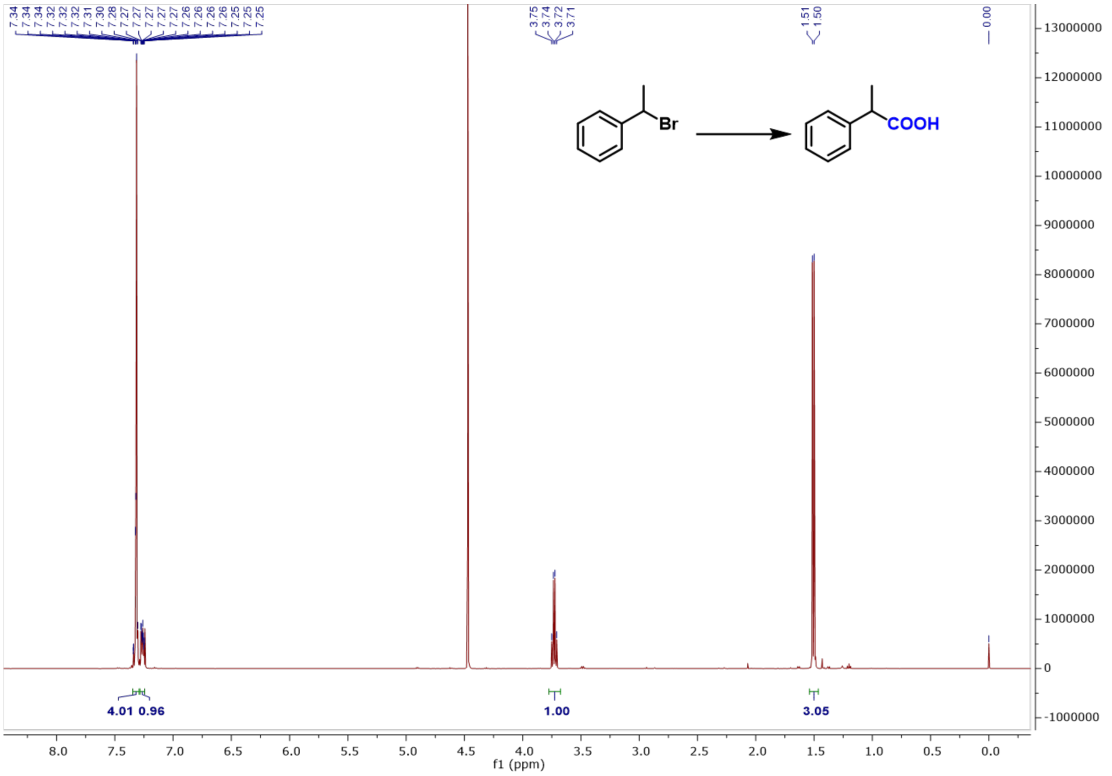

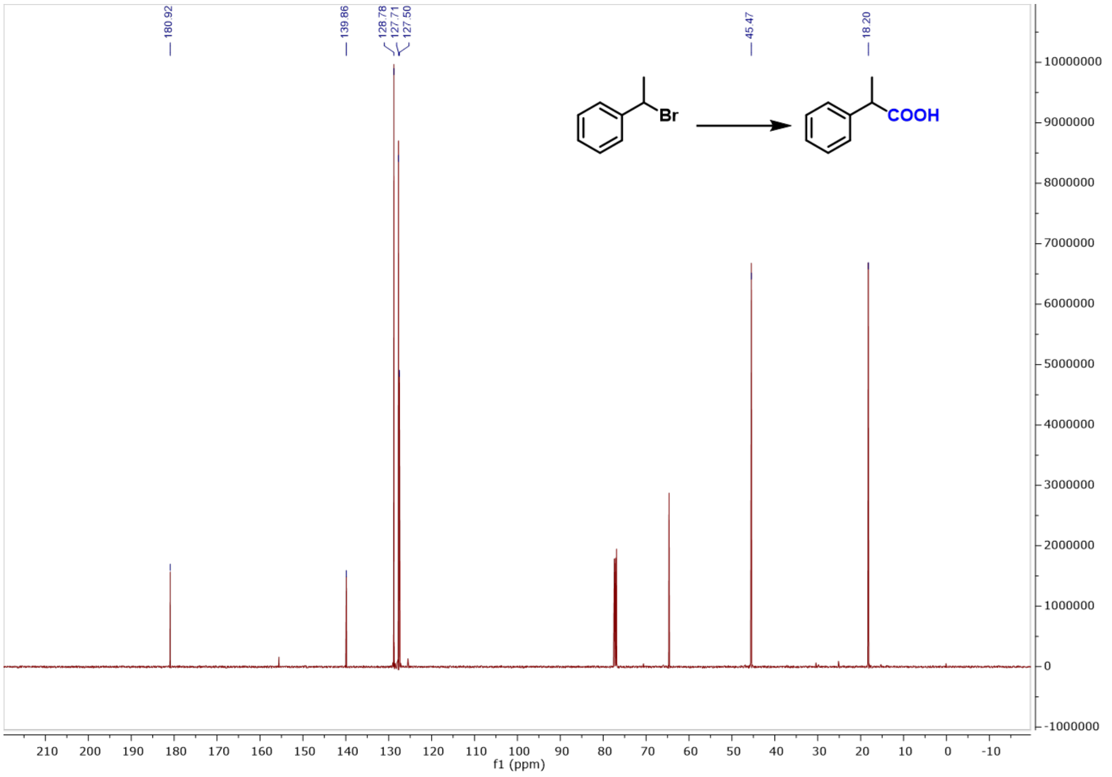

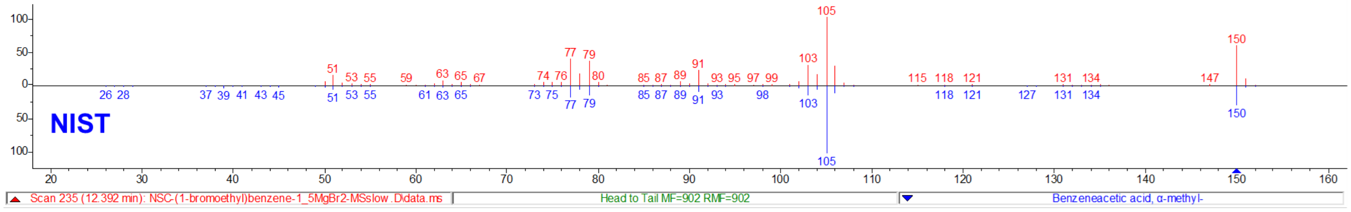
NIST
Scan 235 (12.392 min): NSC-(1-bromoethyl)benzene-1_5MgBr2-MSslow.D\data.ms
Head to Tail MF=902 RMF=902
Benzeneacetic acid, α-methyl-

2-(4-Fluorophenyl)propanoic acid (16a)

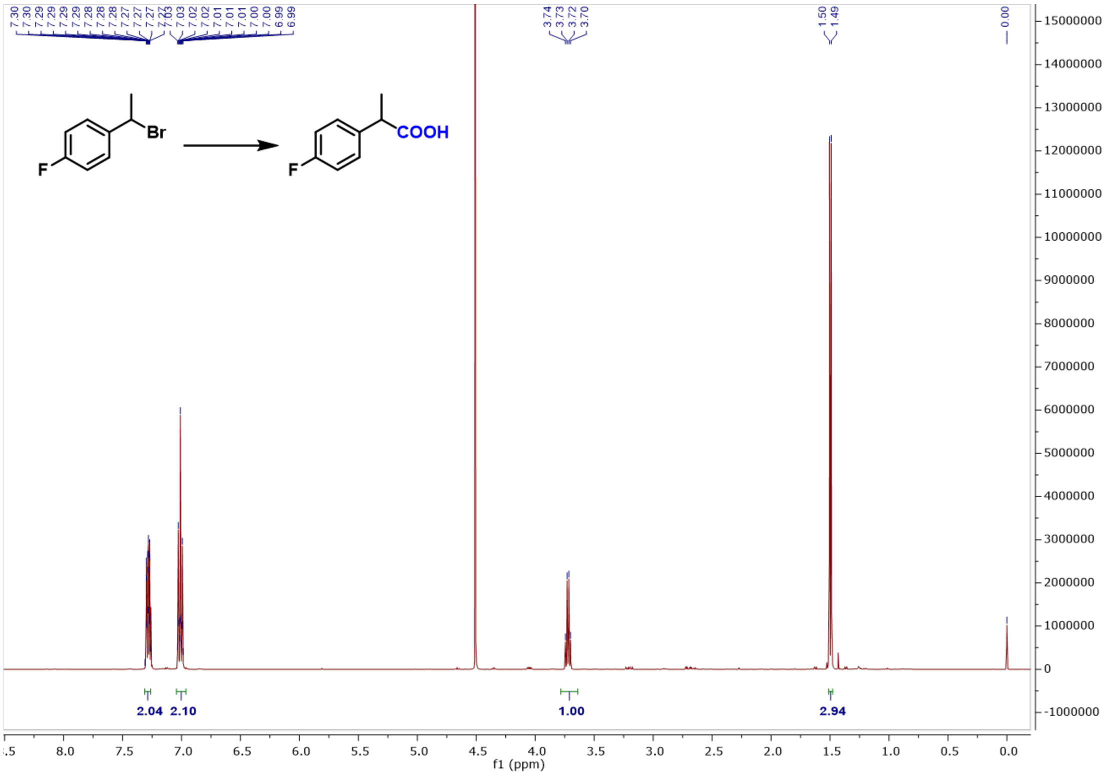

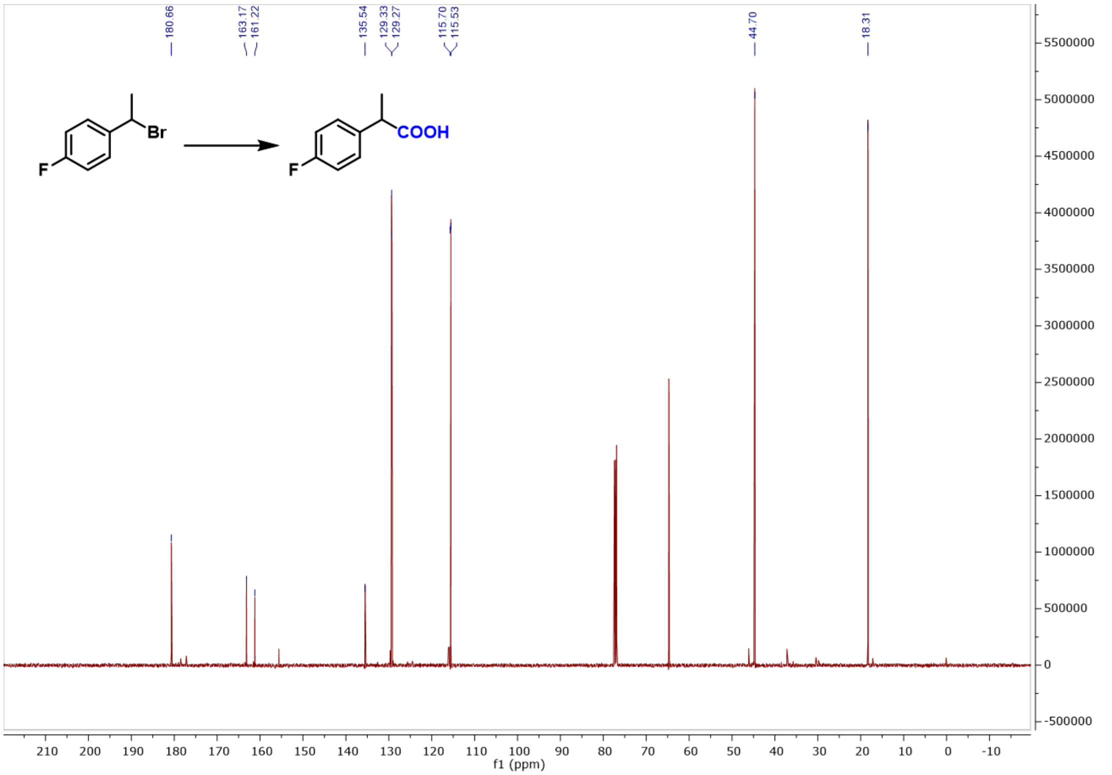

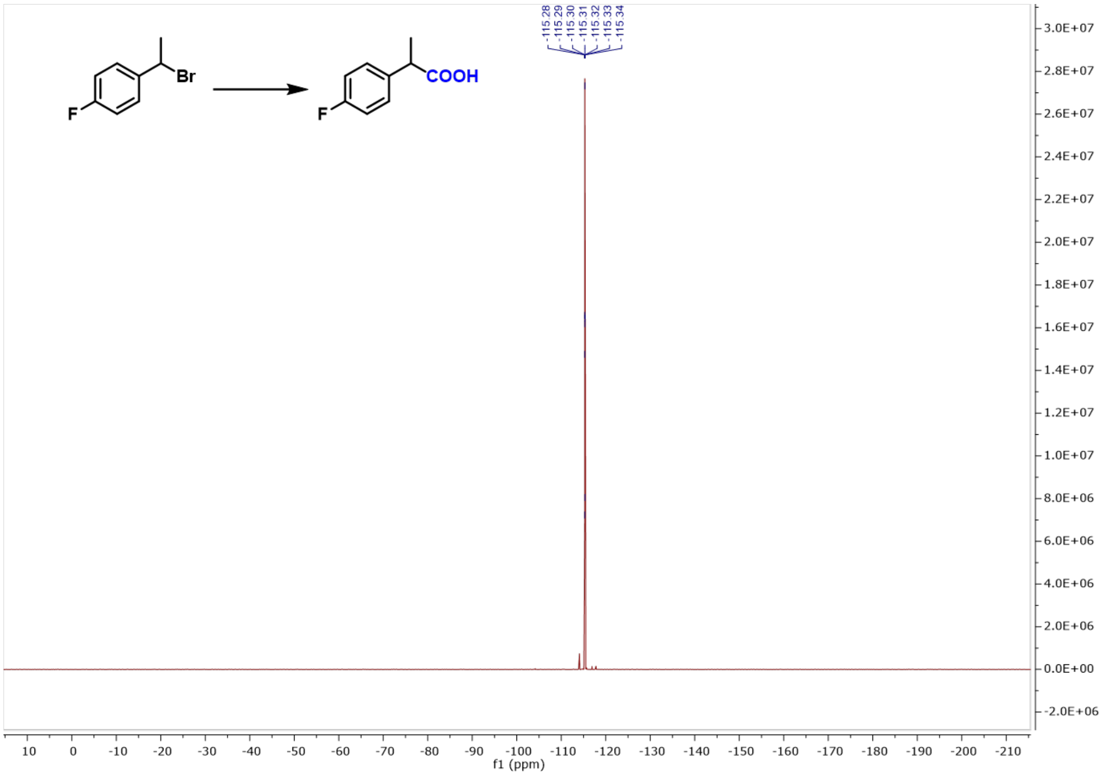

Br
F
COOH
-115.28
-115.29
-115.30
-115.31
-115.32
-115.33
-115.34
f1 (ppm)

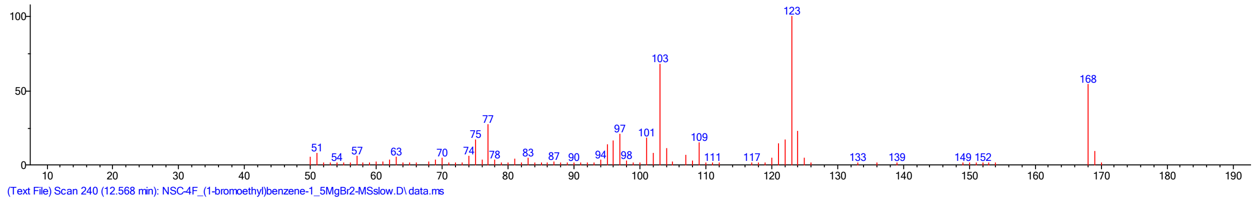

123
103
168
77
97
101
109
(Text File) Scan 240 (12.568 min): NSC-4F_(1-bromoethyl)benzene-1_5MgBr2-MSslow.D\ data.ms

2-(4-Isobutylphenyl)propanoic acid (15a)

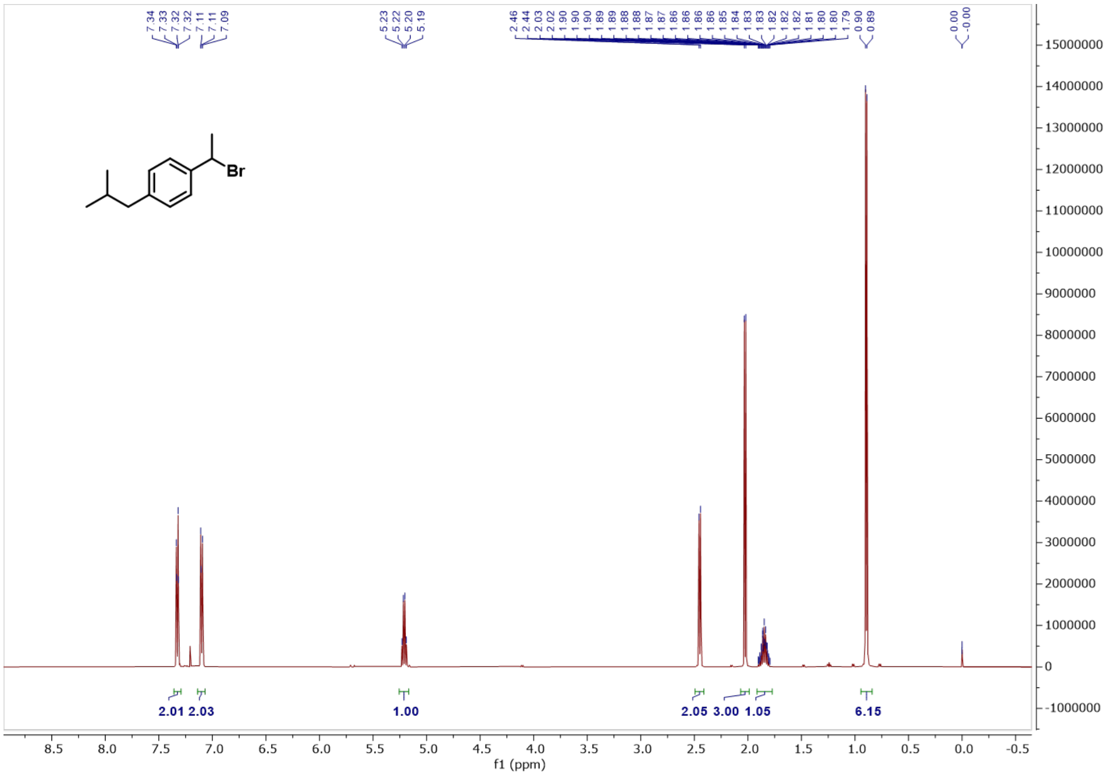

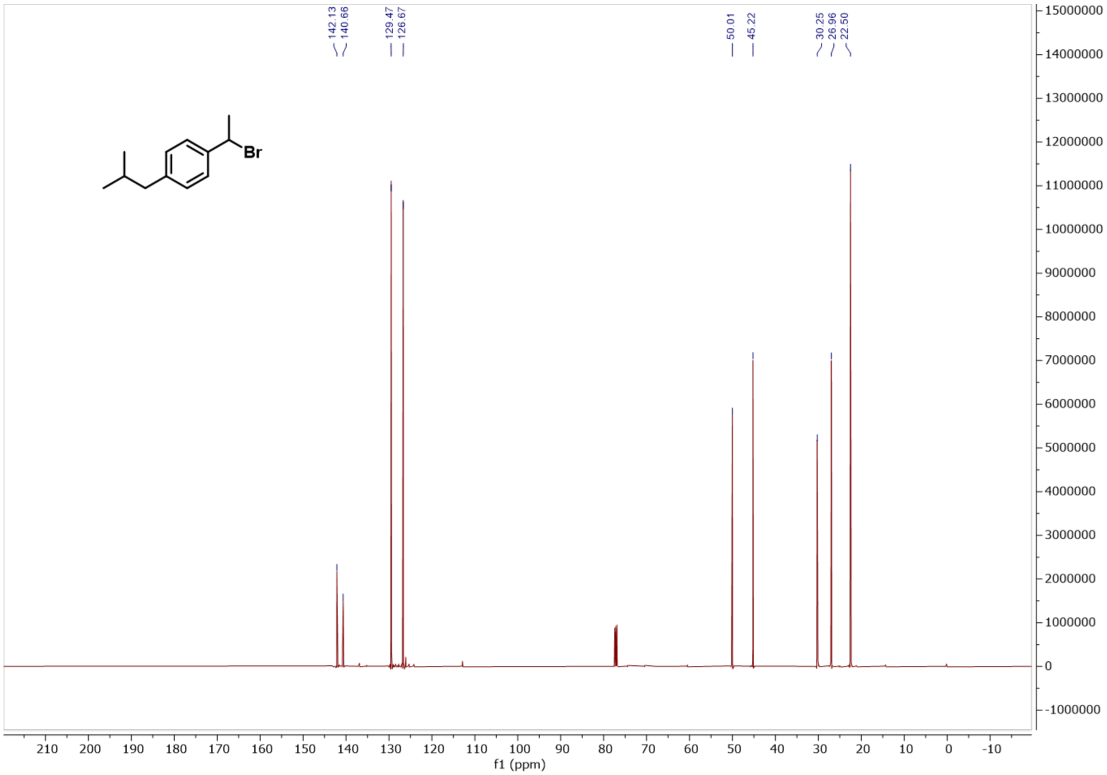

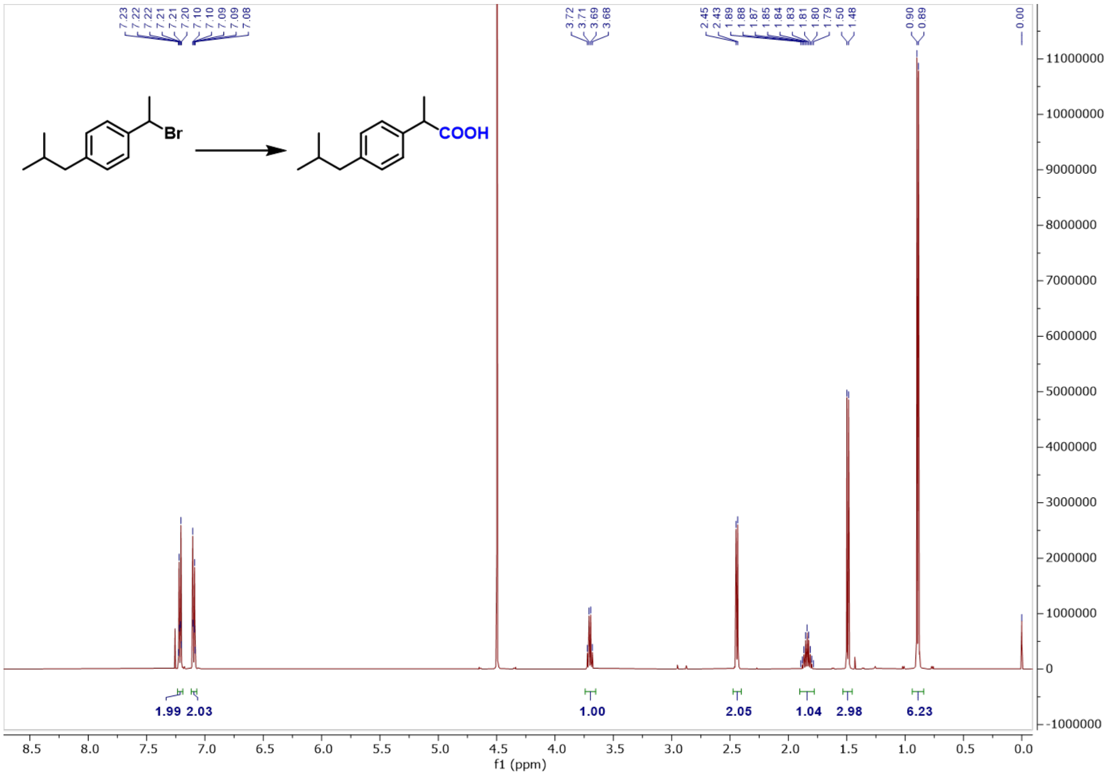

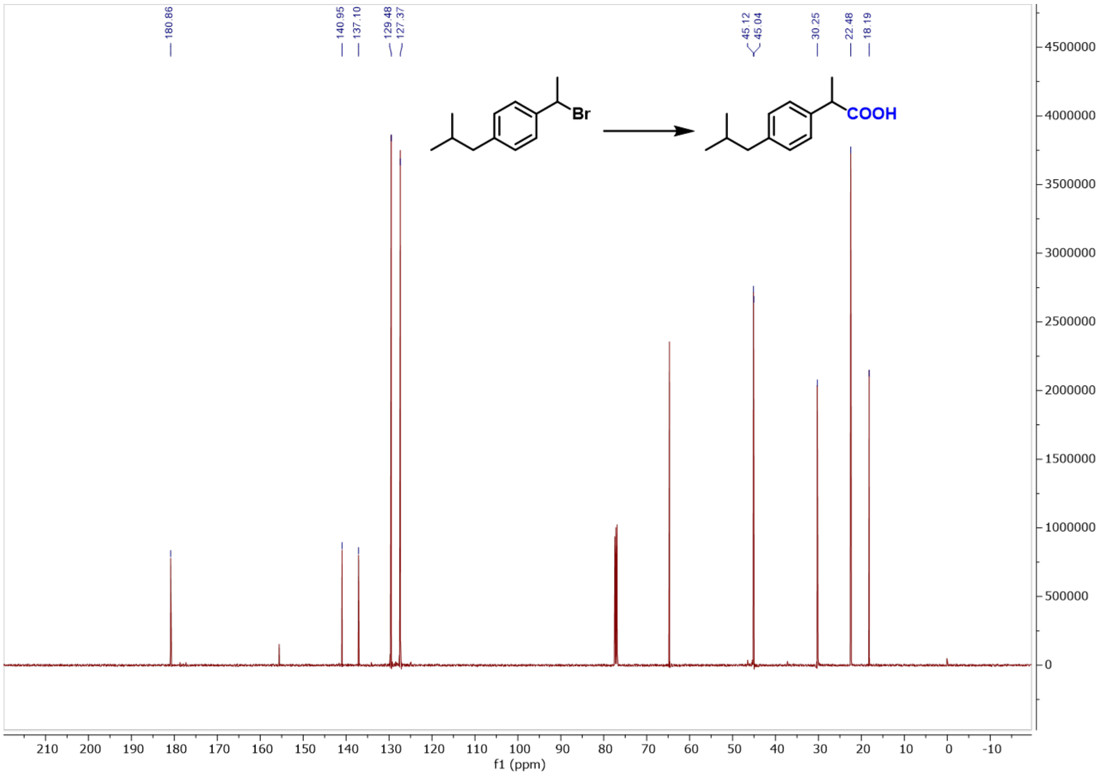

2-Methyl-2-phenylpropanoic acid (17a)

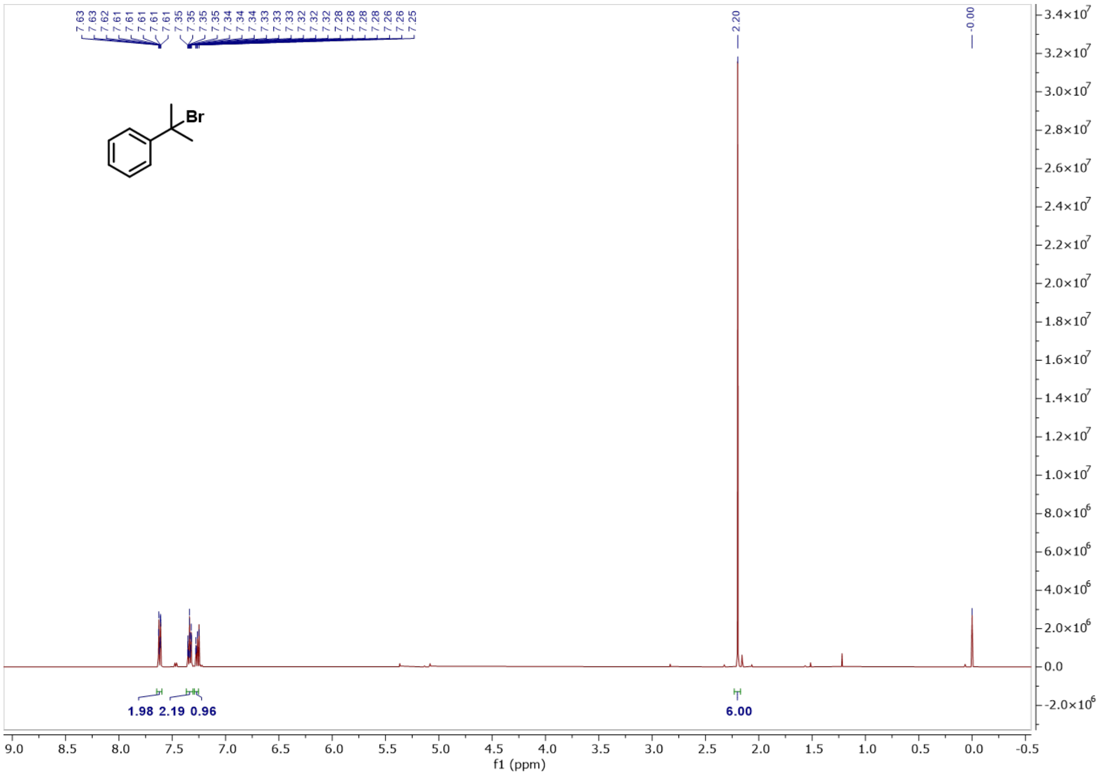

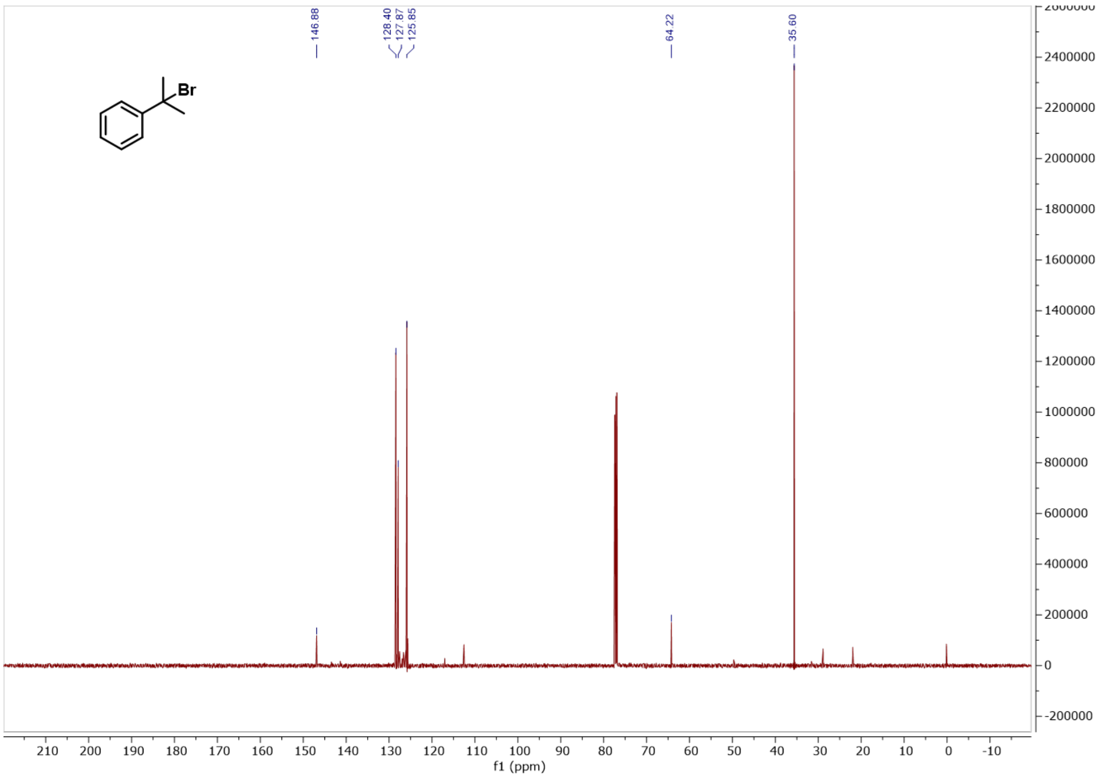

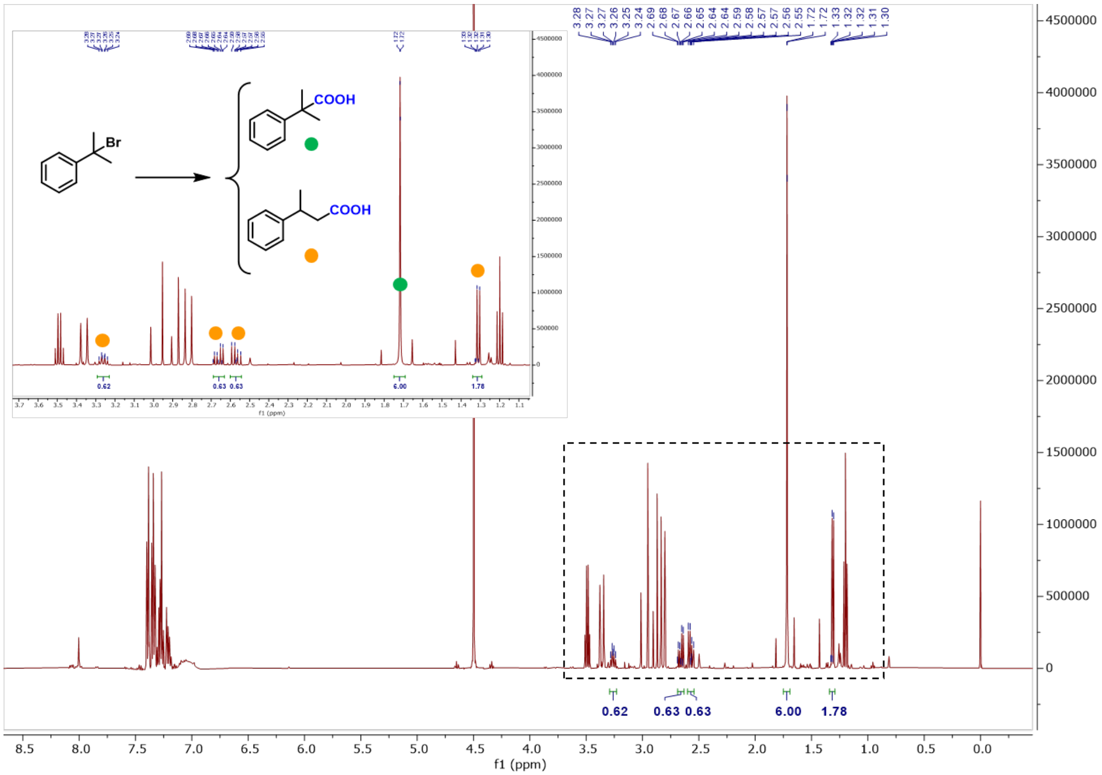
Br
COOH
COOH
0.62
0.63
0.63
6.00
1.78
f1 (ppm)

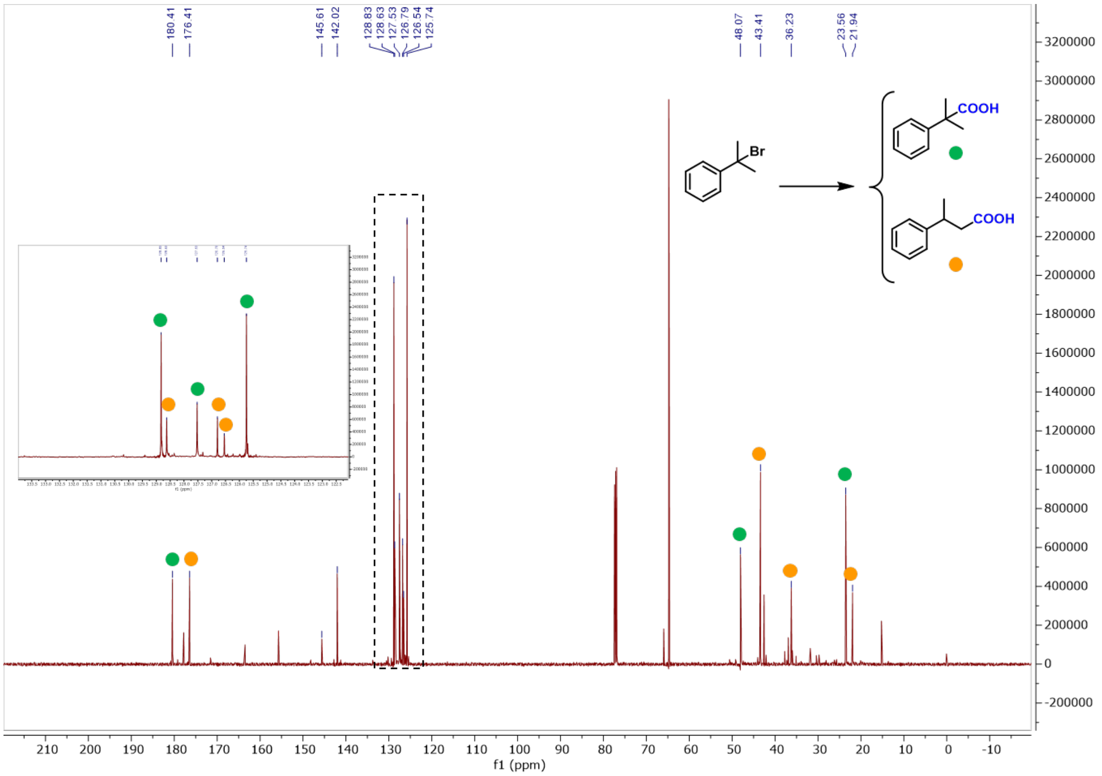
180.41
176.41
145.61
142.02
128.83
128.63
127.53
126.79
126.54
125.74
48.07
43.41
36.23
23.56
21.94
Br
COOH
COOH
f1 (ppm)

Benzoic acid (18a)

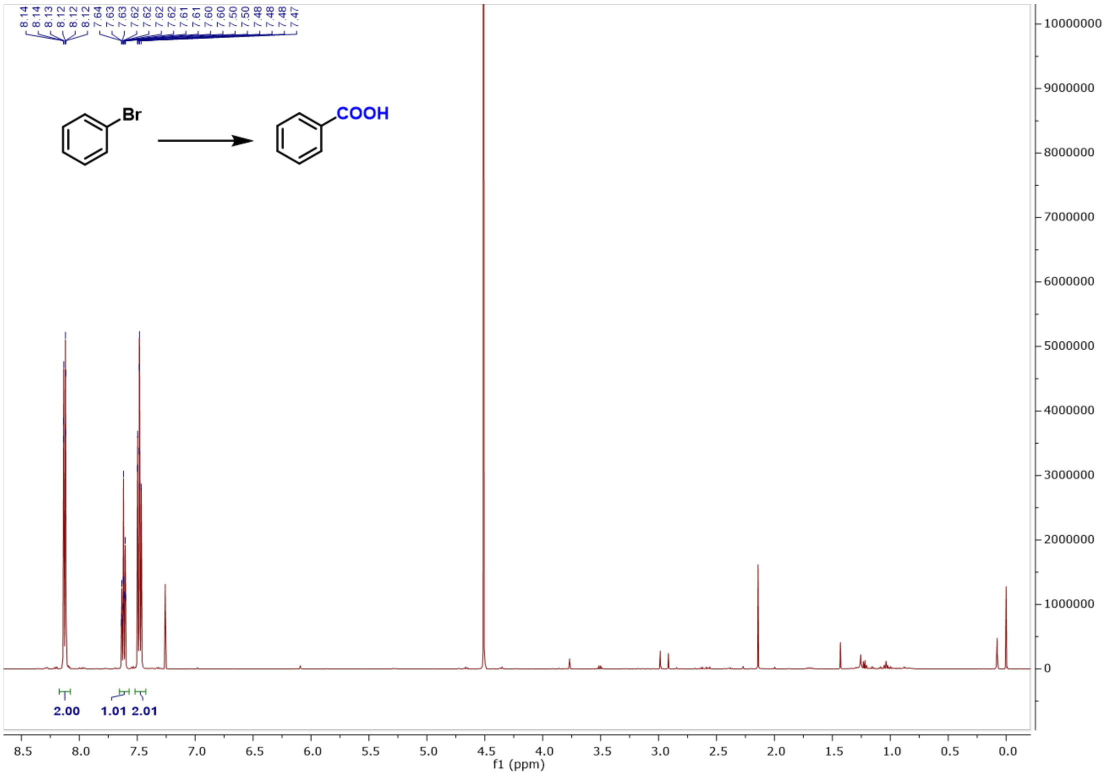

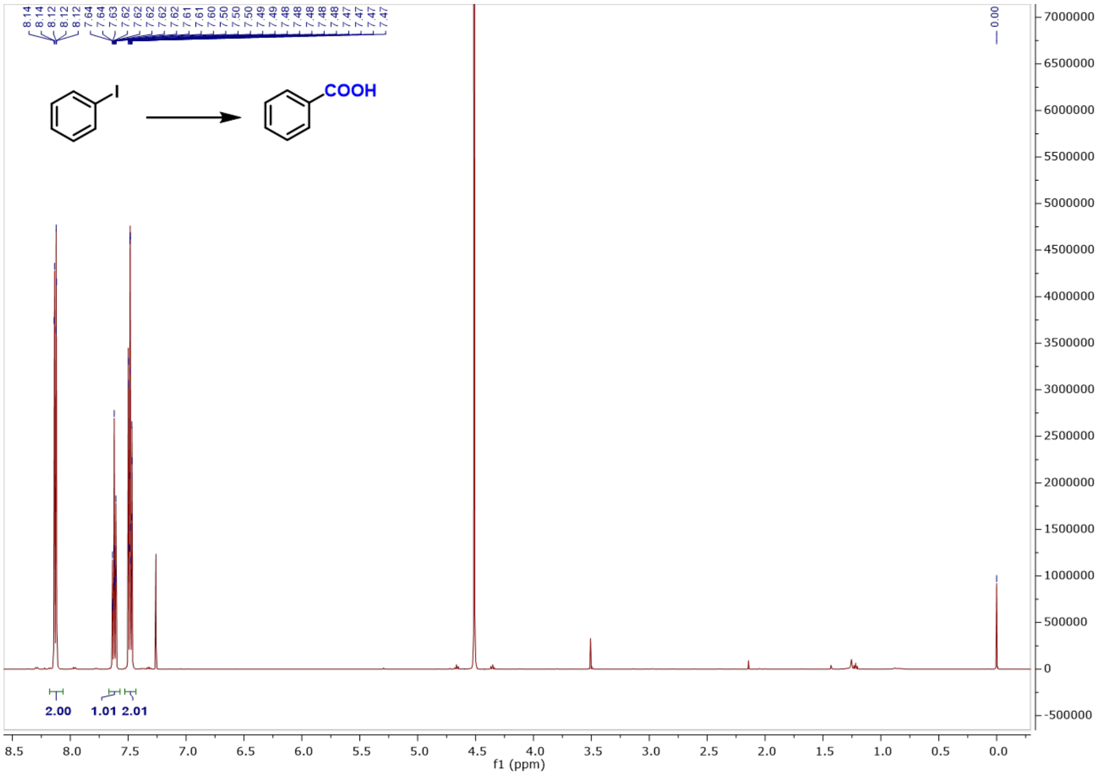

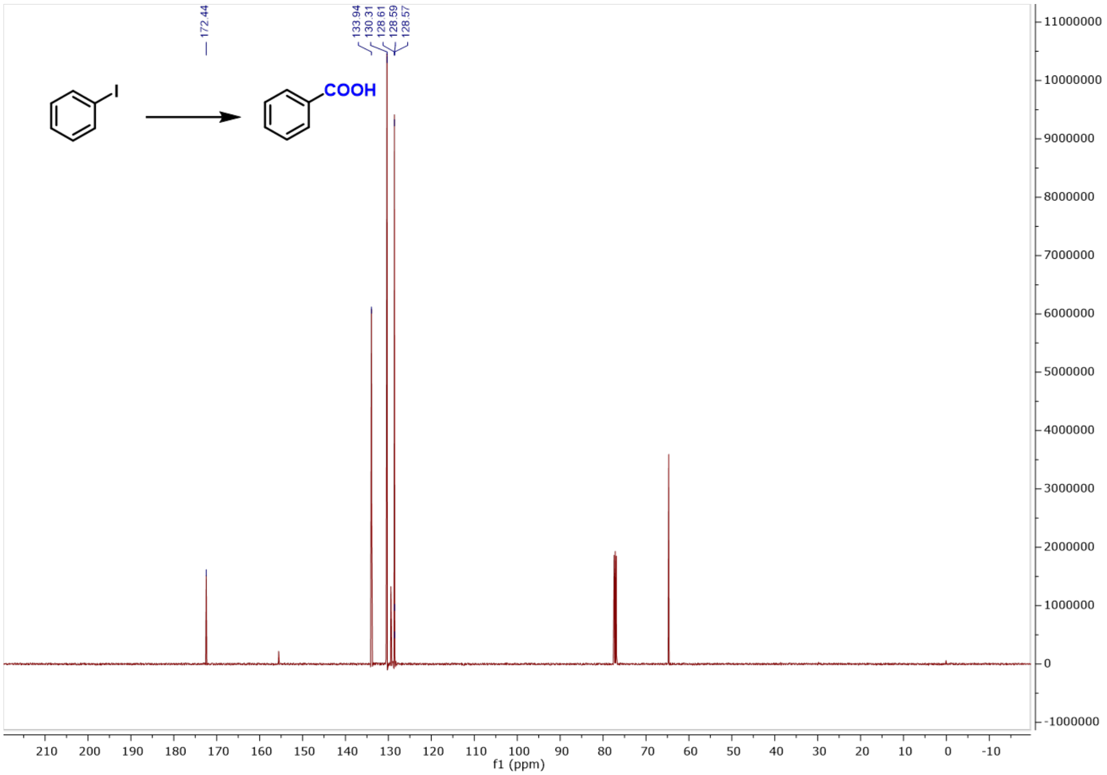

172.44
133.94
130.31
128.61
128.59
128.57
COOH
f1 (ppm)

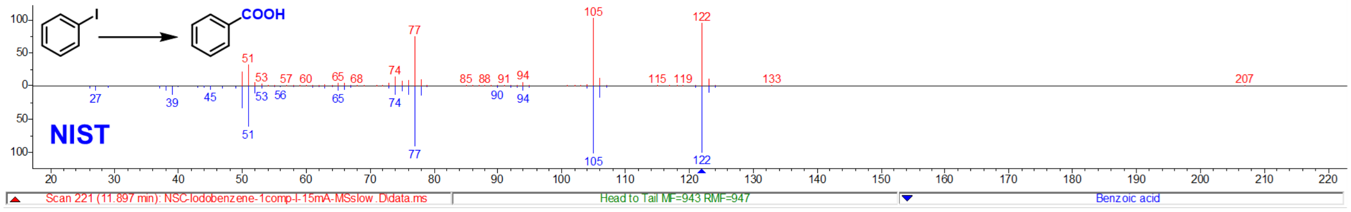

COOH
NIST
Scan 221 (11.897 min): NSC-Iodobenzene-1comp-I-15mA-MSslow .D\data.ms
Head to Tail MF=943 RMF=947
Benzoic acid

13.2 Spectra from Carboxylations with Sacrificial Anodes

4-Phenylbutyric acid (1a)

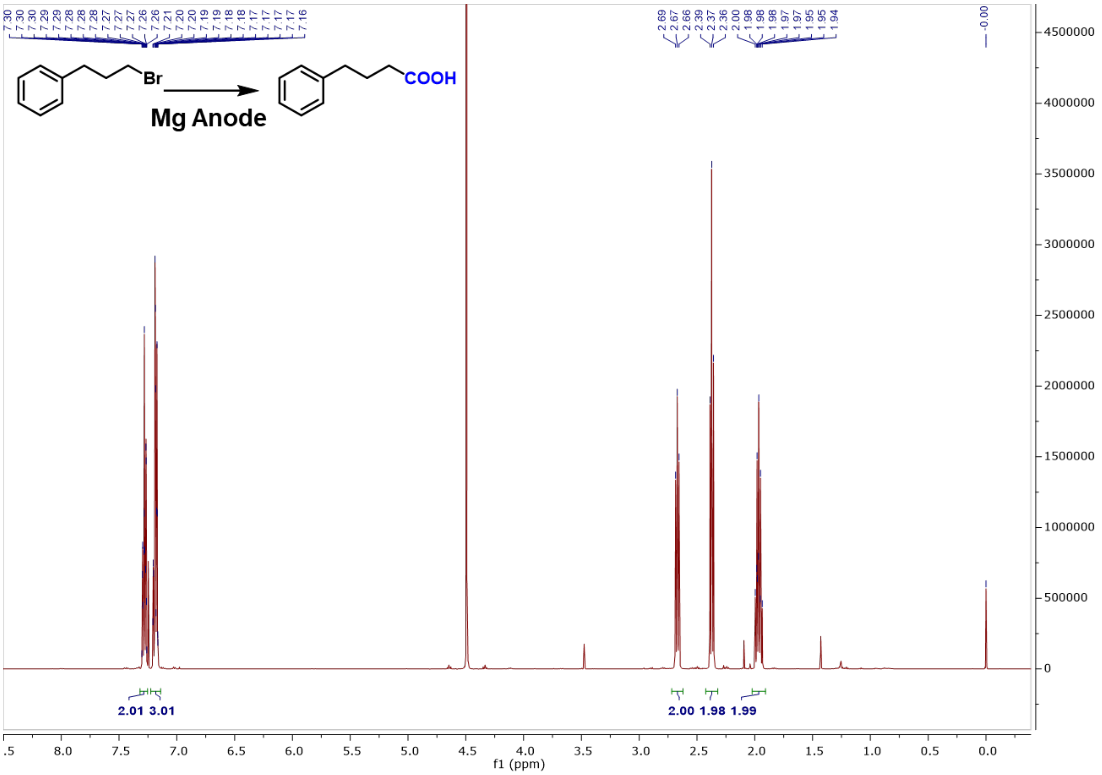

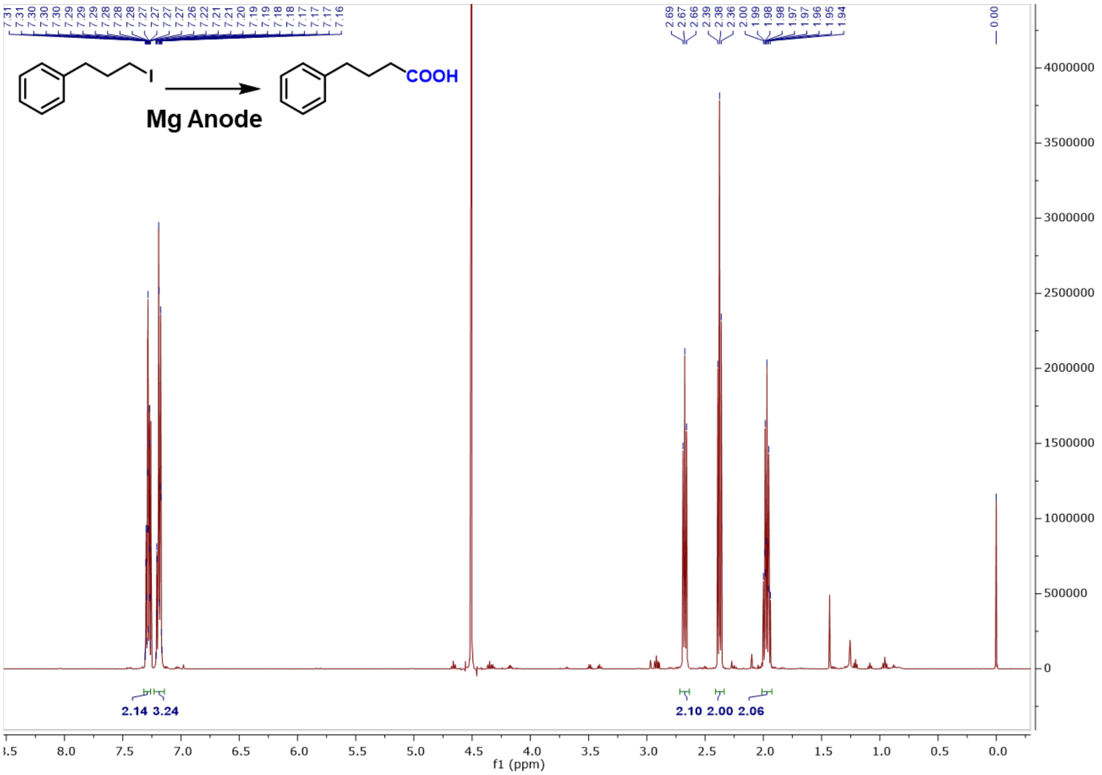

3-Phenylpropanoic acid (2a)

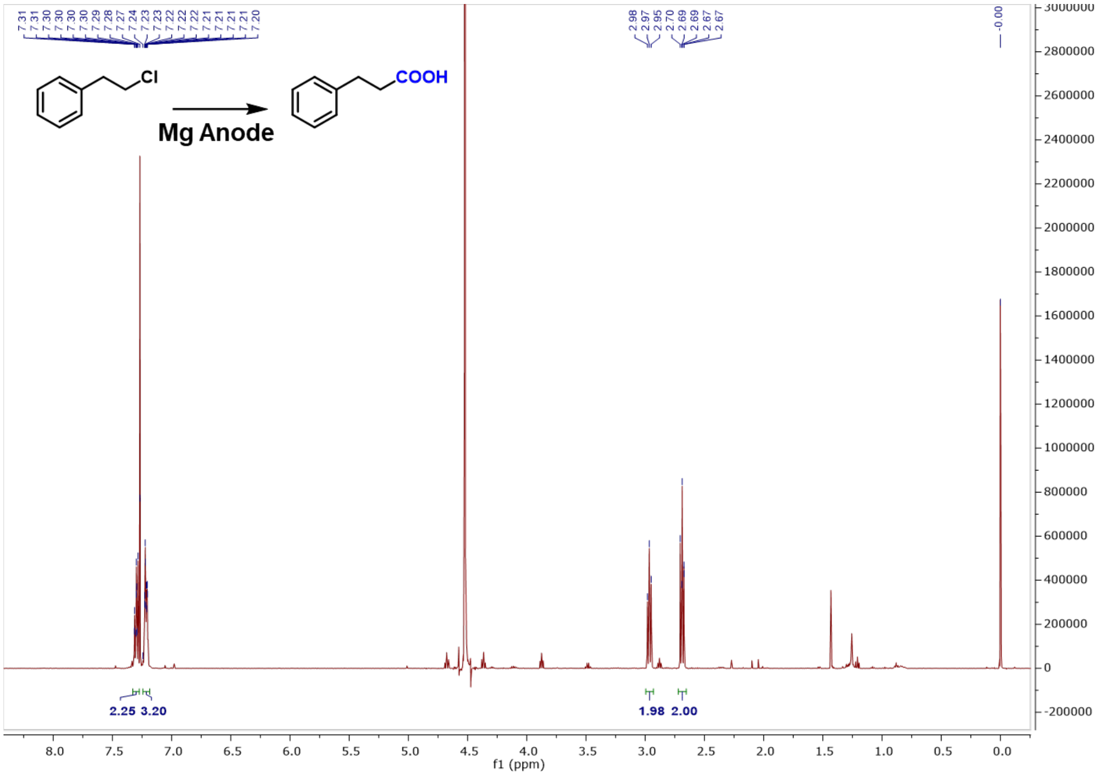

2-Methyl-3-phenylpropanoic acid (6a)

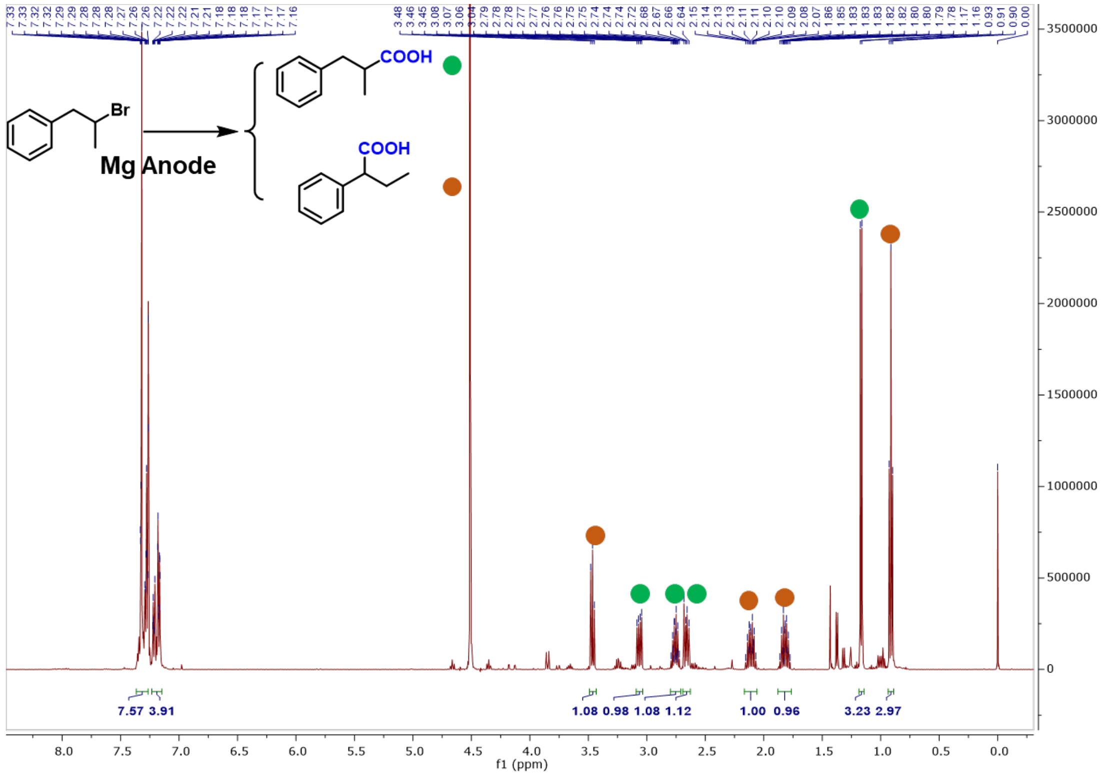

Phenylacetic acid (9a)

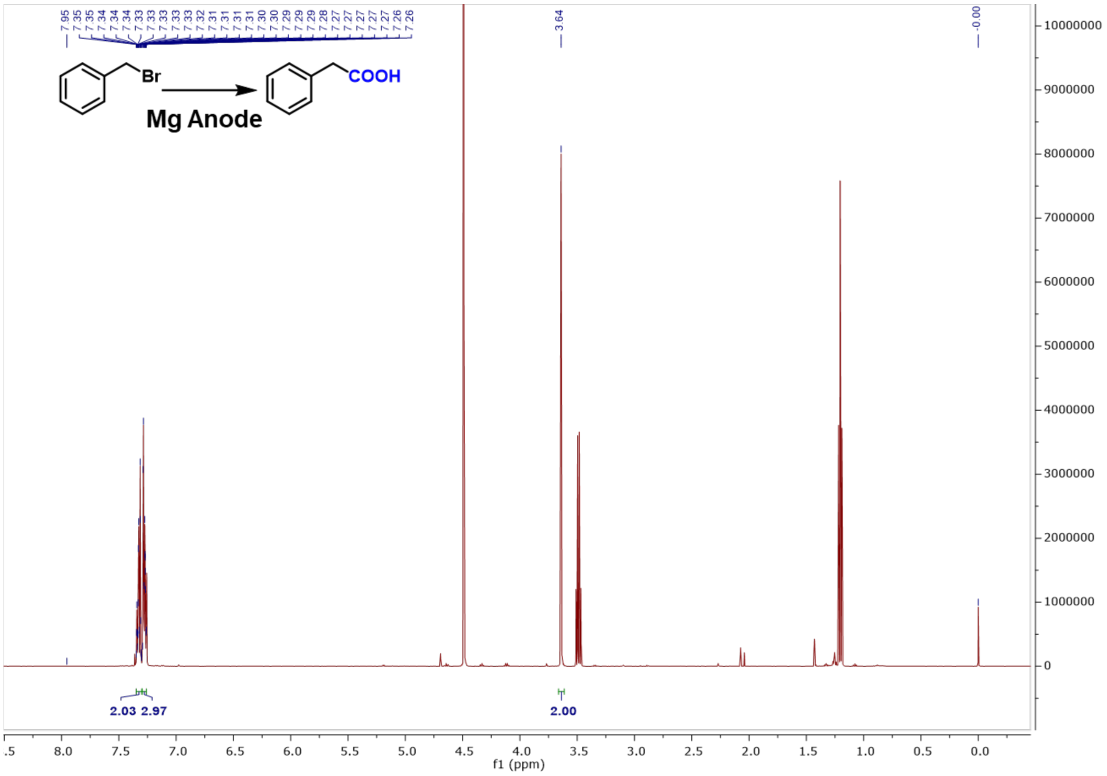

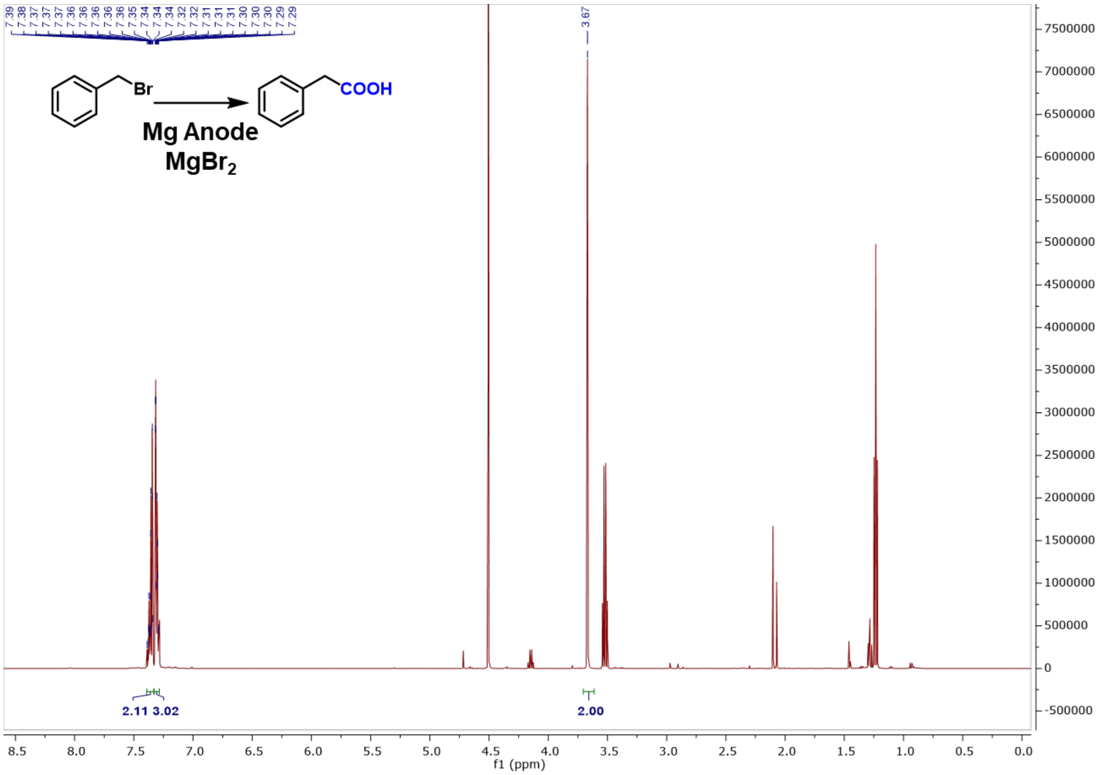

Benzoic acid (18a)

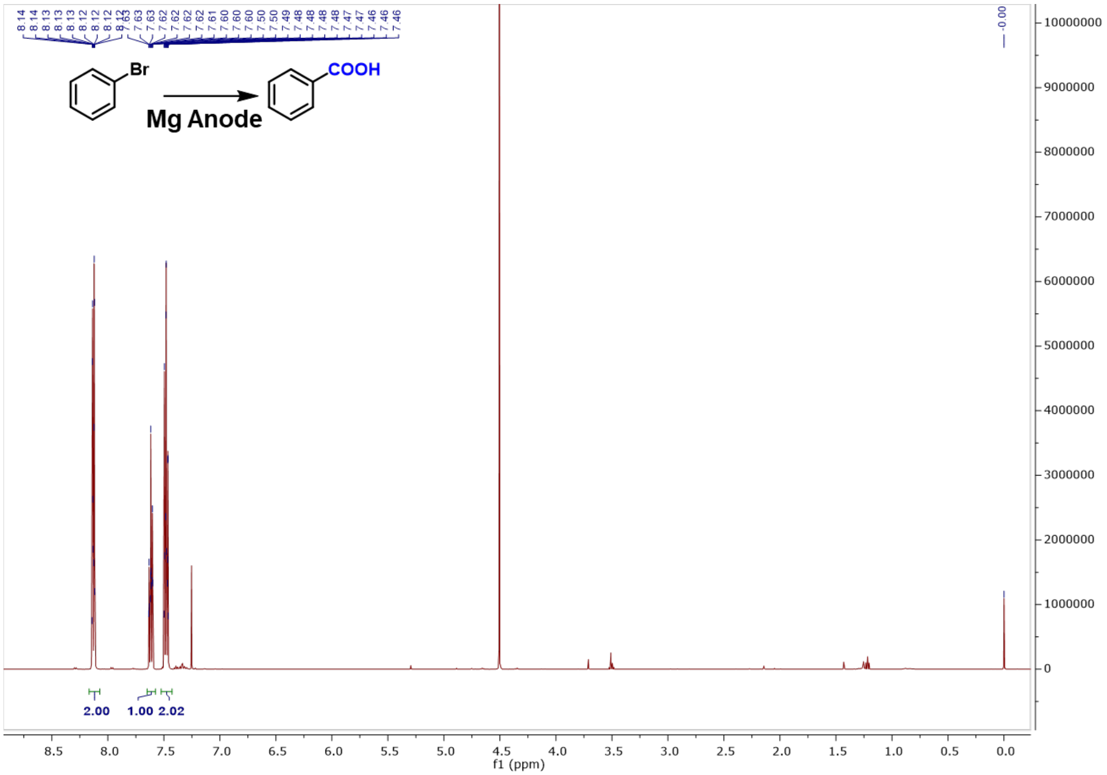

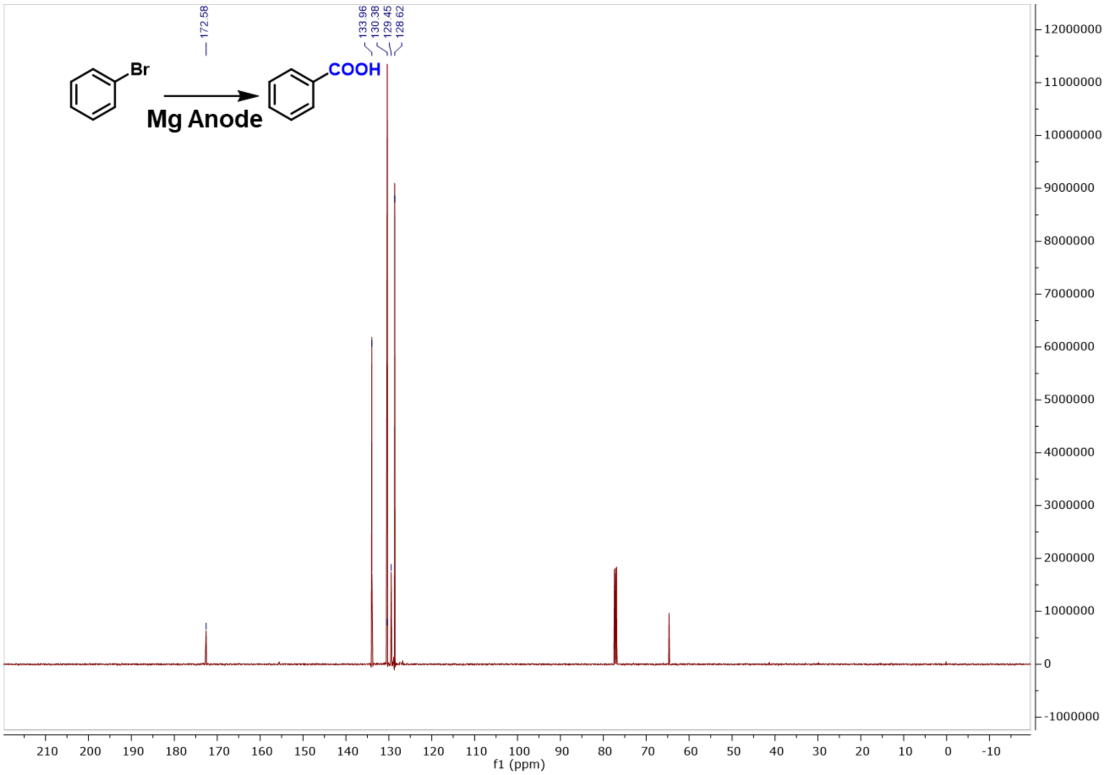

13.3 Spectra from Reactions without Magnesium

1-Bromo-3-phenylpropane

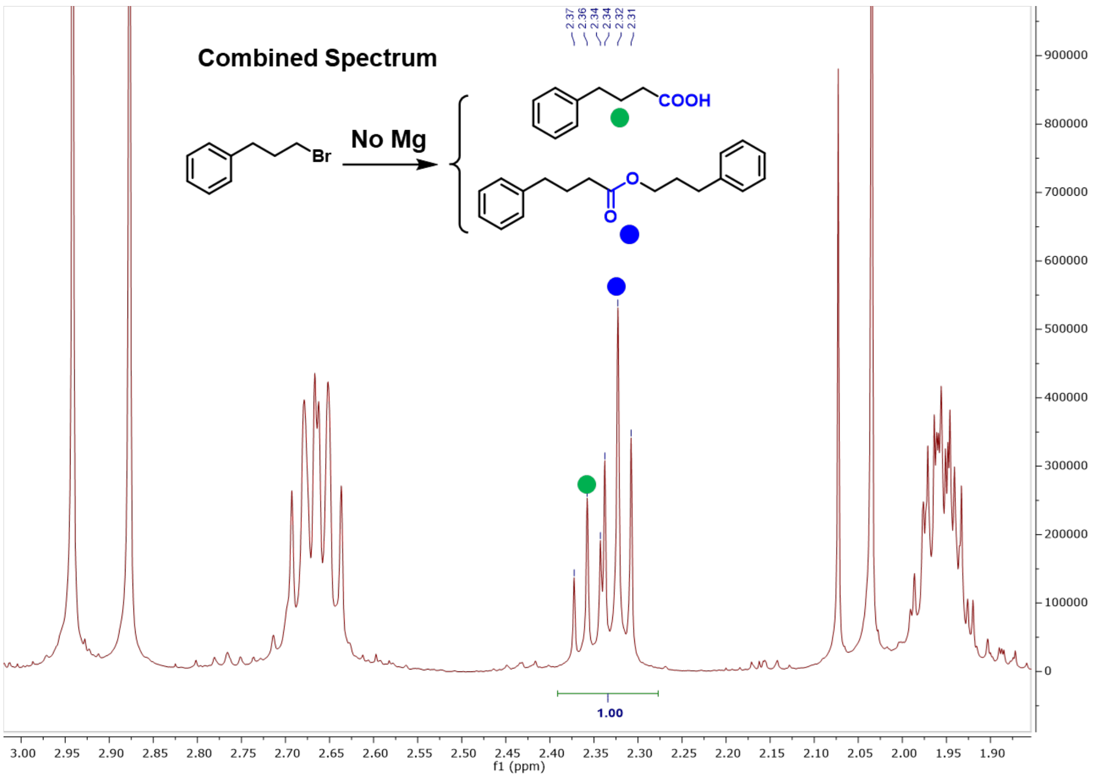

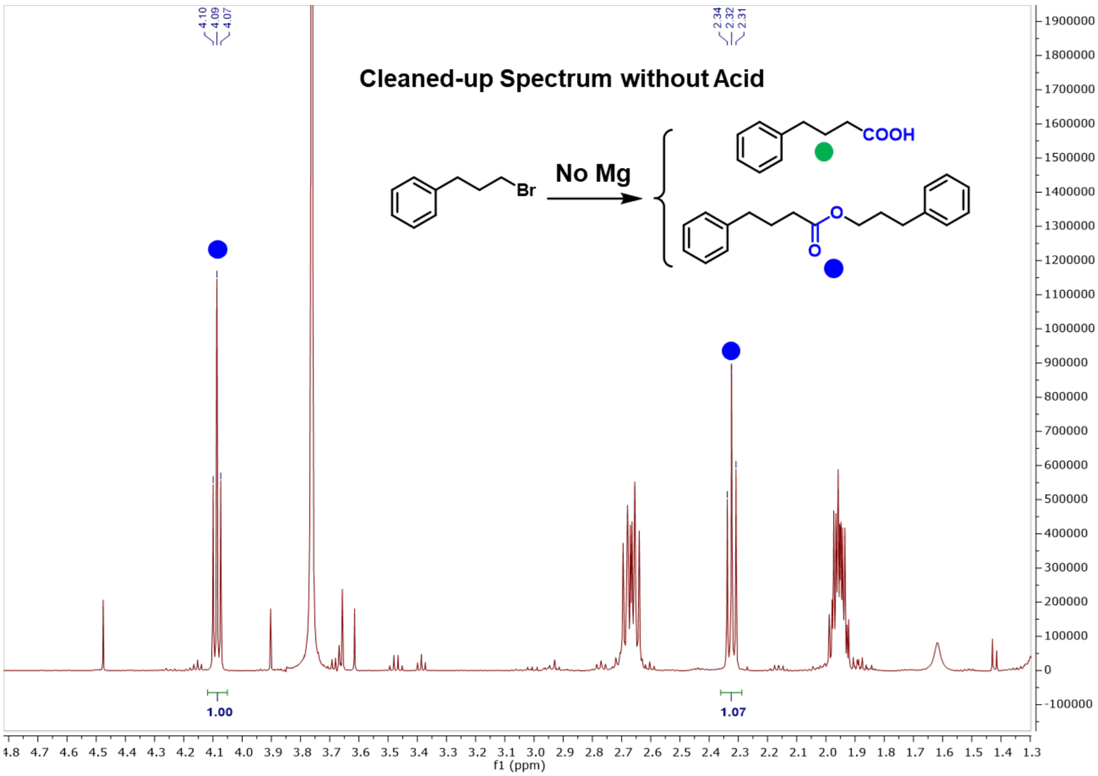

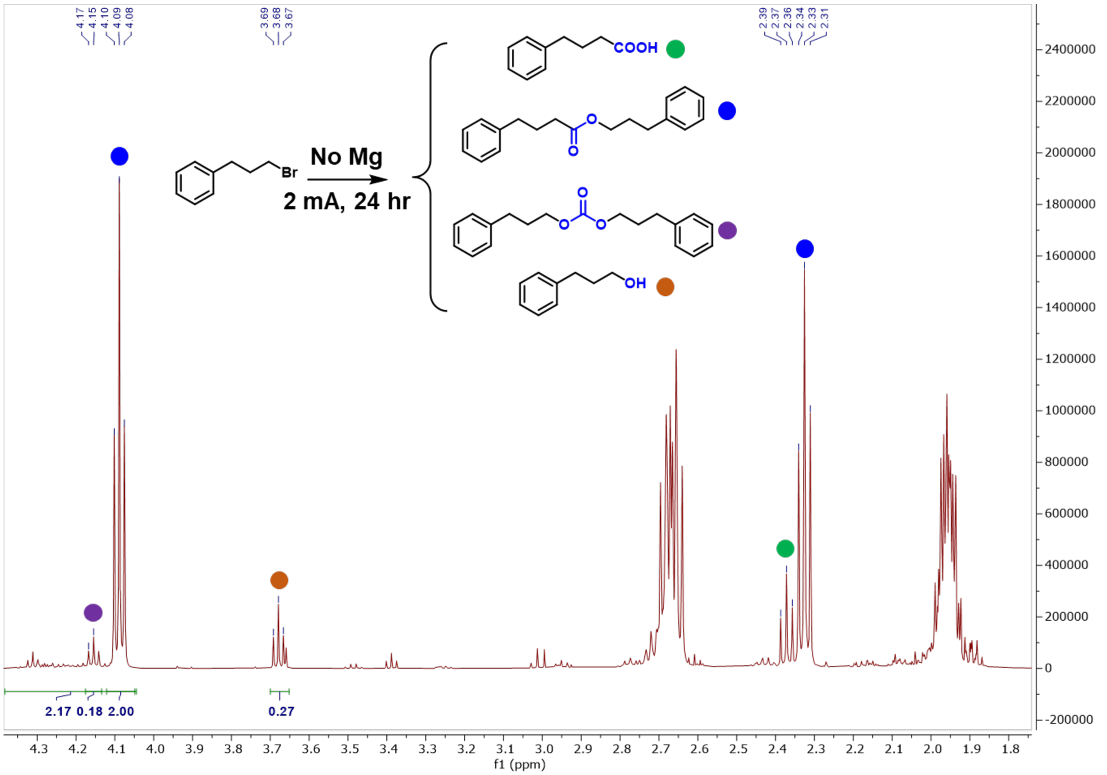

4.17 4.15 4.10 4.09 4.08
3.69 3.68 3.67
2.39 2.37 2.36 2.34 2.33 2.31
No Mg
2 mA, 24 hr
Br
COOH
O
OH
2.17 0.18 2.00
0.27
f1 (ppm)

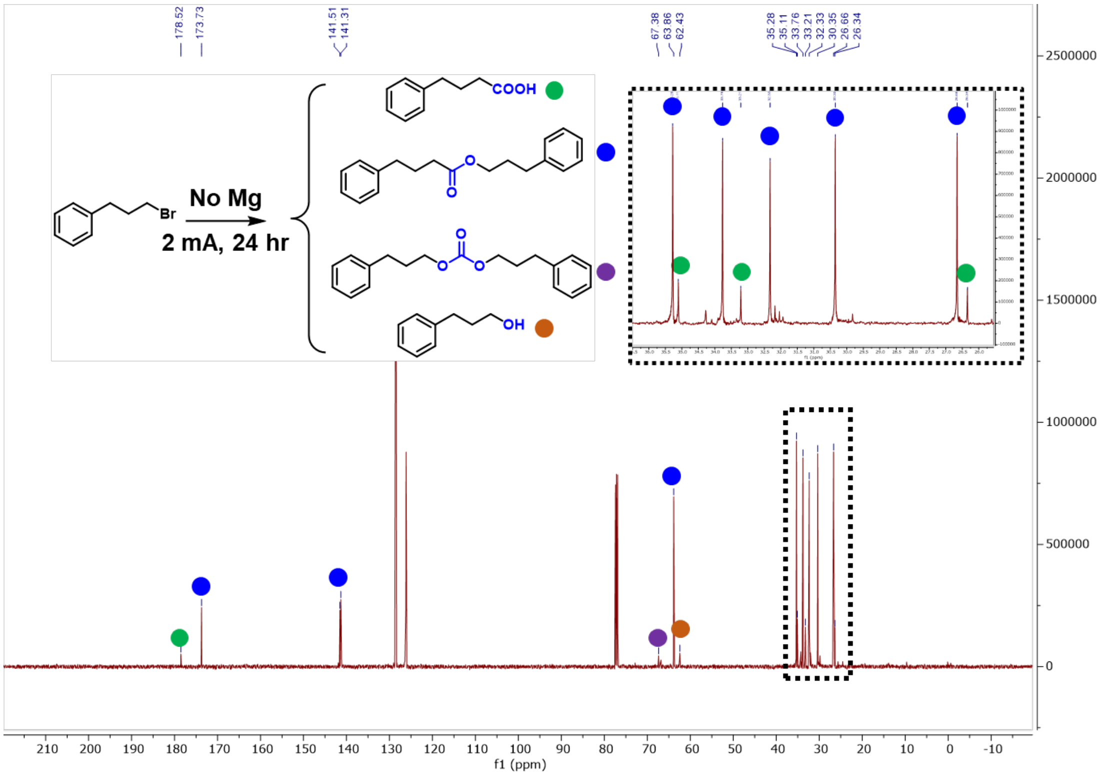

178.52
173.73
141.51 141.31
67.38 63.86 62.43
35.28 35.11 33.76 33.21 32.33 30.35 26.66 26.34
No Mg
2 mA, 24 hr
Br
COOH
O
OH
f1 (ppm)

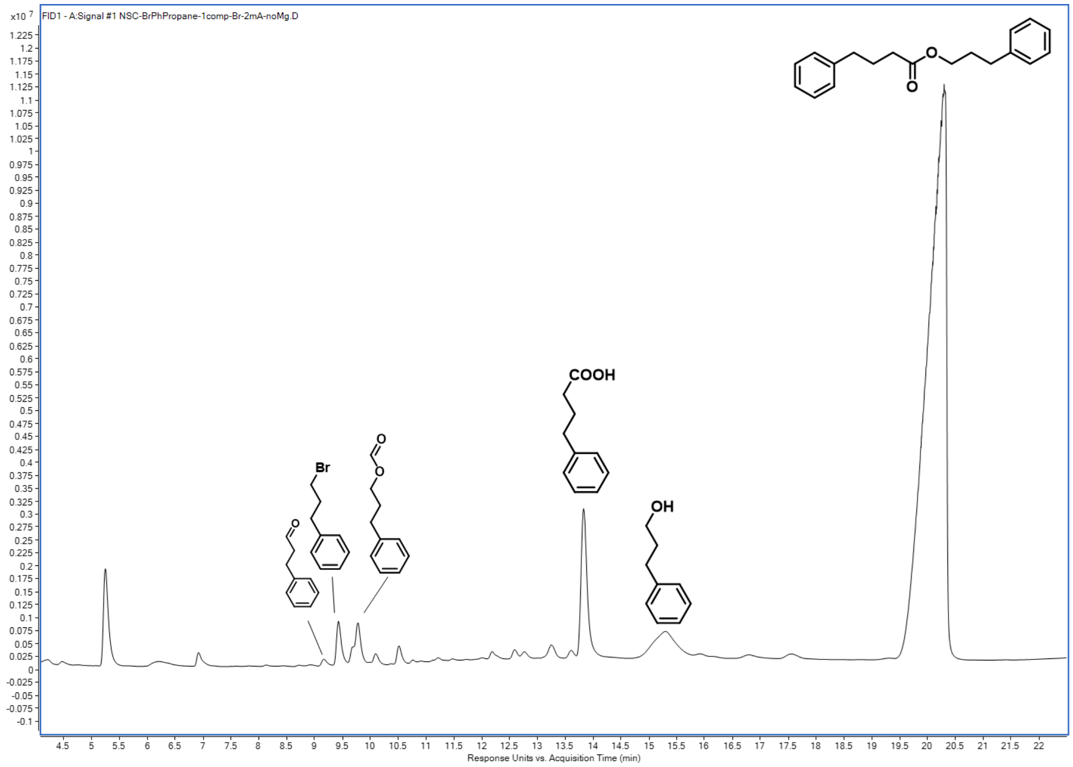
FID1 - A:Signal #1 NSC-BrPhPropane-1comp-Br-2mA-noMg.D
x10 7
Br
COOH
OH
Response Units vs. Acquisition Time (min)

1-Iodo-3-phenylpropane

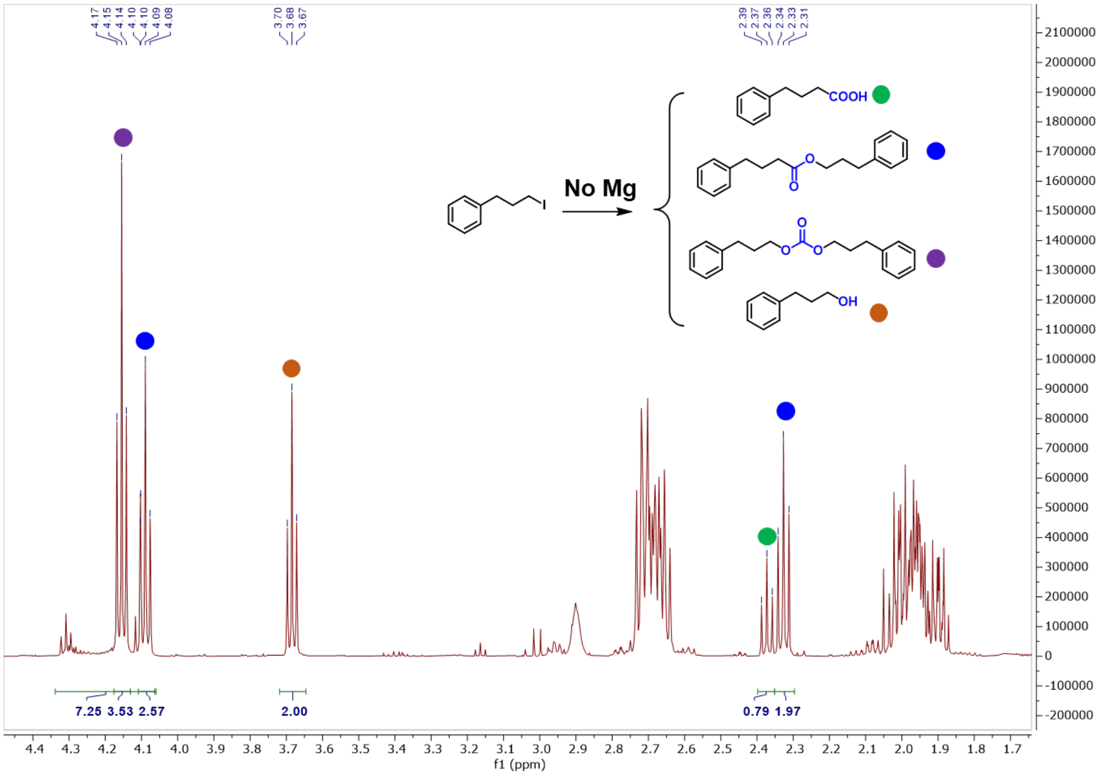

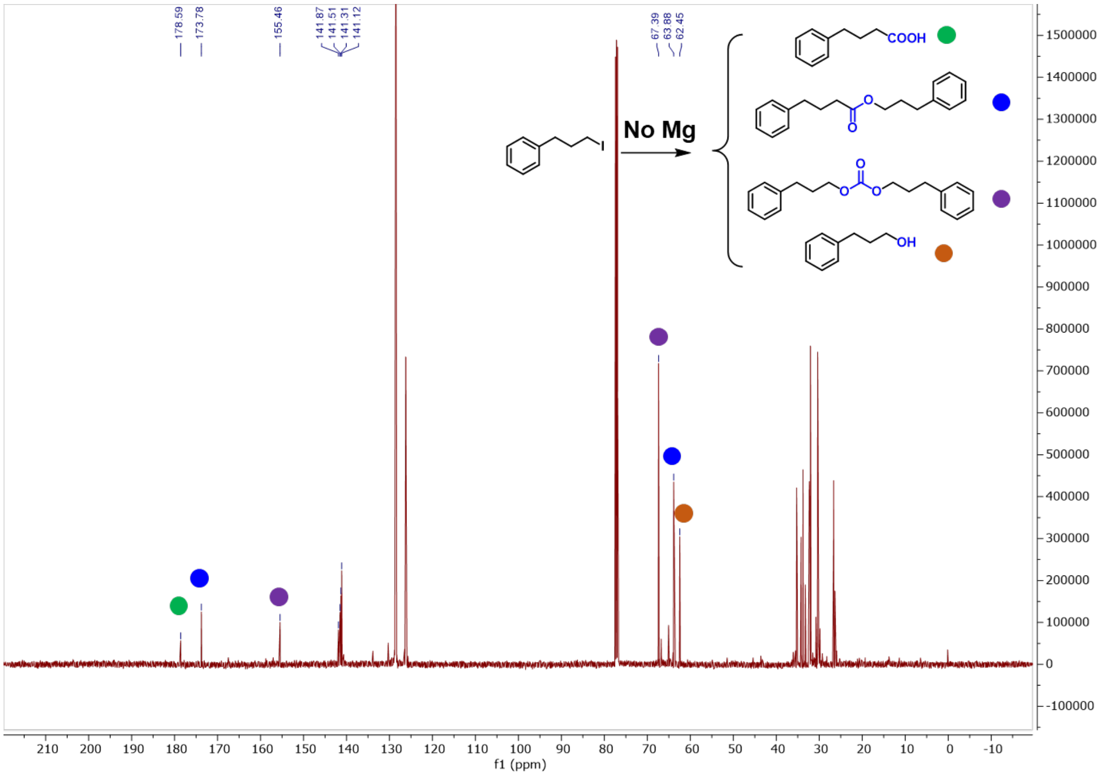

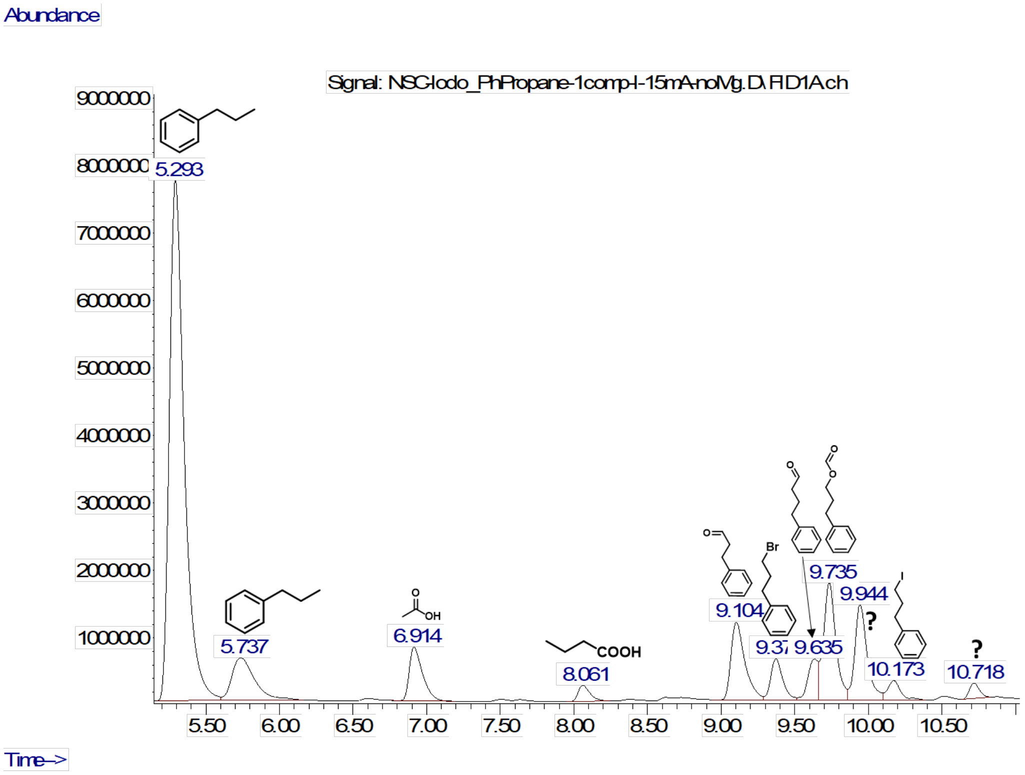

Abundance
Signal: NSC-Iodo_PhPropane-1comp-I-15mA-noMg.D\FID1A.ch
5.293
5.737
6.914
8.061
9.104
9.635
9.735
9.944
10.173
10.718
Time-->

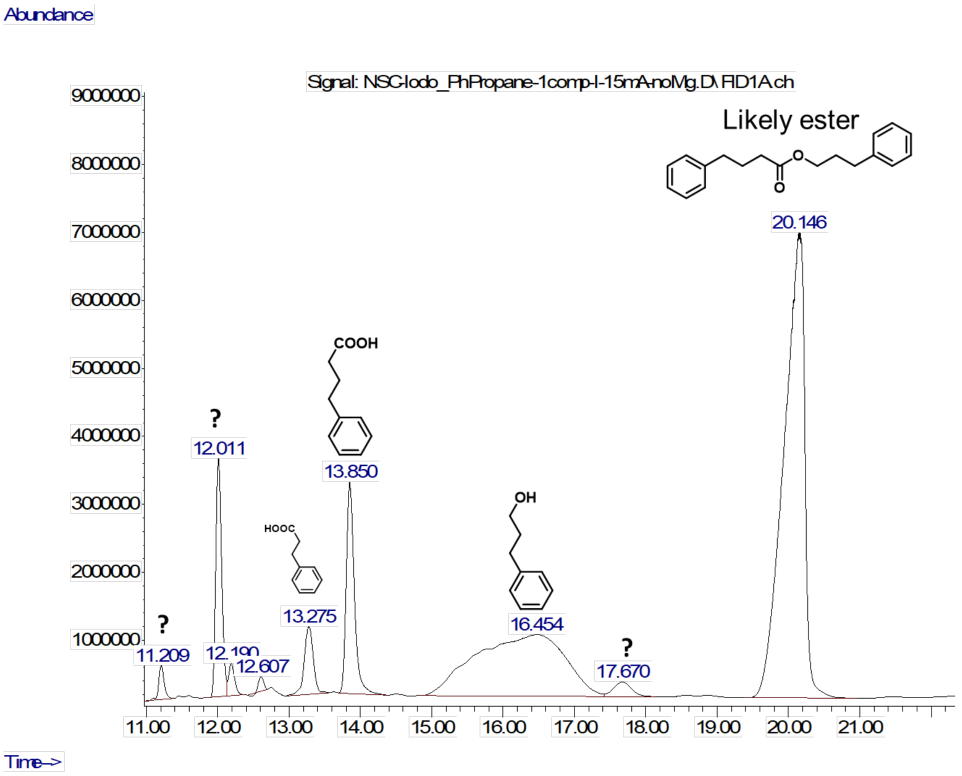

Abundance
Signal: NSC-Iodo_PhPropane-1comp-I-15mA-noMg.D\FID1A.ch
Likely ester
20.146
12.011
13.850
13.275
16.454
11.209
12.607
17.670
Time-->

(2-Chloroethyl)benzene

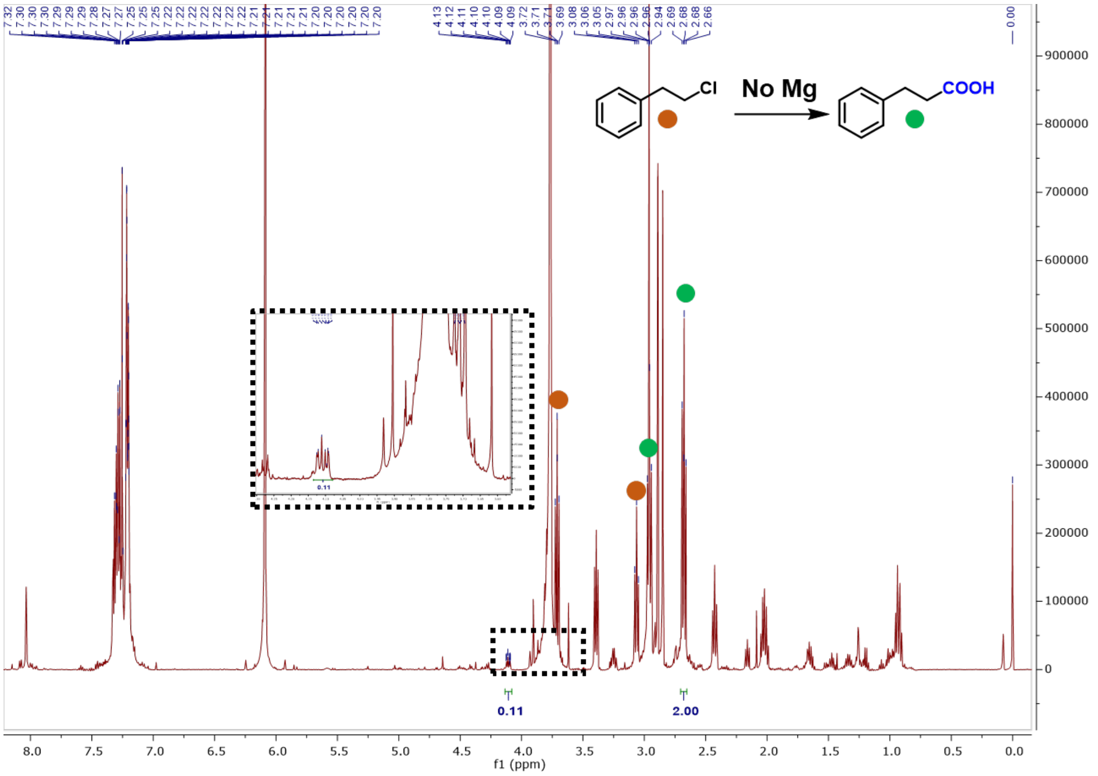

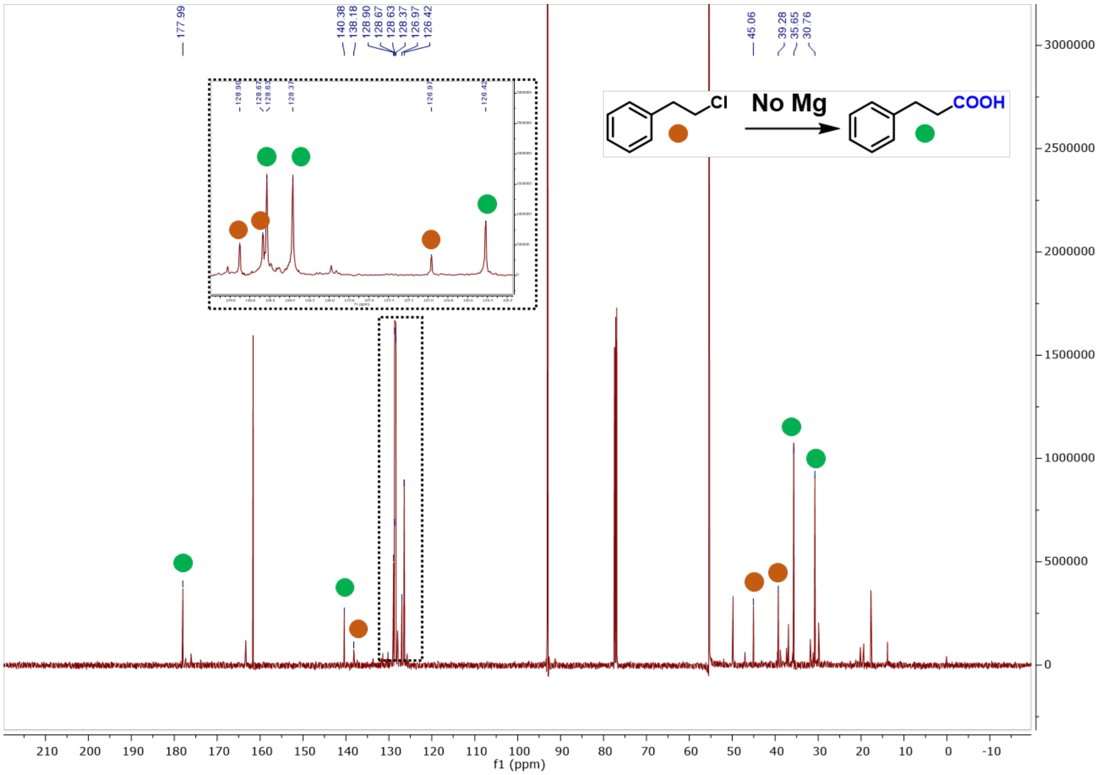

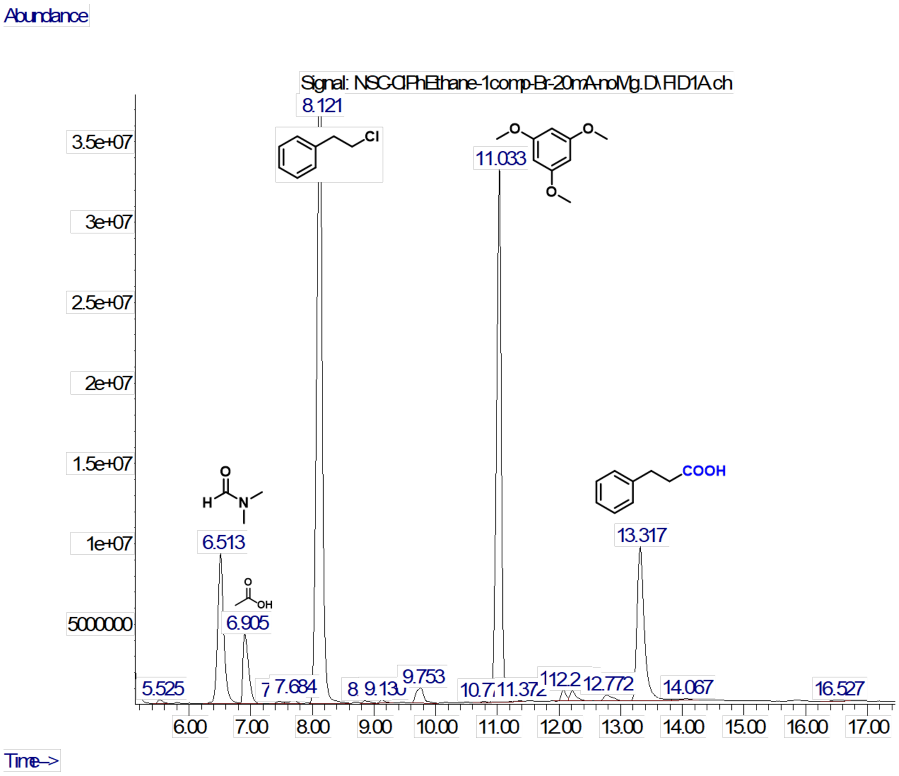
Abundance
Signal: NSC-ClPhEthane-1comp-Br-20mA-noMg.D\FID1A.ch
3.5e+07
3e+07
2.5e+07
2e+07
1.5e+07
1e+07
5000000
8.121
11.033
13.317
6.513
6.905
5.525
7.684
9.753
12.772
14.067
16.527
COOH
Cl
OH
6.00
7.00
8.00
9.00
10.00
11.00
12.00
13.00
14.00
15.00
16.00
17.00
Time-->

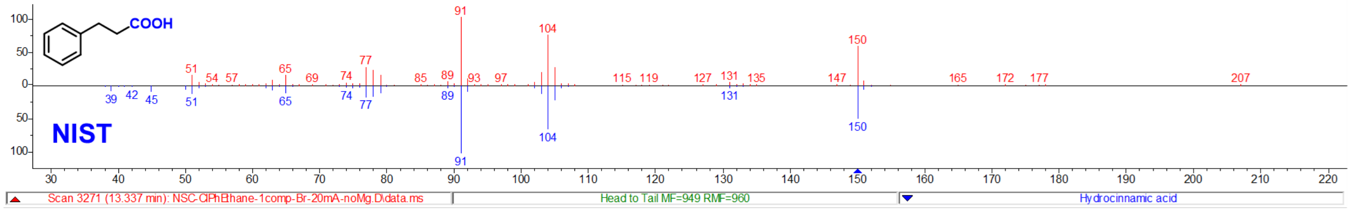
COOH
NIST
Scan 3271 (13.337 min): NSC-ClPhEthane-1comp-Br-20mA-noMg.D\data.ms
Head to Tail MF=949 RMF=960
Hydrocinnamic acid

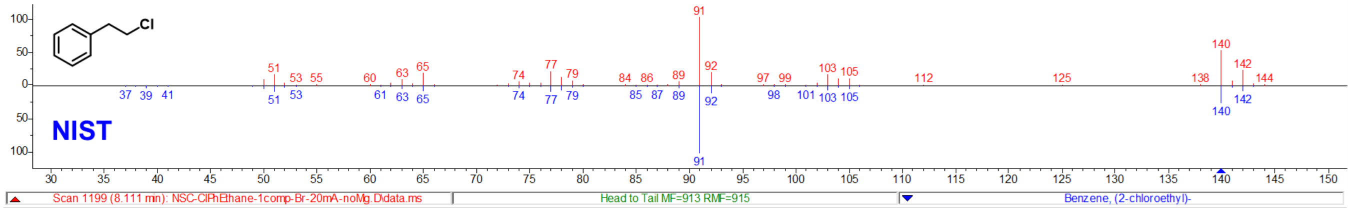
Cl
NIST
Scan 1199 (8.111 min): NSC-ClPhEthane-1comp-Br-20mA-noMg.D\data.ms
Head to Tail MF=913 RMF=915
Benzene, (2-chloroethyl)-

Bromocyclohexane

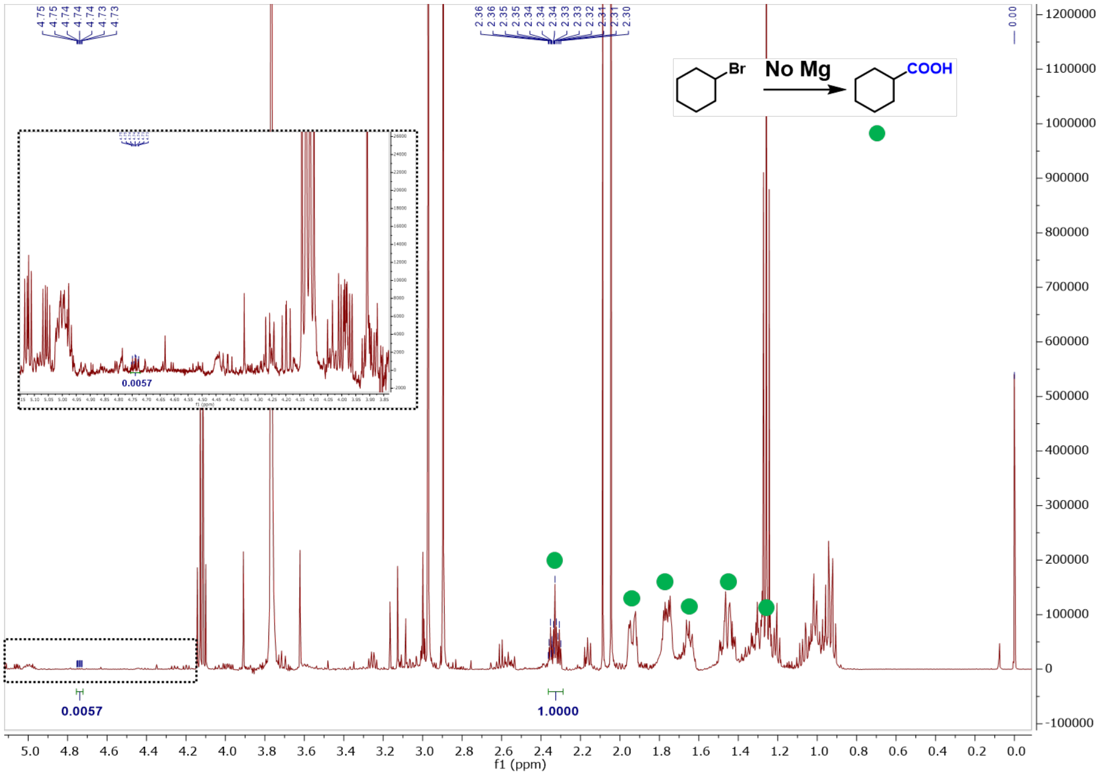

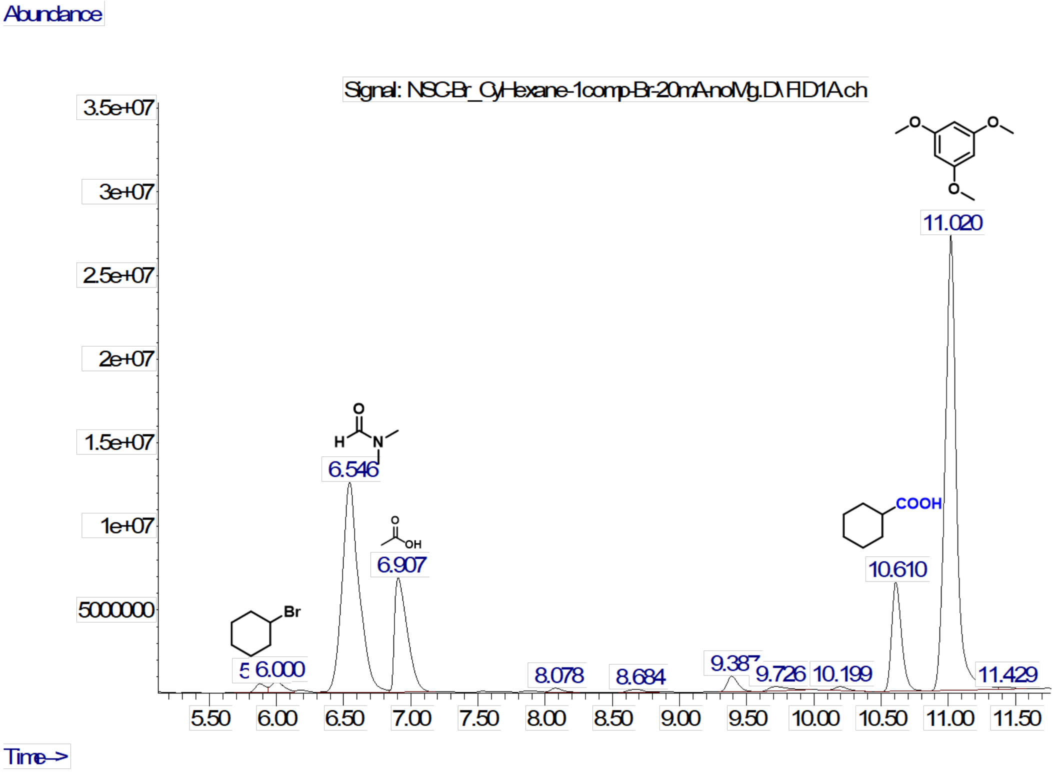

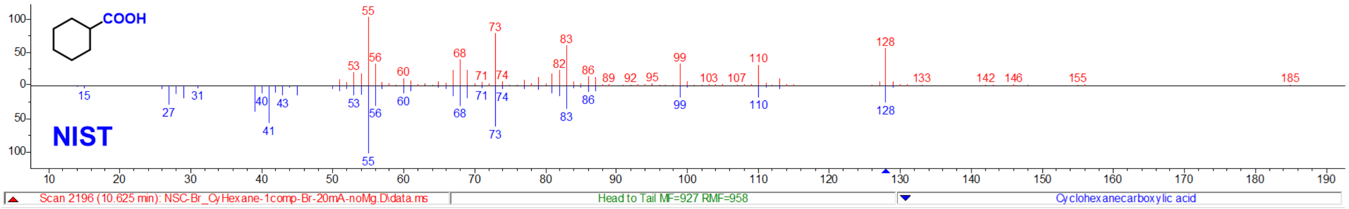
COOH
NIST
Scan 2196 (10.625 min): NSC-Br_CyHexane-1comp-Br-20mA-noMg.D\data.ms
Head to Tail MF=927 RMF=958
Cyclohexanecarboxylic acid

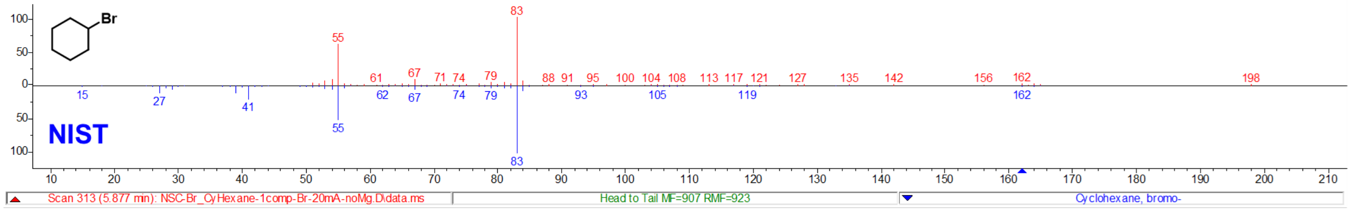
Br
NIST
Scan 313 (5.877 min): NSC-Br_CyHexane-1comp-Br-20mA-noMg.D\data.ms
Head to Tail MF=907 RMF=923
Cyclohexane, bromo-

Iodocyclohexane

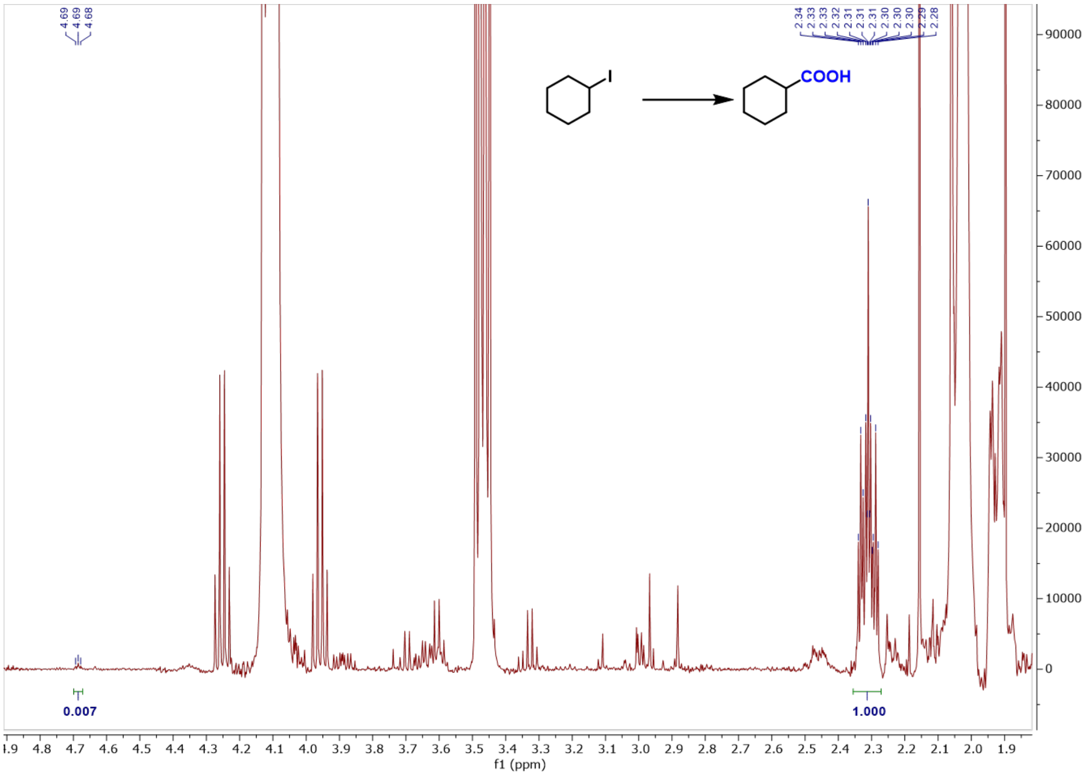

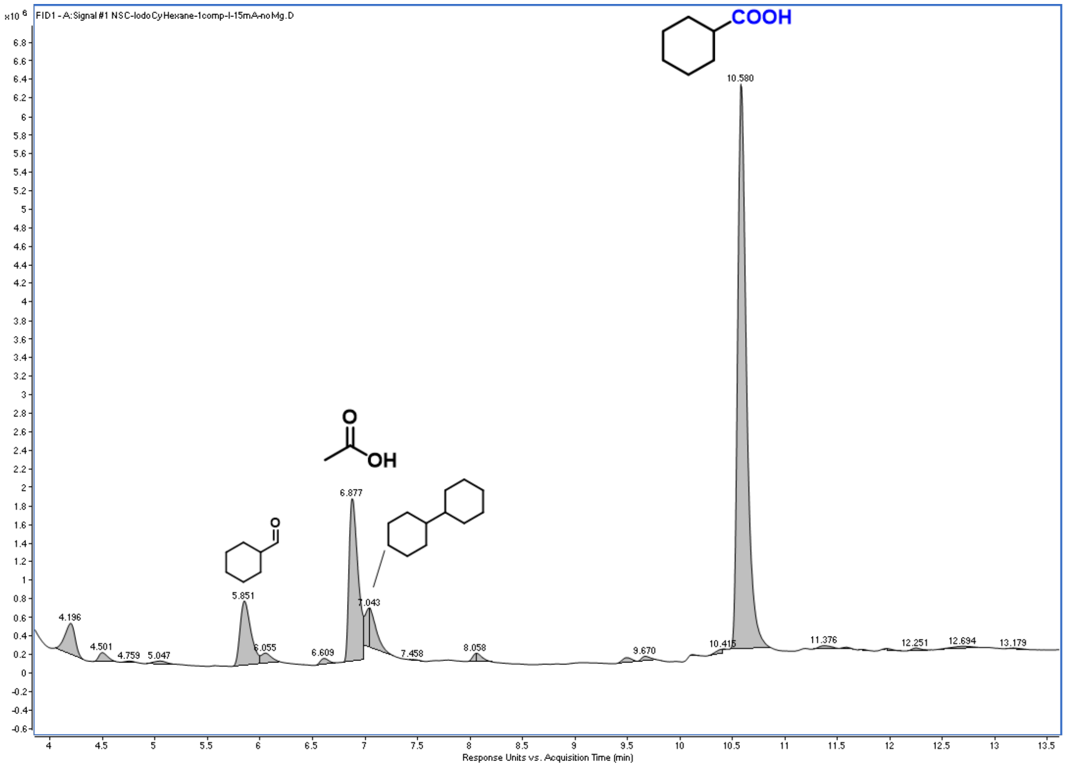

1-Bromo-5-chloropentane

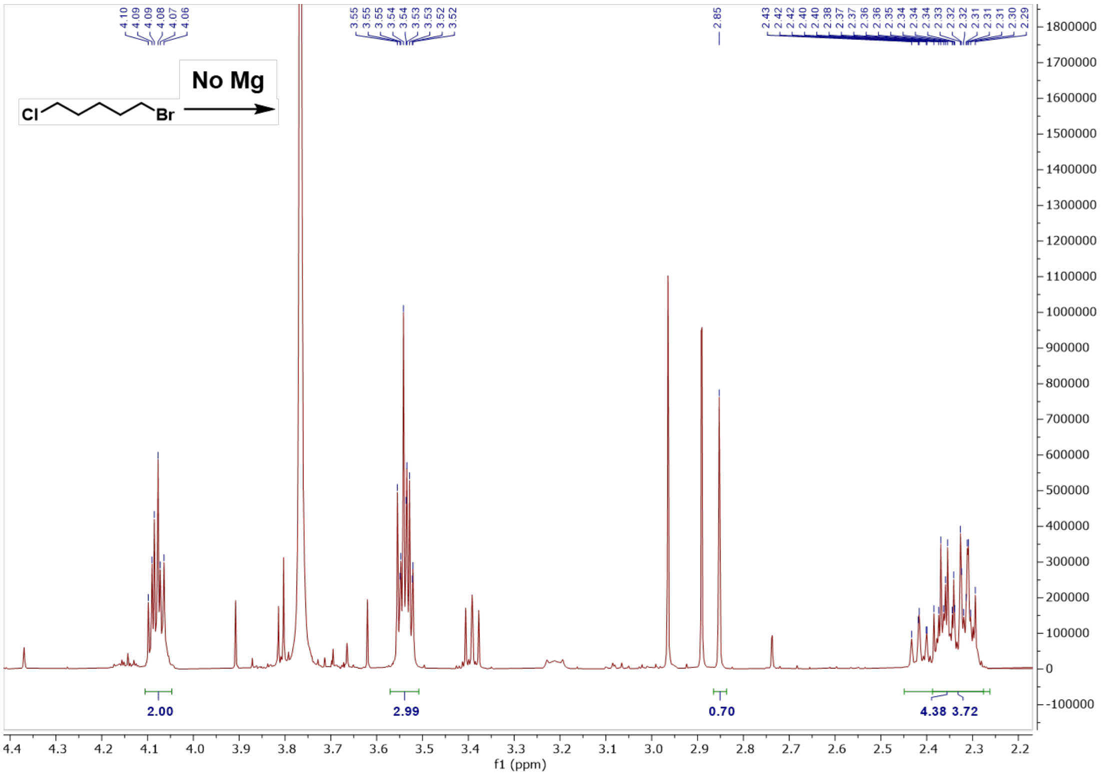

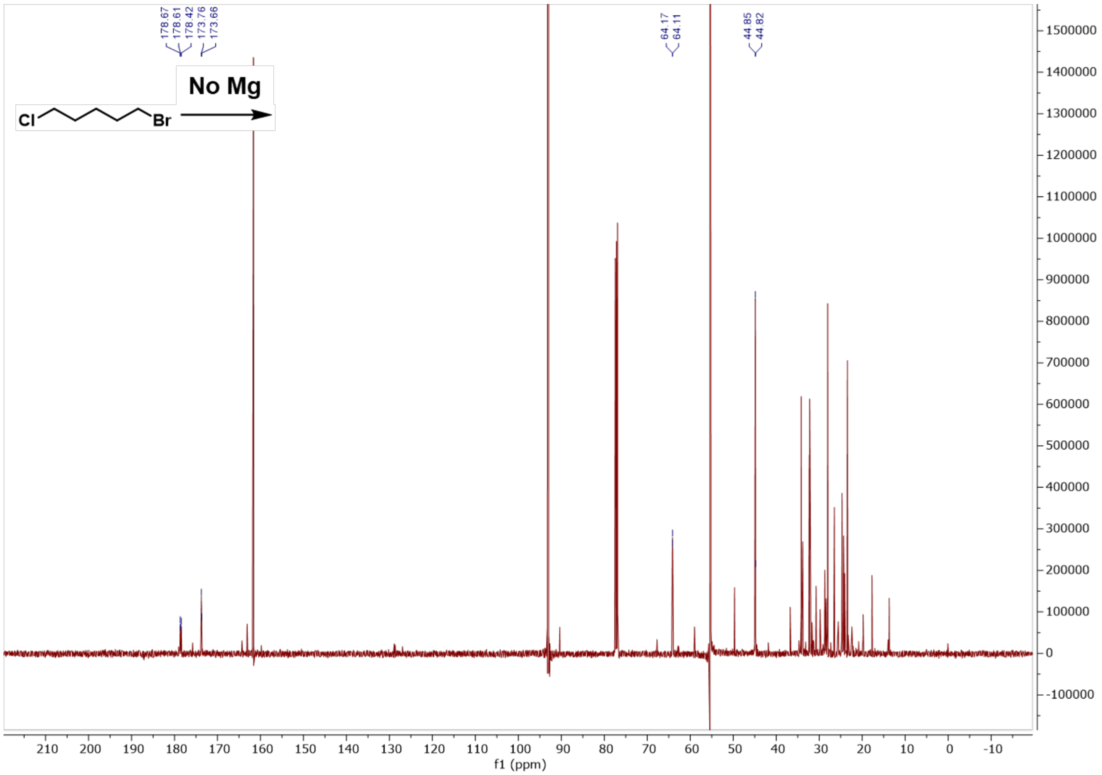

2-Bromo-1-phenylpropane

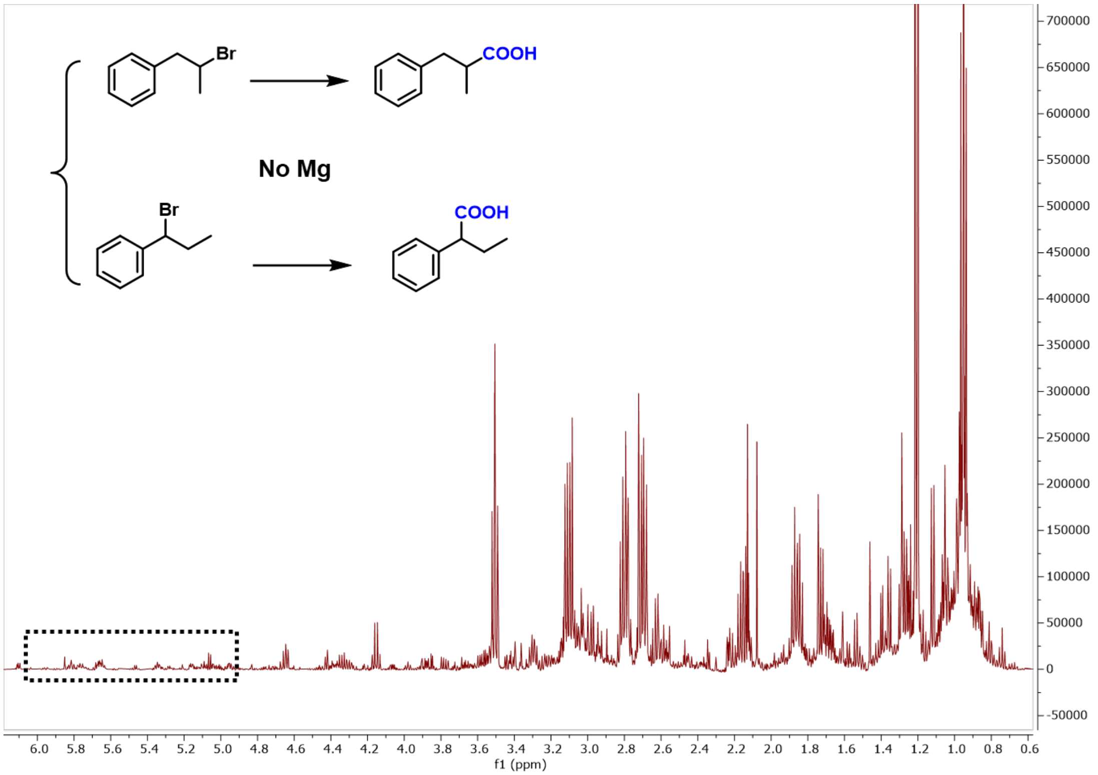

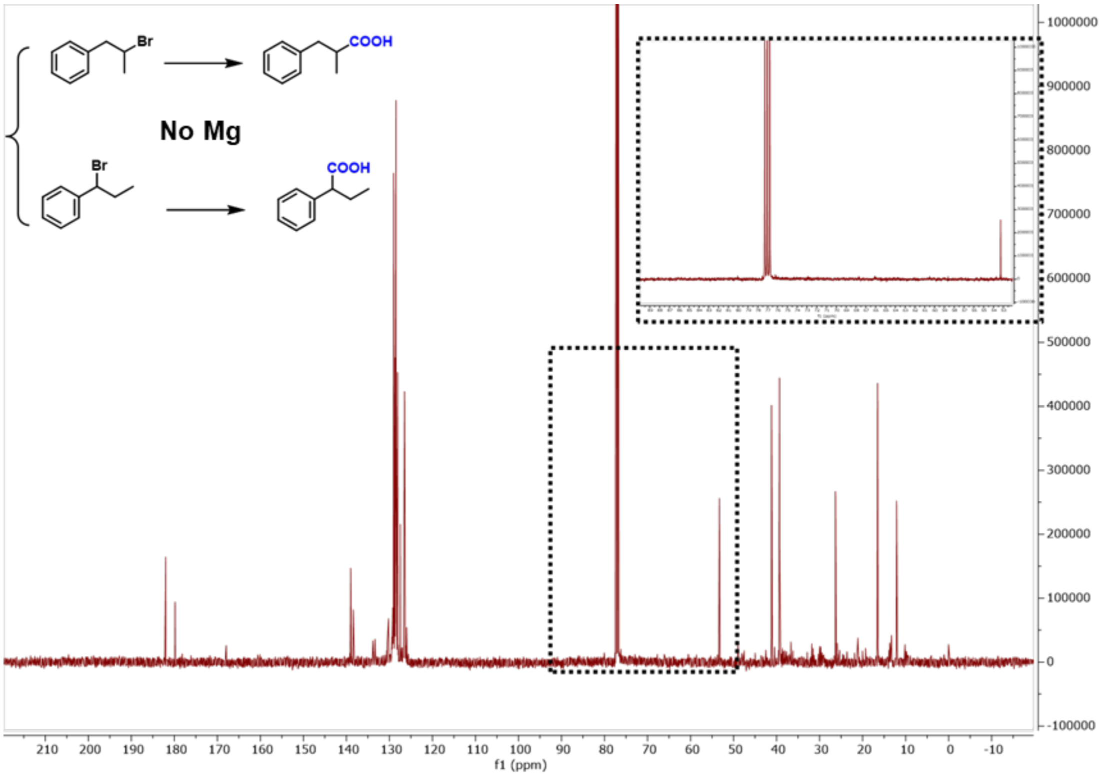

(1-Bromoethyl)benzene

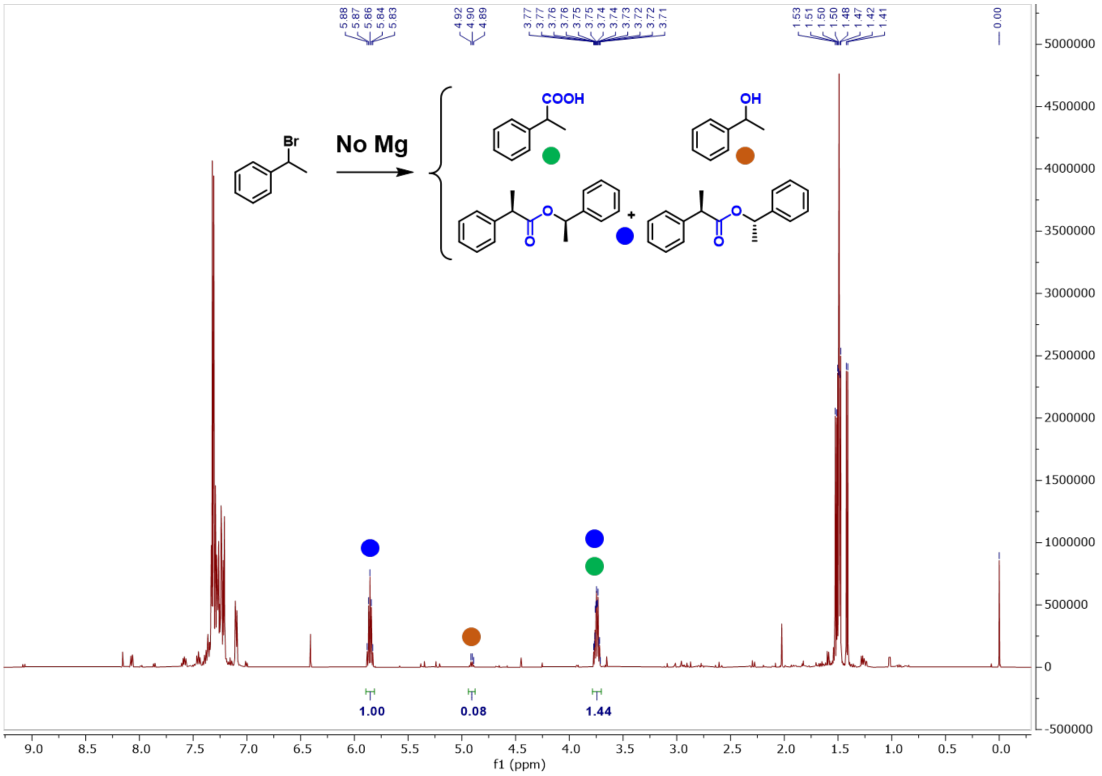

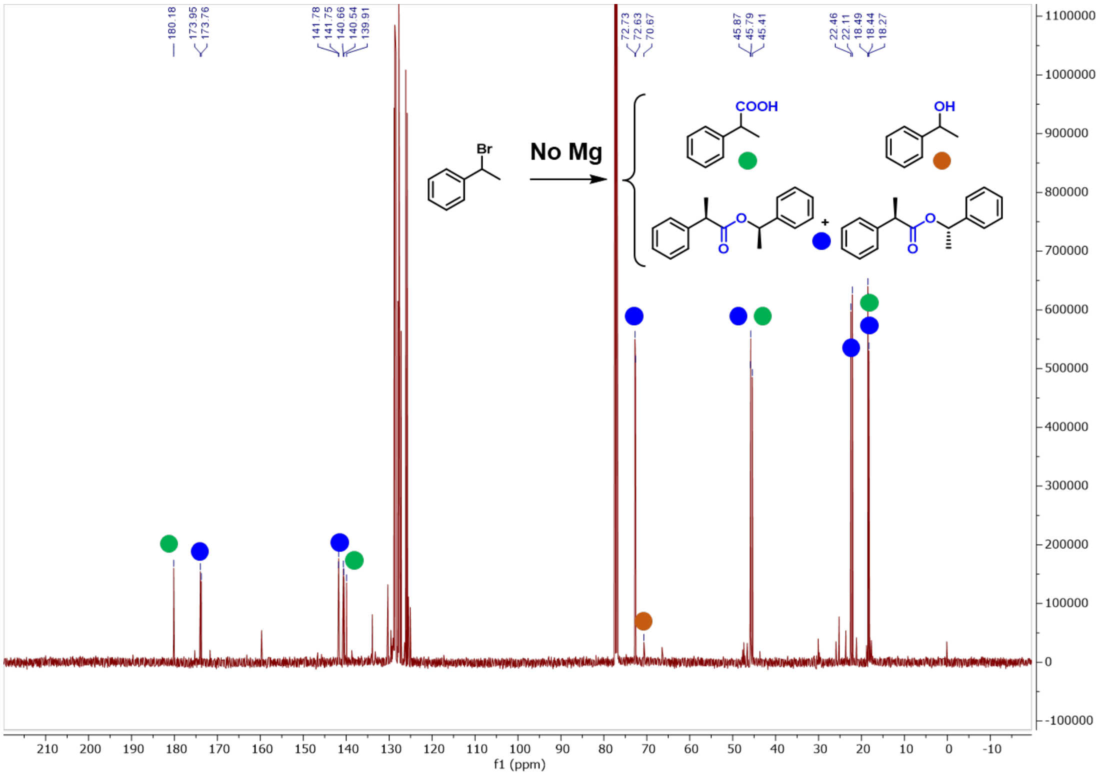

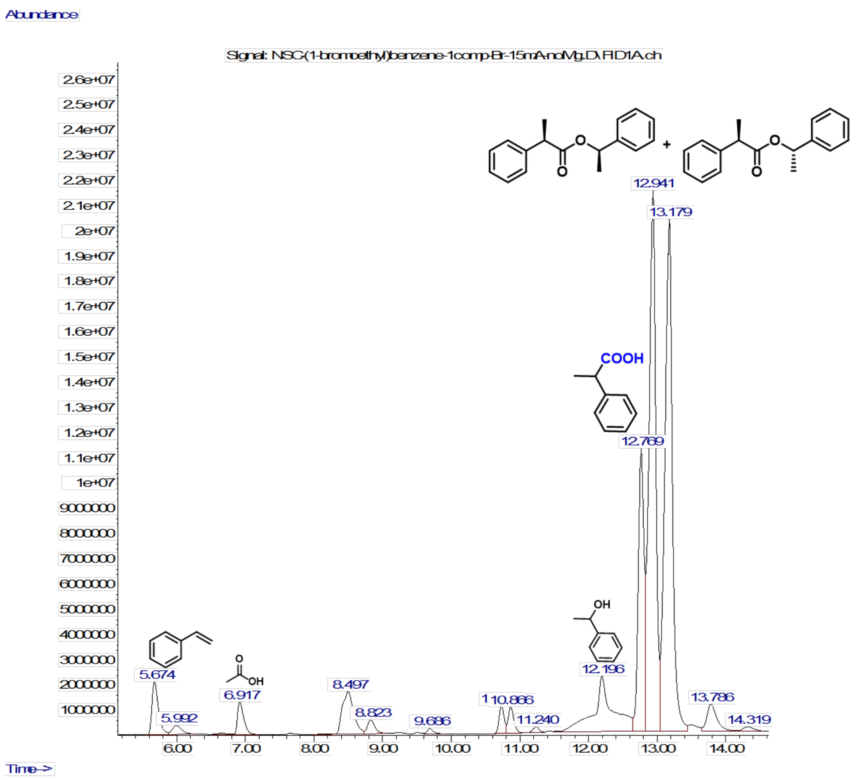
Abundance
Signal: NSC(1-bromoethyl)benzene-1comp-Br-15mAnoMg.D\FID1A.ch
2.6e+07
2.5e+07
2.4e+07
2.3e+07
2.2e+07
2.1e+07
2e+07
1.9e+07
1.8e+07
1.7e+07
1.6e+07
1.5e+07
1.4e+07
1.3e+07
1.2e+07
1.1e+07
1e+07
9000000
8000000
7000000
6000000
5000000
4000000
3000000
2000000
1000000
5.674
5.992
6.917
8.497
8.823
9.686
10.866
11.240
12.196
12.769
12.941
13.179
13.786
14.319
COOH
OH
6.00
7.00
8.00
9.00
10.00
11.00
12.00
13.00
14.00
Time-->

4-Methyl bezyl chloride

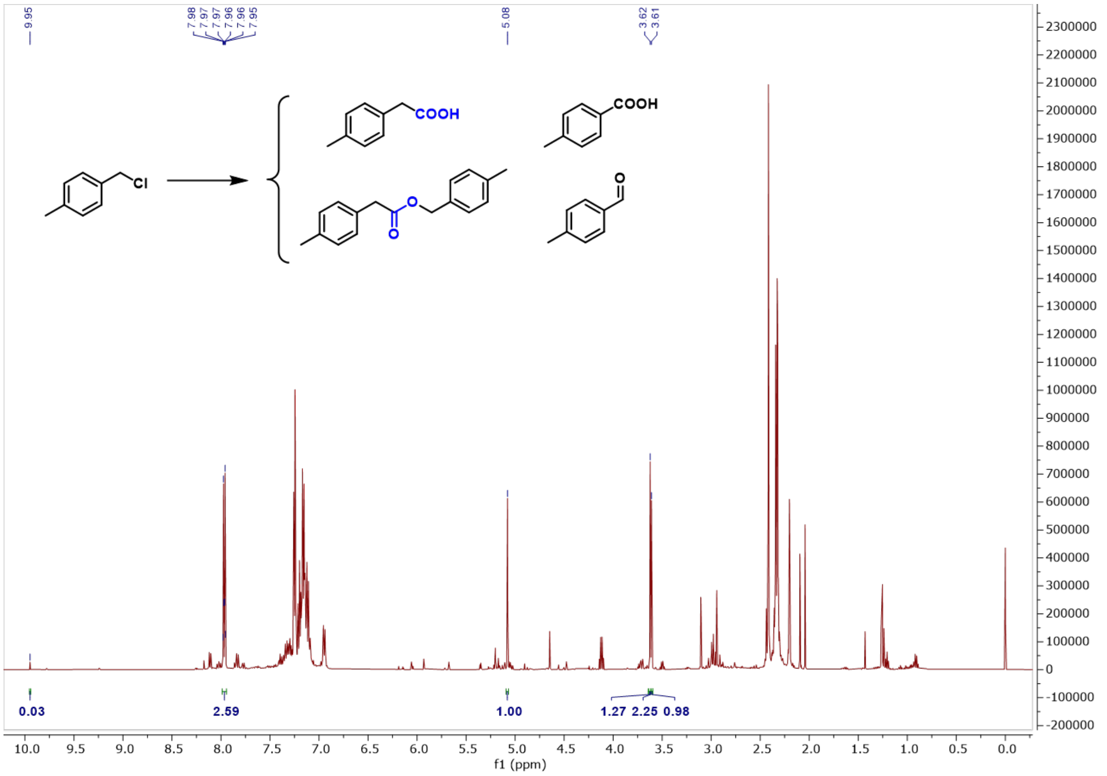

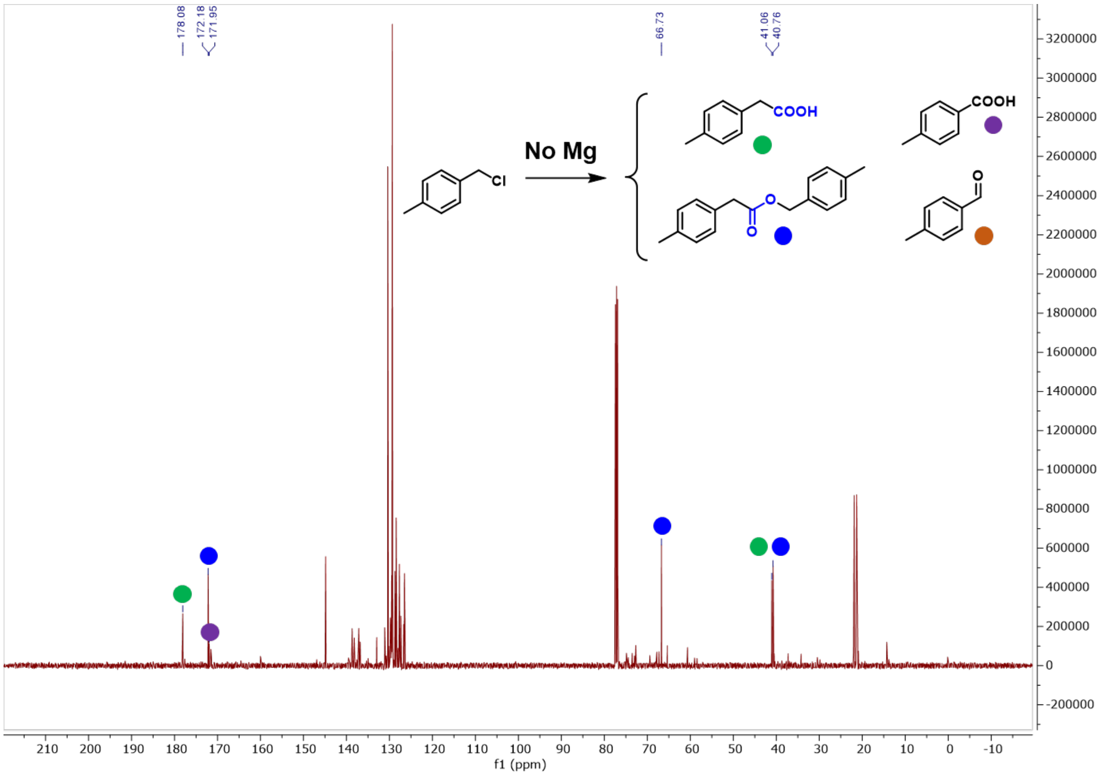

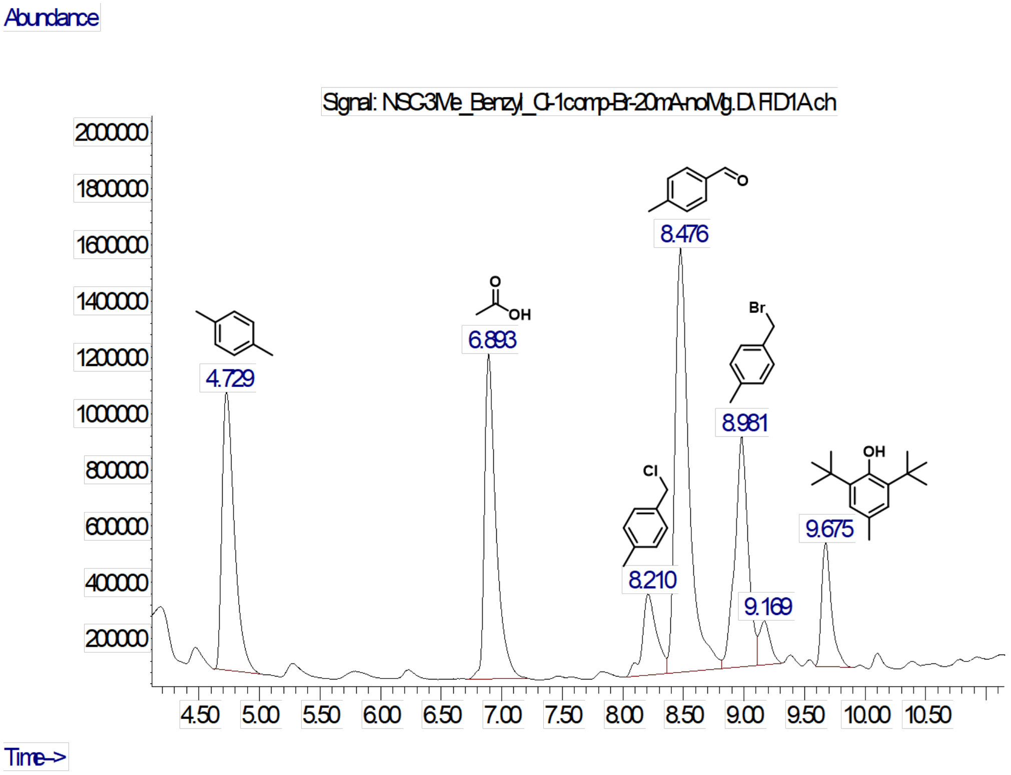
Abundance
Signal: NSC3Me_Benzyl_Cl-1comp-Br-20mA-noMg.D\ FID1A.ch
4.729
6.893
8.210
8.476
8.981
9.169
9.675
Time->

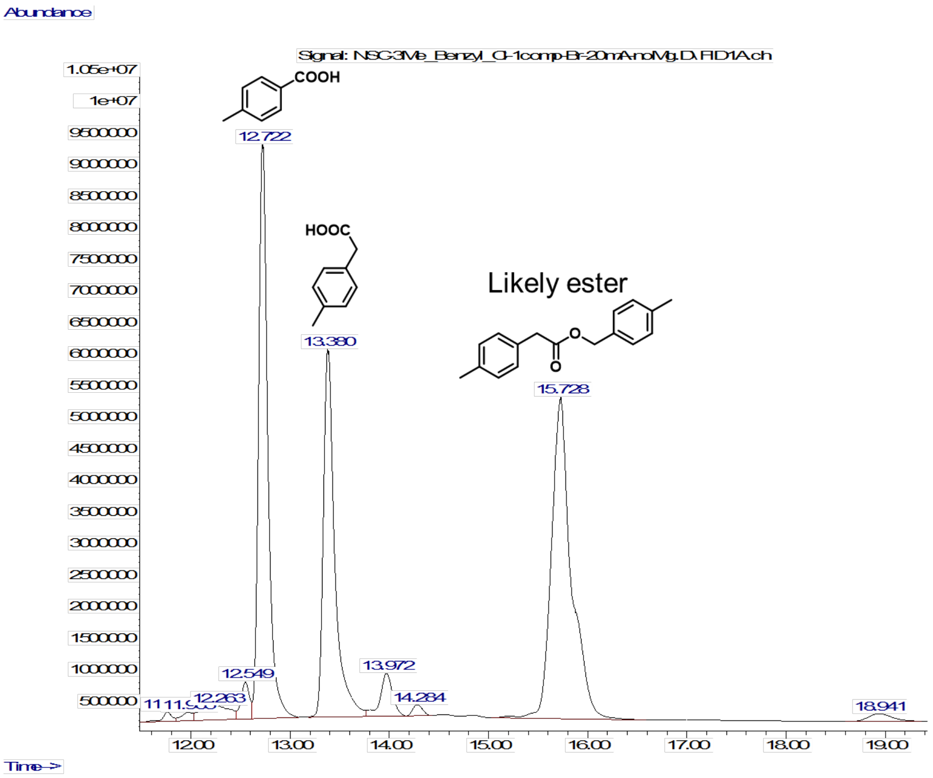
Abundance
Signal: NSC3Me_Benzyl_Cl-1comp-Br-20mA-noMg.D\ FID1A.ch
COOH
12.722
HOOC
13.380
Likely ester
15.728
12.549
12.263
13.972
14.284
18.941
Time->

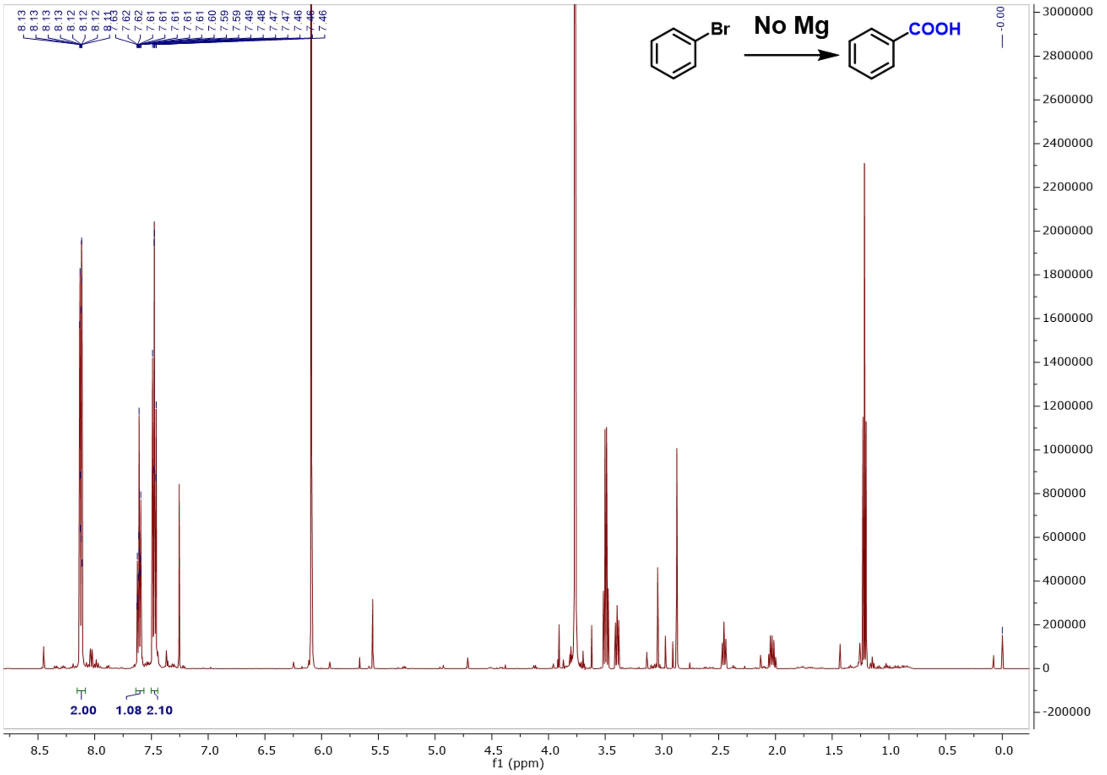
No Mg
Br
COOH
f1 (ppm)
2.00
1.08
2.10

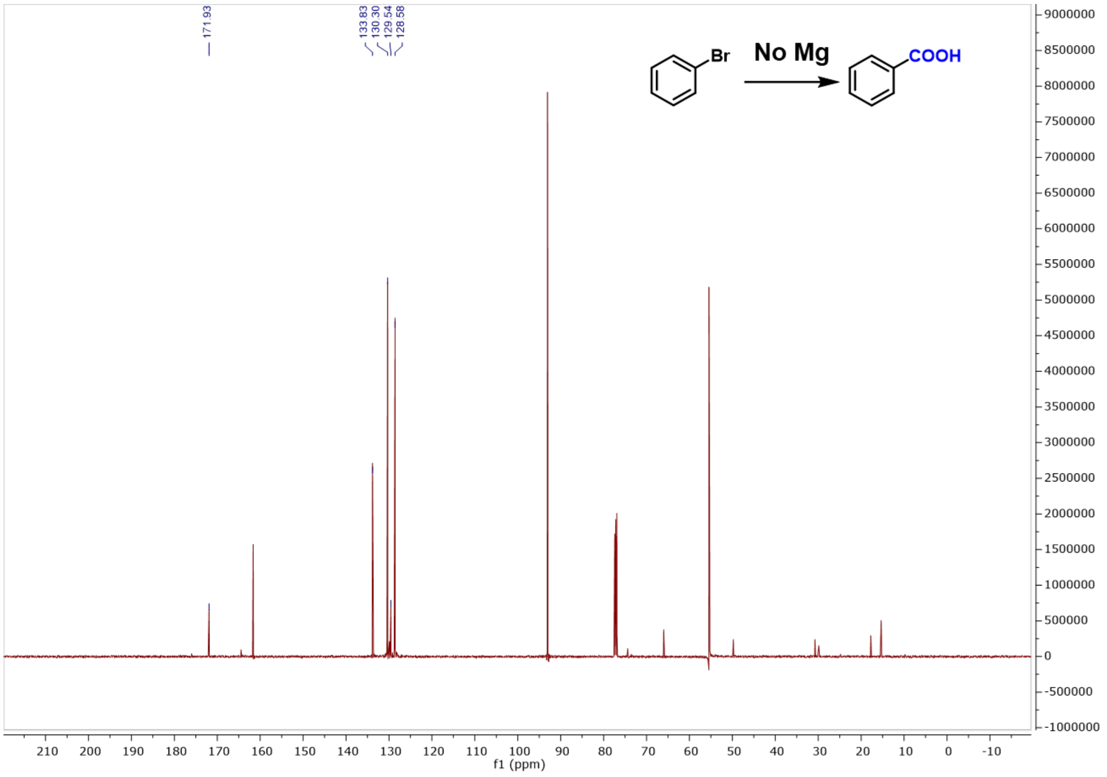
No Mg
Br
COOH
171.93
133.83
130.30
129.54
128.58
f1 (ppm)

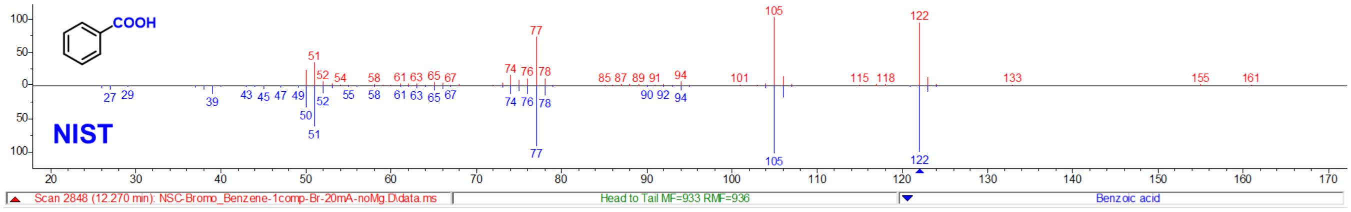
COOH
NIST
Scan 2848 (12.270 min): NSC-Bromo_Benzene-1comp-Br-20mA-noMg.D\data.ms
Head to Tail MF=933 RMF=936
Benzoic acid

13.4 Reactions Which Had Cell Leakage

5-Fluorovaleric acid

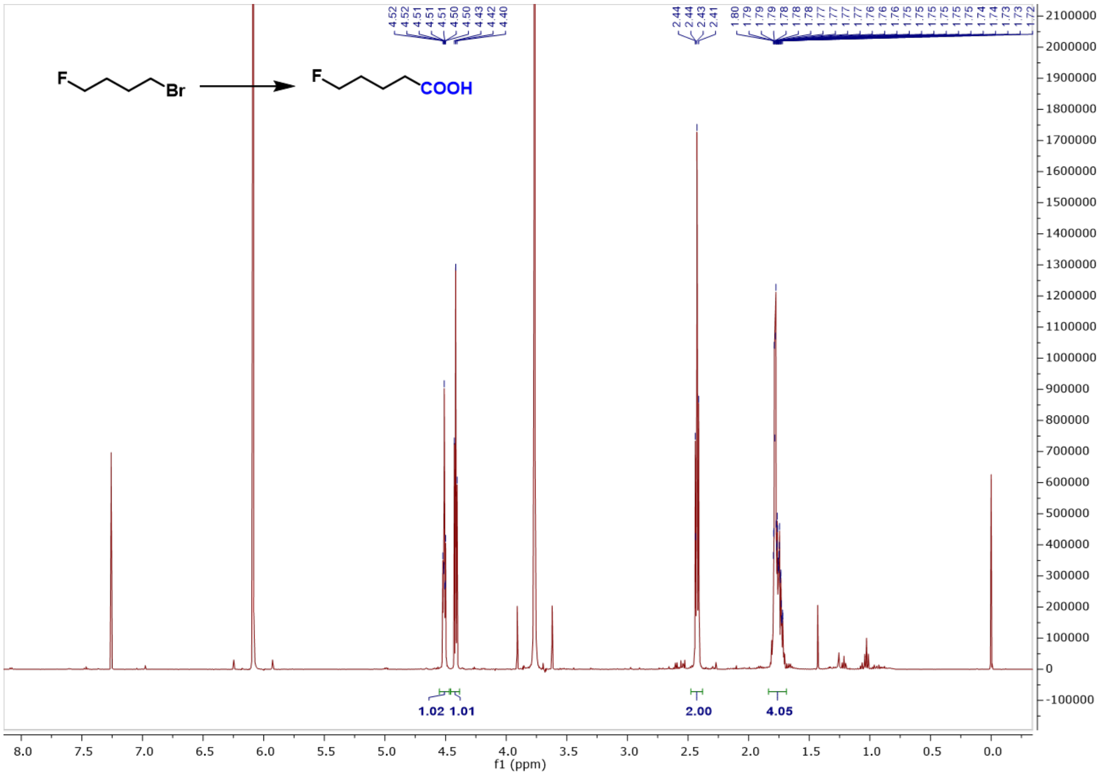

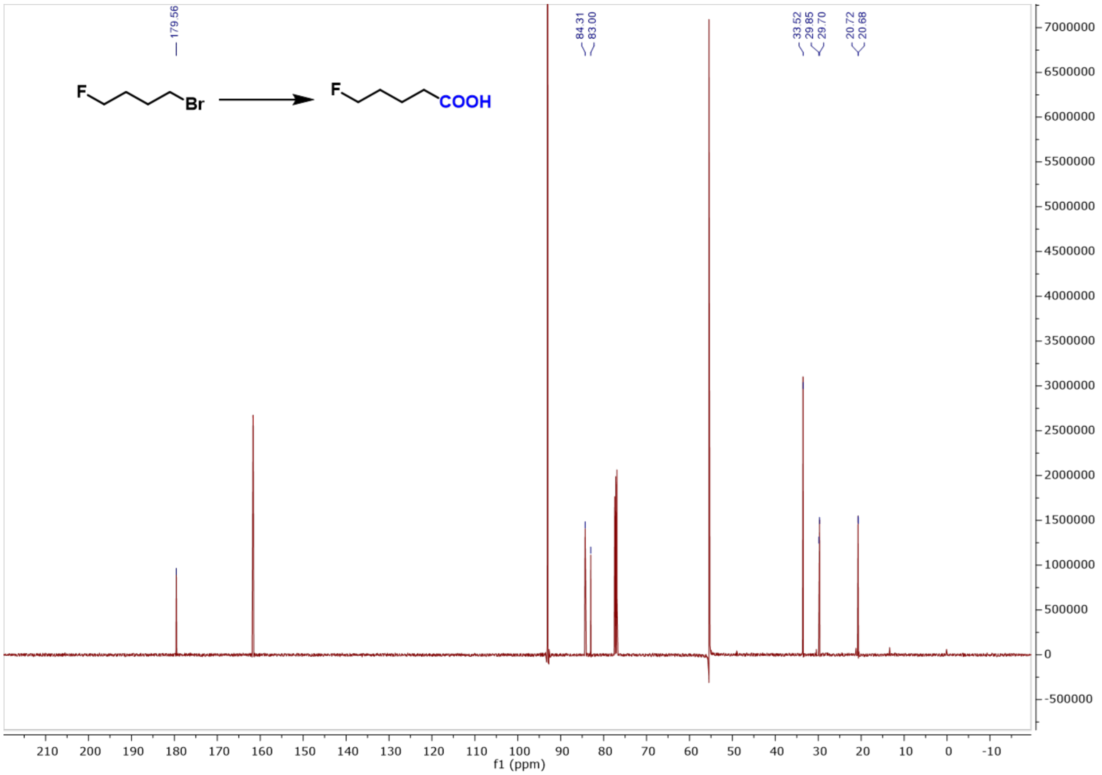

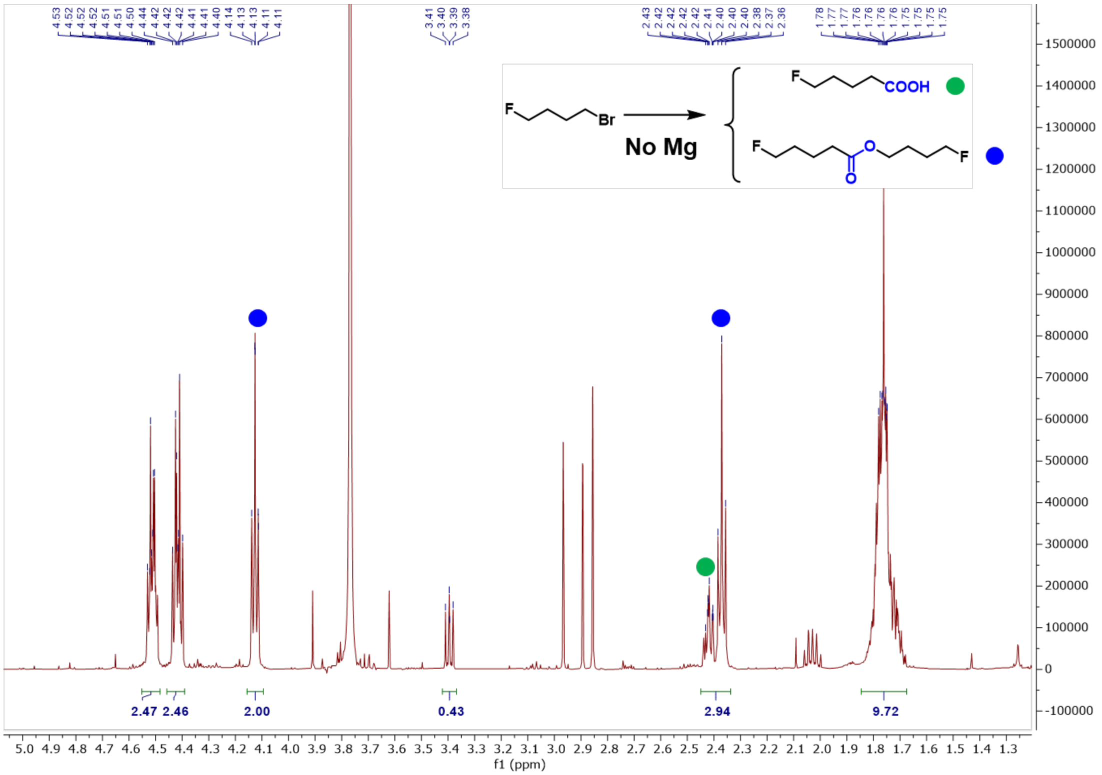

4.53 4.52 4.52 4.52 4.51 4.51 4.50 4.44 4.42 4.42 4.42 4.41 4.41 4.40 4.14 4.13 4.13 4.11 4.11
3.41 3.40 3.39 3.38
2.43 2.42 2.42 2.42 2.41 2.40 2.40 2.40 2.38 2.37 2.36
1.78 1.77 1.77 1.76 1.76 1.76 1.76 1.75 1.75 1.75 1.75
F
Br
No Mg
COOH
O
F
2.47 2.46
2.00
0.43
2.94
9.72
f1 (ppm)

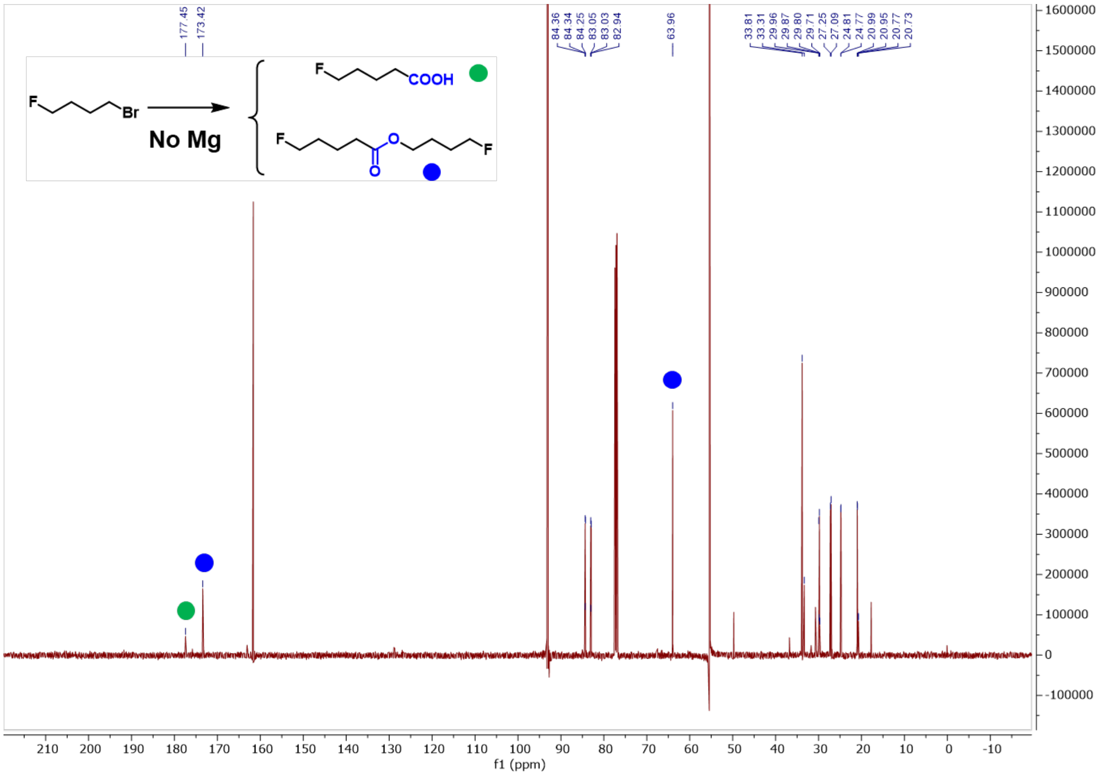

177.45
173.42
84.36 84.34 84.25 83.05 83.03 82.94
63.96
33.81 33.31 29.96 29.87 29.80 29.71 27.25 27.09 24.81 24.77 20.99 20.95 20.77 20.73
F
Br
No Mg
COOH
O
F
f1 (ppm)

Heptanoic acid

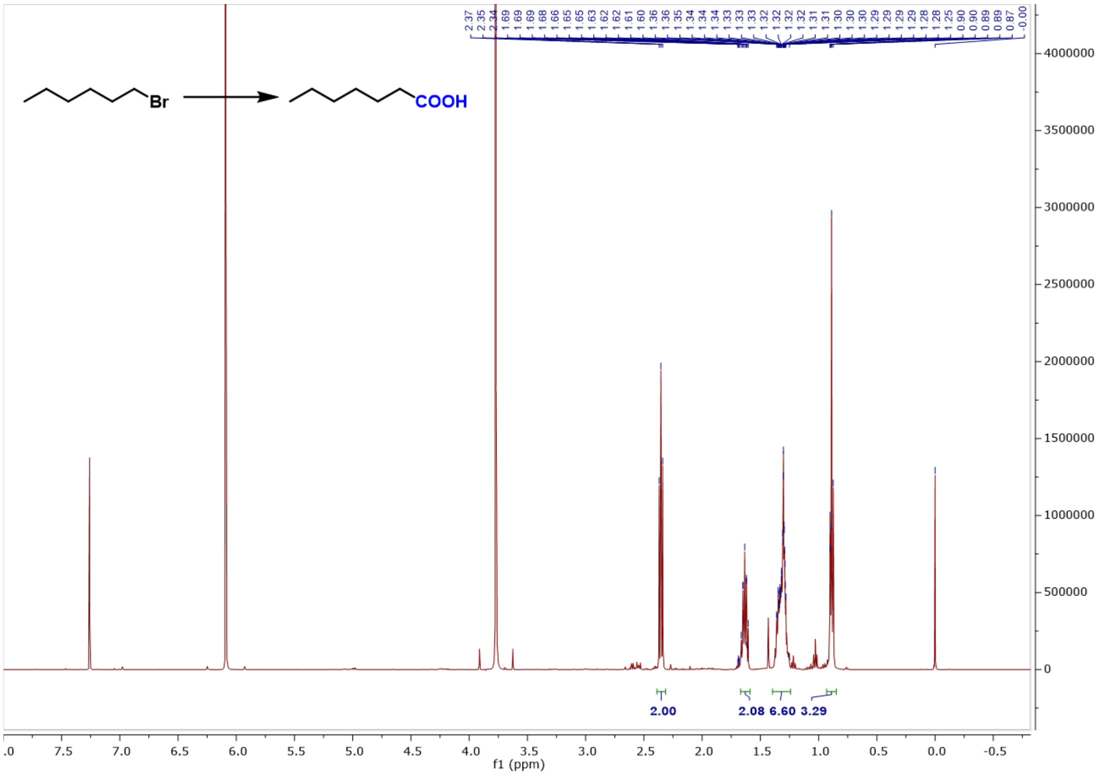

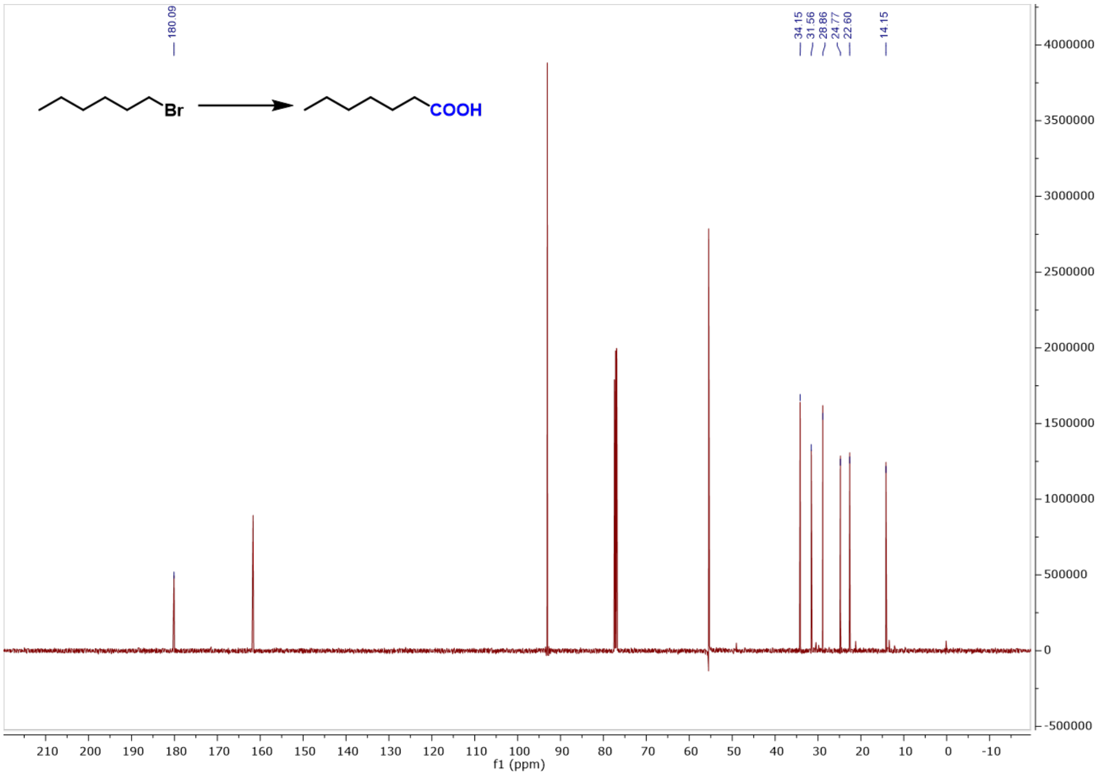

2-(4-Trifluoromethyl)phenyl)acetic acid

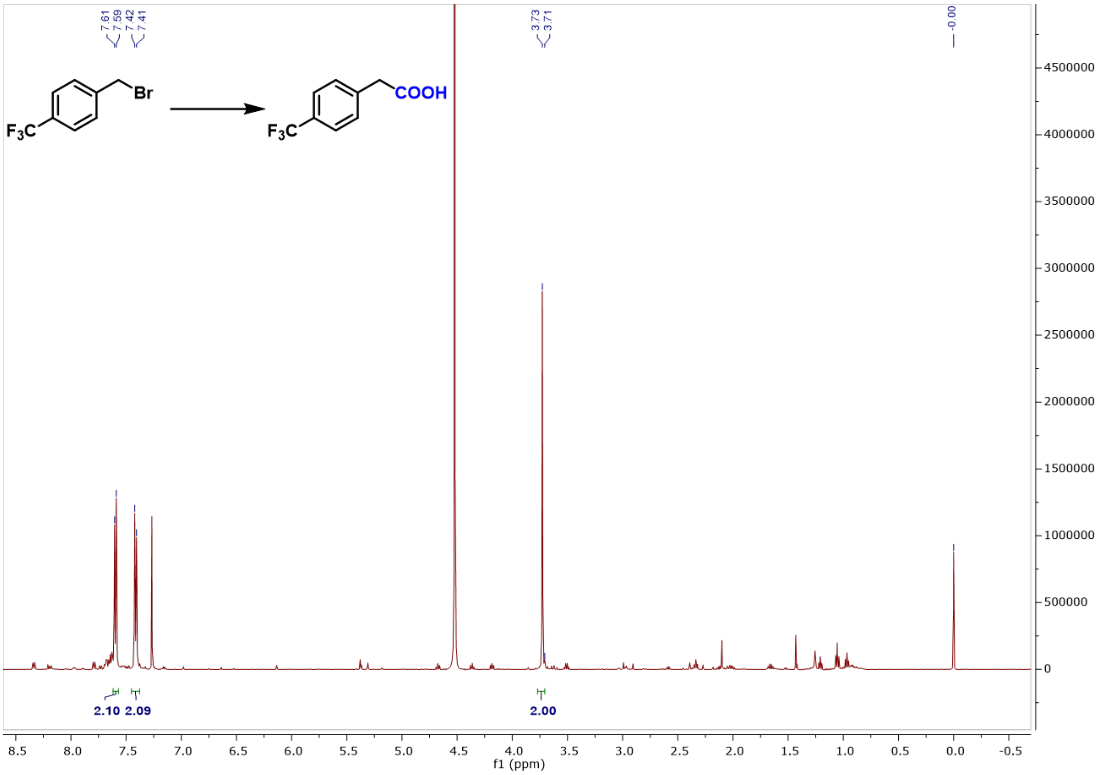

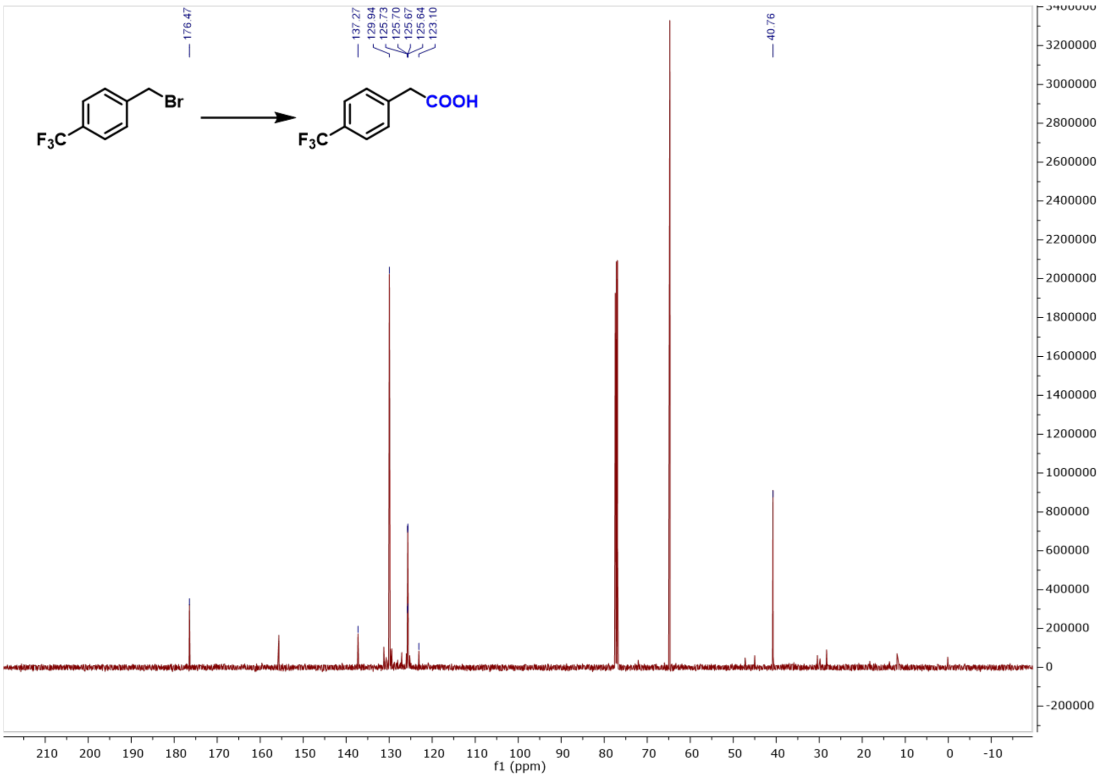

14 References

(1) Rablen, P. R.; McLarney, B. D.; Karlow, B. J.; Schneider, J. E. *J. Org. Chem.* **2014**, *79* (3), 867–879.

(2) Vermeeren, P.; Hansen, T.; Jansen, P.; Swart, M.; Hamlin, T. A.; Bickelhaupt, F. M. *Chem. – A Eur. J.* **2020**, *26* (67), 15538–15548.

(3) Brug, G. J.; van den Eeden, A. L. G.; Sluyters-Rehbach, M.; Sluyters, J. H. *J. Electroanal. Chem.* **1984**, *176* (1–2), 275–295.

(4) Devos, O.; Gabrielli, C.; Tribollet, B. *Electrochim. Acta* **2006**, *51* (8–9), 1413–1422.

(5) Lu, B.; Zhu, F.; Sun, H. M.; Shen, Q. *Org. Lett.* **2017**, *19* (5), 1132–1135.

(6) Sonawane, R. B.; Sonawane, S. R.; Rasal, N. K.; Jagtap, S. V. *Green Chem.* **2020**, *22* (10), 3186–3195.

(7) Zou, Q.; Long, G.; Zhao, T.; Hu, X. *Green Chem.* **2020**, *22* (4), 1134–1138.

(8) Bosset, C.; Beucher, H.; Bretel, G.; Pasquier, E.; Queguiner, L.; Henry, C.; Vos, A.; Edwards, J. P.; Meerpoel, L.; Berthelot, D. *Org. Lett.* **2018**, *20* (19), 6003–6006.

(9) Meng, S. S.; Lin, L. R.; Luo, X.; Lv, H. J.; Zhao, J. L.; Chan, A. S. C. *Green Chem.* **2019**, *21* (22), 6187–6193.

(10) Ojha, D. P.; Gadde, K.; Prabhu, K. R. *Org. Lett.* **2016**, *18* (19), 5062–5065.

(11) Cole, J. P.; Chen, D. F.; Kudisch, M.; Pearson, R. M.; Lim, C. H.; Miyake, G. M. *J. Am. Chem. Soc.* **2020**, *142* (31), 13573–13581.

(12) Jiang, X.; Zhang, J.; Ma, S. *J. Am. Chem. Soc.* **2016**, *138* (27), 8344–8347.

(13) Li, C.; Zhao, P.; Li, R.; Zhang, B.; Zhao, W. *Angew. Chem., Int. Ed.* **2020**, *59* (27), 10913–10917.

(14) Meng, Q. Y.; Wang, S.; König, B. *Angew. Chem., Int. Ed.* **2017**, *56* (43), 13426–13430.

(15) Ren, W.; Chu, J.; Sun, F.; Shi, Y. *Org. Lett.* **2019**, *21* (15), 5967–5970.

(16) Jung, H. Y.; Chang, S.; Hong, S. *Org. Lett.* **2019**, *21* (17), 7099–7103.

(17) Seo, H.; Liu, A.; Jamison, T. F. *J. Am. Chem. Soc.* **2017**, *139* (40), 13969–13972.

(18) Obst, M. F.; Gevorgyan, A.; Bayer, A.; Hopmann, K. H. *Organometallics* **2020**, *39* (9), 1545–1552.

(19) Liu, Y.; Xu, Y.; Jung, S. H.; Chae, J. *Synlett* **2012**, *23* (18), 2692–2698.

(20) Tan, X.; Song, T.; Wang, Z.; Chen, H.; Cui, L.; Li, C. *Org. Lett.* **2017**, *19* (7), 1634–1637.

(21) Moragas, T.; Gaydou, M.; Martin, R. *Angew. Chem., Int. Ed.* **2016**, *55* (16), 5053–5057.

(22) Yang, D. T.; Zhu, M.; Schiffer, Z. J.; Williams, K.; Song, X.; Liu, X.; Manthiram, K. *ACS Catal.* **2019**, *9* (5), 4699–4705.

(23) Xu, C.; Xu, J. *Org. Biomol. Chem.* **2019**, *18* (1), 127–134.

(24) Xinyuan, L.; Xiaoyang, D.; Yufeng, Z. Method for Catalytic Asymmetric Cross Coupling Synthesis of Alkyne. CN110627610, 2019.

(25) Ajvazi, N.; Stavber, S. *Tetrahedron Lett.* **2016**, *57* (22), 2430–2433.

(26) Bartalucci, N.; Bortoluzzi, M.; Zacchini, S.; Pampaloni, G.; Marchetti, F. *Dalt. Trans.* **2019**, *48* (5), 1574–1577.

(27) Wang, Y.; Ren, W.; Li, J.; Wang, H.; Shi, Y. *Org. Lett.* **2014**, *16* (22), 5960–5963.

(28) Yuan, Y. C.; Kamaraj, R.; Bruneau, C.; Labasque, T.; Roisnel, T.; Gramage-Doria, R. *Org. Lett.* **2017**, *19* (23), 6404–6407.

(29) Miura, T.; Funakoshi, Y.; Nakahashi, J.; Moriyama, D.; Murakami, M. *Angew. Chem., Int. Ed.* **2018**, *57* (47), 15455–15459.

(30) Saputra, M. A.; Forgey, R. L.; Henry, J. L.; Kartika, R. *Tetrahedron Lett.* **2015**, *56* (11), 1392–1396.

(31) Schoenberger, T.; Menges, S.; Bernstein, M. A.; Pérez, M.; Seoane, F.; Sýkora, S.; Cobas, C. *Anal. Chem.* **2016**, *88* (7), 3836–3843.

(32) Murata, T.; Hiyoshi, M.; Ratanasak, M.; Hasegawa, J. Y.; Ema, T. *Chem. Commun.* **2020**, *56* (43), 5783–5786.

(33) Jefferies, L. R.; Cook, S. P. *Org. Lett.* **2014**, *16* (7), 2026–2029.

(34) Lu, P.; Hou, T.; Gu, X.; Li, P. *Org. Lett.* **2015**, *17* (8), 1954–1957.

(35) Yamada, K.; Liu, J.; Kunishima, M. *Org. Biomol. Chem.* **2018**, *16* (35), 6569–6575.

(36) Lu, L.; Cheng, D.; Zhan, Y.; Shi, R.; Chiang, C. W.; Lei, A. *Chem. Commun.* **2017**, *53* (51), 6852–6855.

(37) Coulbeck, E.; Eames, J. *Tetrahedron Asymmetry* **2007**, *18* (19), 2313–2325.

(38) Li, J.; Wang, C.; Xue, D.; Wei, Y.; Xiao, J. *Green Chem.* **2013**, *15* (10), 2685.

(39) Fujii, I.; Semba, K.; Li, Q. Z.; Sakaki, S.; Nakao, Y. *J. Am. Chem. Soc.* **2020**, *142* (27), 11647–11652.

(40) Niu, T.; Chen, S.; Hong, M.; Zhang, T.; Chen, J.; Dong, X.; Ni, B. *Green Chem.* **2020**, *22* (15), 5042–5049.

(41) Hoffmann, M.; Chen, X.; Hirano, M.; Arimitsu, K.; Kimura, H.; Higuchi, T.; Decker, M. *ChemMedChem* **2018**, *13* (23), 2546–2557.

(42) Gaydou, M.; Moragas, T.; Juliá-Hernández, F.; Martin, R. *J. Am. Chem. Soc.* **2017**, *139* (35), 12161–12164.
